# Supplementary figures and images for: An individualized causal framework for learning intercellular communication networks that define microenvironments of individual tumors
Source: PLoS Comput Biol. 2022 Dec 22;18(12):e1010761. doi: 10.1371/journal.pcbi.1010761 (PMC9822106; doi:10.1371/journal.pcbi.1010761)

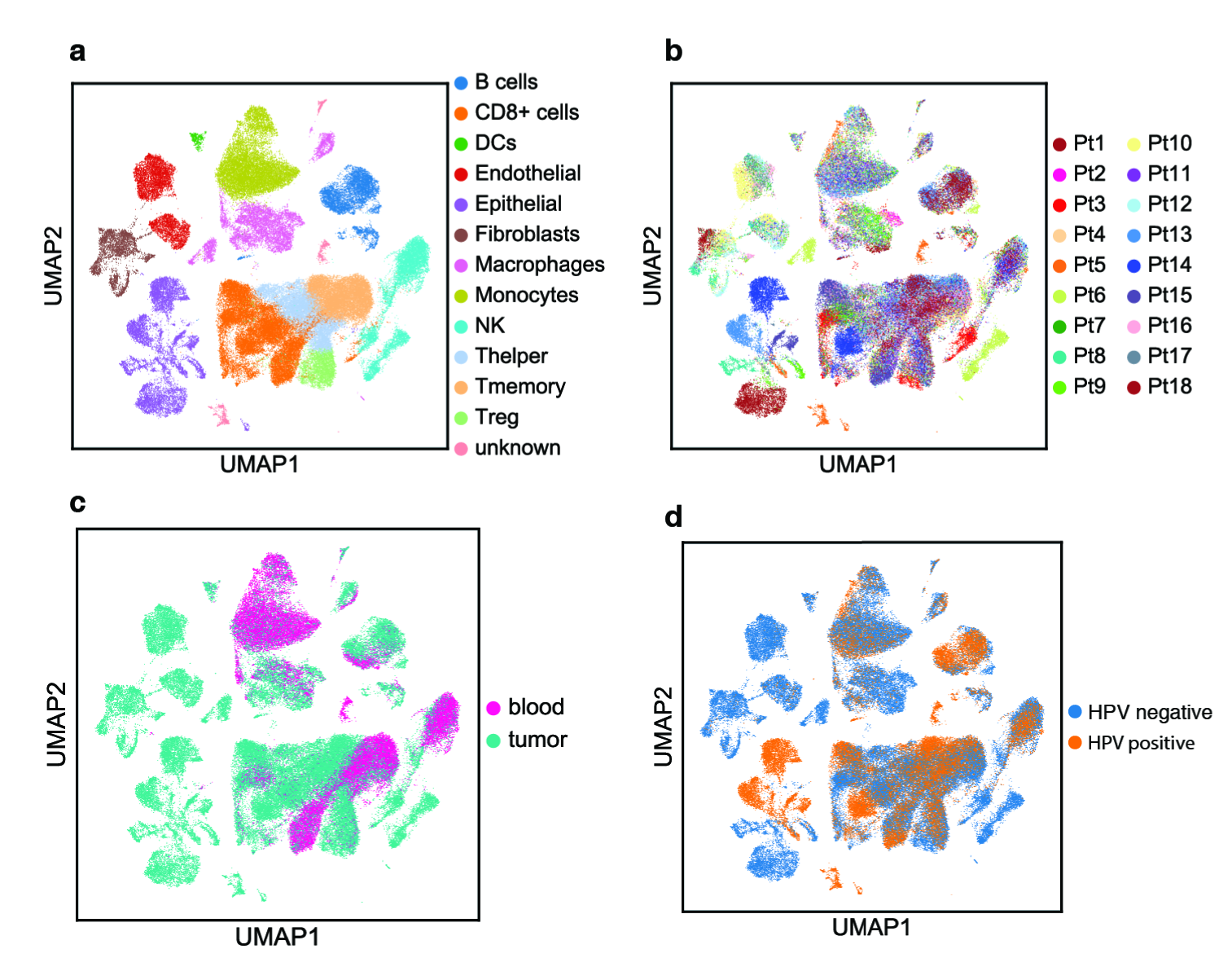

Supplement: S1 Fig — a. UMAP visualization of major types of cells. b. Visualization of cells from different tumors in UMAP 2-D space. C. Distribution of cells from tumors and peripheral blood. D. Distribution of cells from tumors with different human papillomavirus status. (TIF) [file pcbi.1010761.s001.tif]

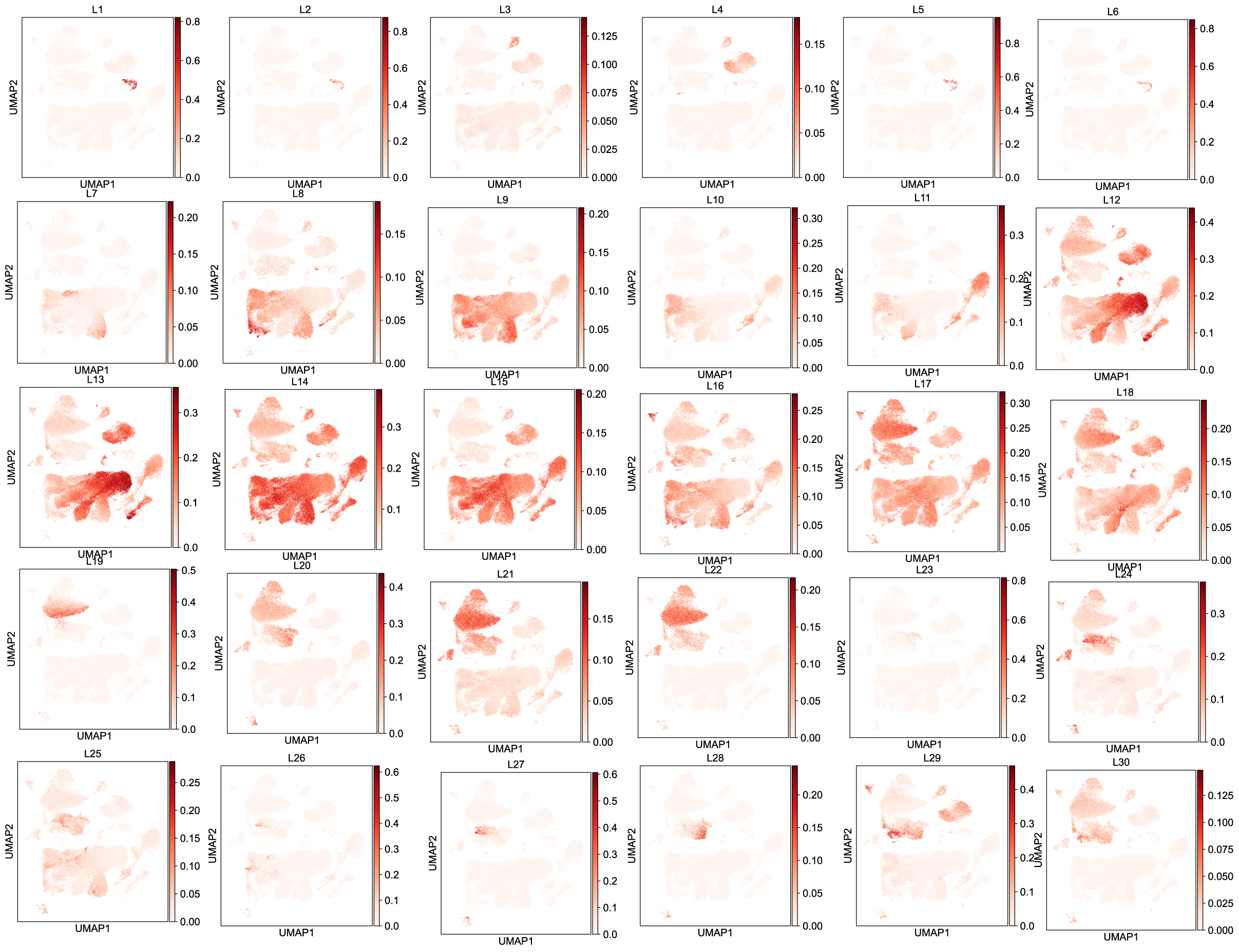

Supplement: S3 Fig — (TIF) [file pcbi.1010761.s003.tif]

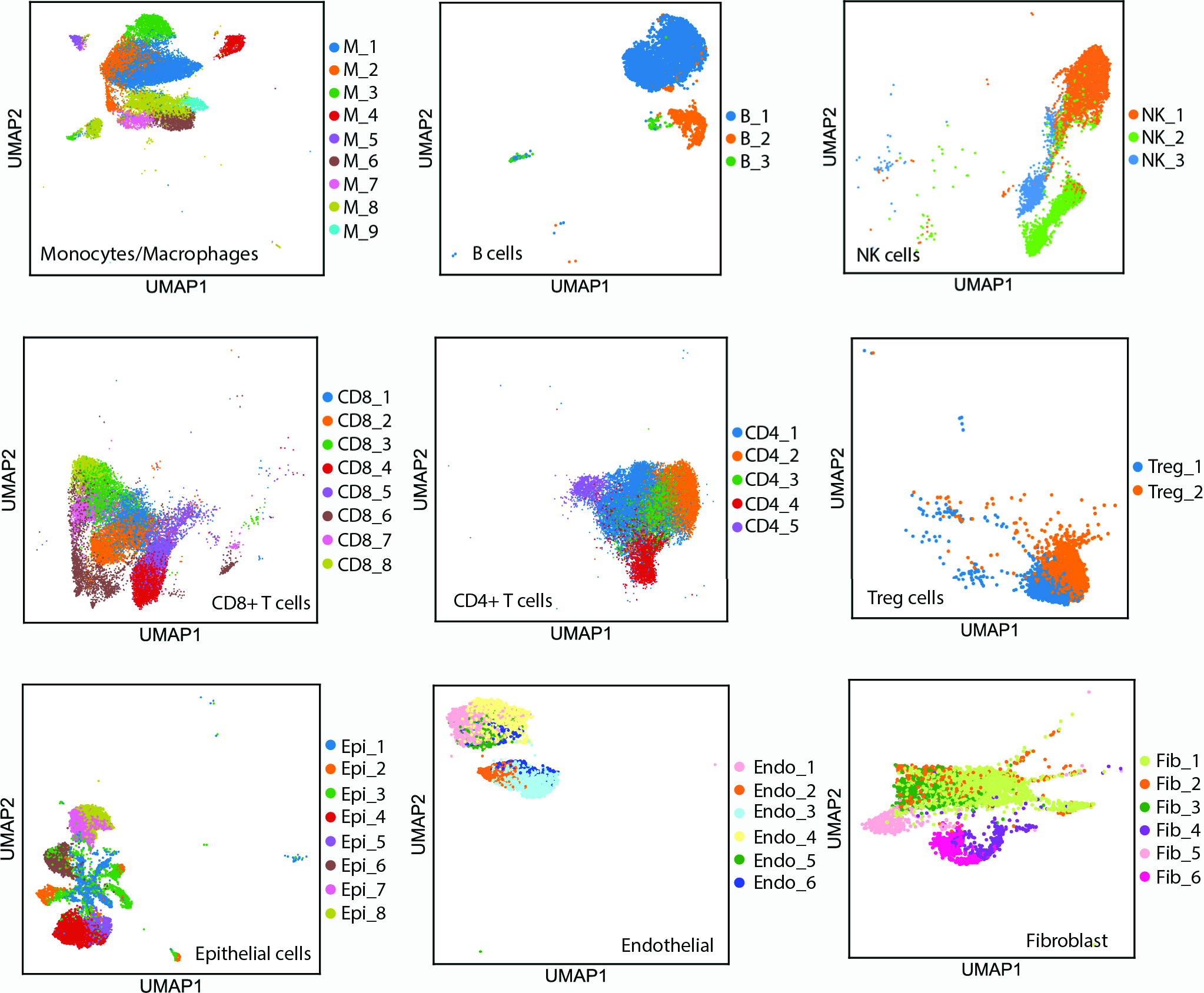

Supplement: S4 Fig — Cells were first divided into 9 major categories based on the expression of markers. Within each major category, cells were represented in the GEM space and analyzed using consensus clustering. Each block shows the subtyping within one of 9 major categories. (TIF) [file pcbi.1010761.s004.tif]

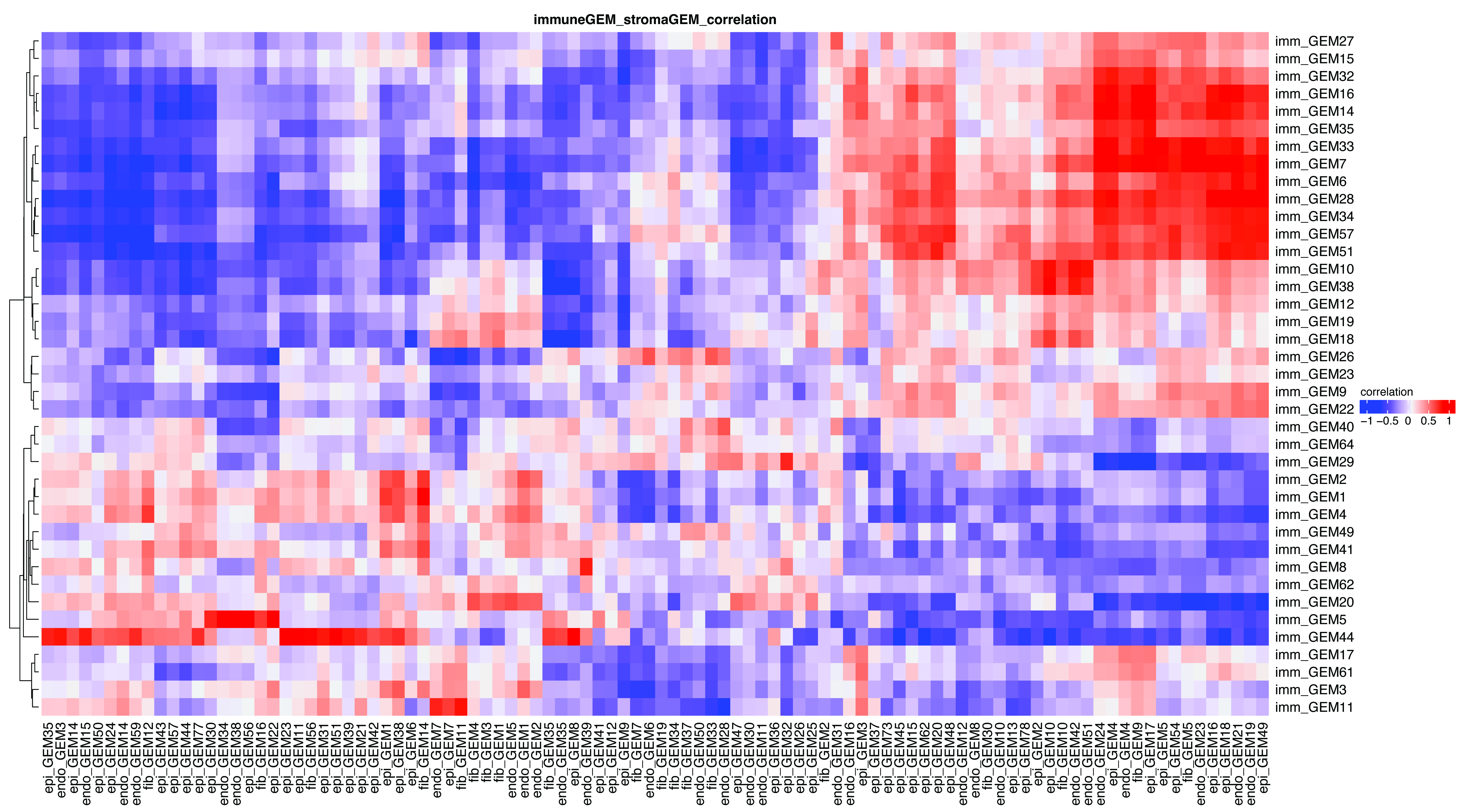

Supplement: S5 Fig — Value of correlation coefficients are color-coded. (TIF) [file pcbi.1010761.s005.tif]

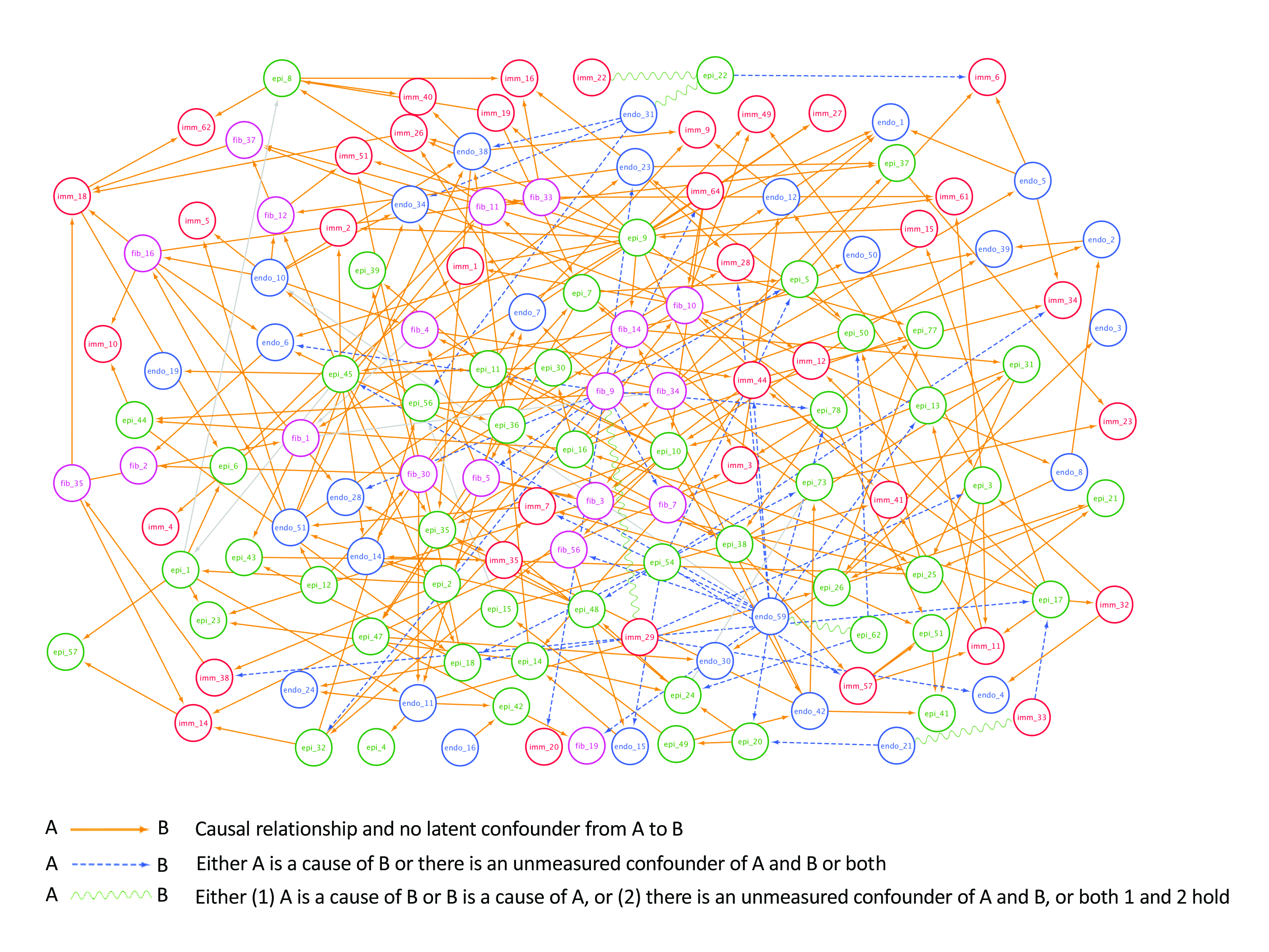

Supplement: S6 Fig — (TIF) [file pcbi.1010761.s006.tif]

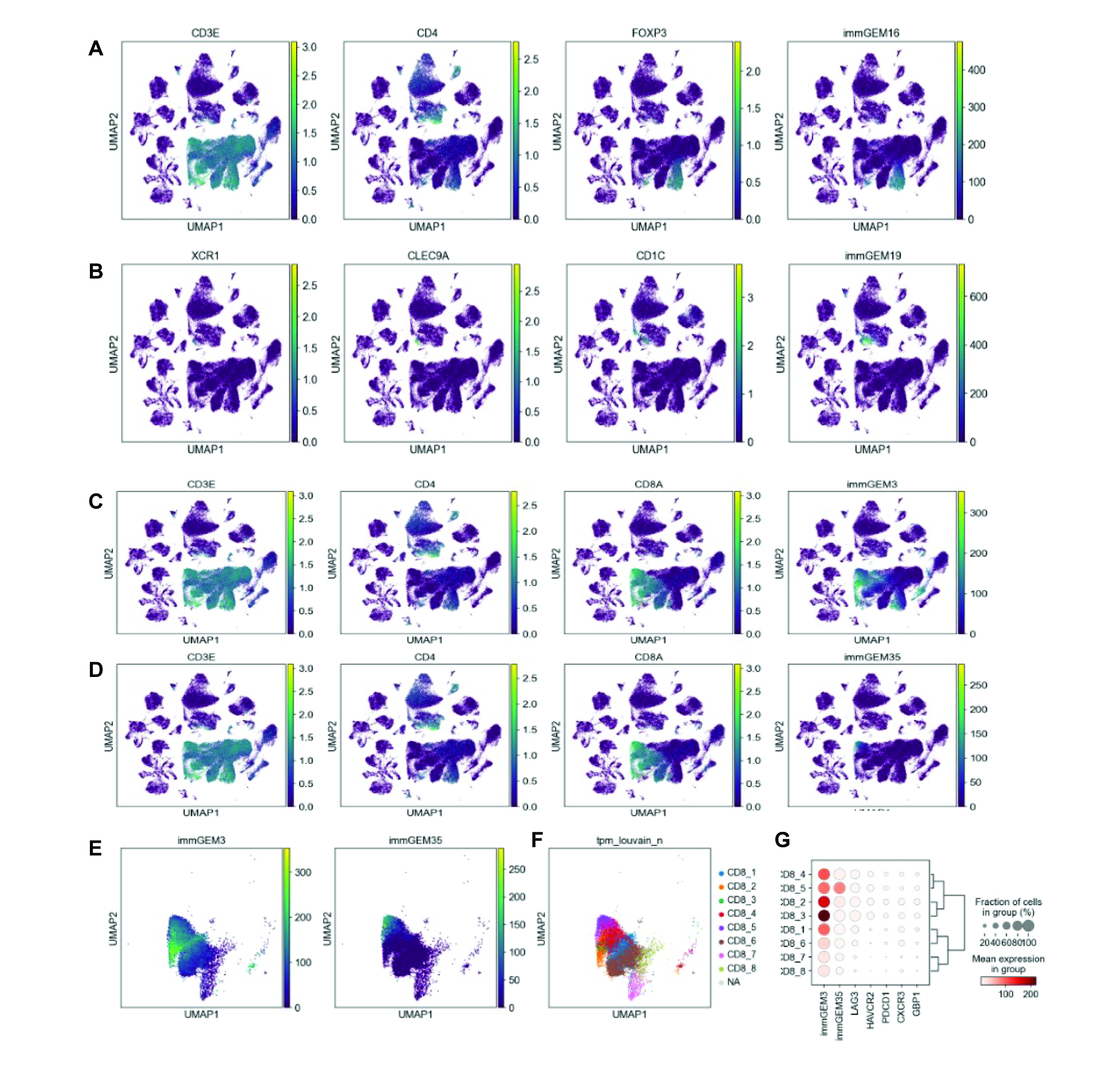

Supplement: S7 Fig — A. UMAP projections of Treg markers (CD3E, CD4, FOXP3) and immGEM16. B. UMAP projections of dendric cell markers (XCR1, CLEC9A, CD1C) and immGEM19. C & D. UMAP projection of CD8 cell markers (CD3E, CD4, CD8A), immGEM3 and immGEM35 respectively. E. UMAP projections comparing distributions of immGEM3 and immGEM35 among CD8+ cells. F. CD8+ cell clusters. G. Dot plot show the enrichment of immGEM3 and immGEM35 among the subtypes of CD8+ cells. (TIF) [file pcbi.1010761.s007.tif]

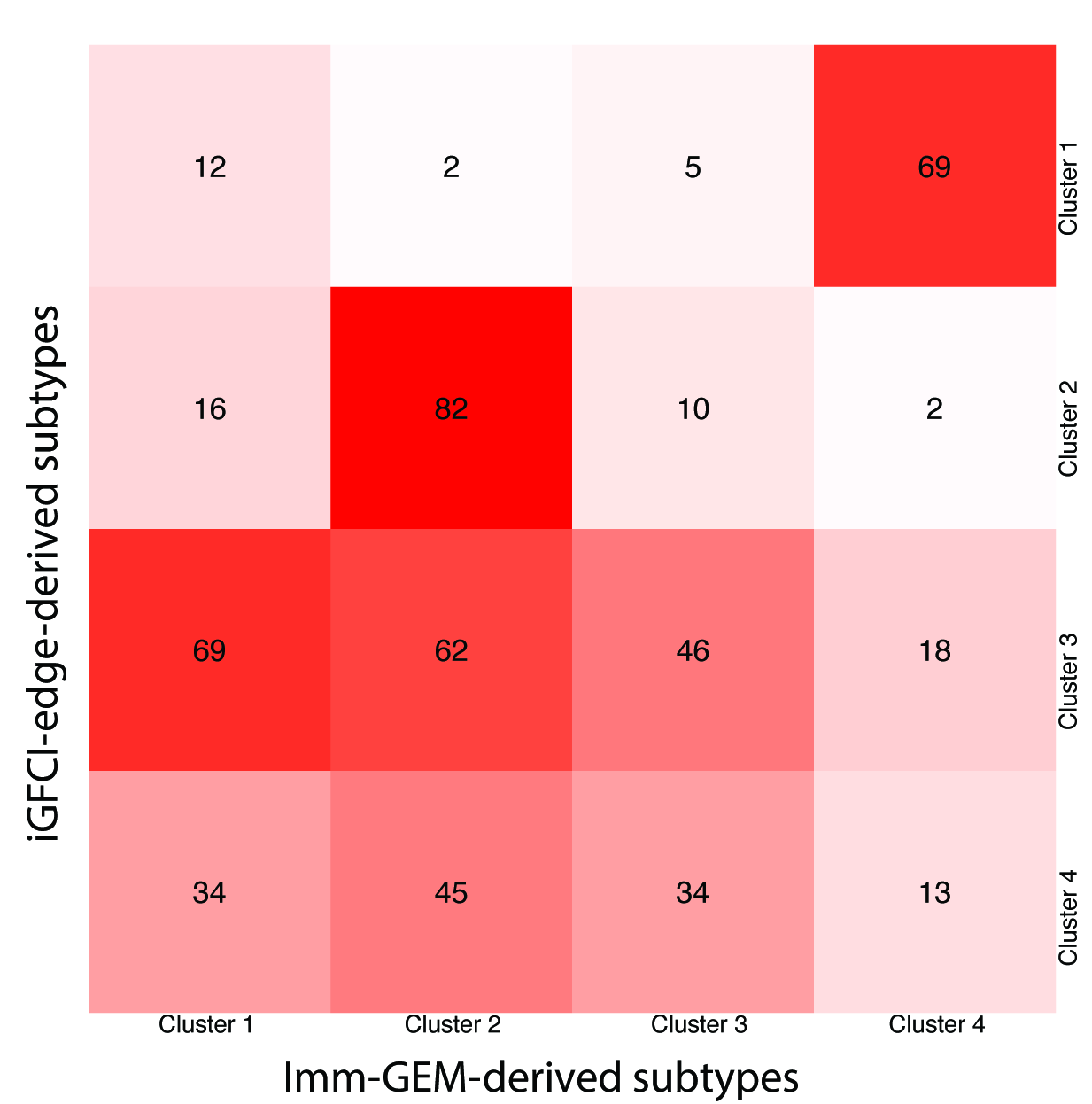

Supplement: S8 Fig — (TIF) [file pcbi.1010761.s008.tif]
