# Supplementary material for: An individualized causal framework for learning intercellular communication networks that define microenvironments of individual tumors
Source: PLoS Comput Biol. 2022 Dec 22;18(12):e1010761. doi: 10.1371/journal.pcbi.1010761 (PMC9822106; doi:10.1371/journal.pcbi.1010761)

Imm GEM 1

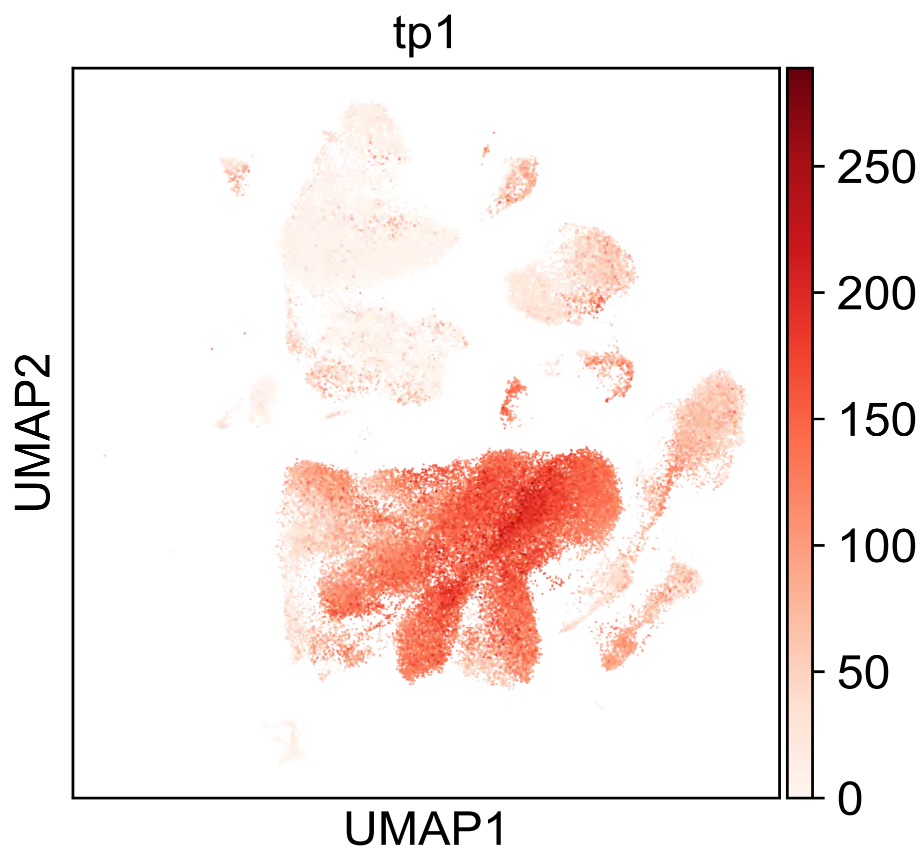

Cell Type

CD4 T cells

CD8 T cells

Tregs

NK cells

B cells

Top 20 genes

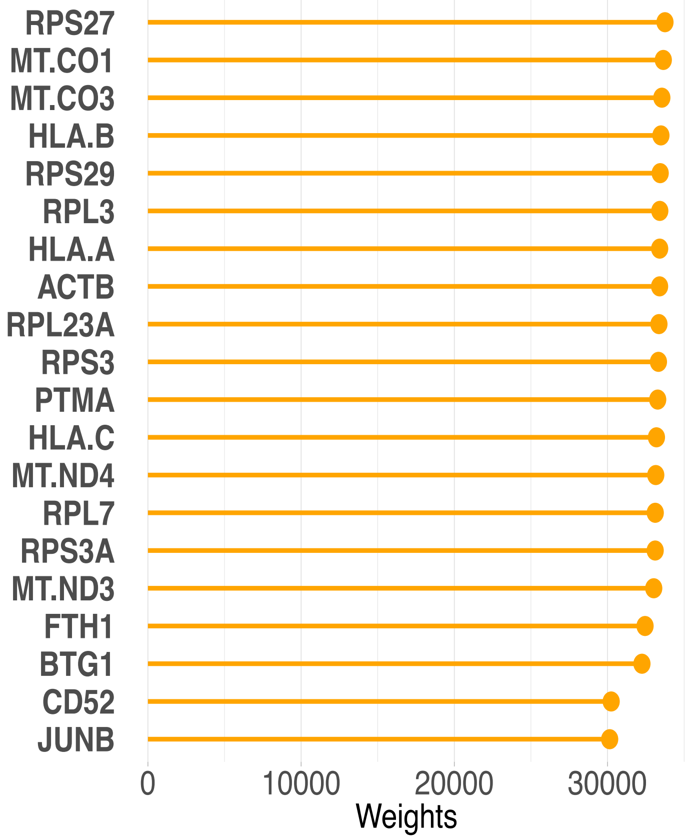

Imm GEM 2

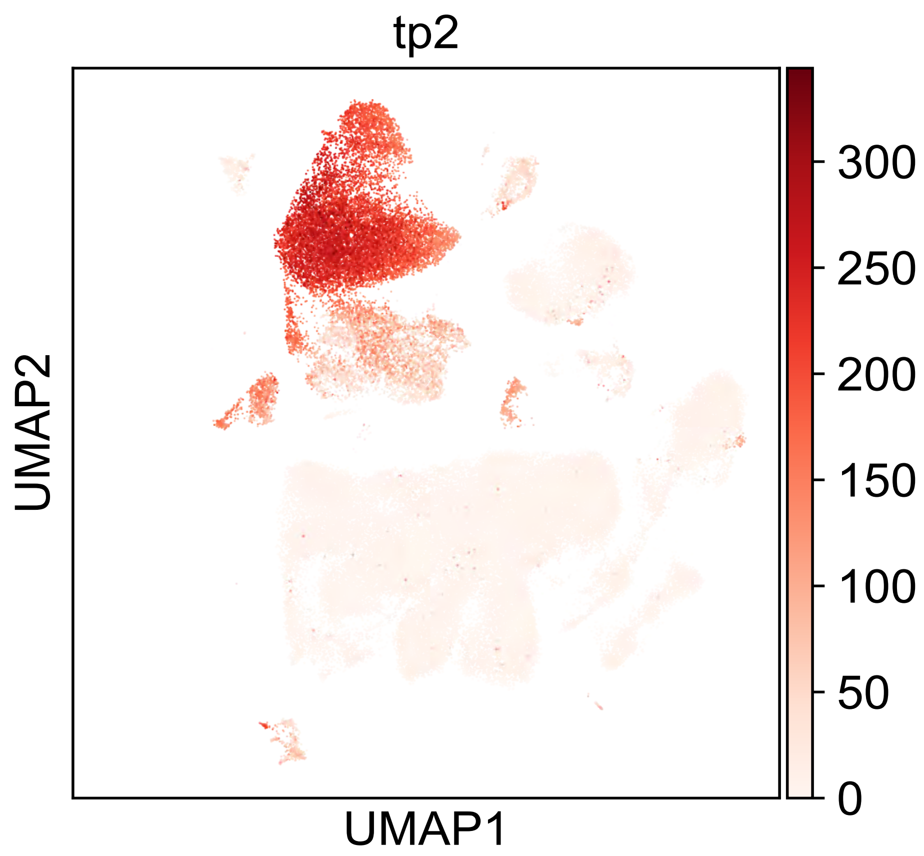

Cell Type

Monocytes

Macrophage

Top 20 genes

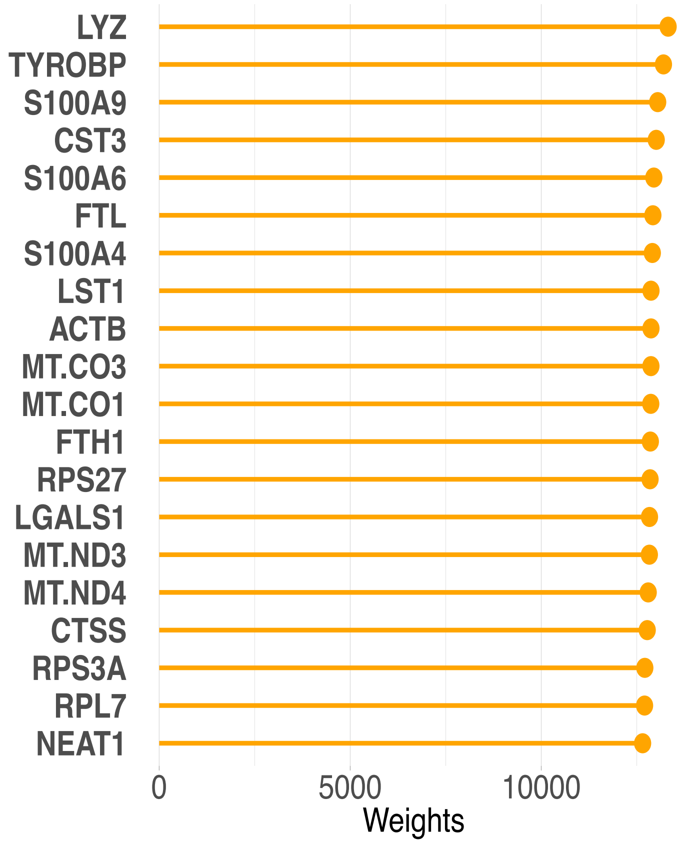

Imm GEM 3

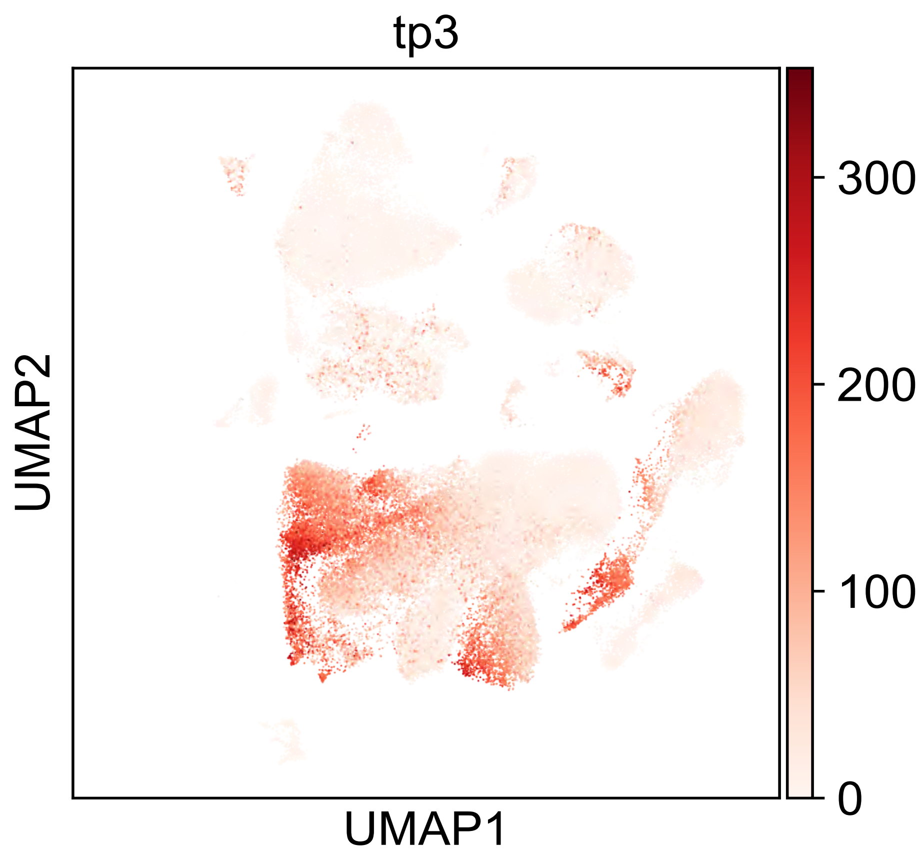

Cell Type

CD8 T cells

CD4 T cells

Tregs

NK cells

Top 20 genes

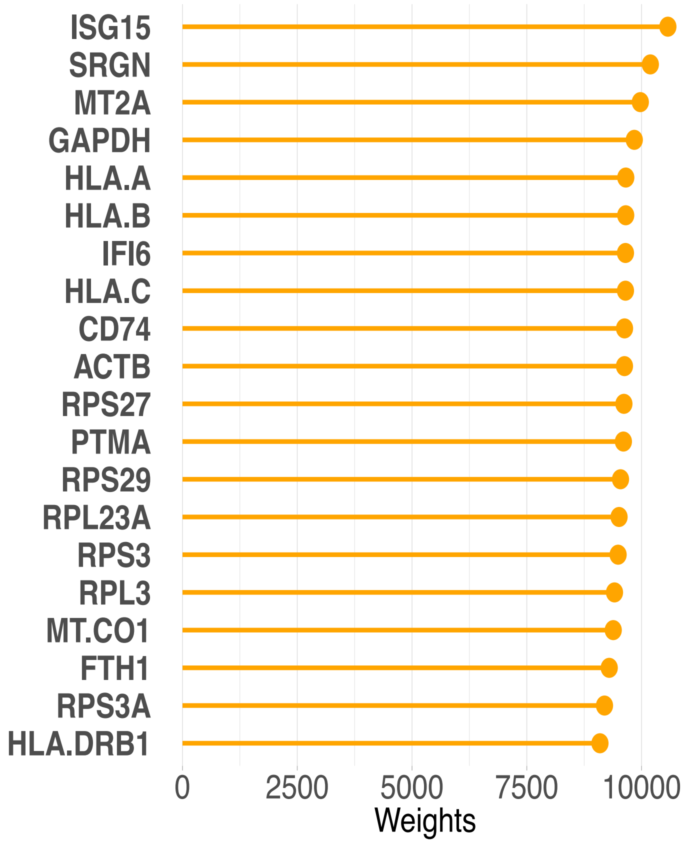

Imm GEM 4

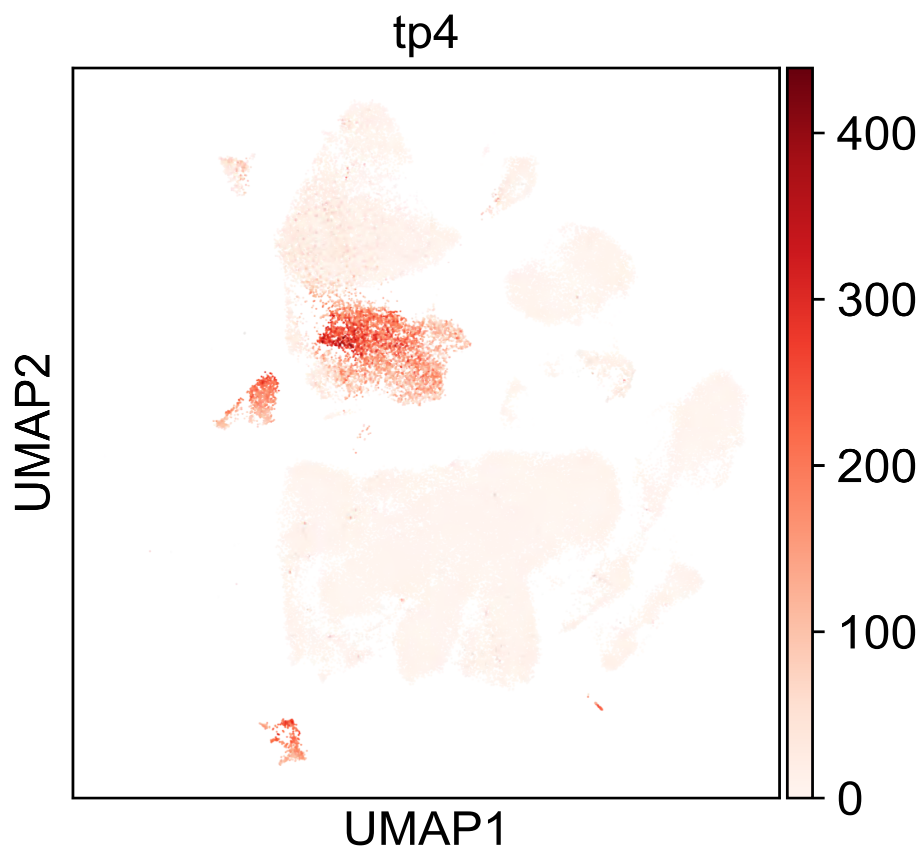

Cell Type

Macrophage

unknown

Top 20 genes

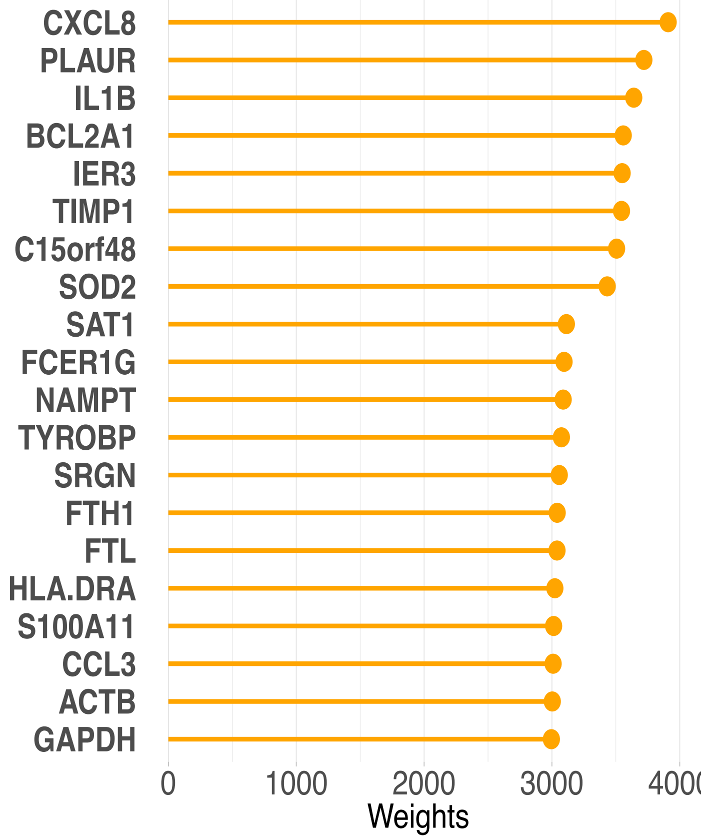

Imm GEM 5

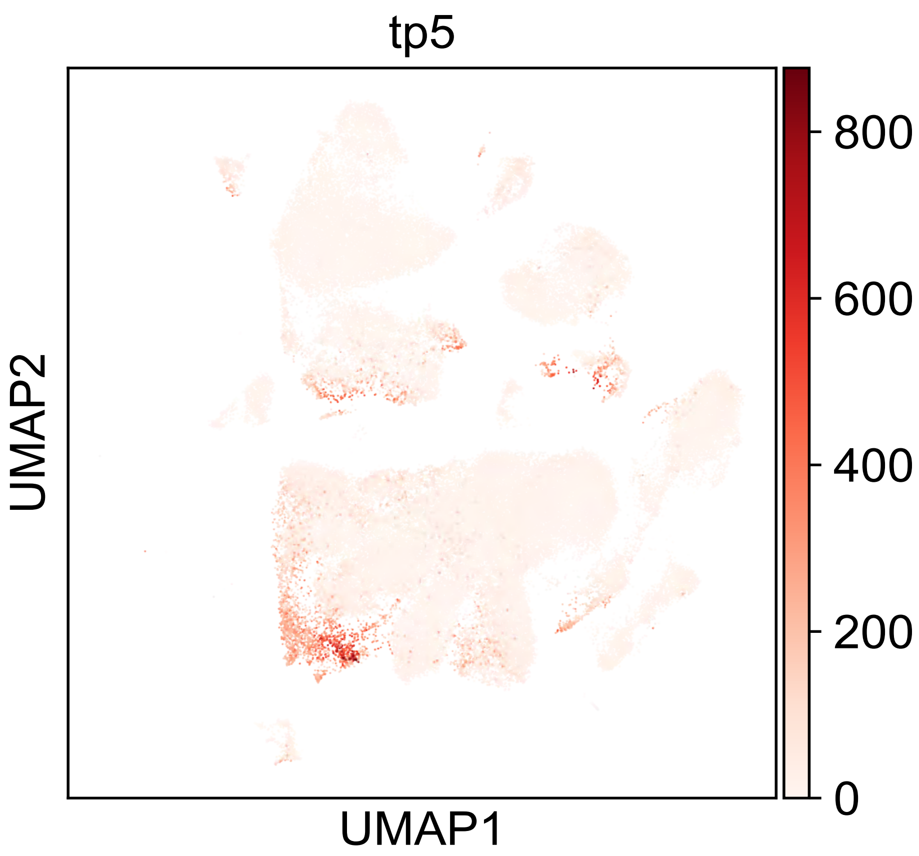

Cell Type

CD8 T cells

Top 20 genes

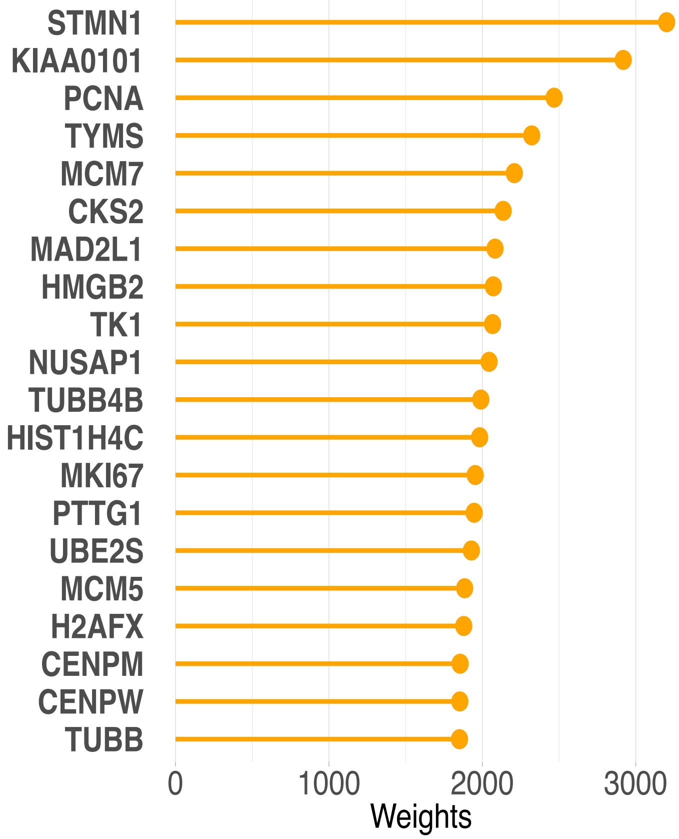

Imm GEM 6

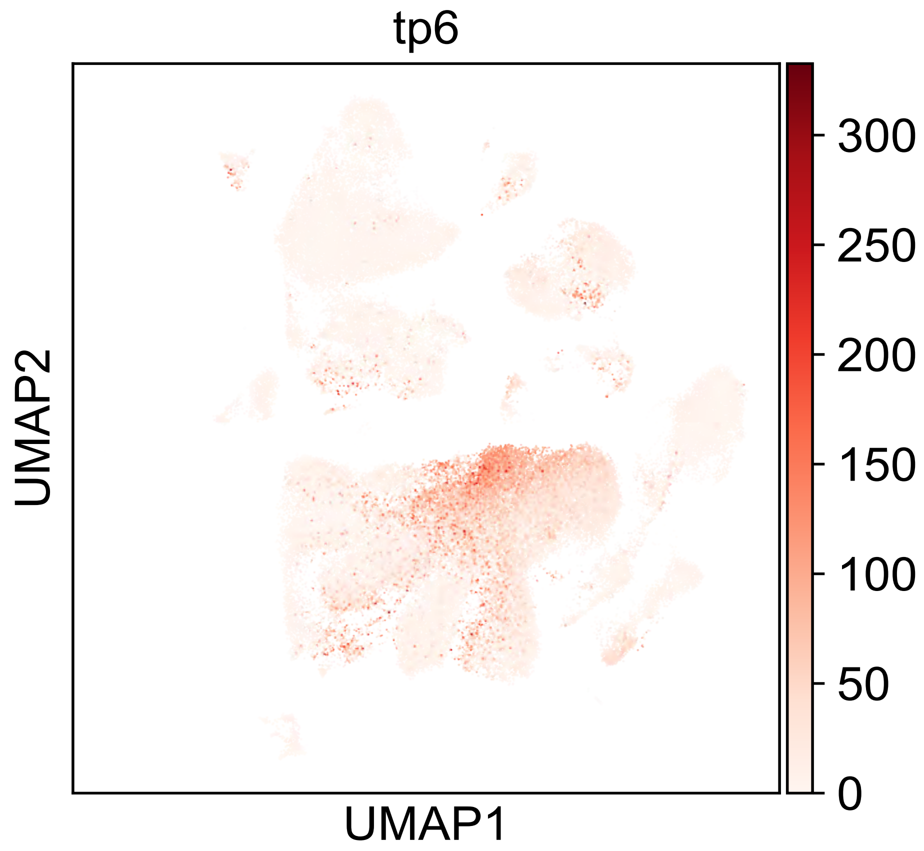

Cell Type

CD4 T cells

Top 20 genes

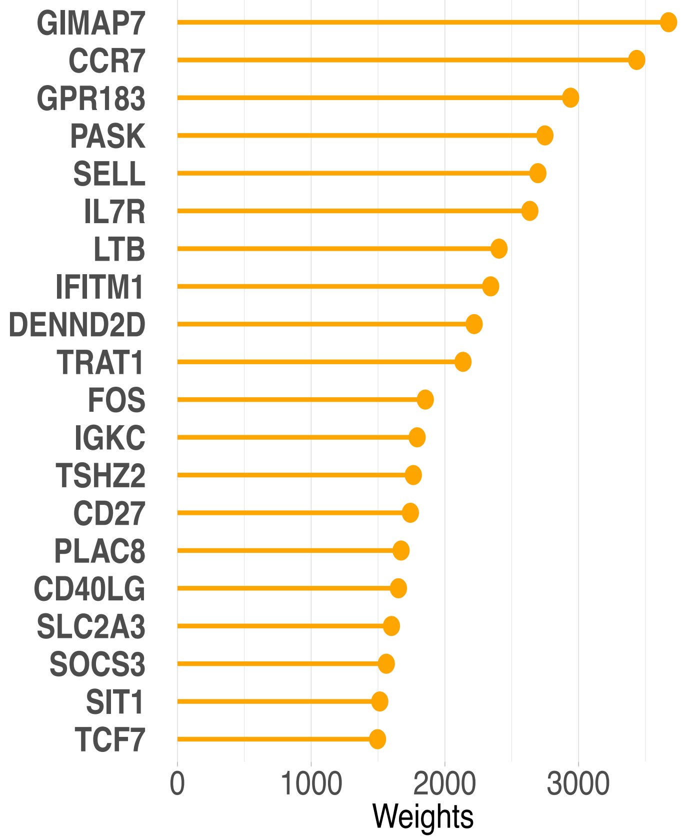

Imm GEM 7

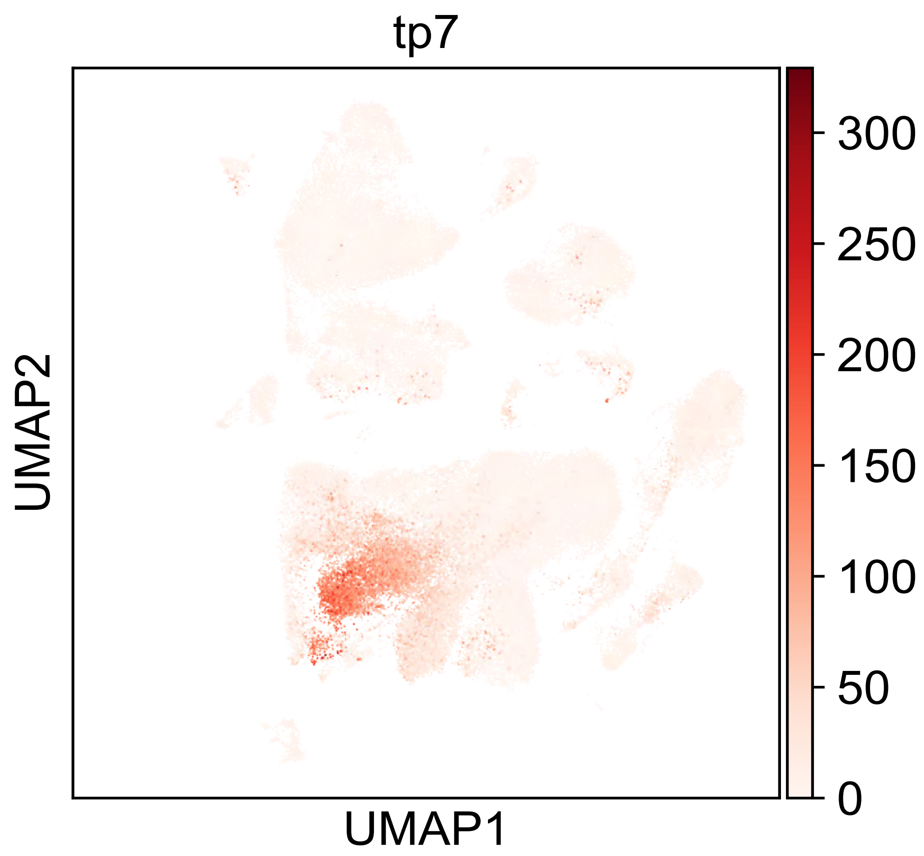

Cell Type

CD8 T cells

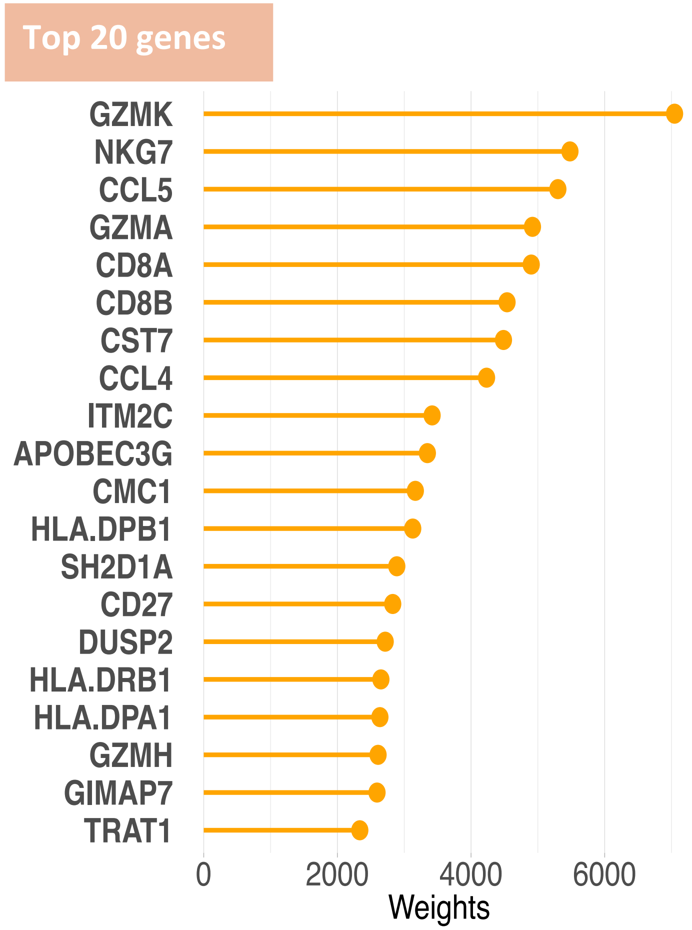

Imm GEM 8

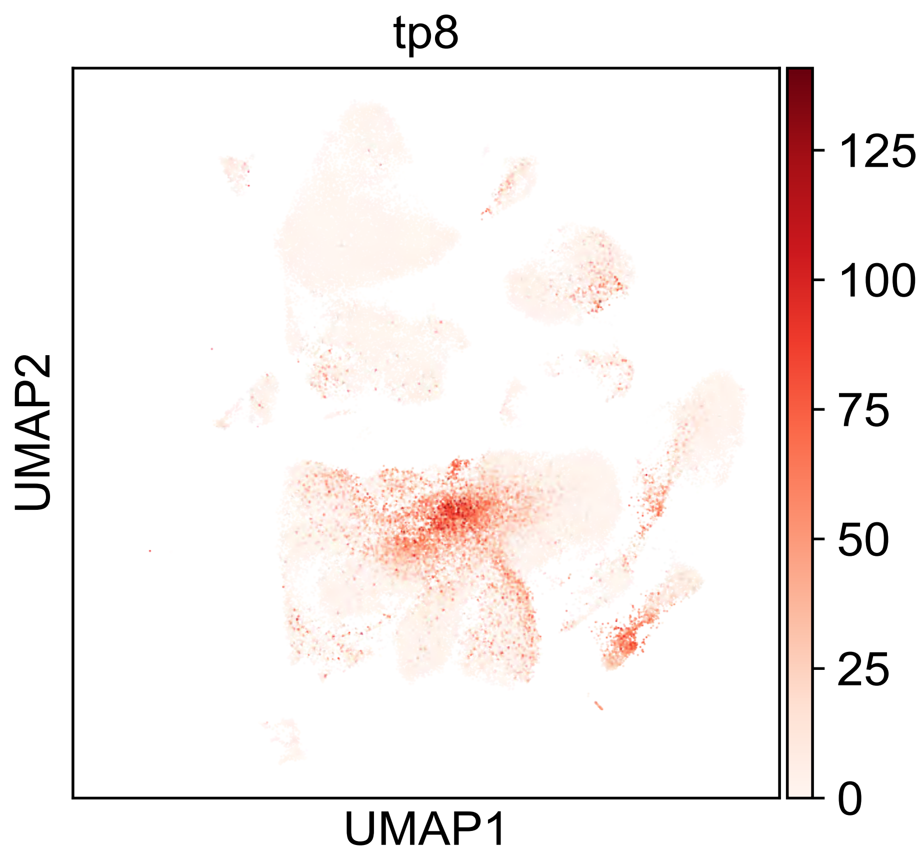

Cell Type

CD4 T cells

NK cells

Top 20 genes

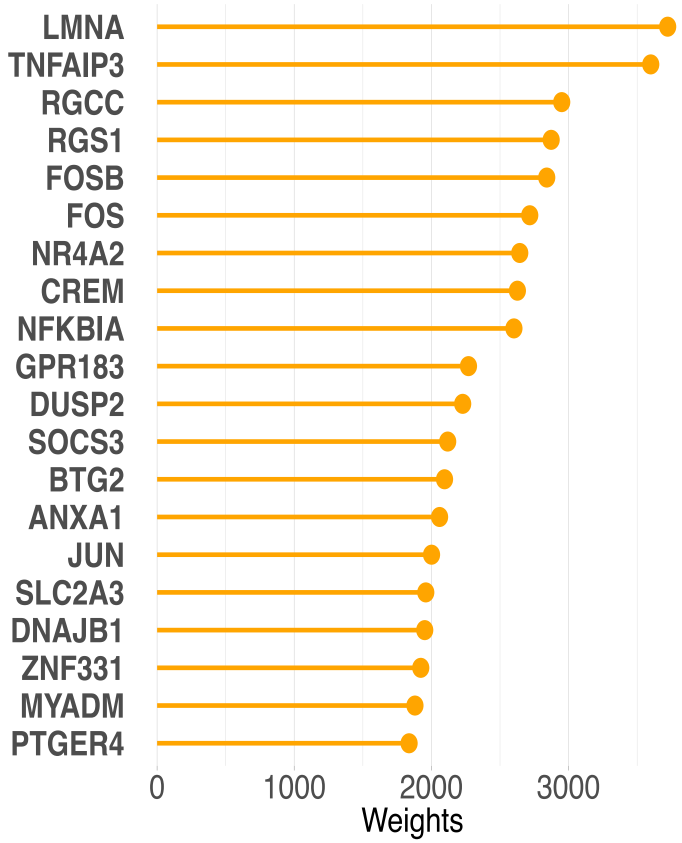

Imm GEM 9

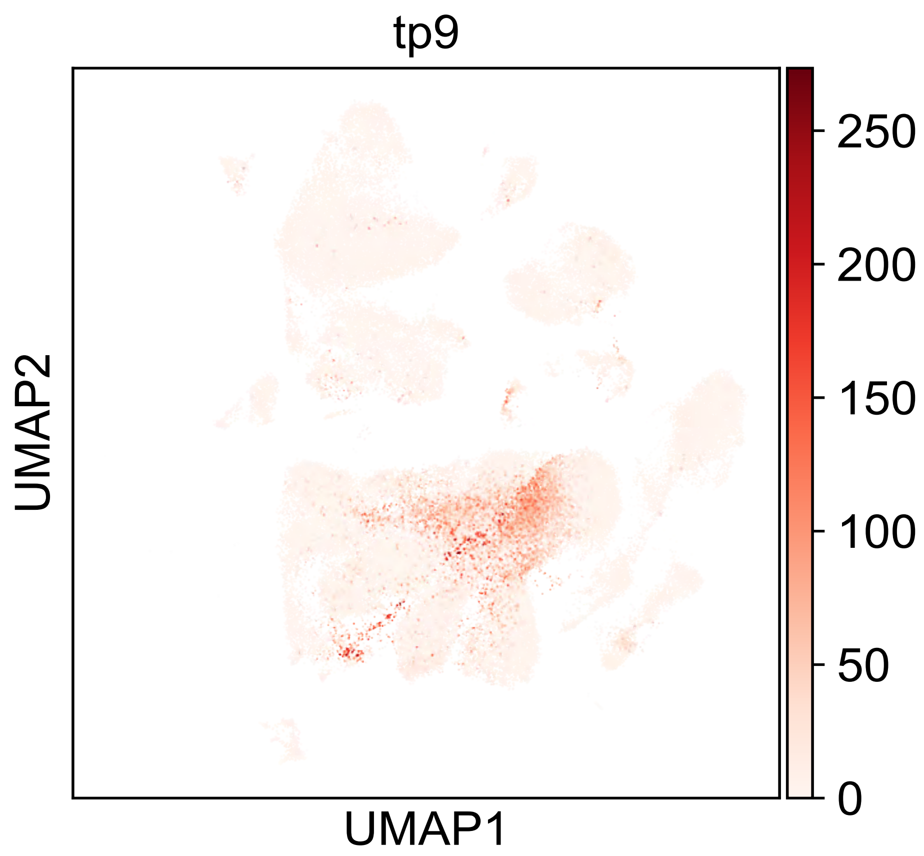

Cell Type

CD4 T cells

CD8 T cells

Top 20 genes

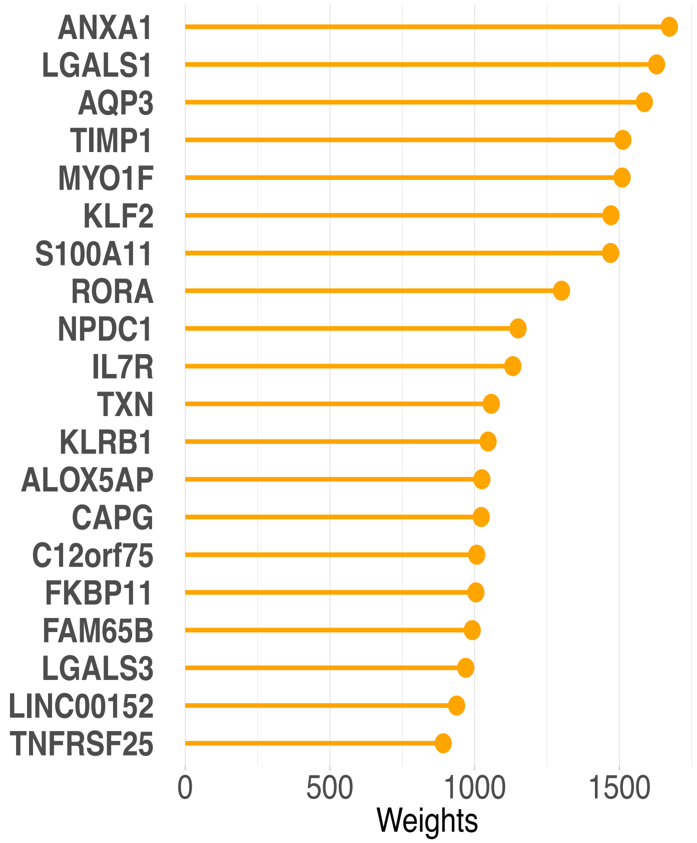

Imm GEM 10

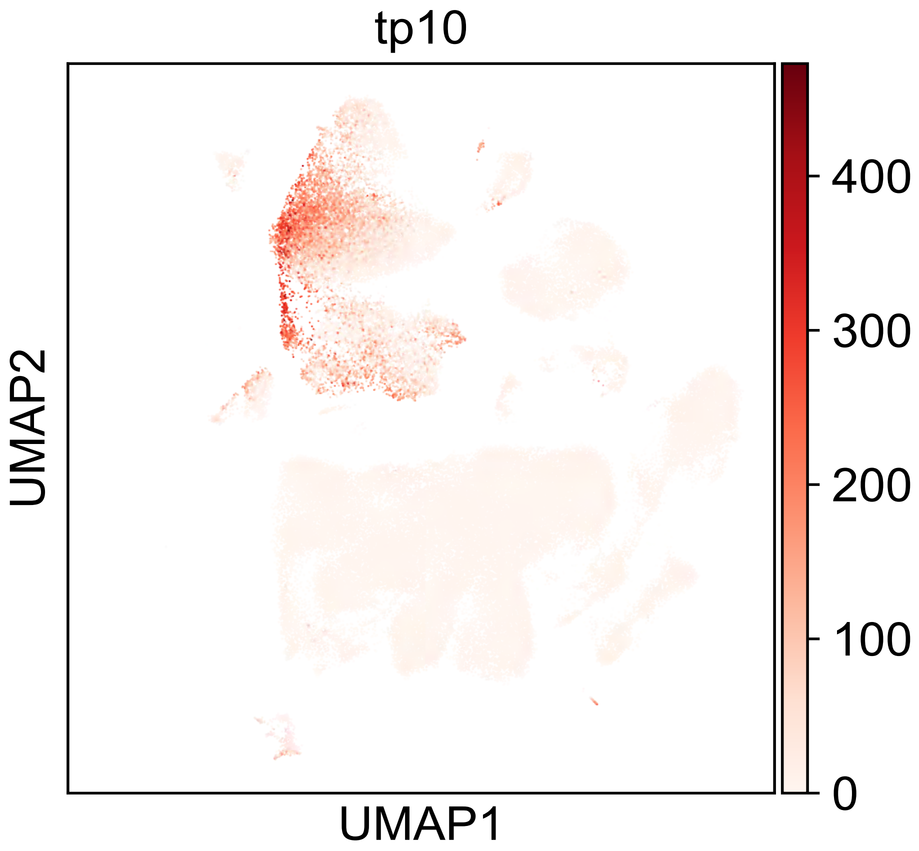

Cell Type

Monocytes

Macrophage

Top 20 genes

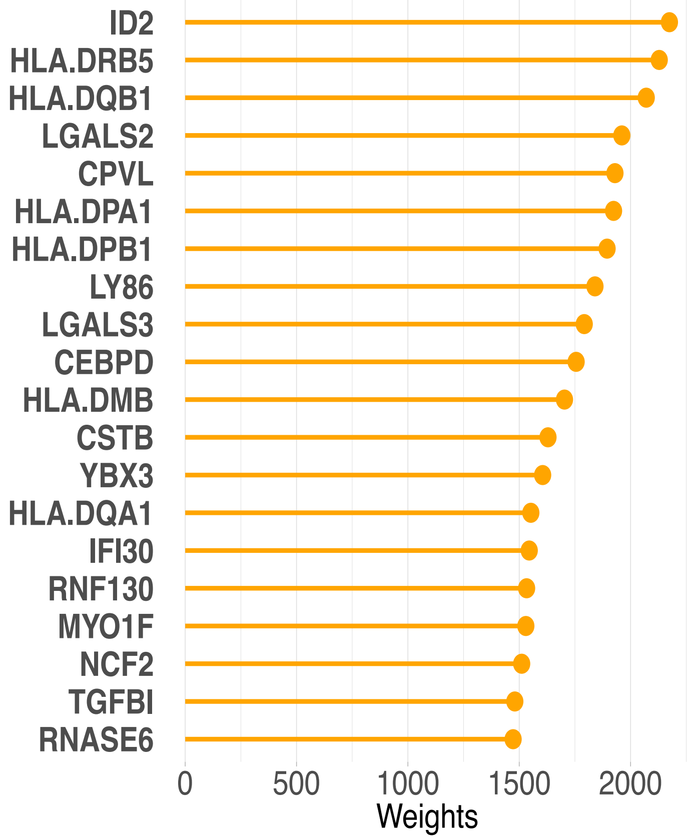

Imm GEM 11

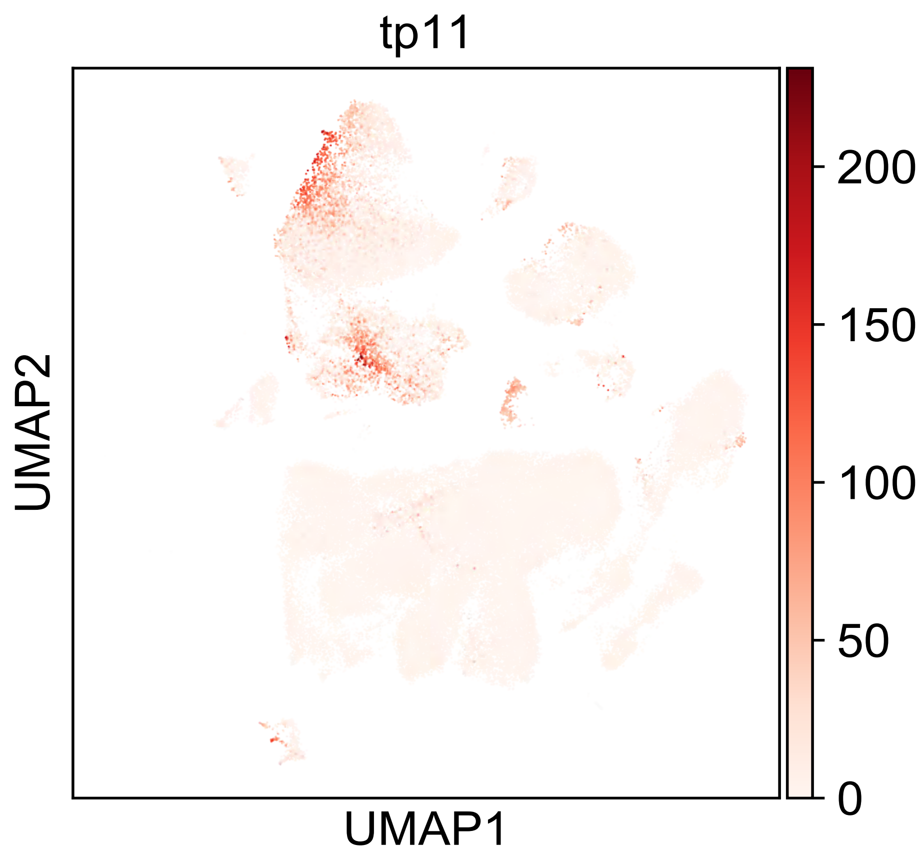

Cell Type

Monocytes

Macrophage

Top 20 genes

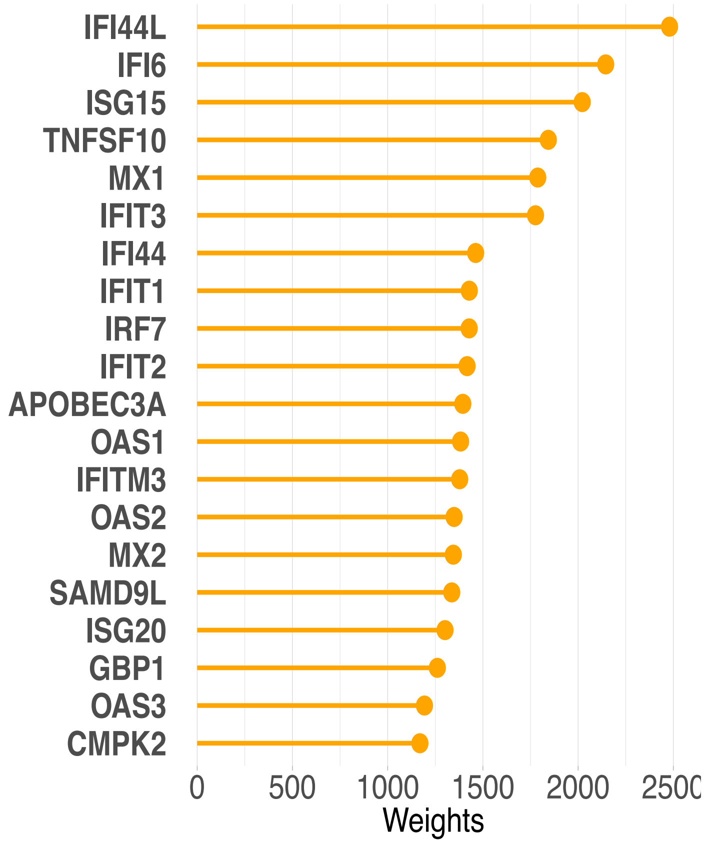

Imm GEM 12

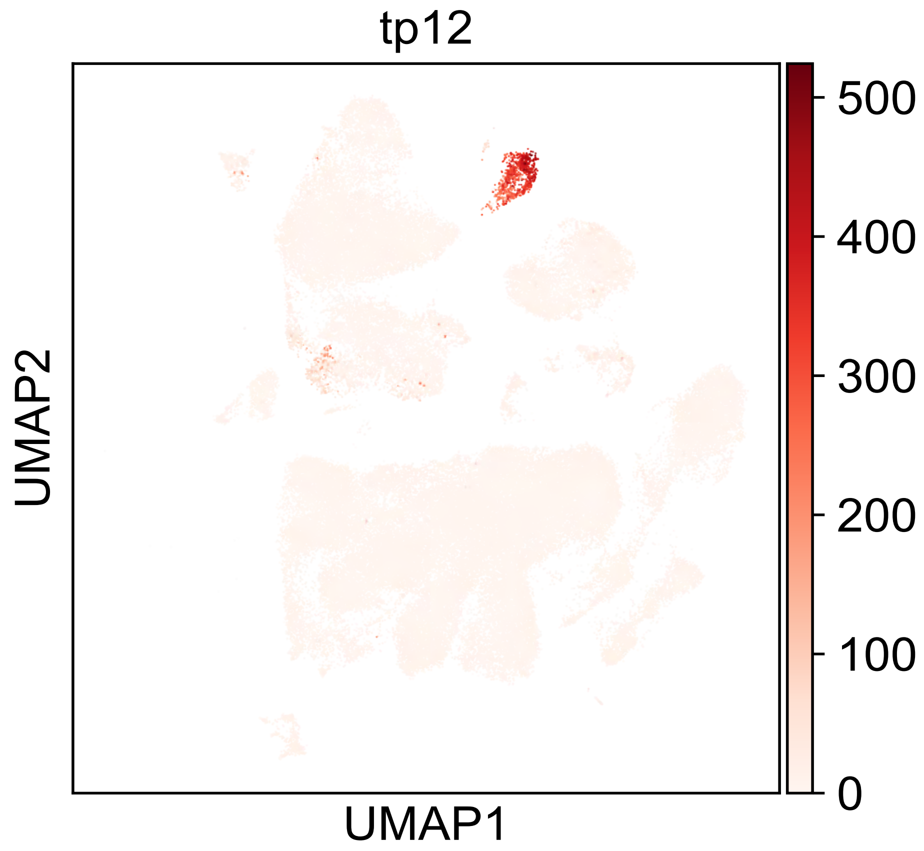

Cell Type

unknown

Top 20 genes

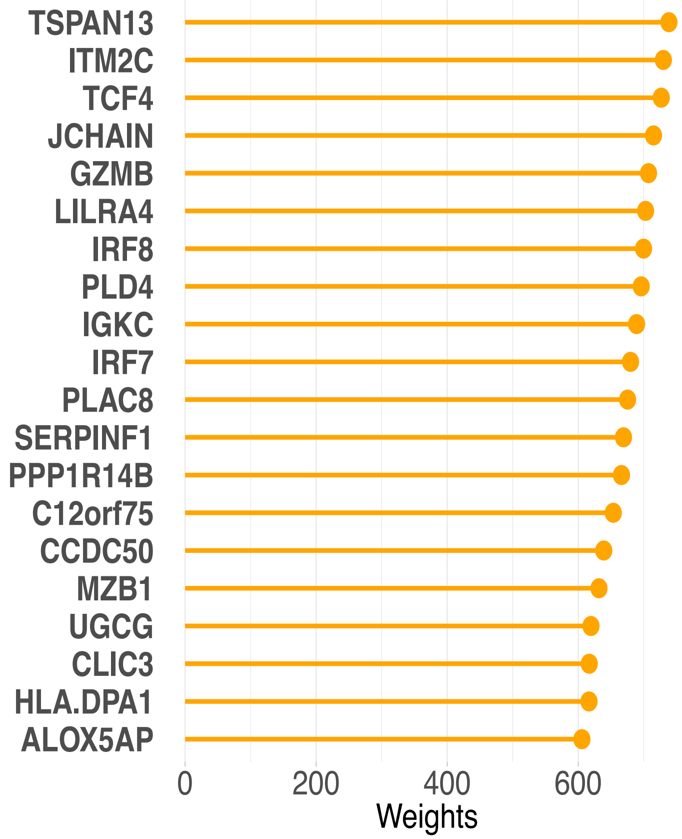

Imm GEM 14

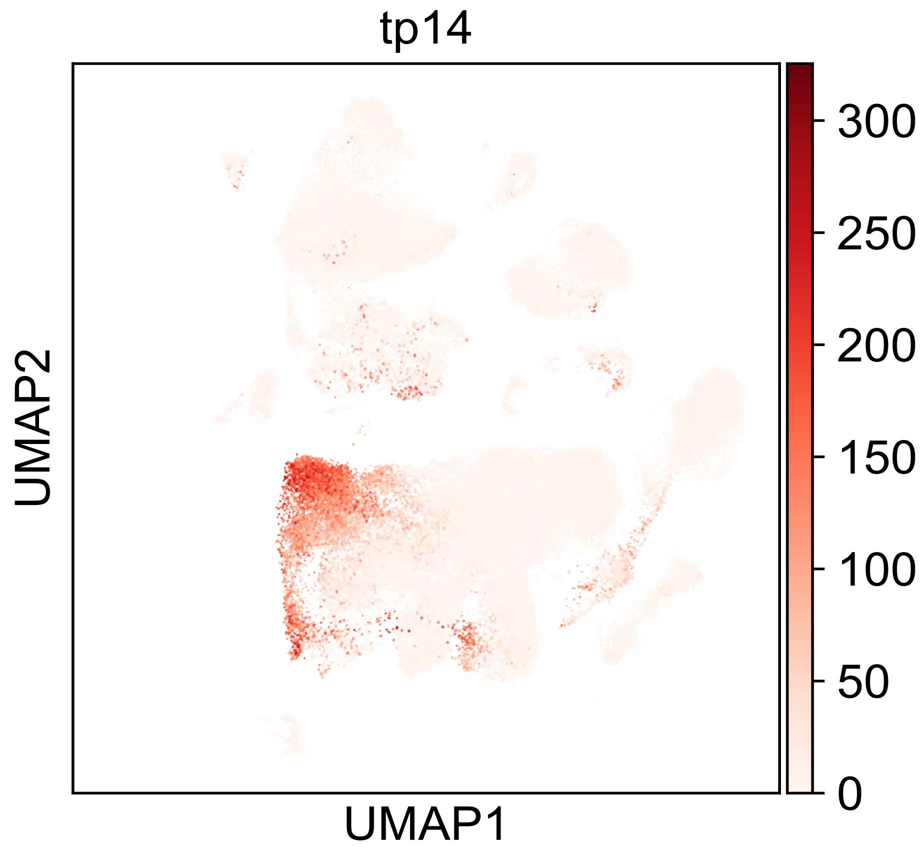

Cell Type

CD8 T cells

Tregs

NK cells

Top 20 genes

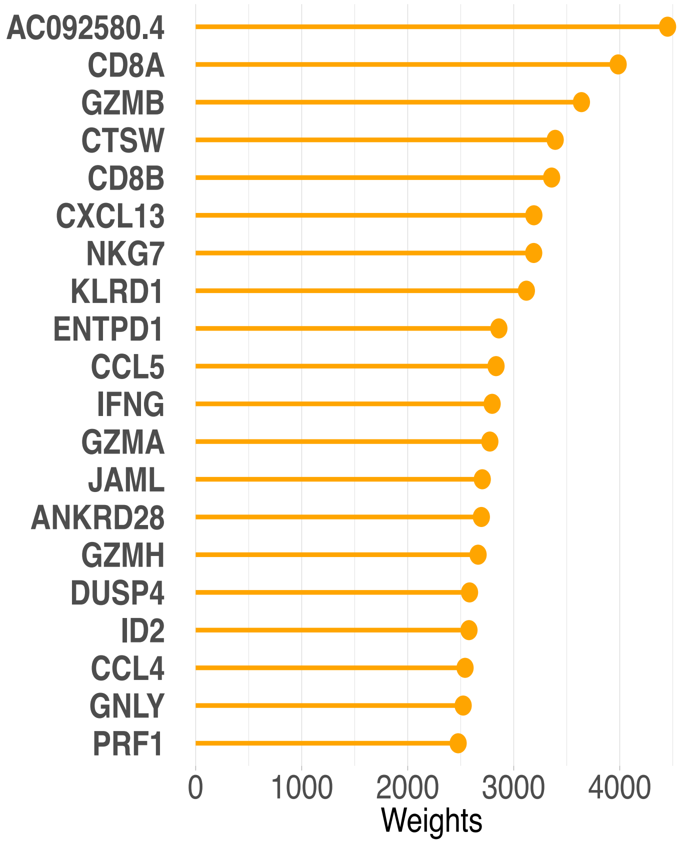

Imm GEM 15

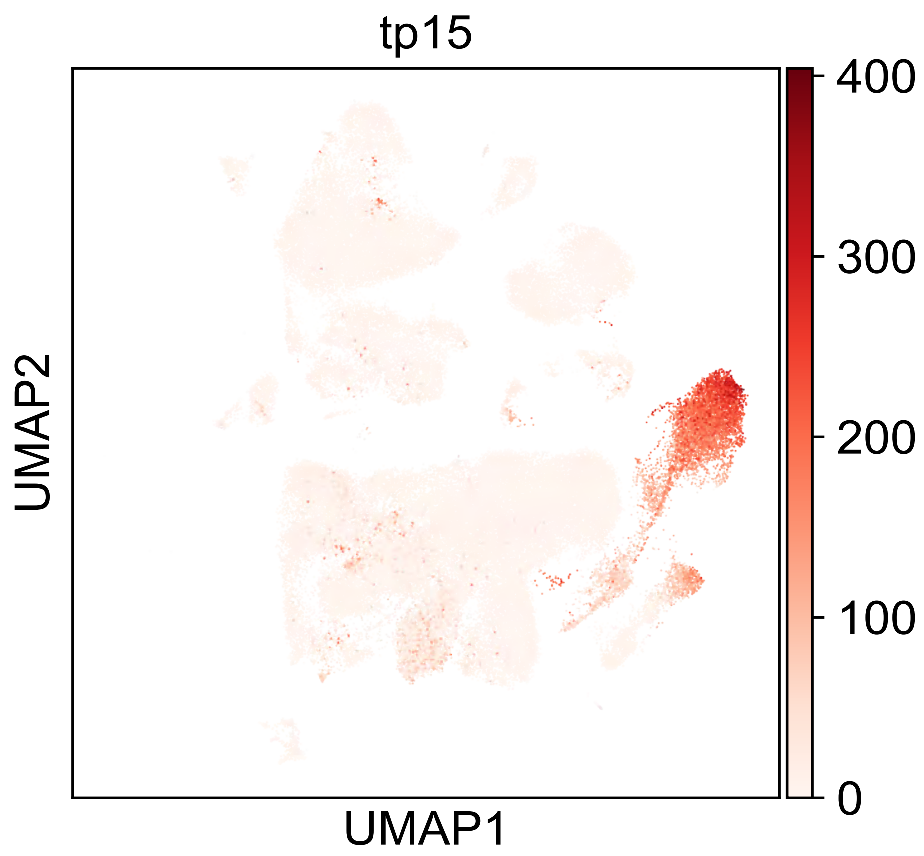

Cell Type

NK cells

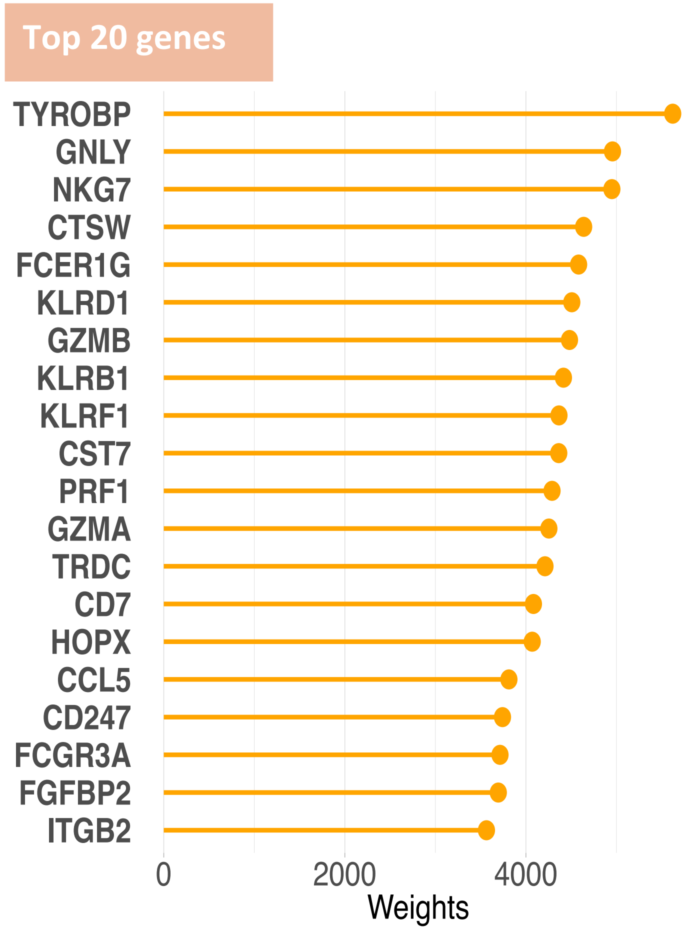

Imm GEM 16

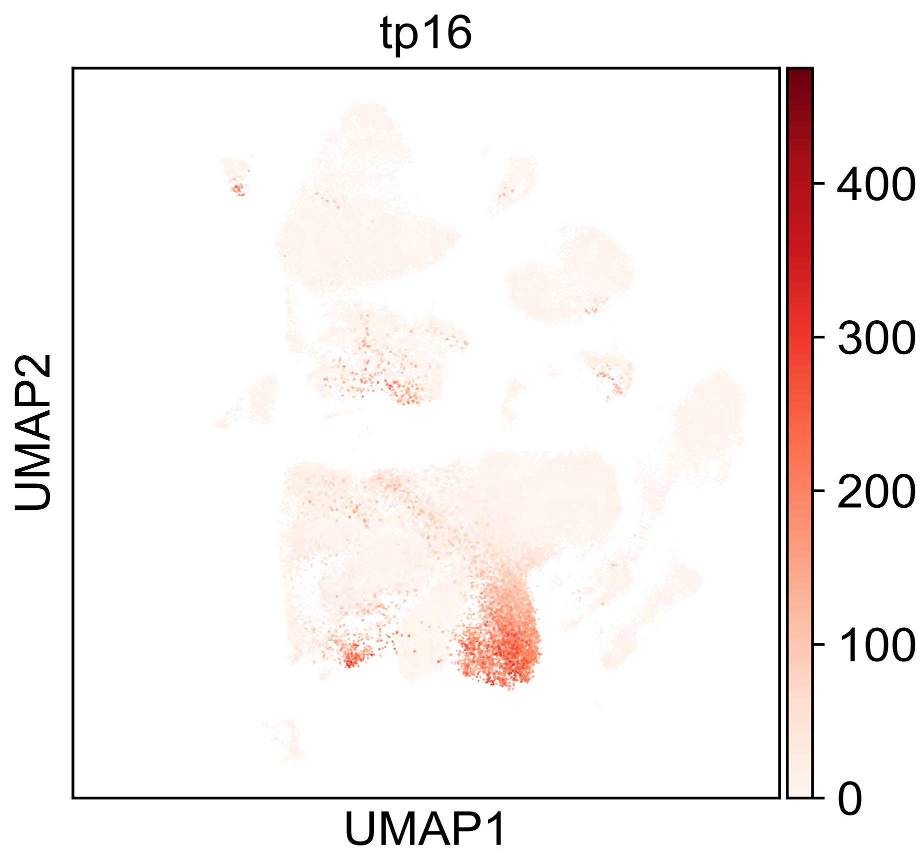

Cell Type

Tregs

CD4 T cells

Top 20 genes

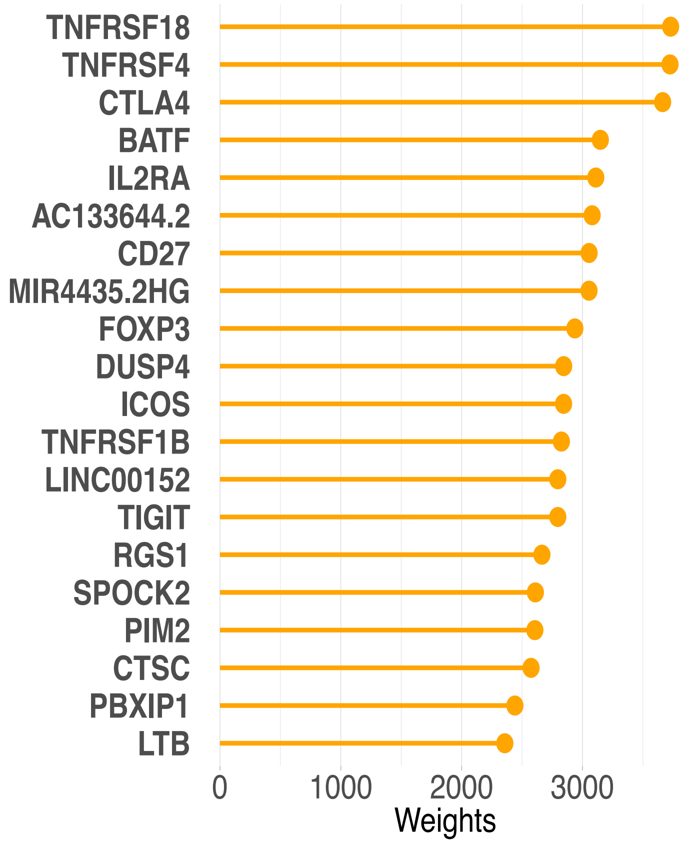

Imm GEM 17

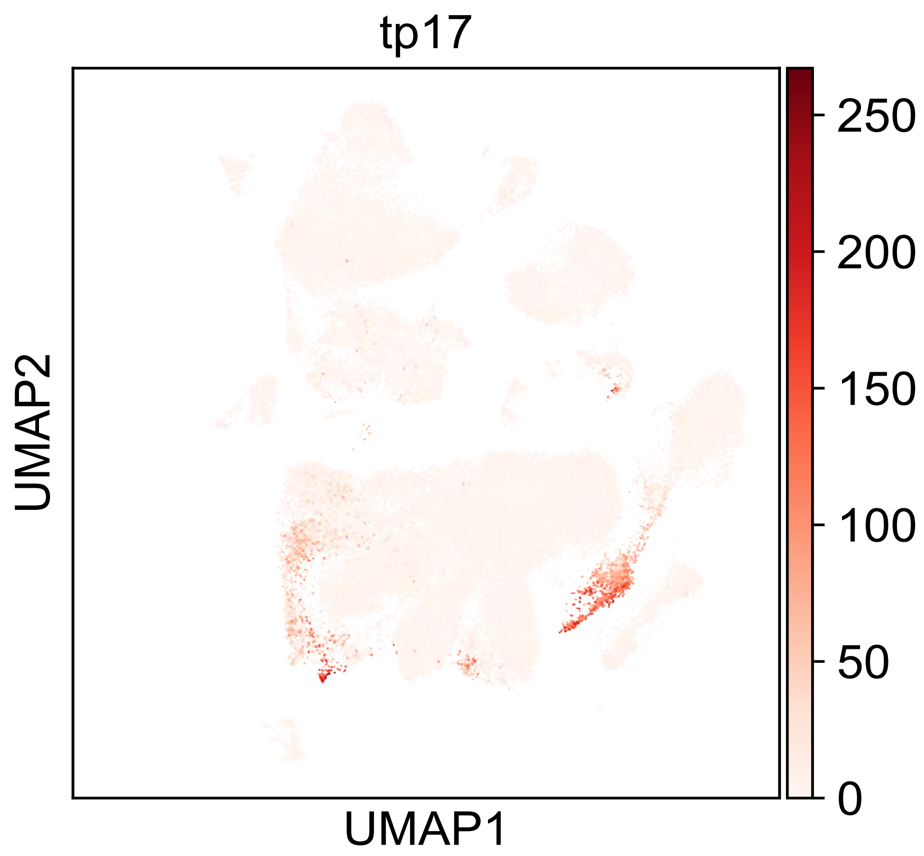

Cell Type

NK cells

CD8 T cells

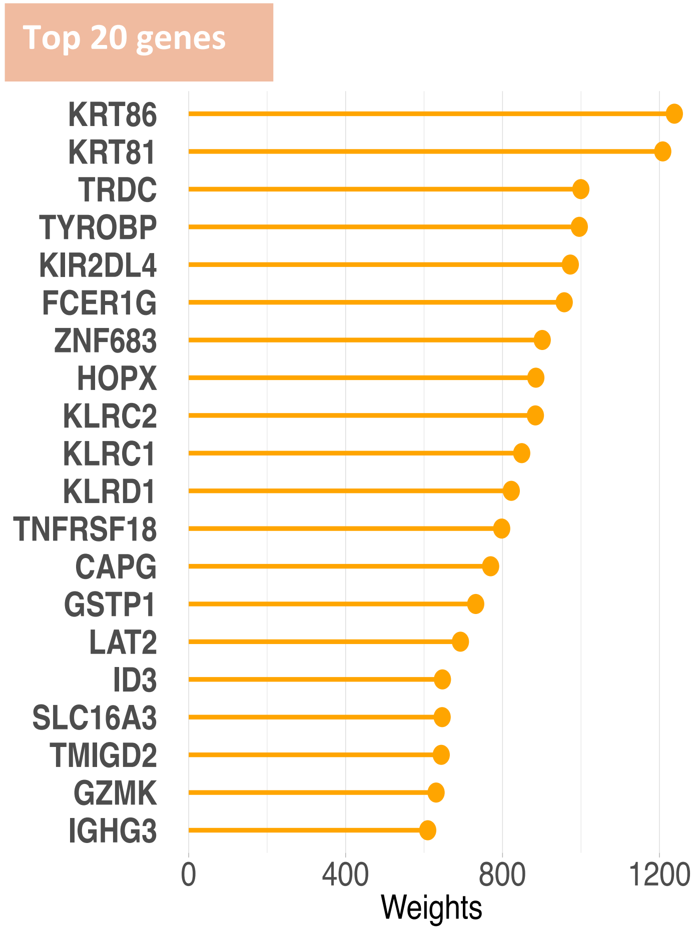

Imm GEM 18

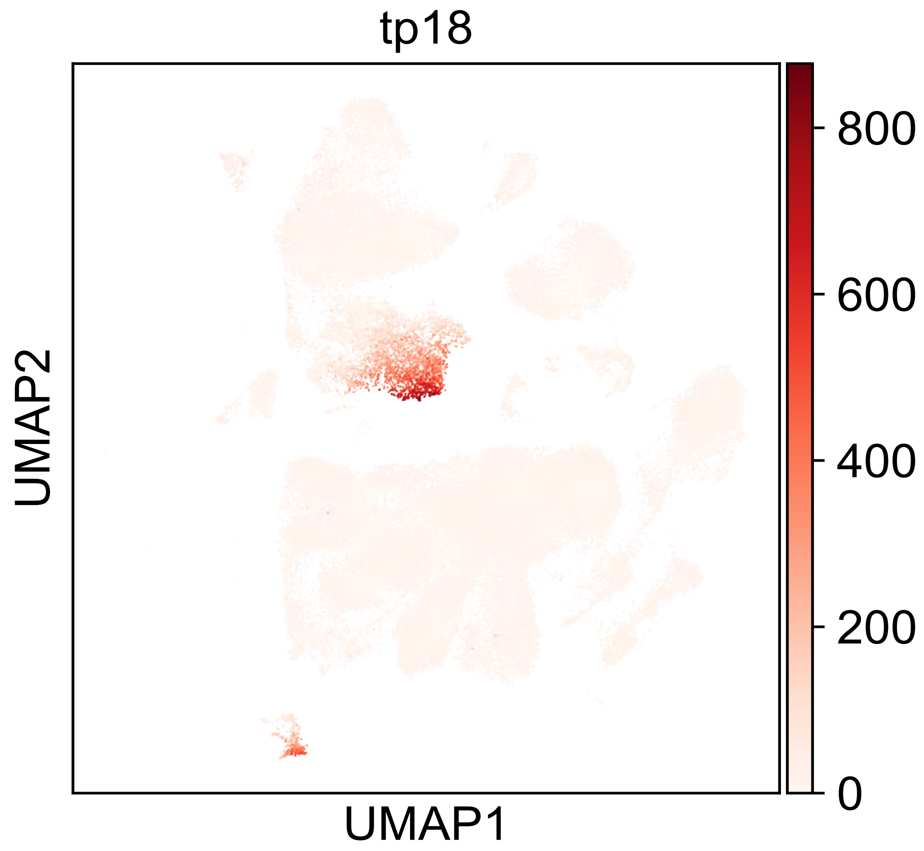

Cell Type

Macrophage

Top 20 genes

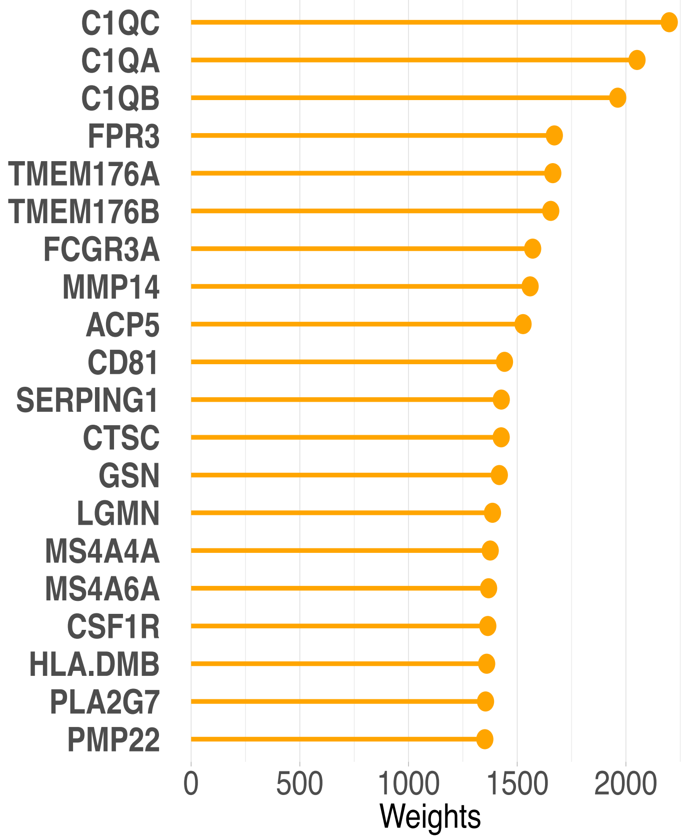

Imm GEM 19

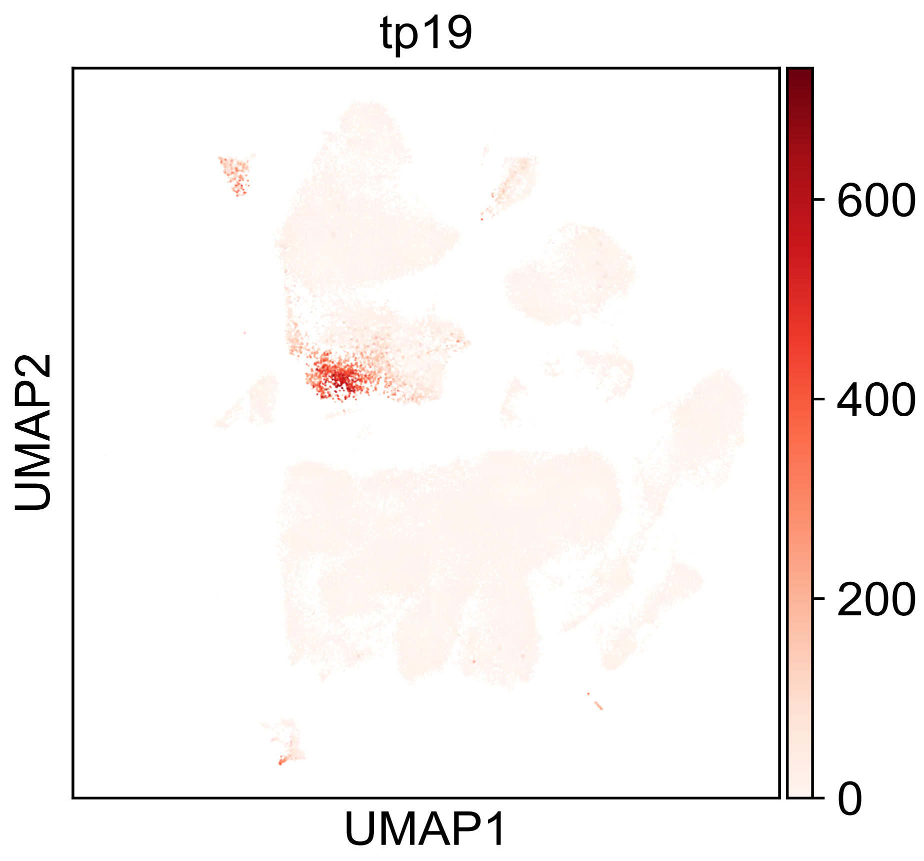

Cell Type

Macrophage

Dendritic

Top 20 genes

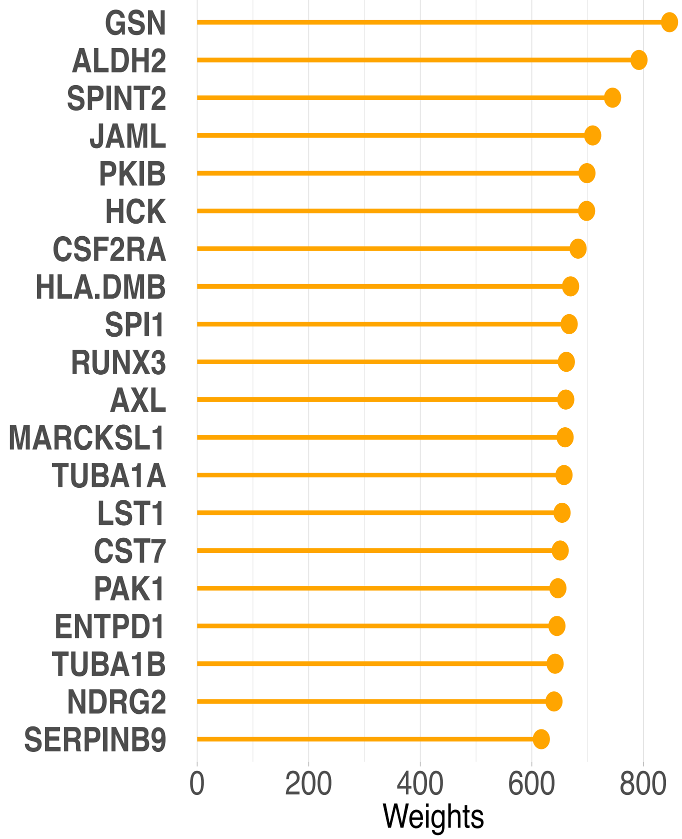

Imm GEM 20

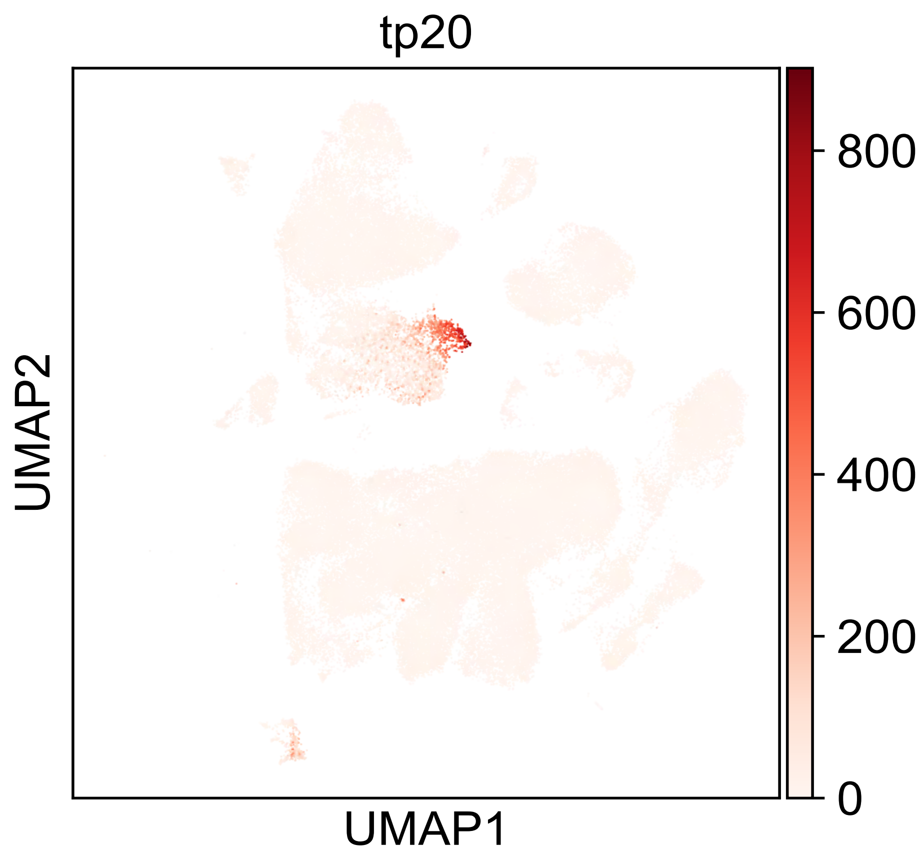

Cell Type

Macrophage

Top 20 genes

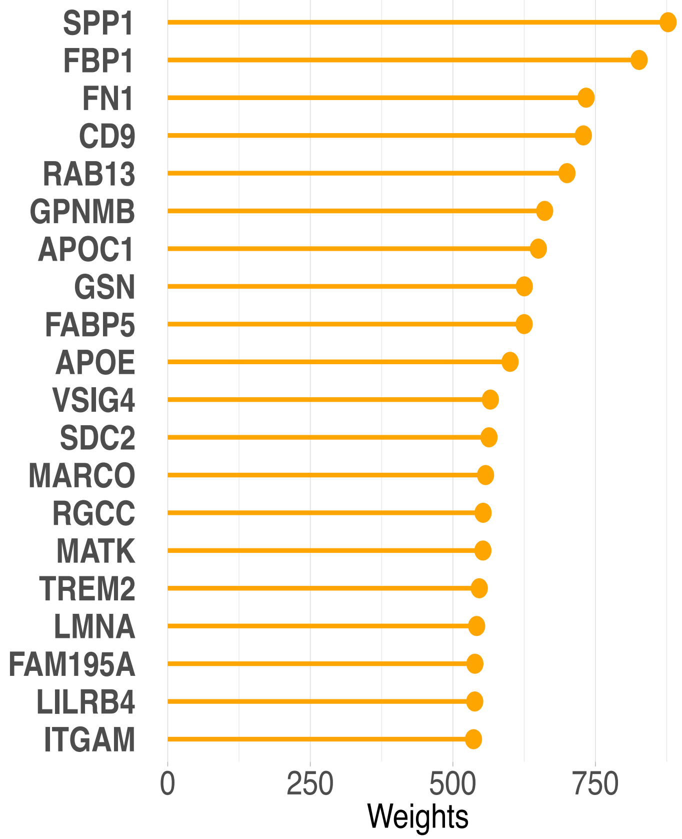

Imm GEM 22

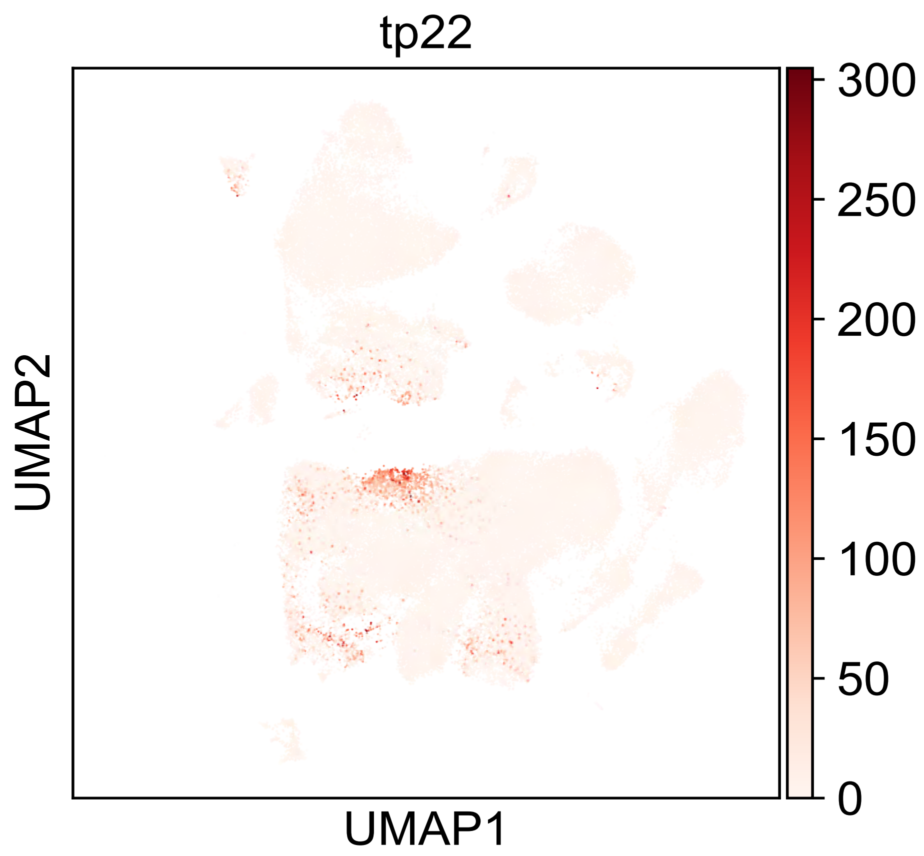

Cell Type

CD4 T cells

CD8 T cells

Top 20 genes

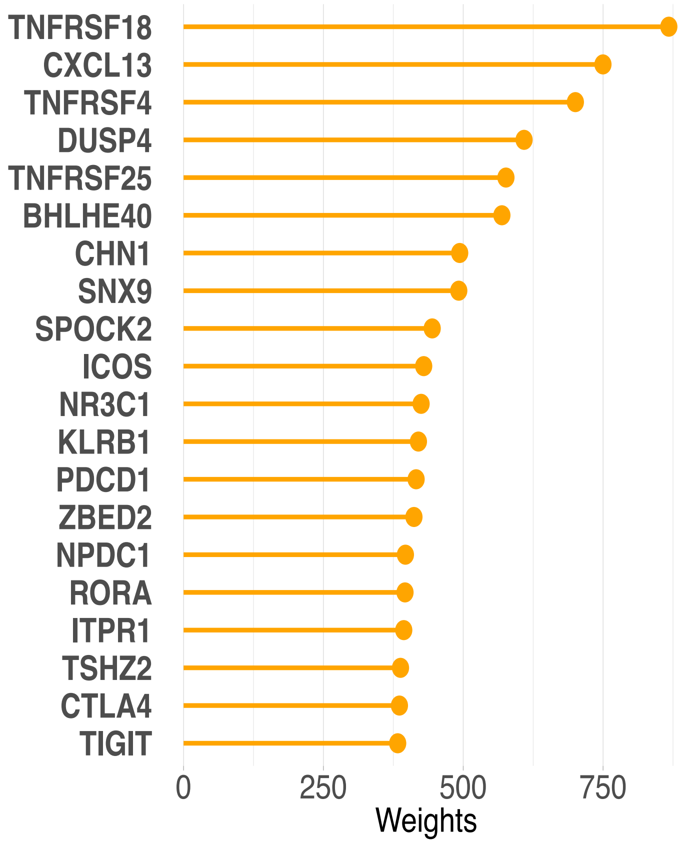

Imm GEM 23

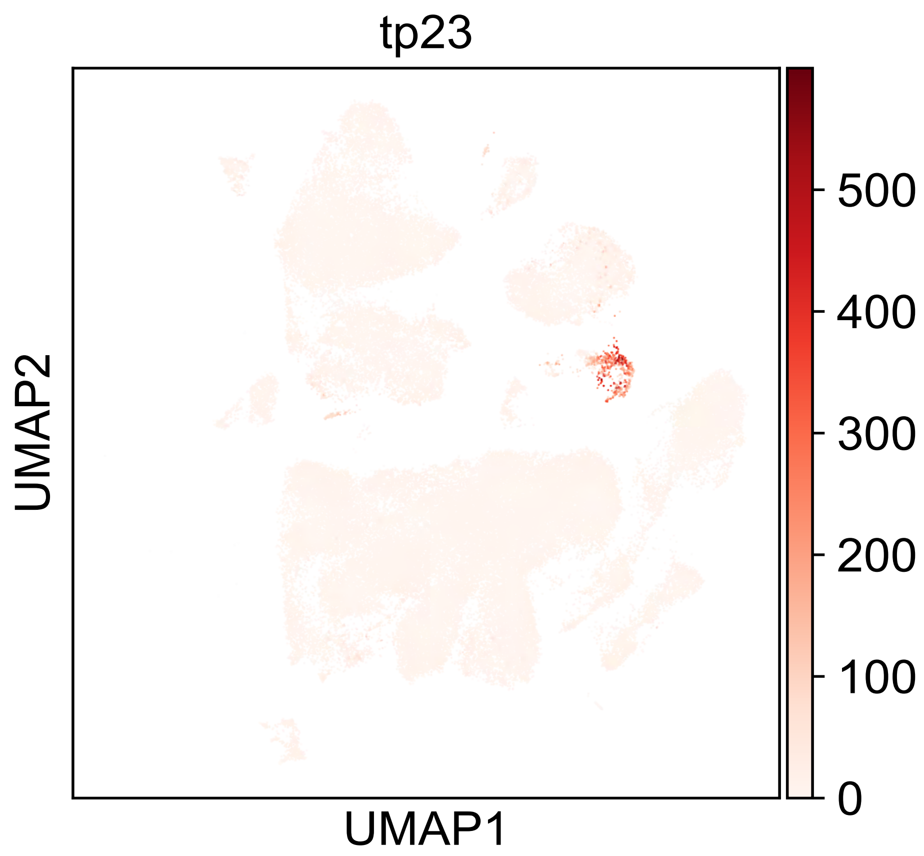

Cell Type

B cells

Top 20 genes

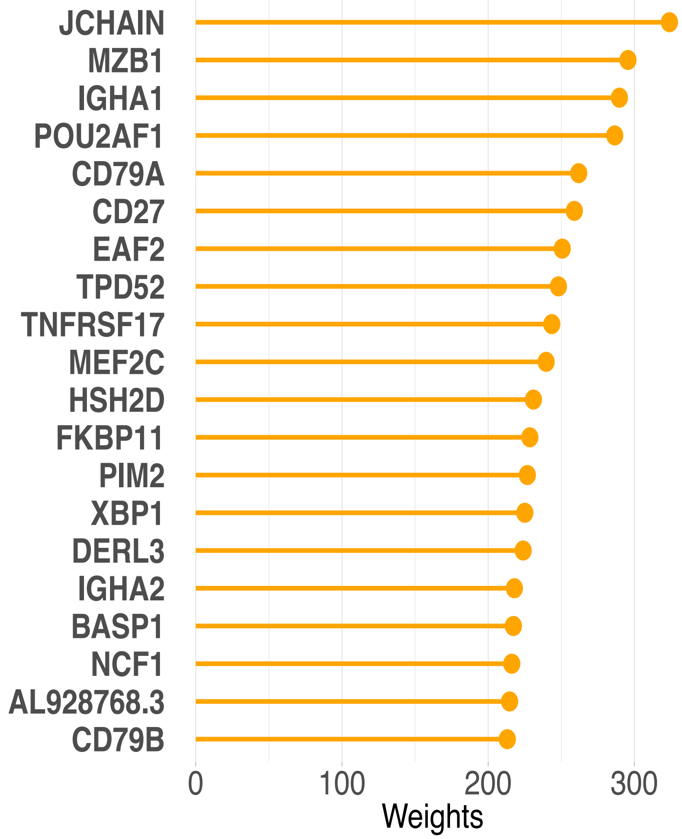

Imm GEM 26

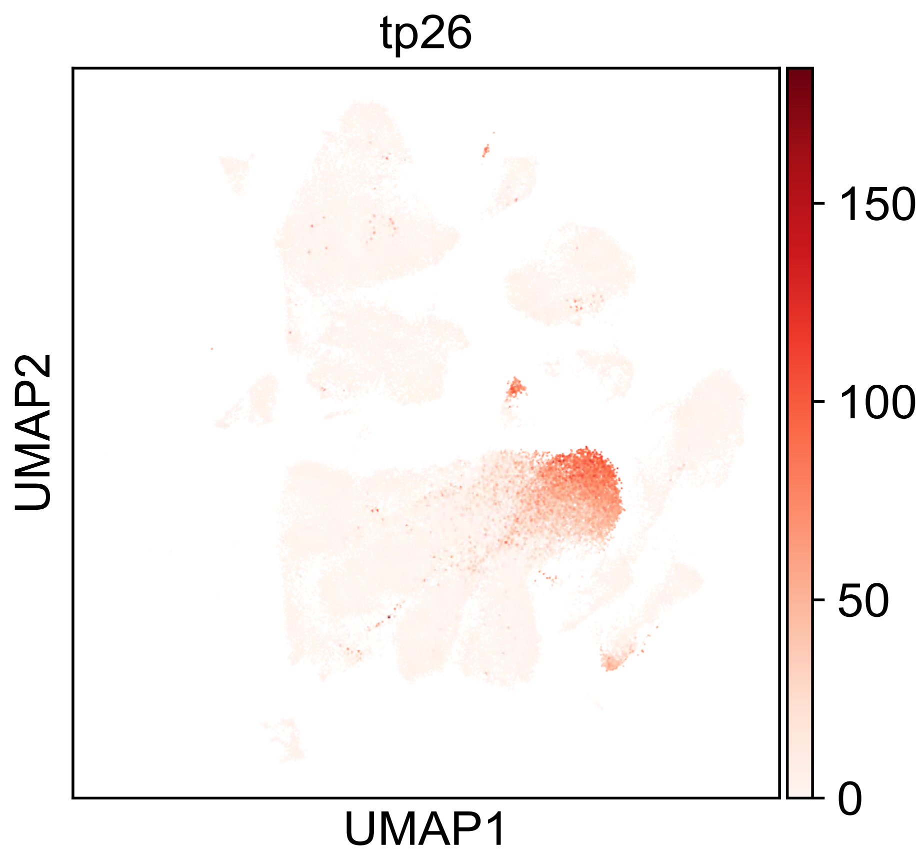

Cell Type

CD4 T cells

NK cells

Top 20 genes

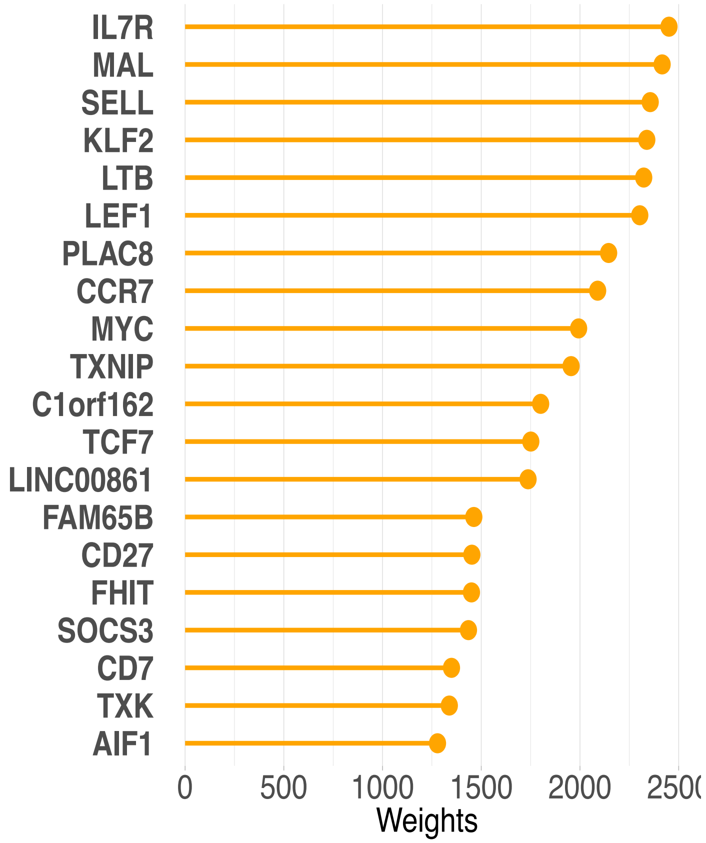

Imm GEM 27

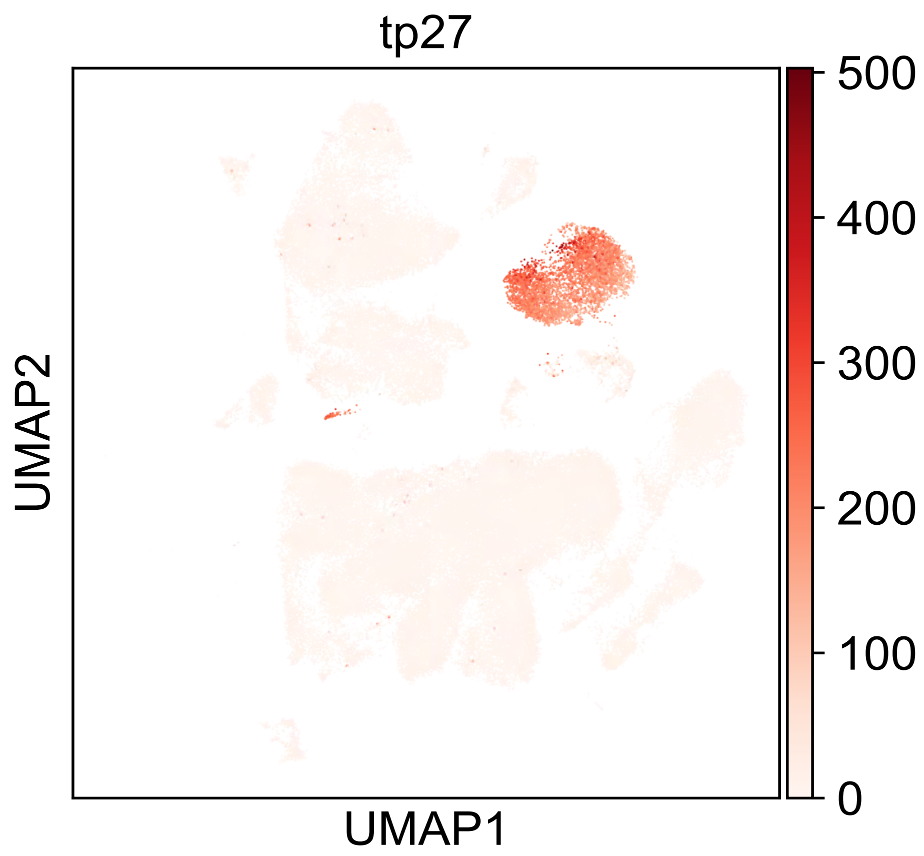

Cell Type

B cells

Top 20 genes

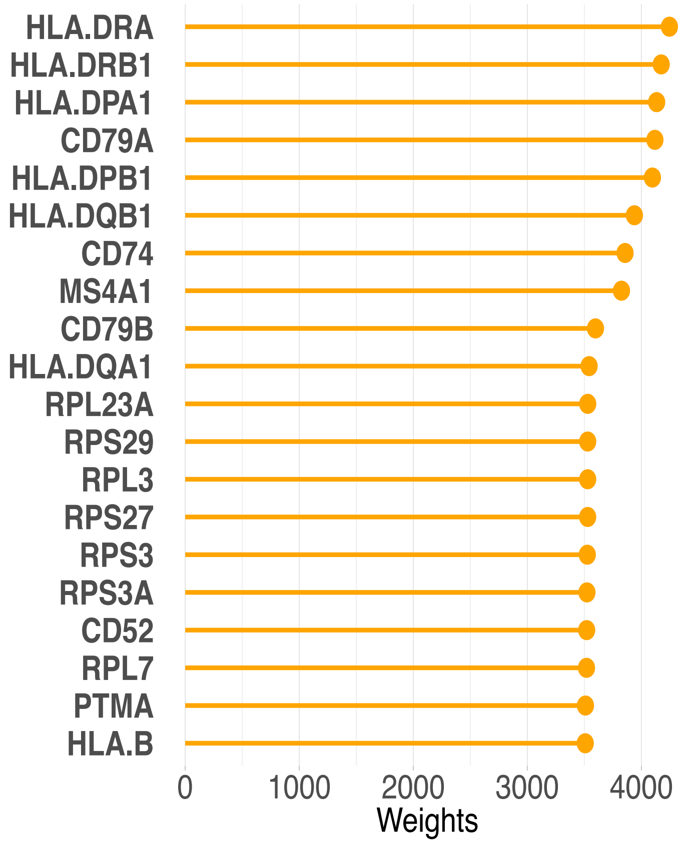

Imm GEM 28

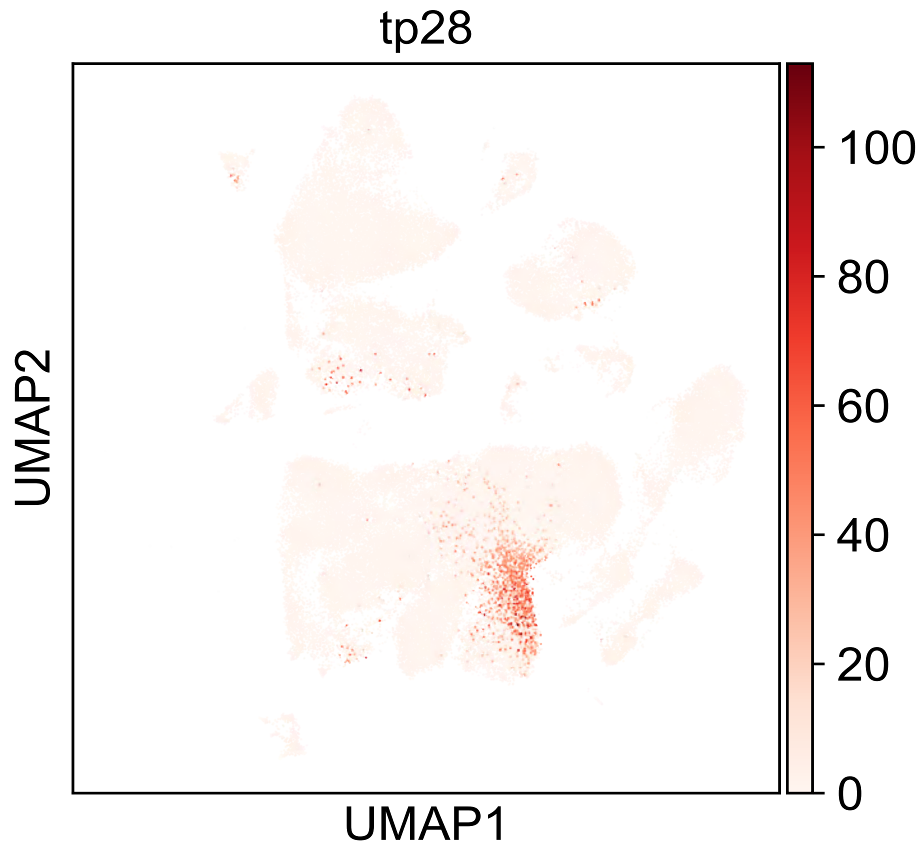

Cell Type

CD4 T cells

Tregs

Top 20 genes

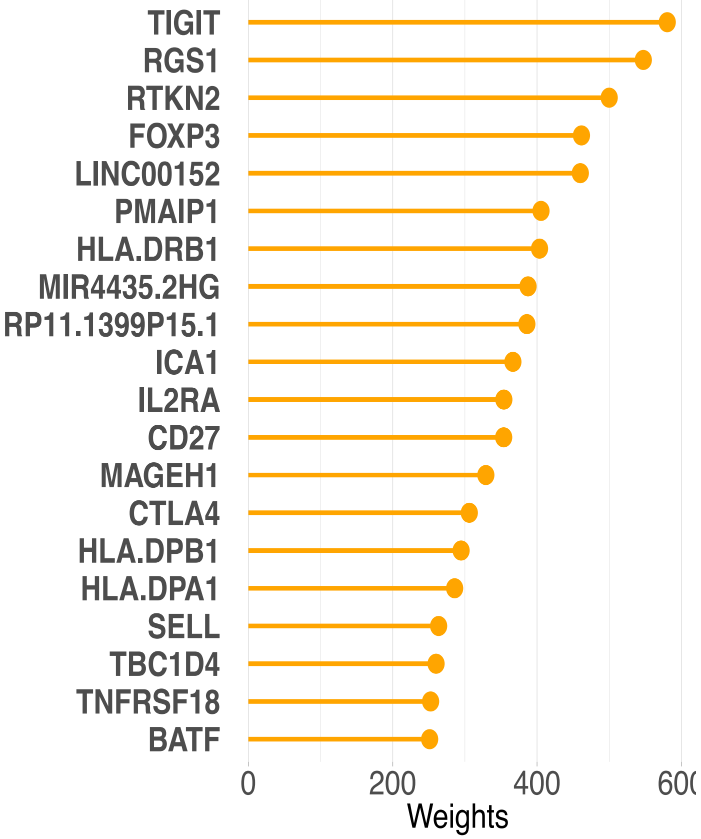

Imm GEM 29

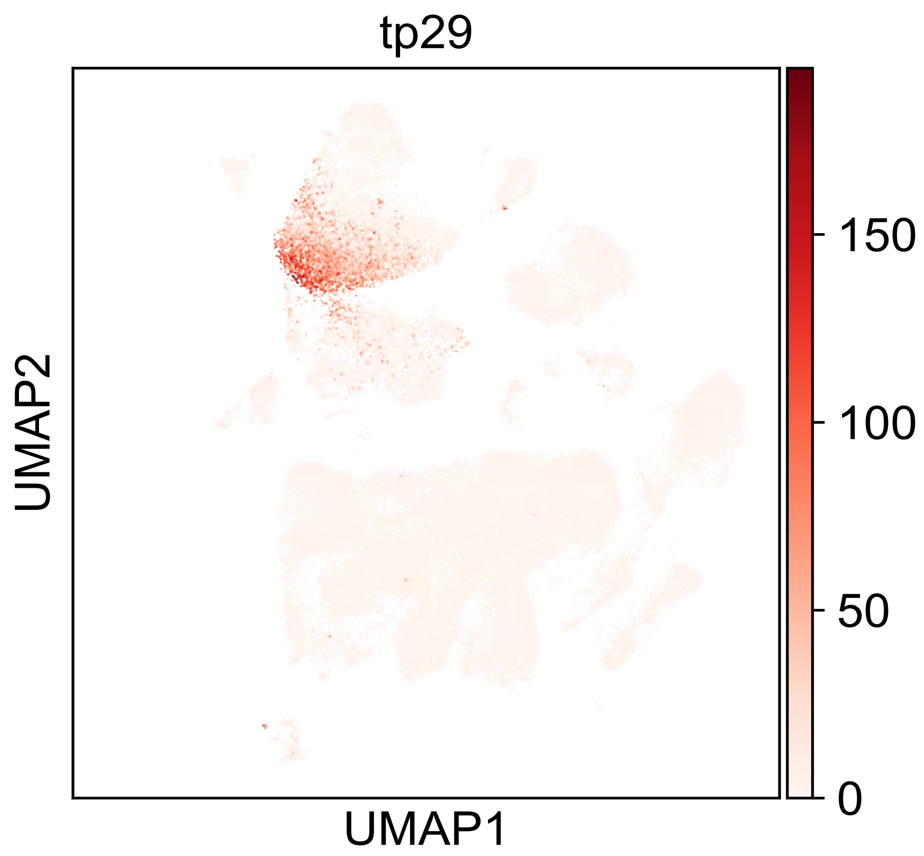

Cell Type

Monocytes

Top 20 genes

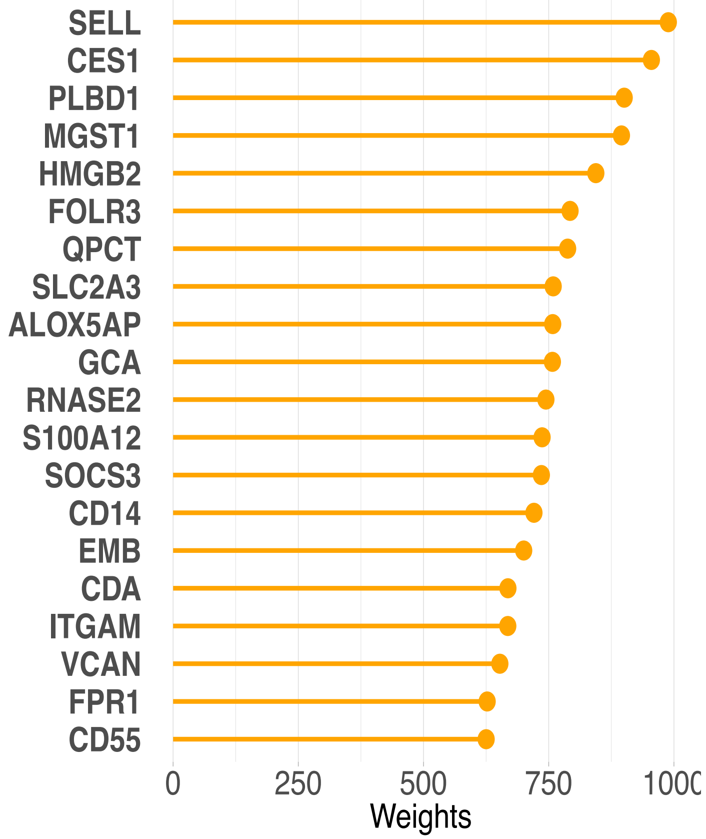

Imm GEM 32

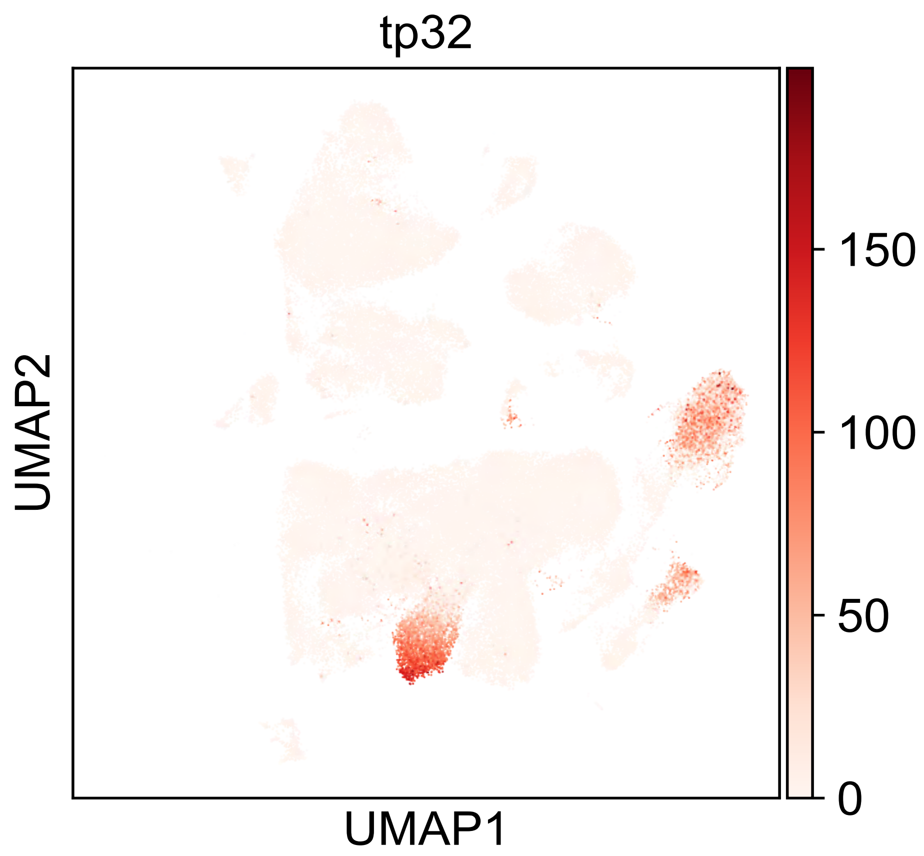

Cell Type

CD8 T cells

NK cells

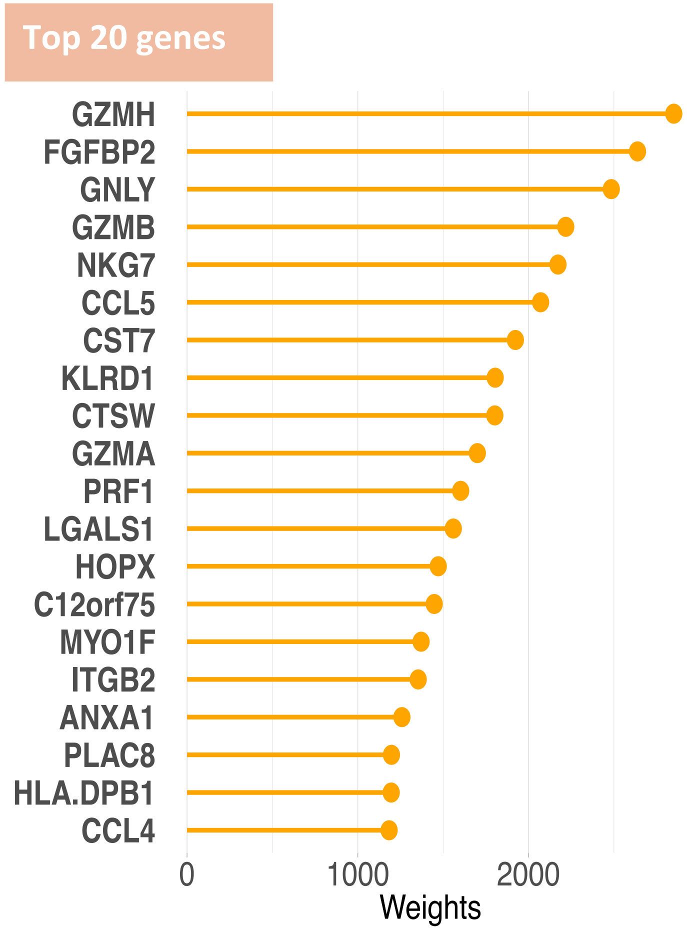

Imm GEM 33

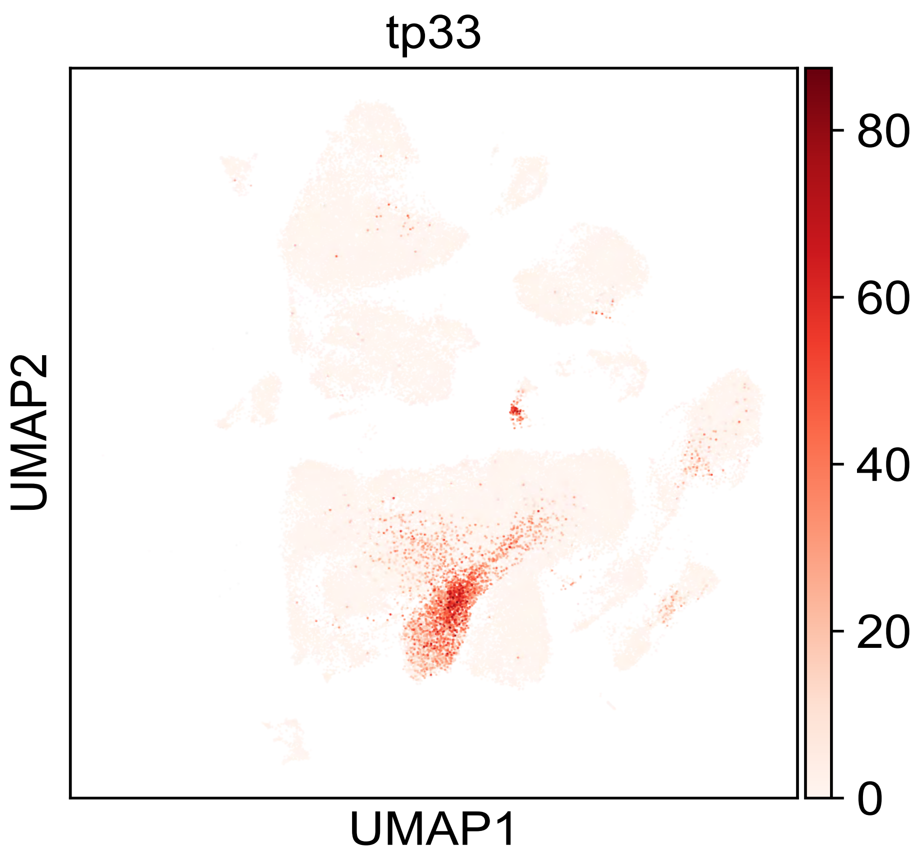

Cell Type

CD8 T cells

Top 20 genes

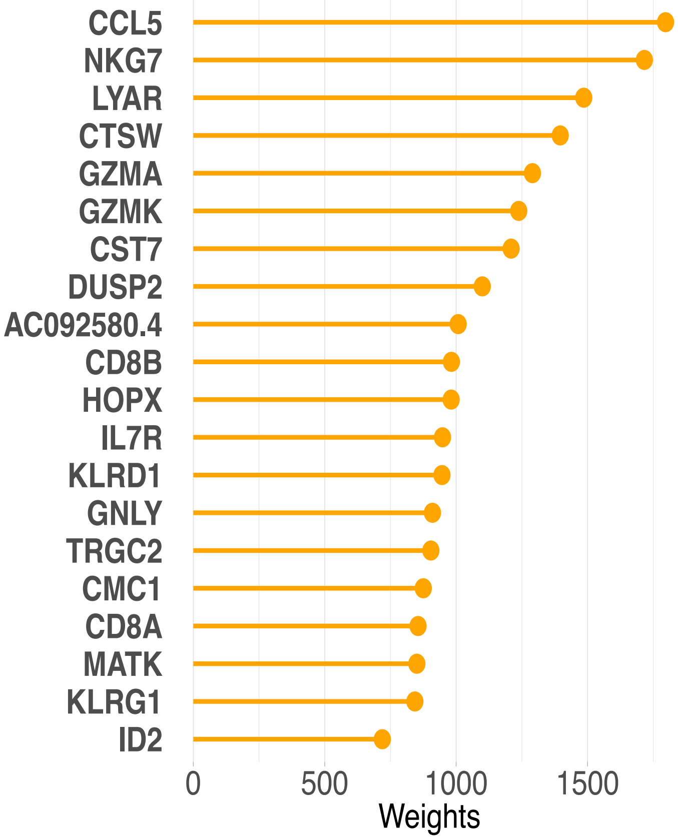

Imm GEM 34

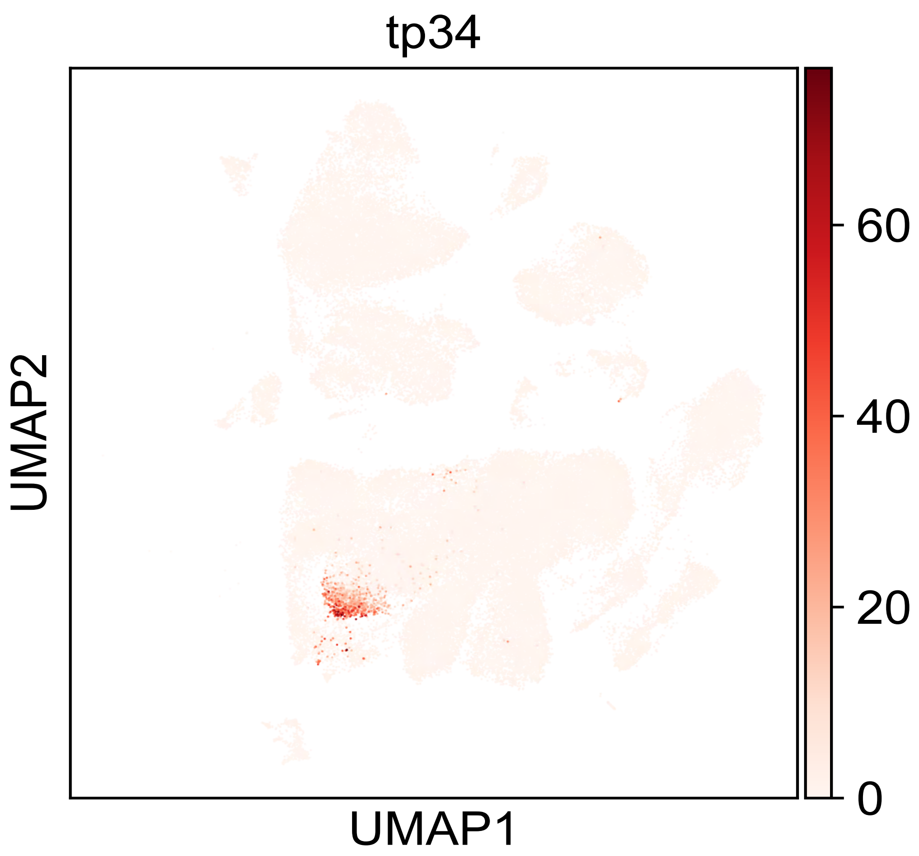

Cell Type

CD8 T cells

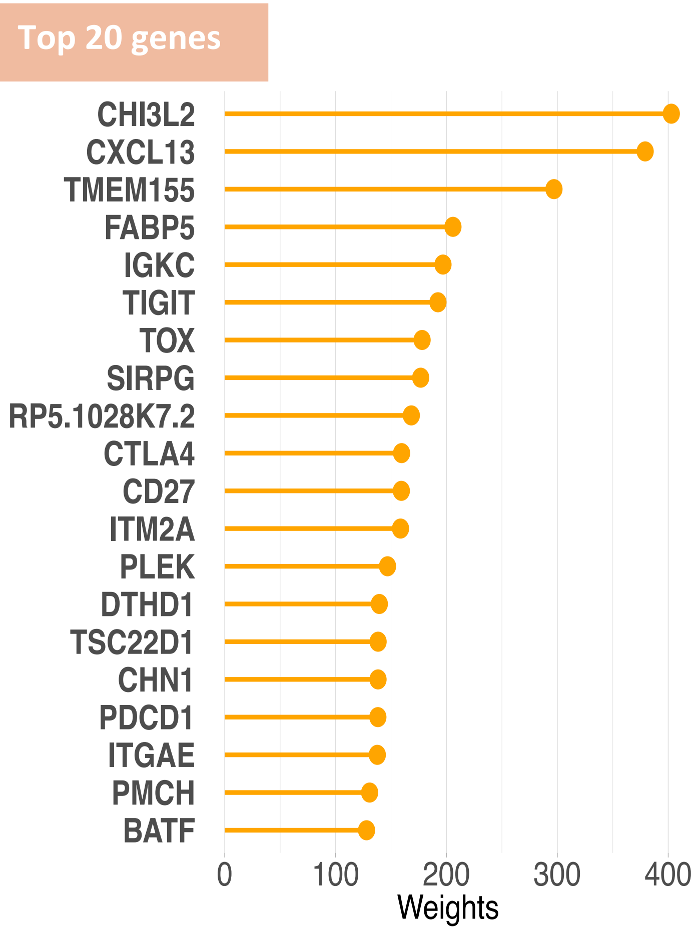

Imm GEM 35

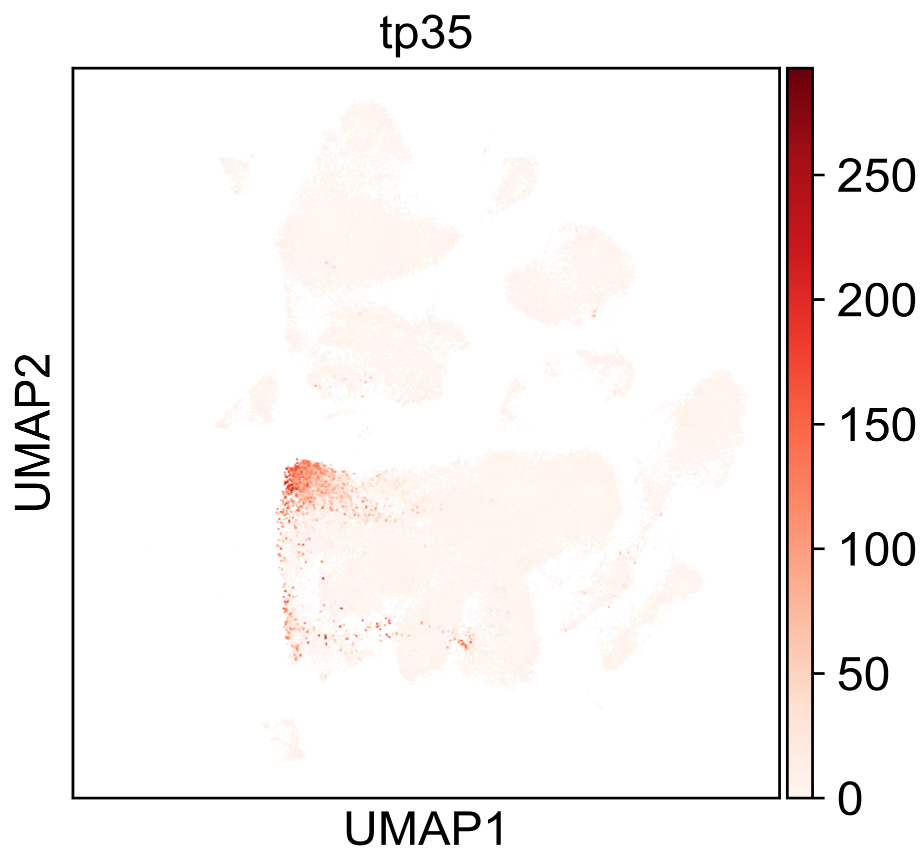

Cell Type

CD8 T cells

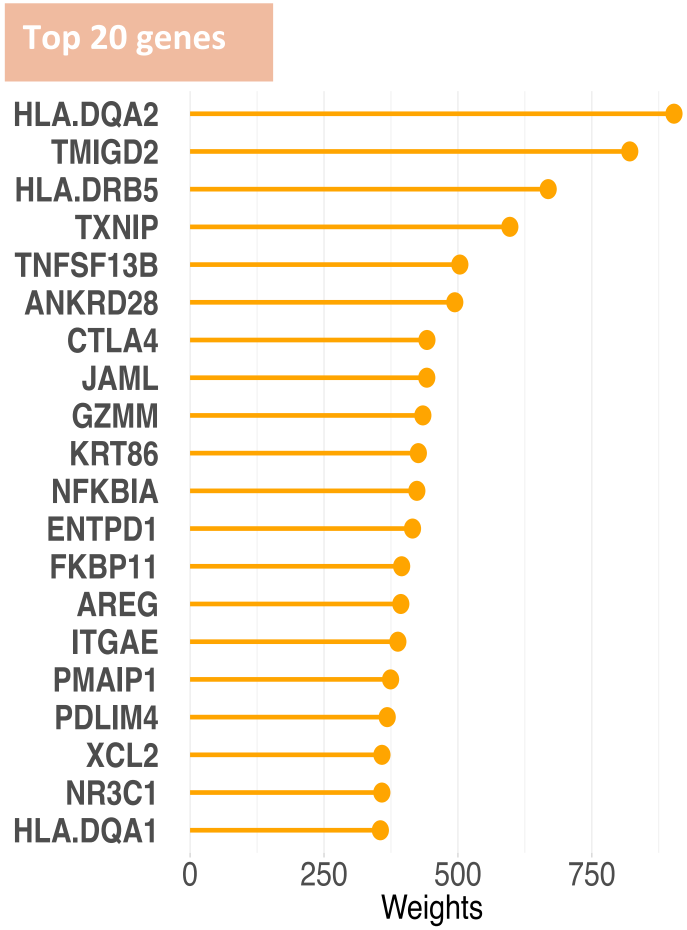

Imm GEM 38

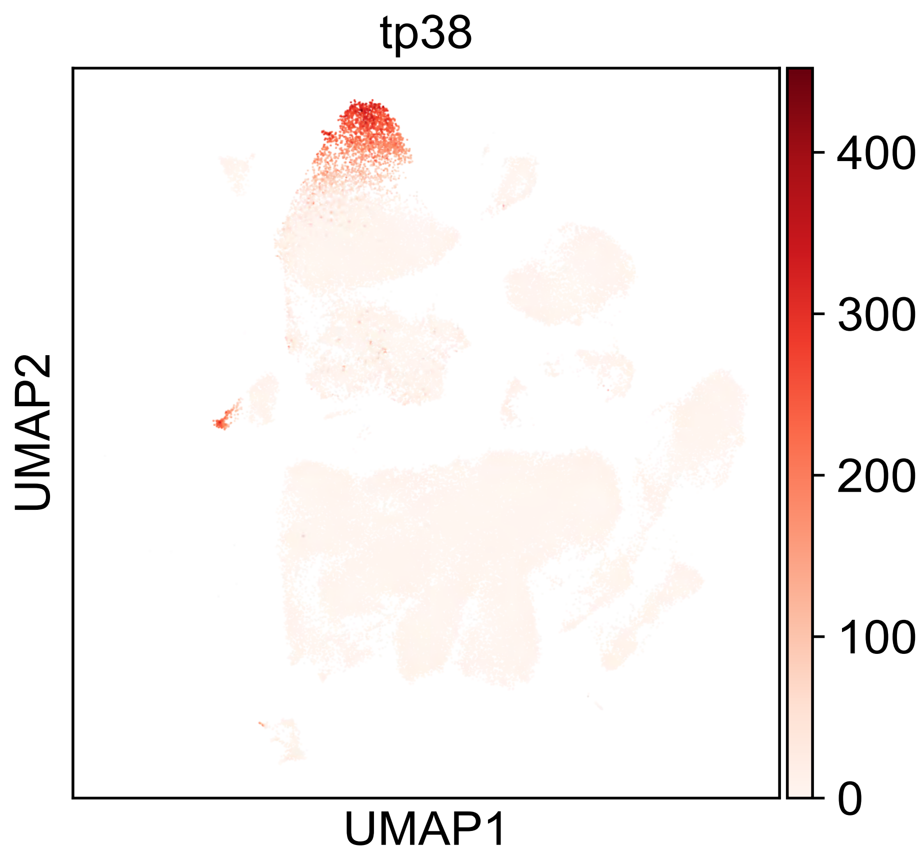

Cell Type

Monocytes

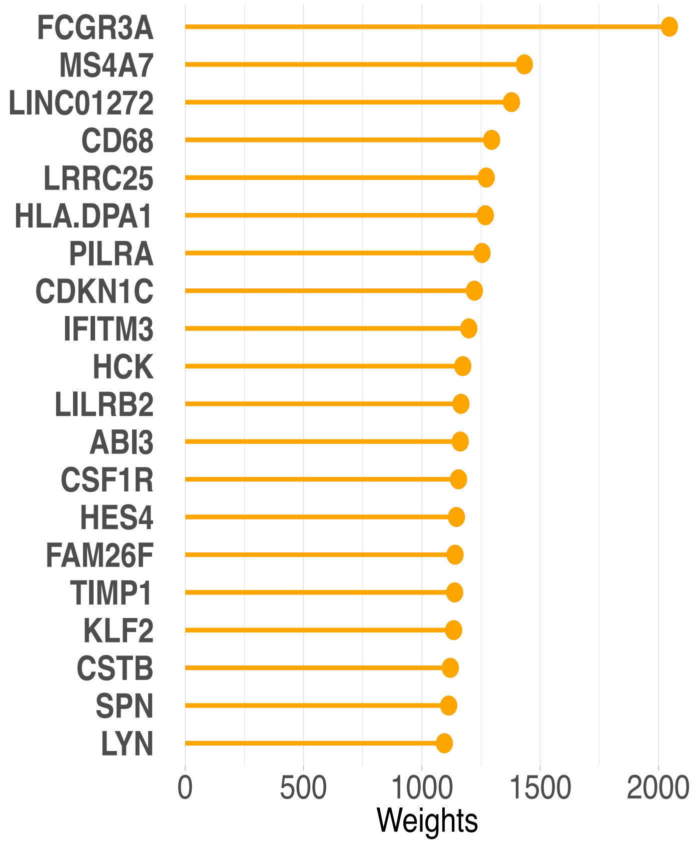

Imm GEM 40

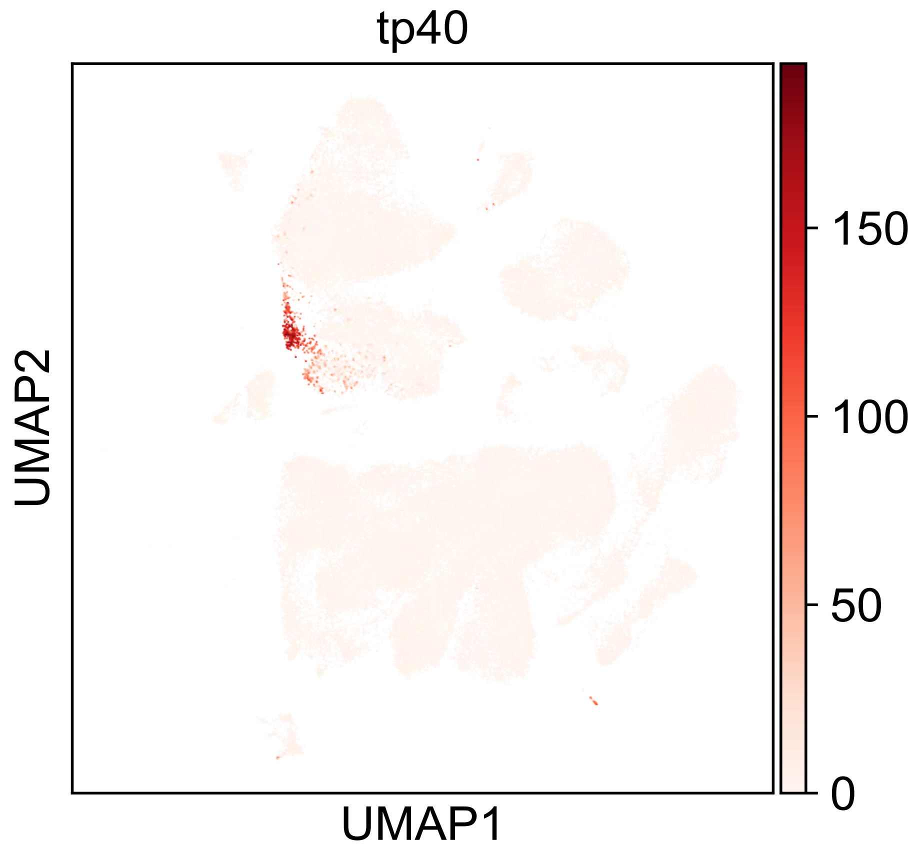

Cell Type

Macrophage

Top 20 genes

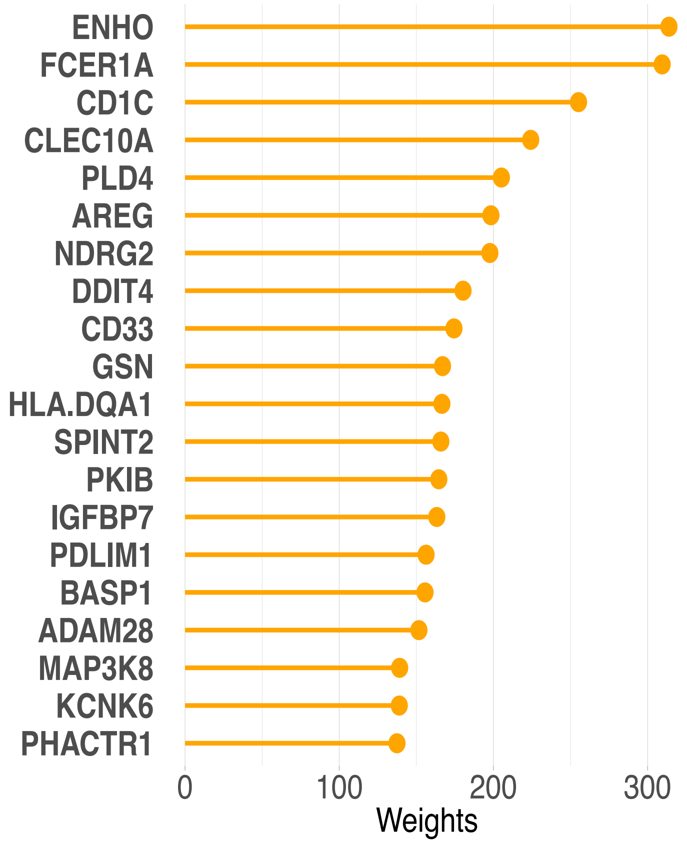

Imm GEM 41

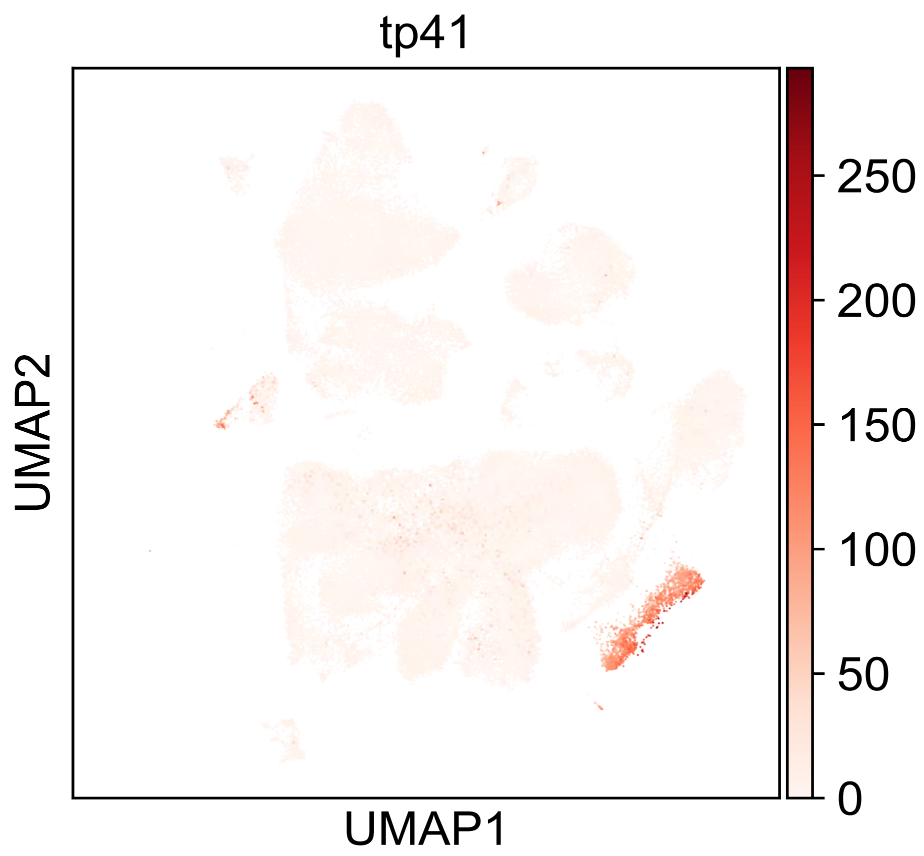

Cell Type

NK cells

Top 20 genes

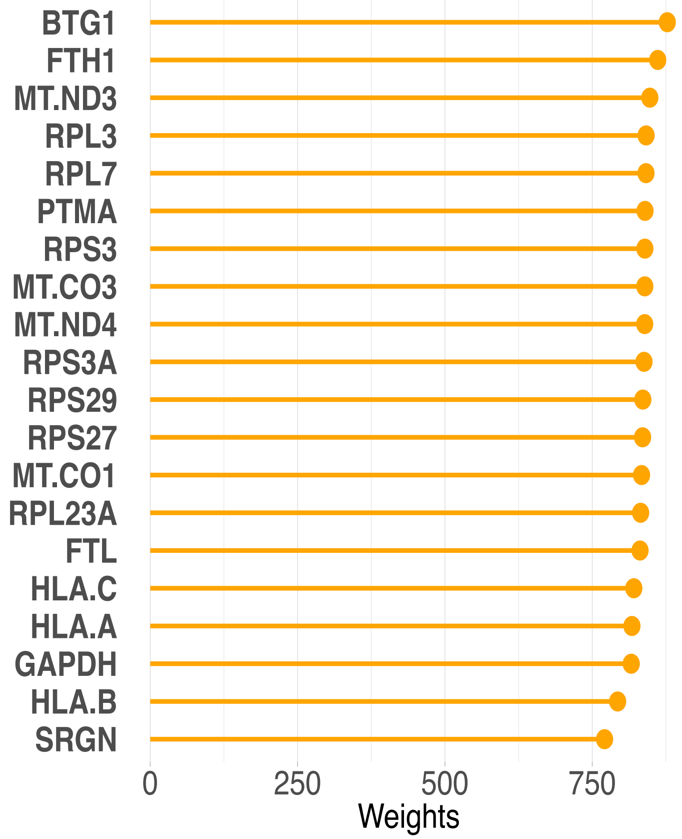

Imm GEM 44

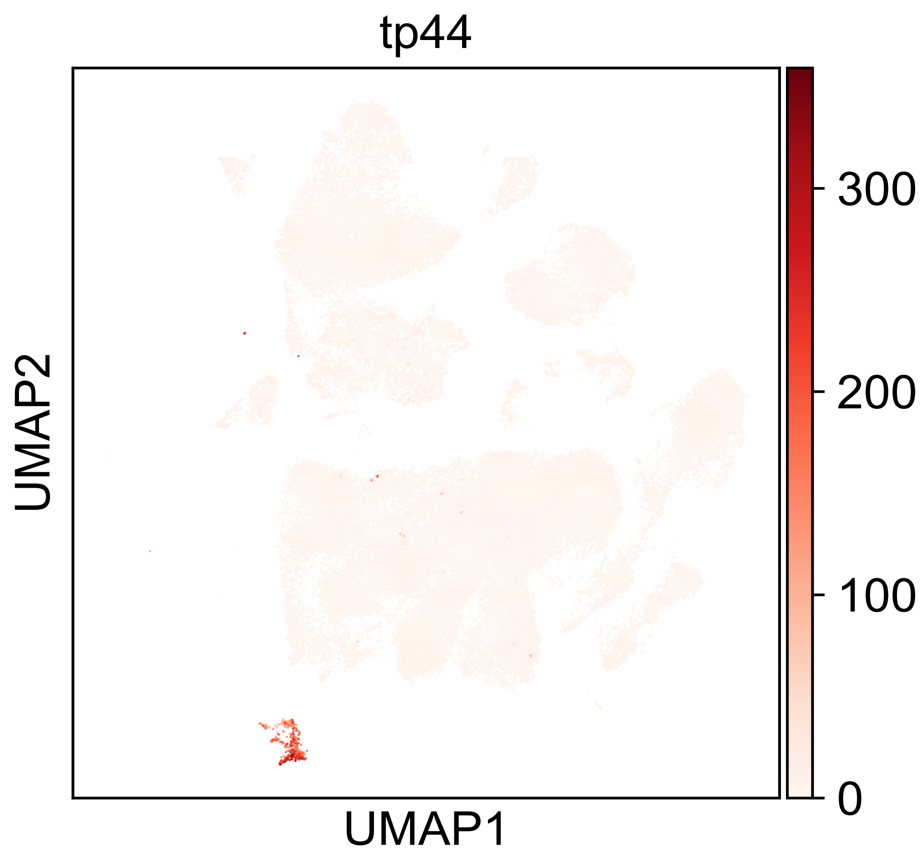

Cell Type

unknown

Top 20 genes

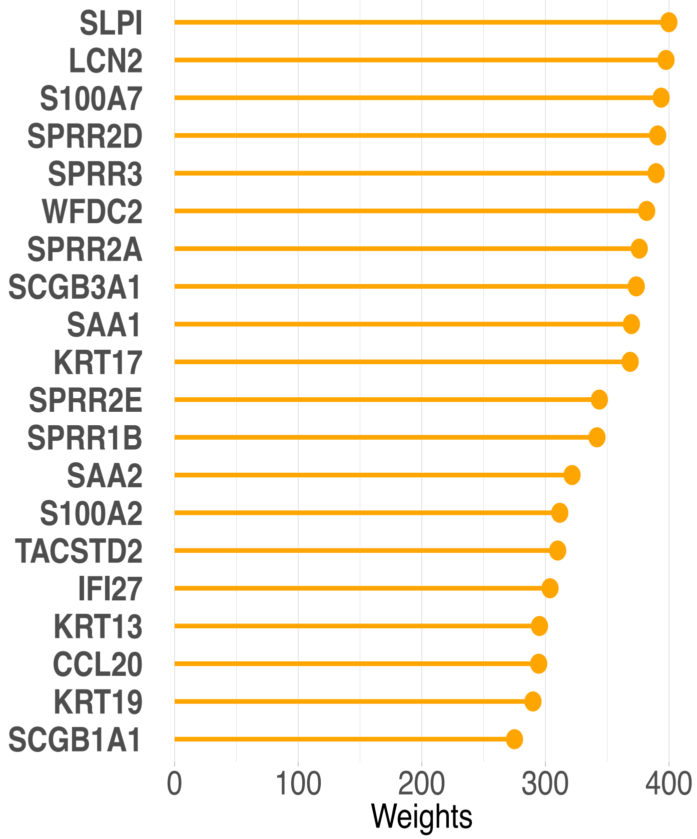

Imm GEM 49

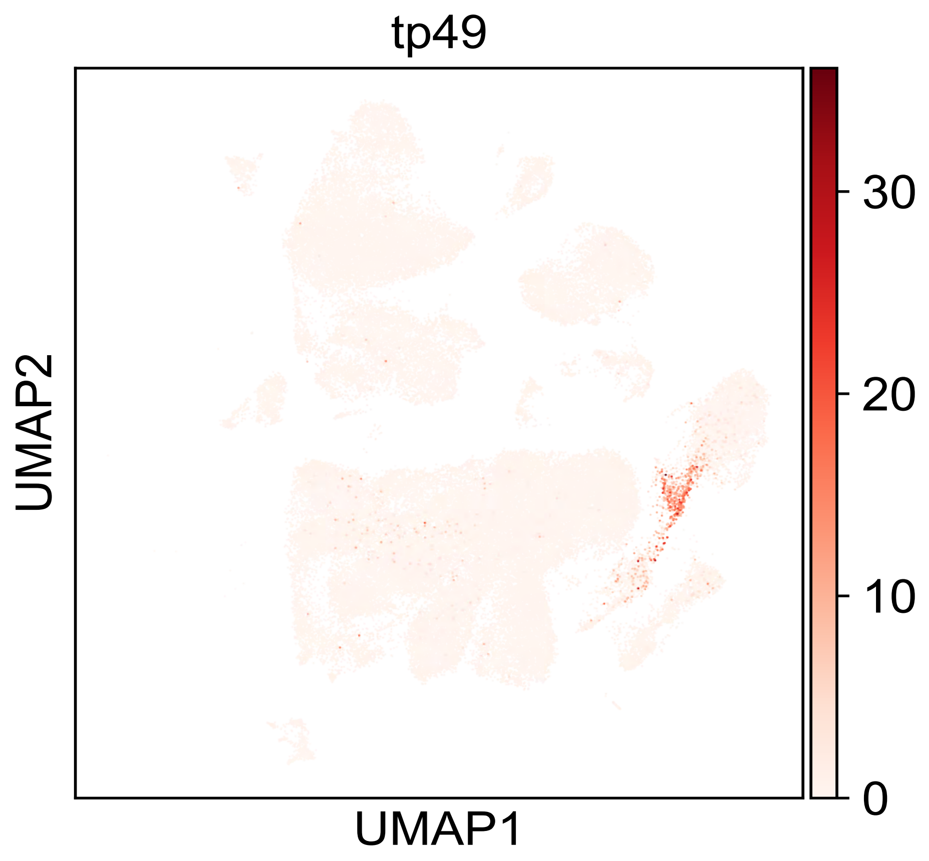

Cell Type

NK cells

Top 20 genes

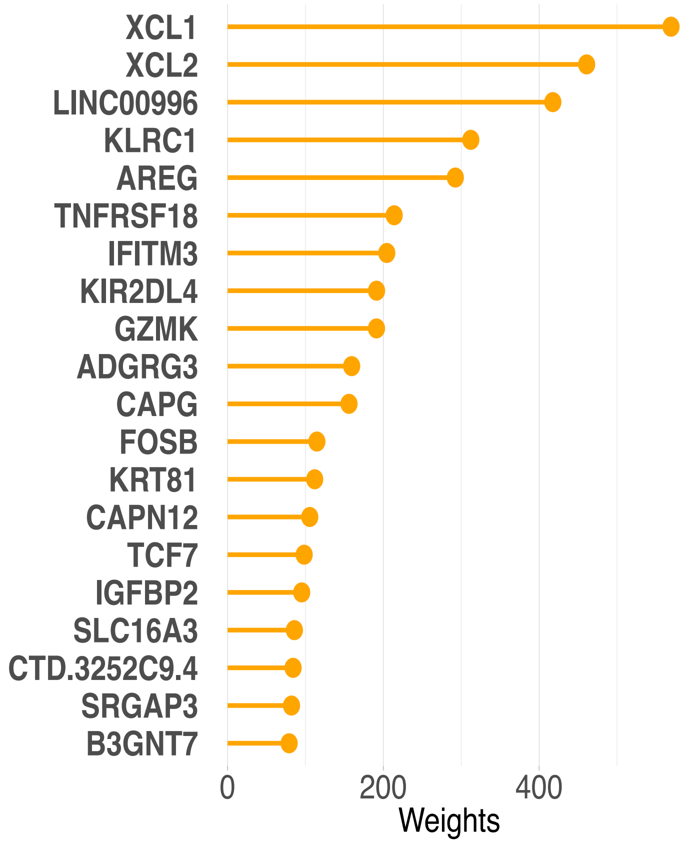

Imm GEM 51

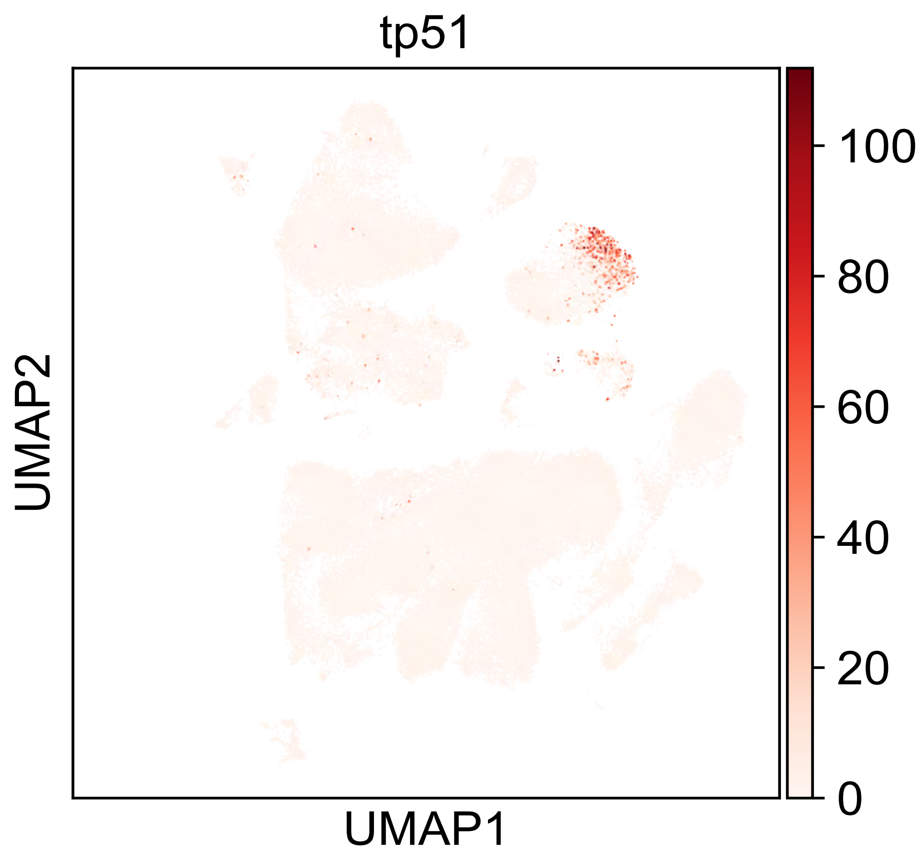

Cell Type

B cells

Top 20 genes

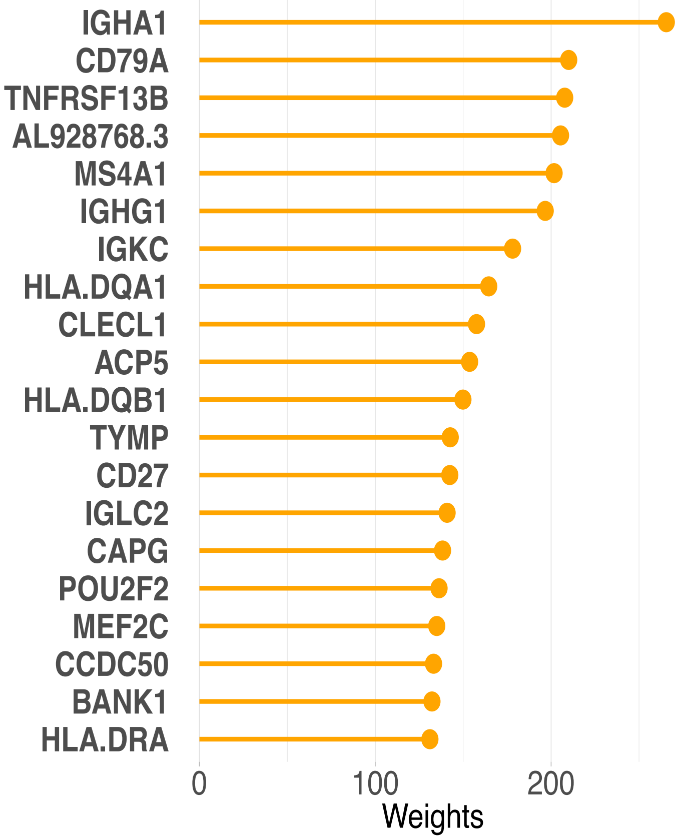

Imm GEM 57

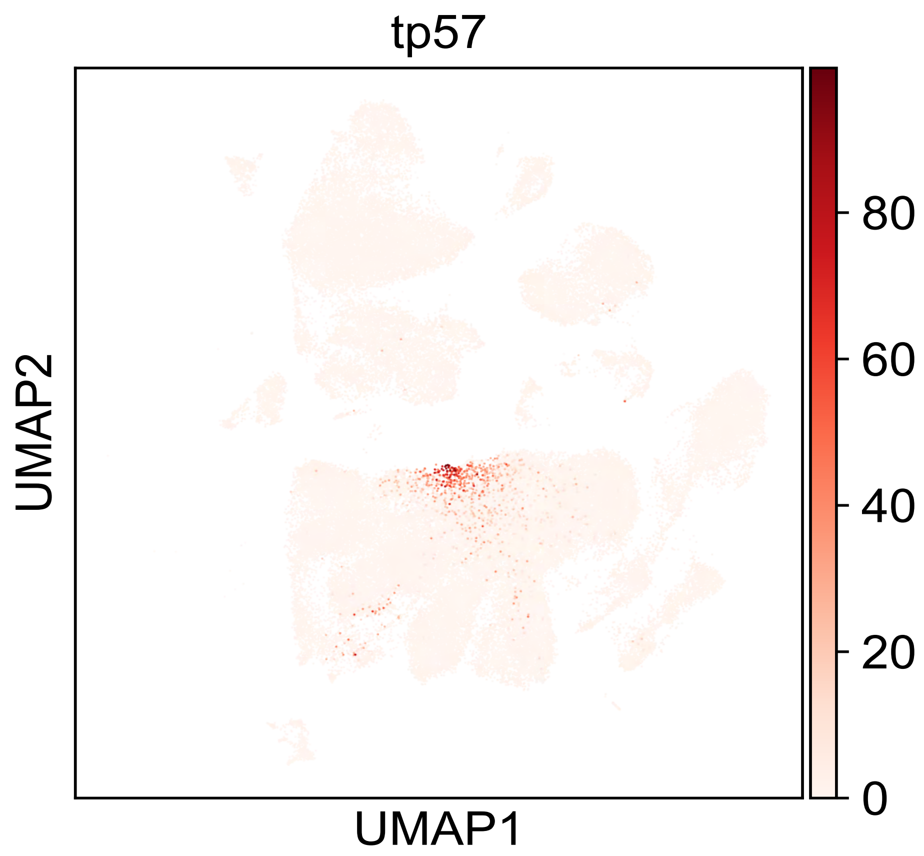

Cell Type

CD4 T cells

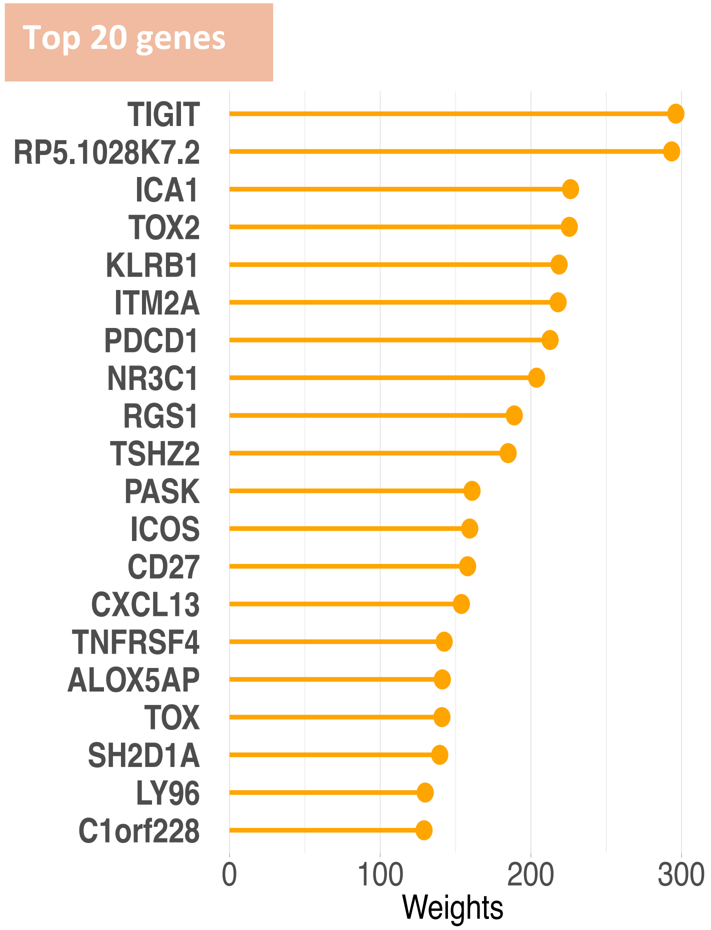

Imm GEM 61

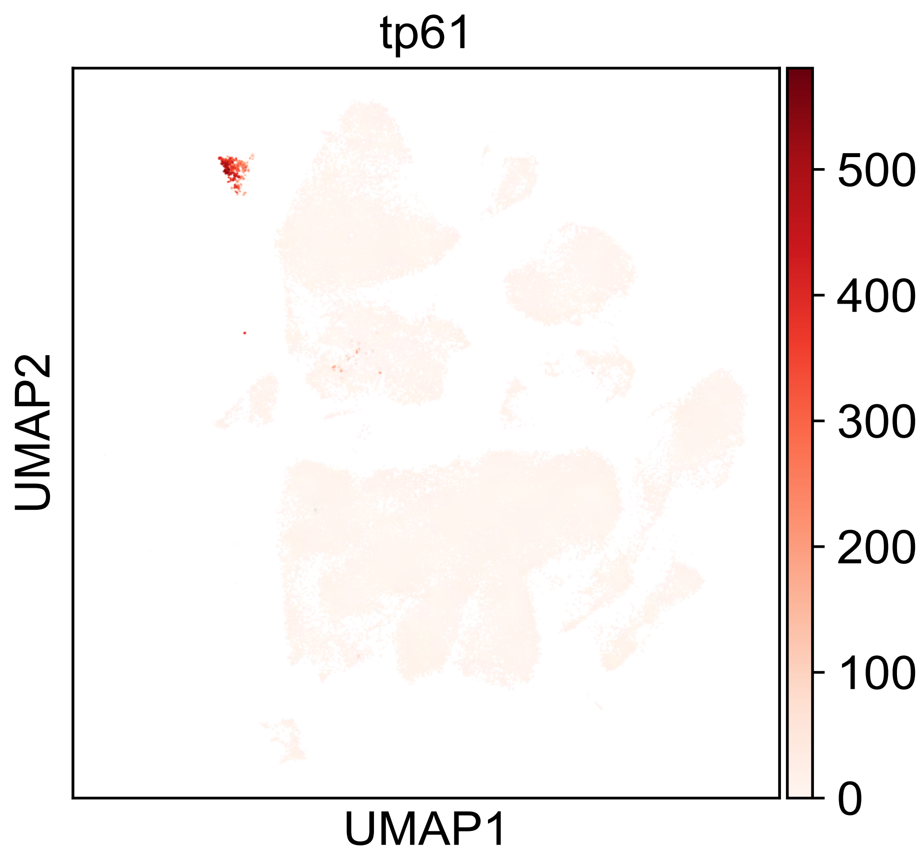

Cell Type

Dendritic

Top 20 genes

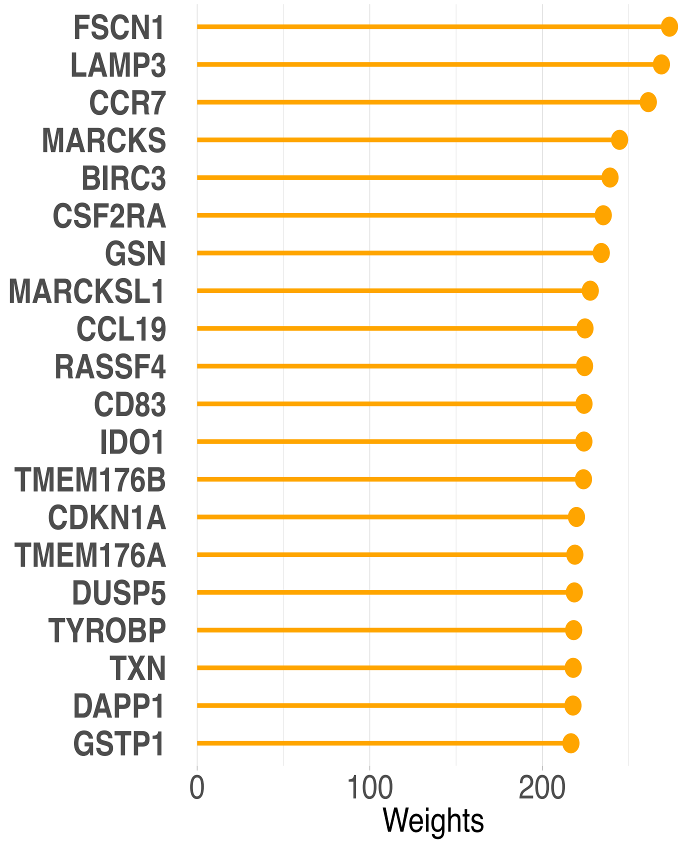

Imm GEM 62

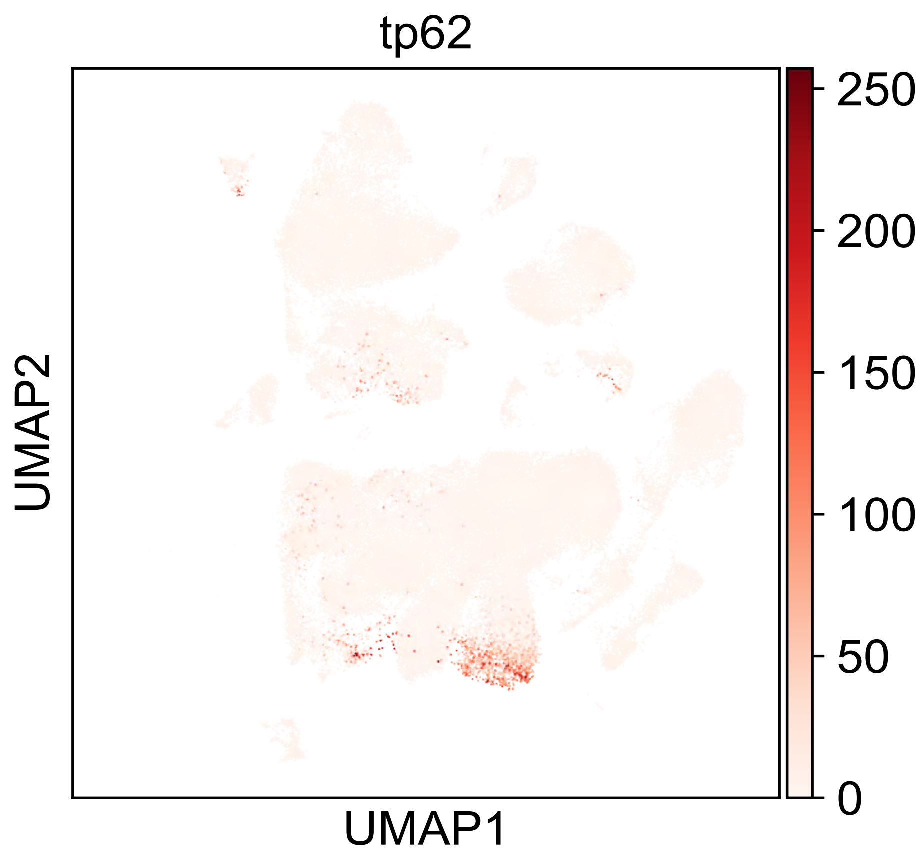

Cell Type
Tregs
CD8 T cells

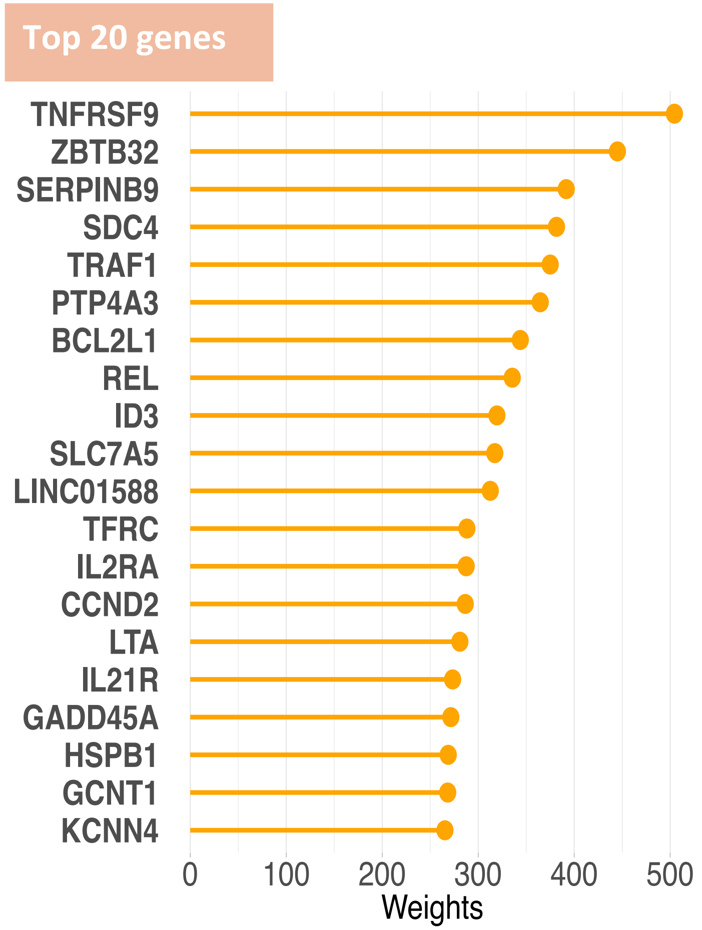

Imm GEM 64

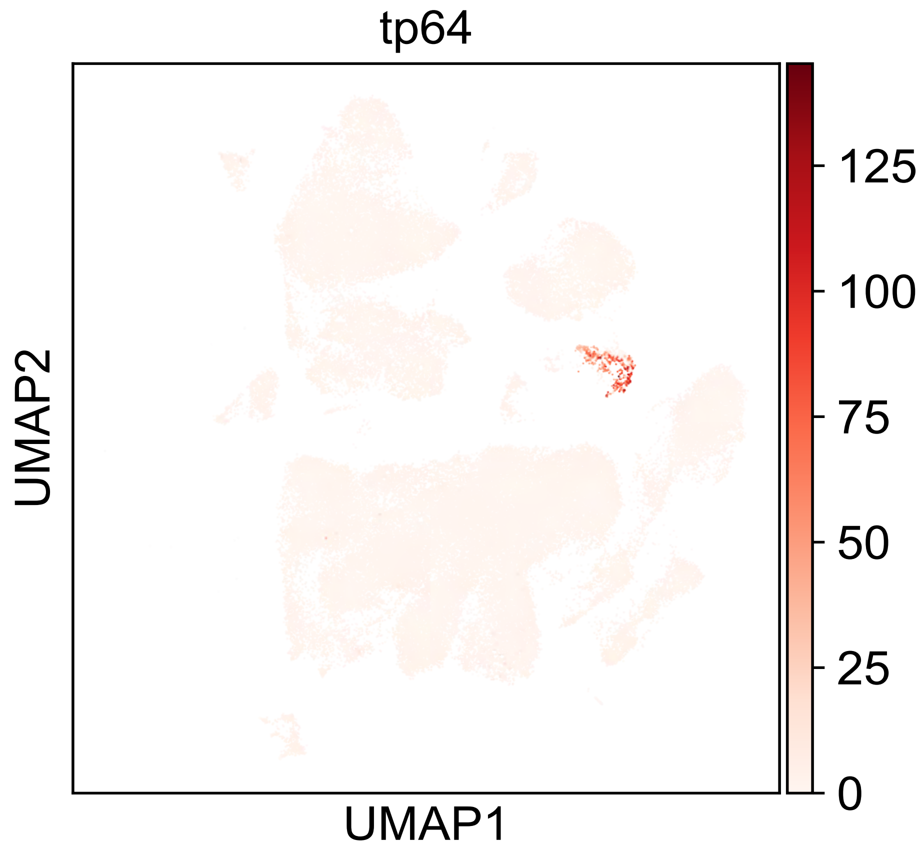

Cell Type

B cells

Top 20 genes

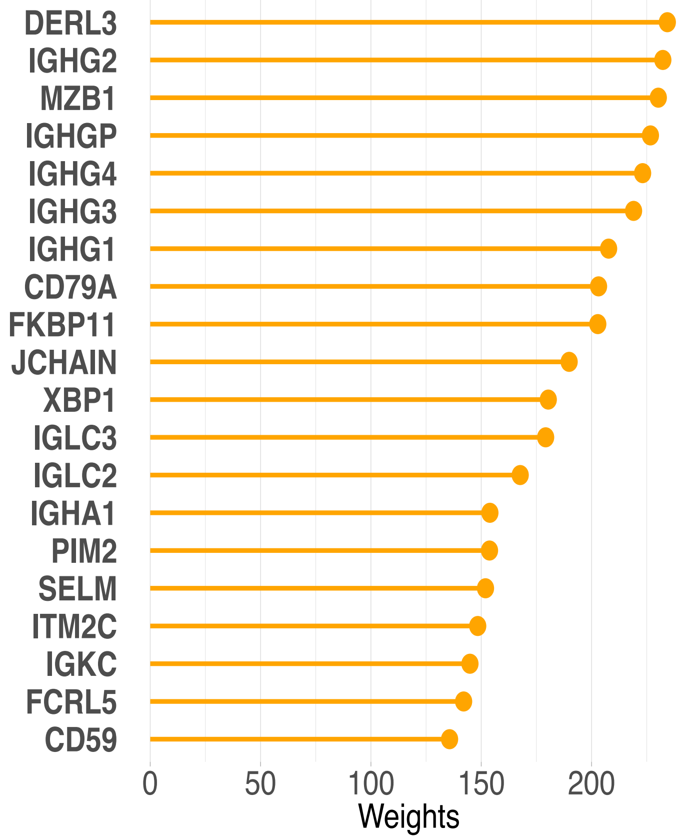

Epi GEM 1

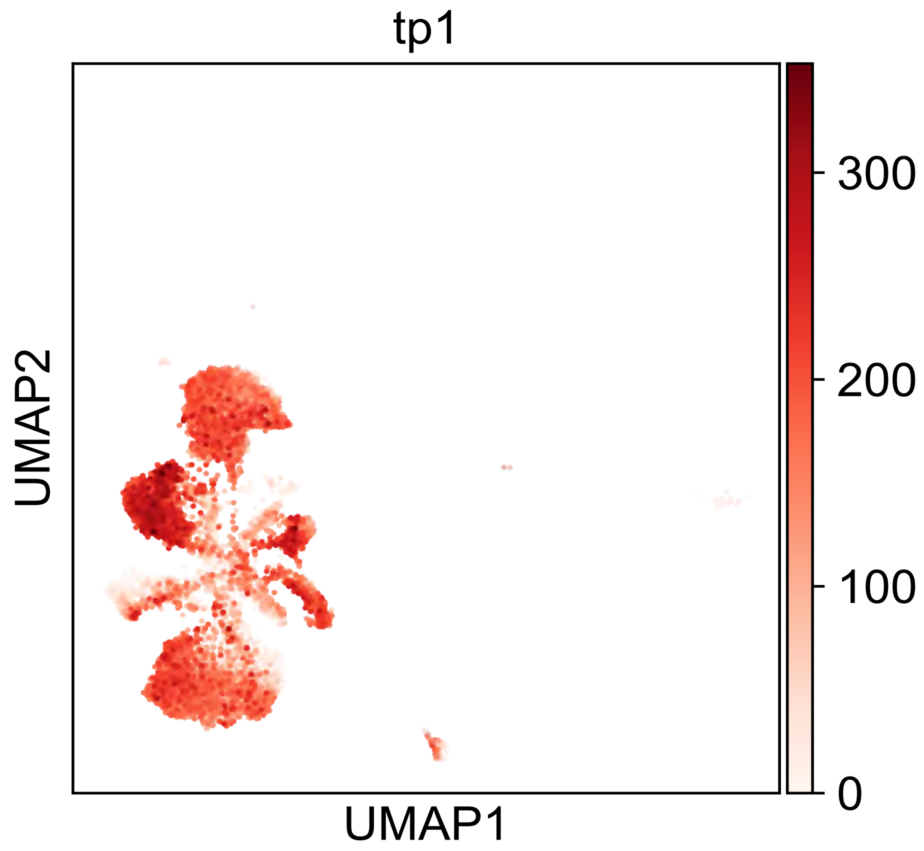

Cell Type

Epi_2

Epi_3

Epi_4

Epi_5

Epi_6

Epi_7

Epi_8

Top 20 genes

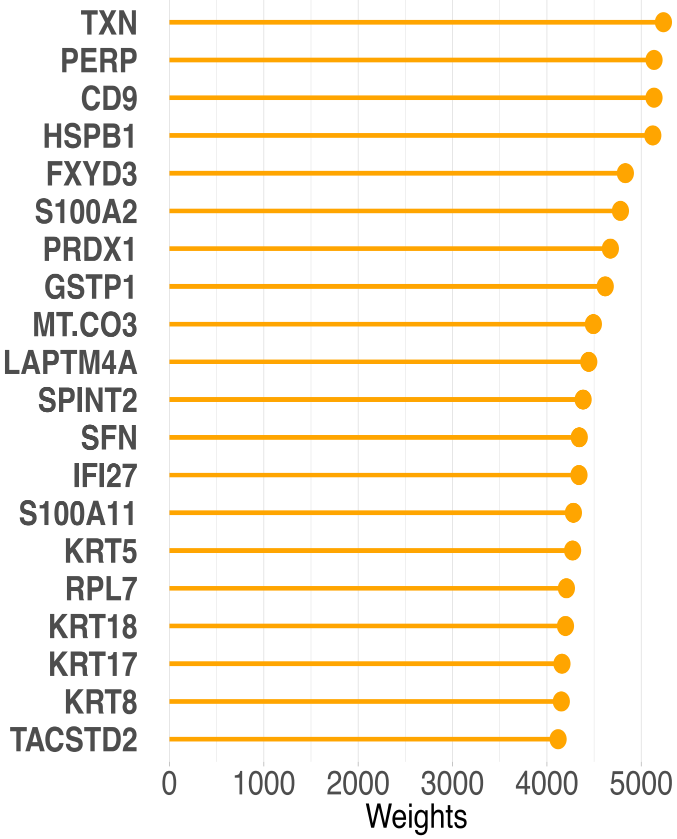

Epi GEM 2

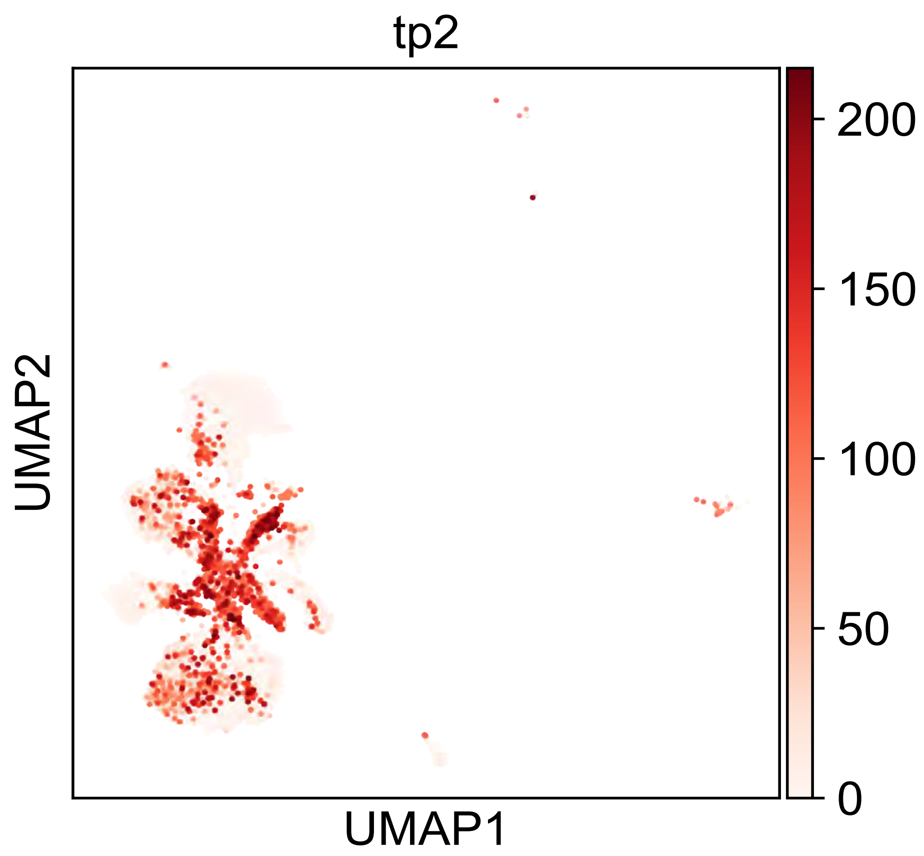

Cell Type

Epi_1

Epi_4

Top 20 genes

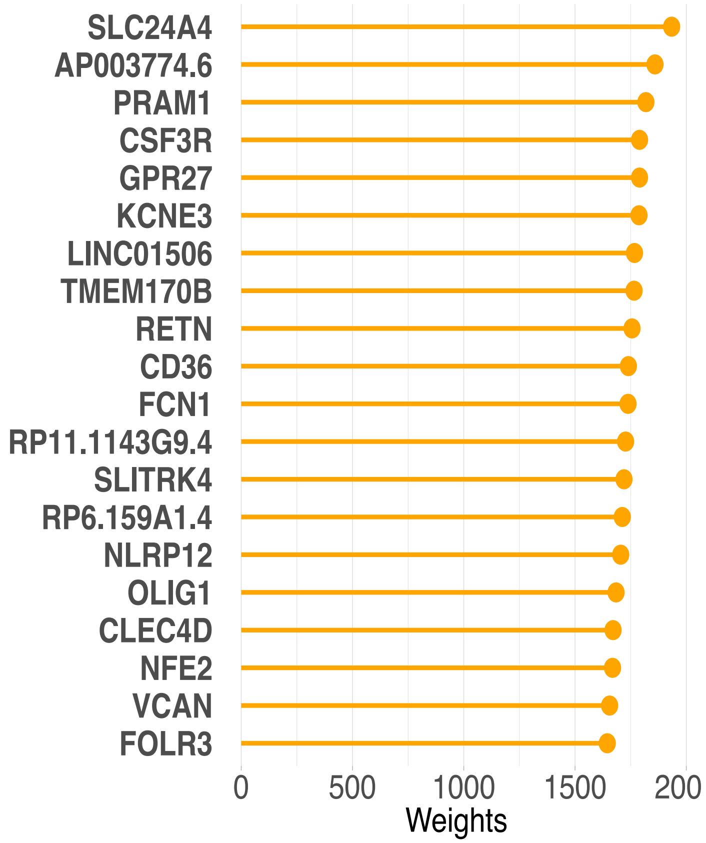

Epi GEM 3

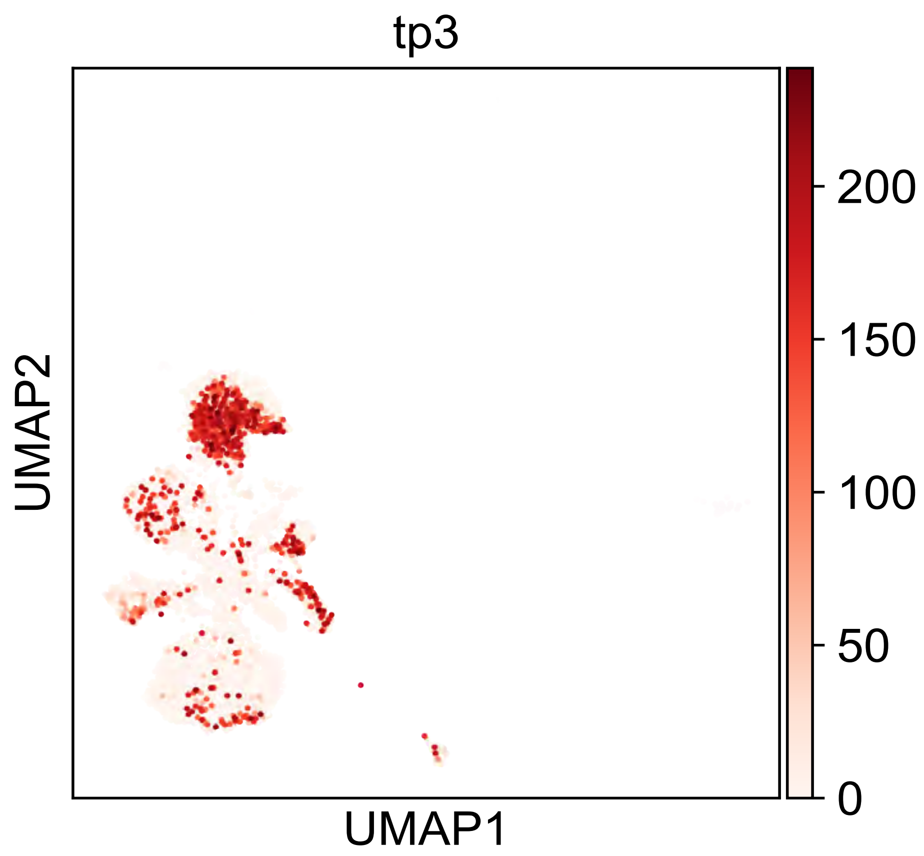

Cell Type

Epi_1

Epi_2

Epi_3

Top 20 genes

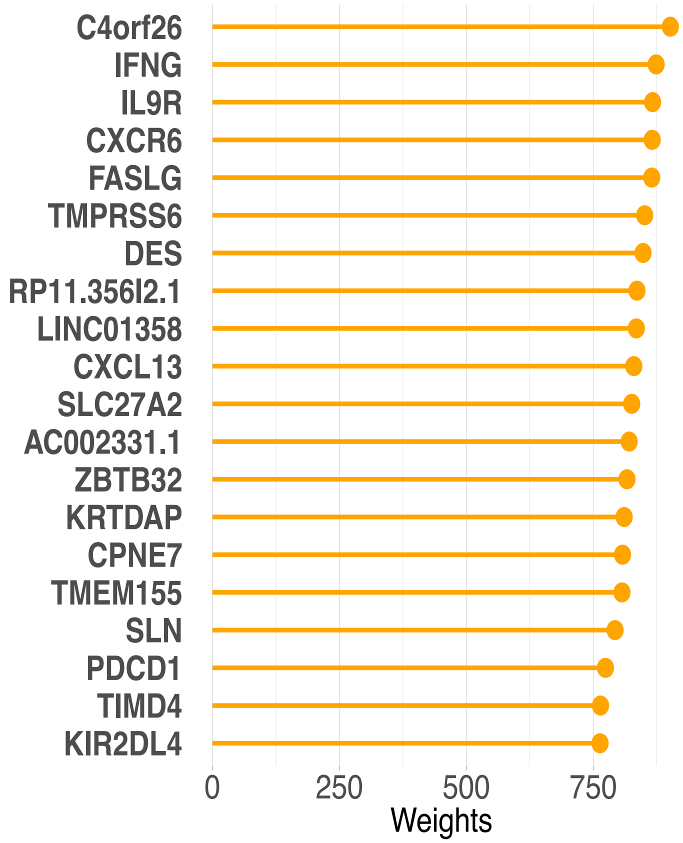

Epi GEM 4

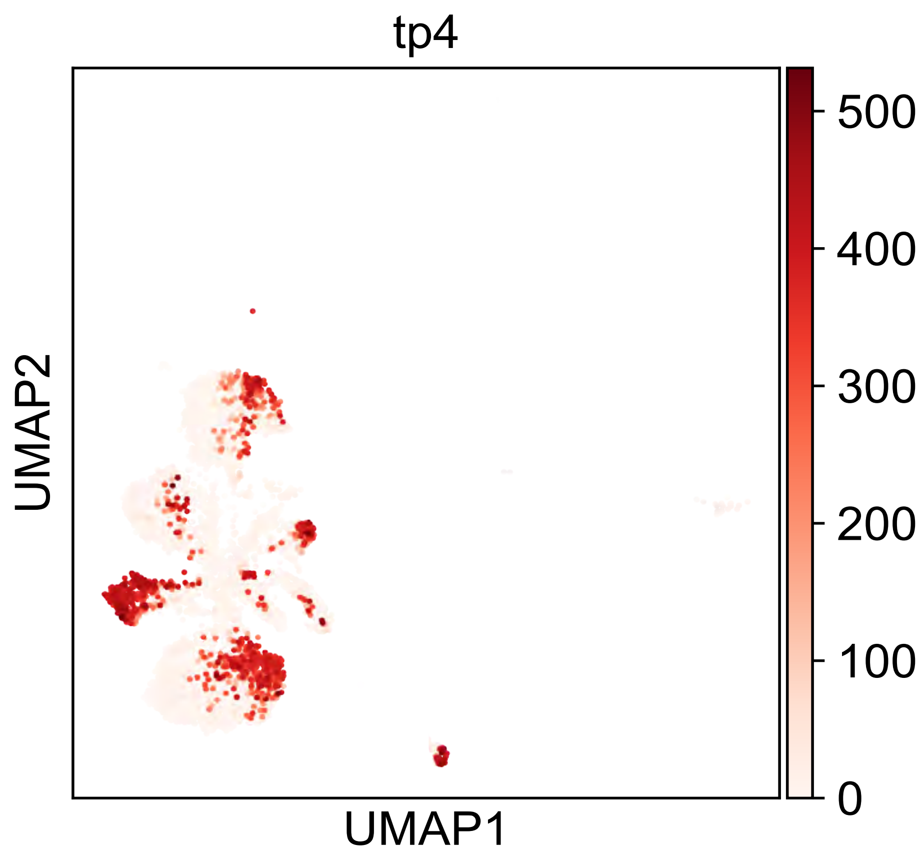

Cell Type

Epi_5

Epi_2

Epi_8

Top 20 genes

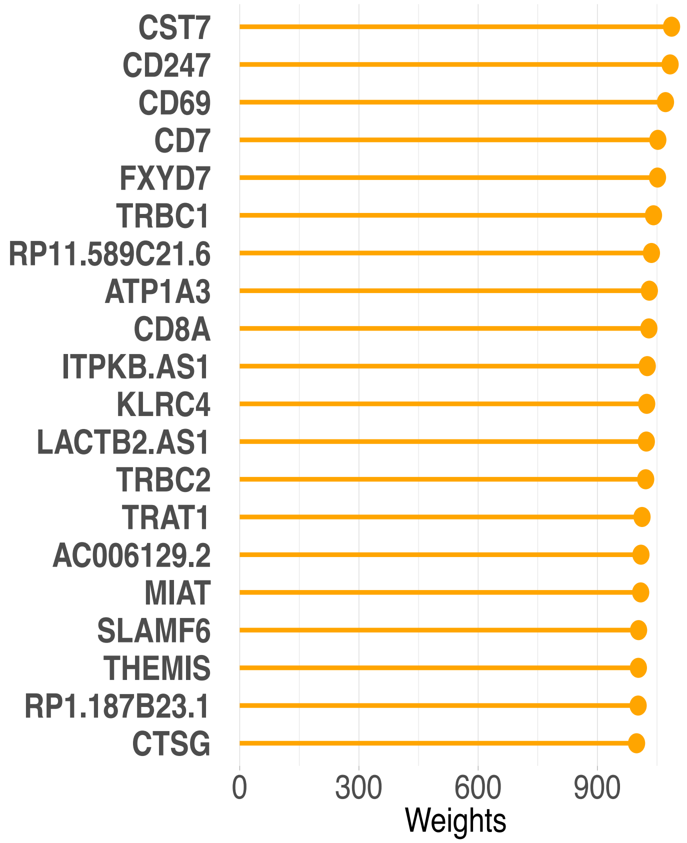

Epi GEM 5

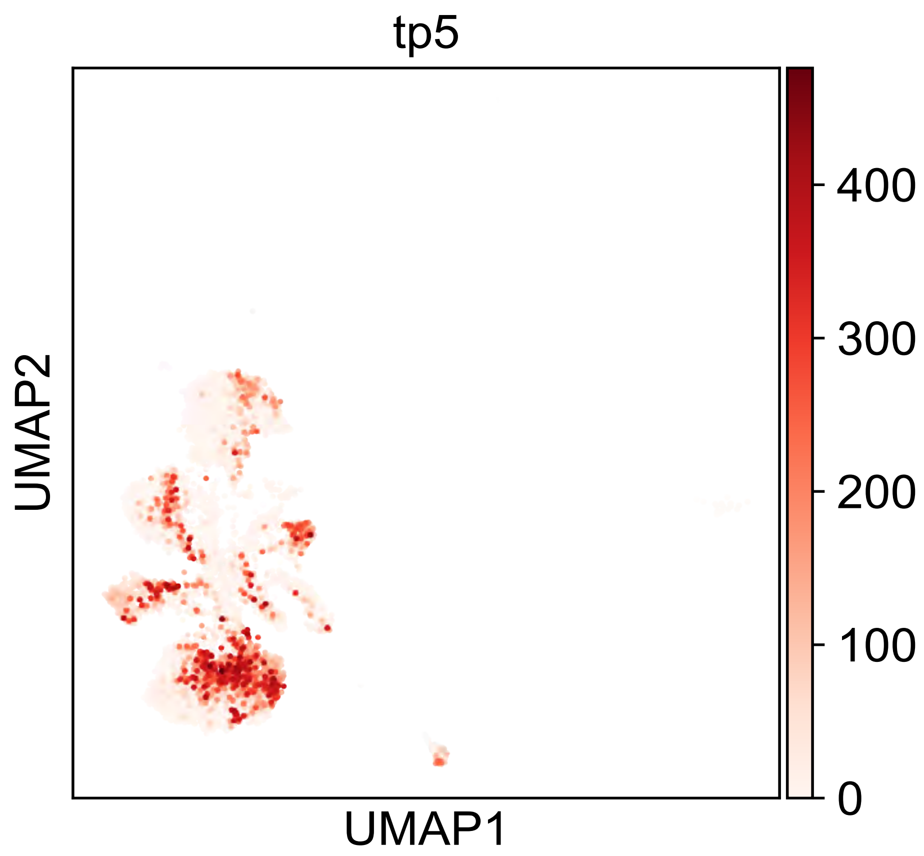

Cell Type

Epi_1

Epi_2

Epi_3

Top 20 genes

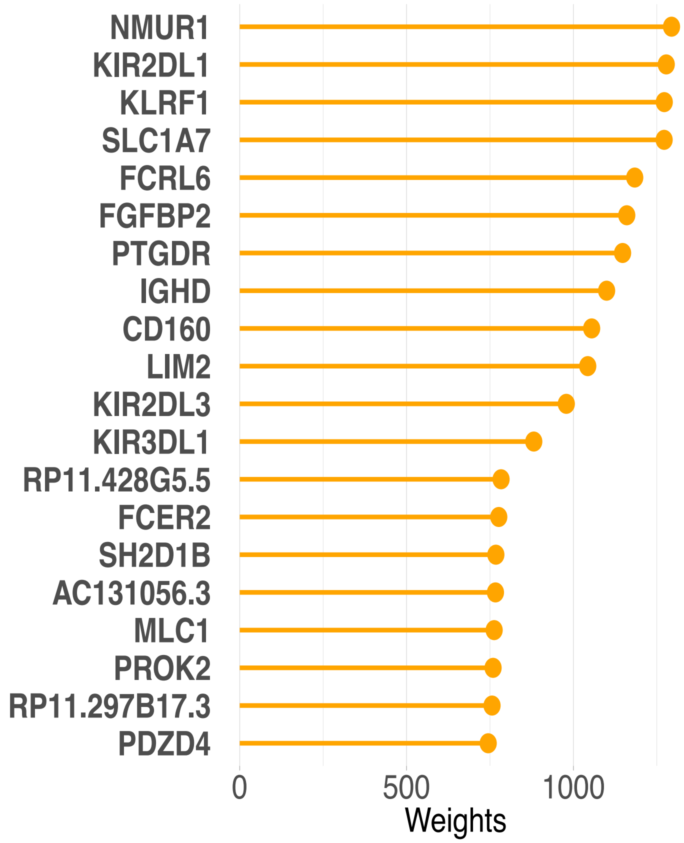

Epi GEM 6

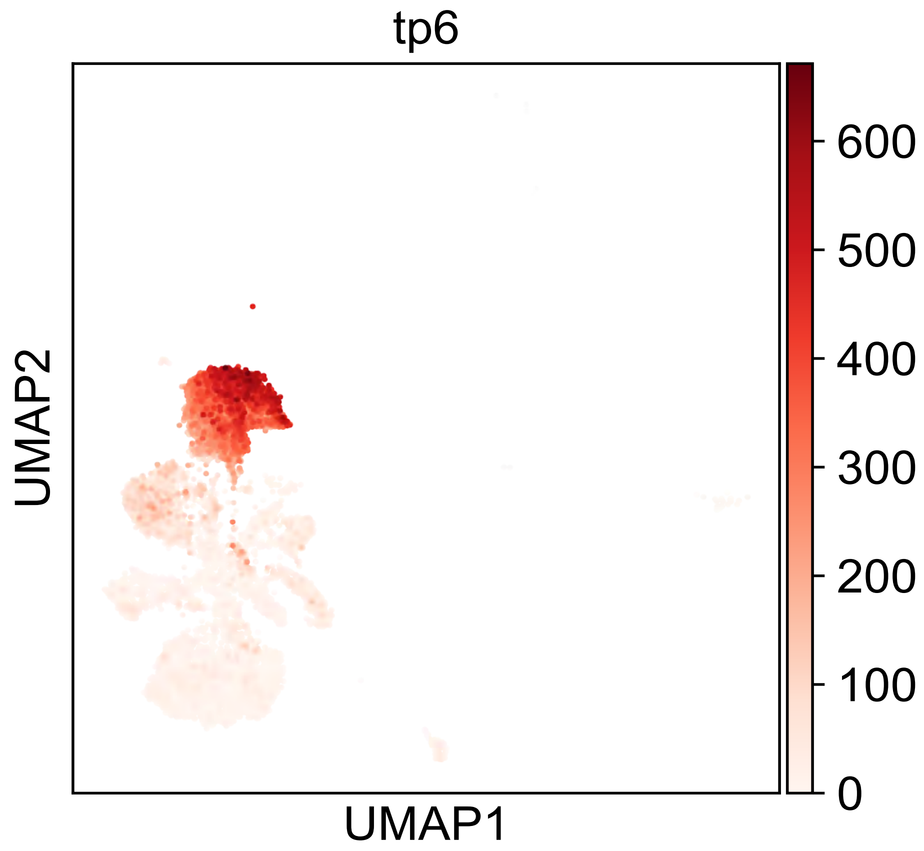

Cell Type

Epi_7

Epi_8

Top 20 genes

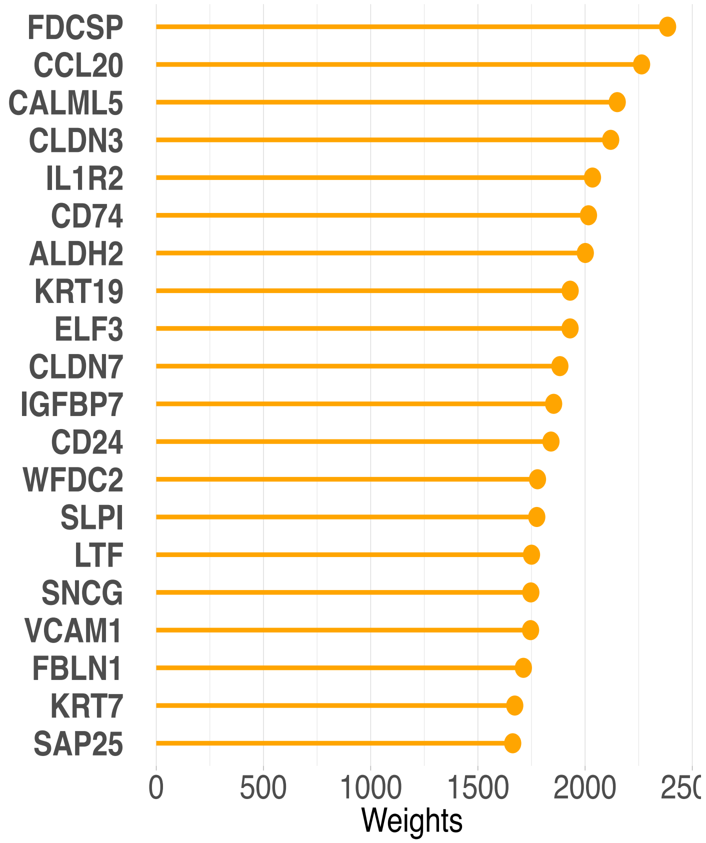

Epi GEM 7

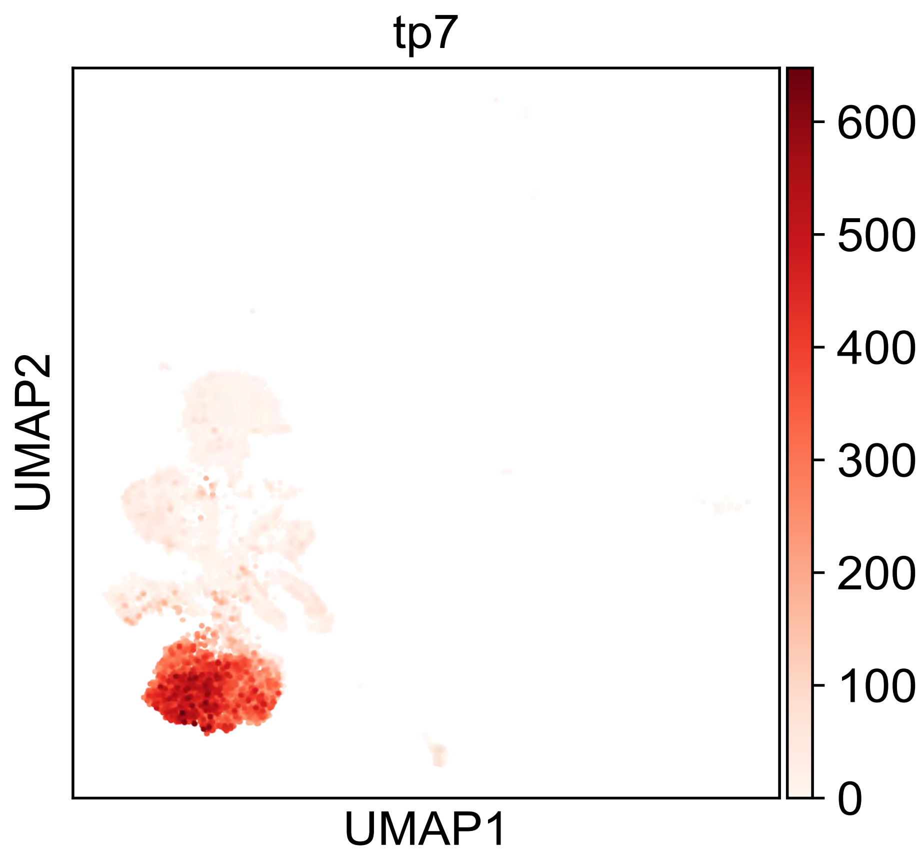

Cell Type

Epi_4

Epi_5

Top 20 genes

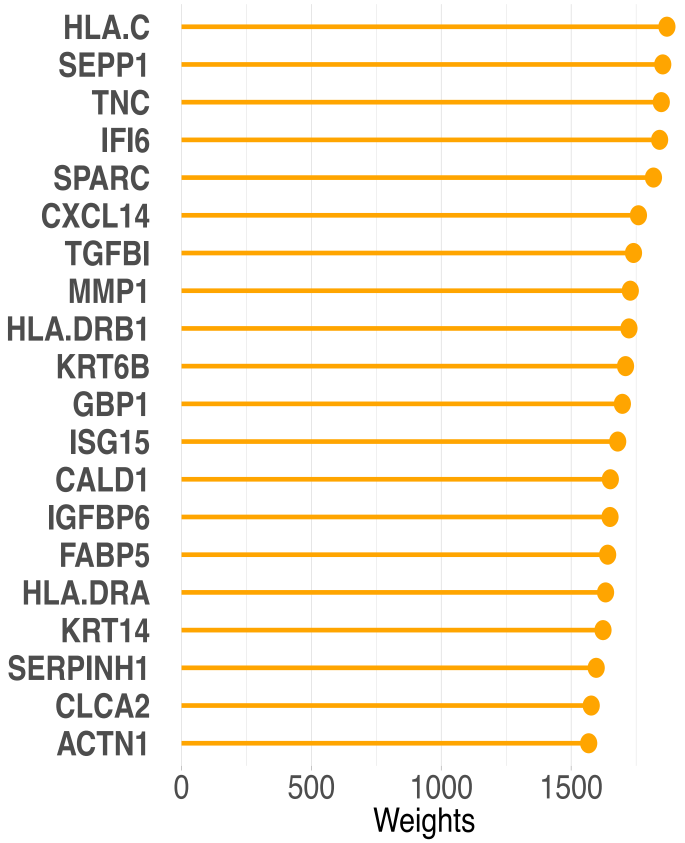

Epi GEM 8

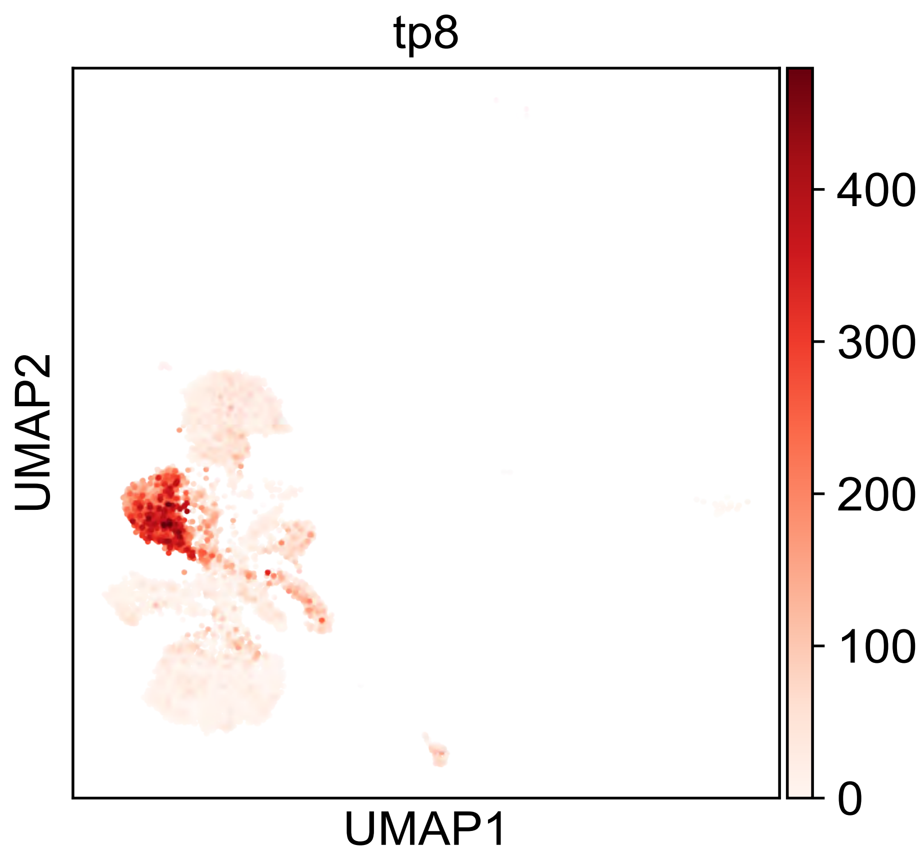

Cell Type

Epi_6

Top 20 genes

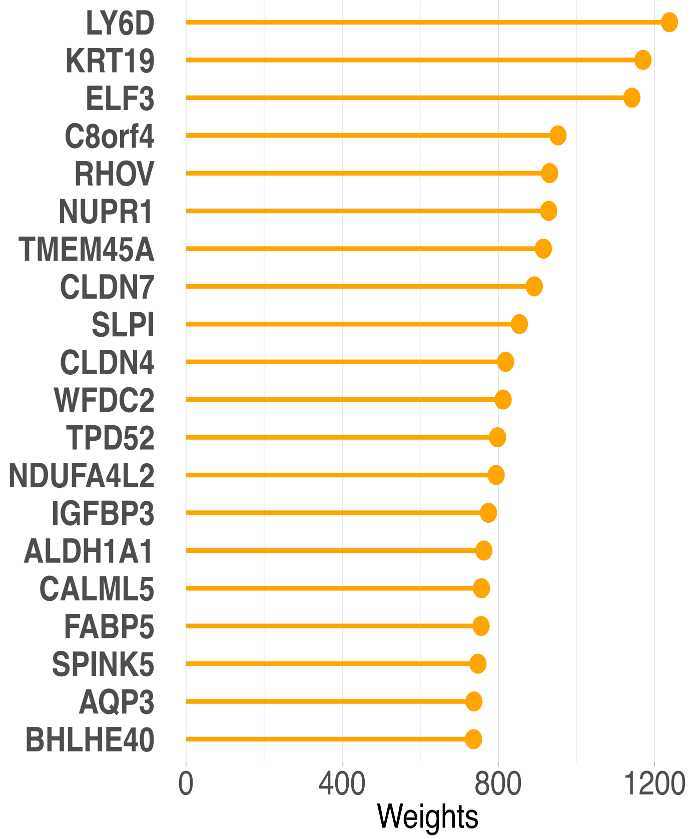

Epi GEM 9

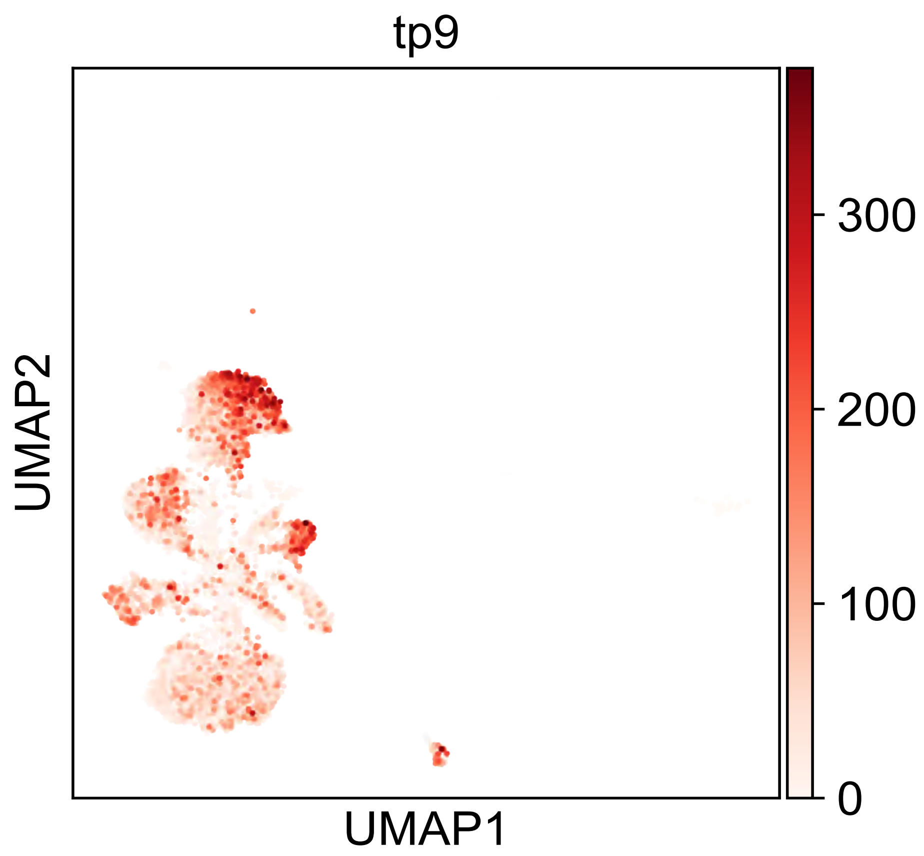

Cell Type

Epi_8

Epi_2

Epi_6

Top 20 genes

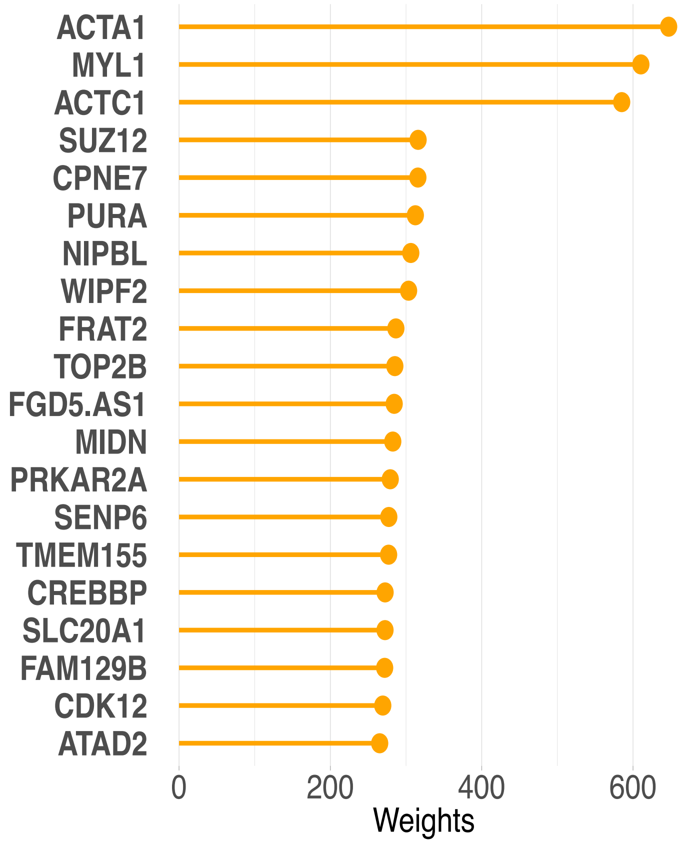

Epi GEM 10

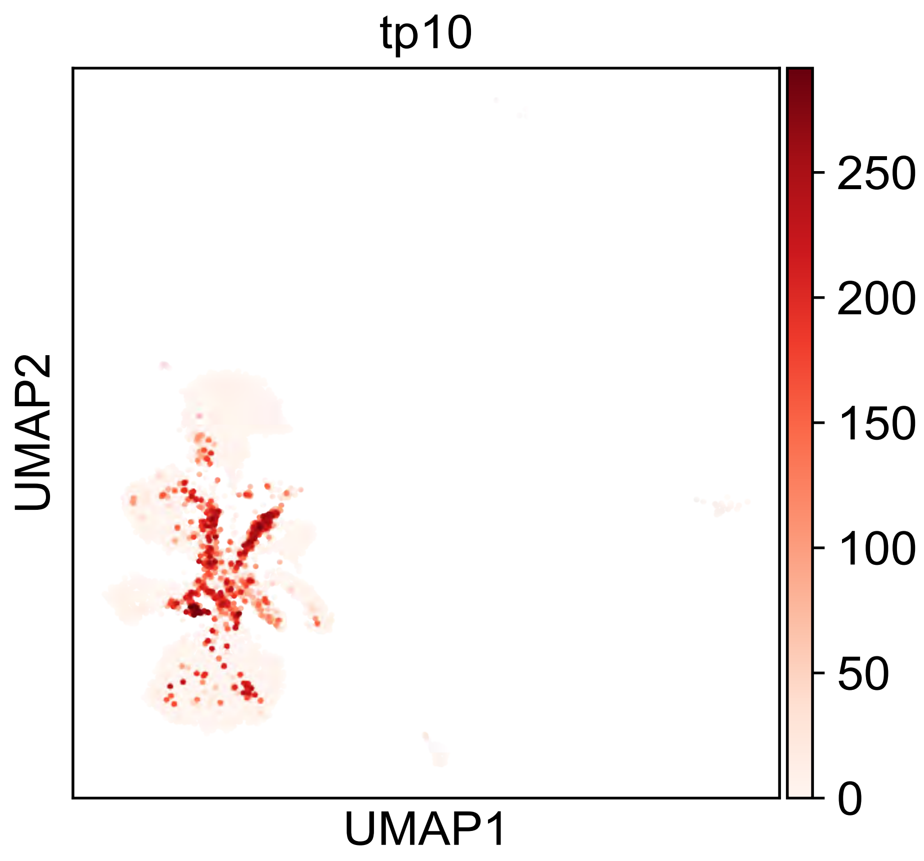

Cell Type

Epi_1

Top 20 genes

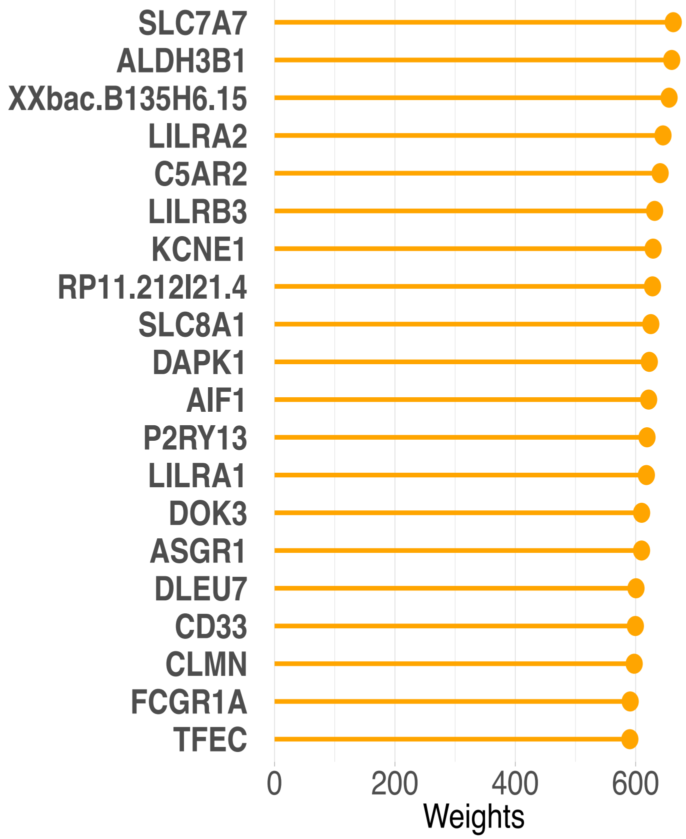

Epi GEM 11

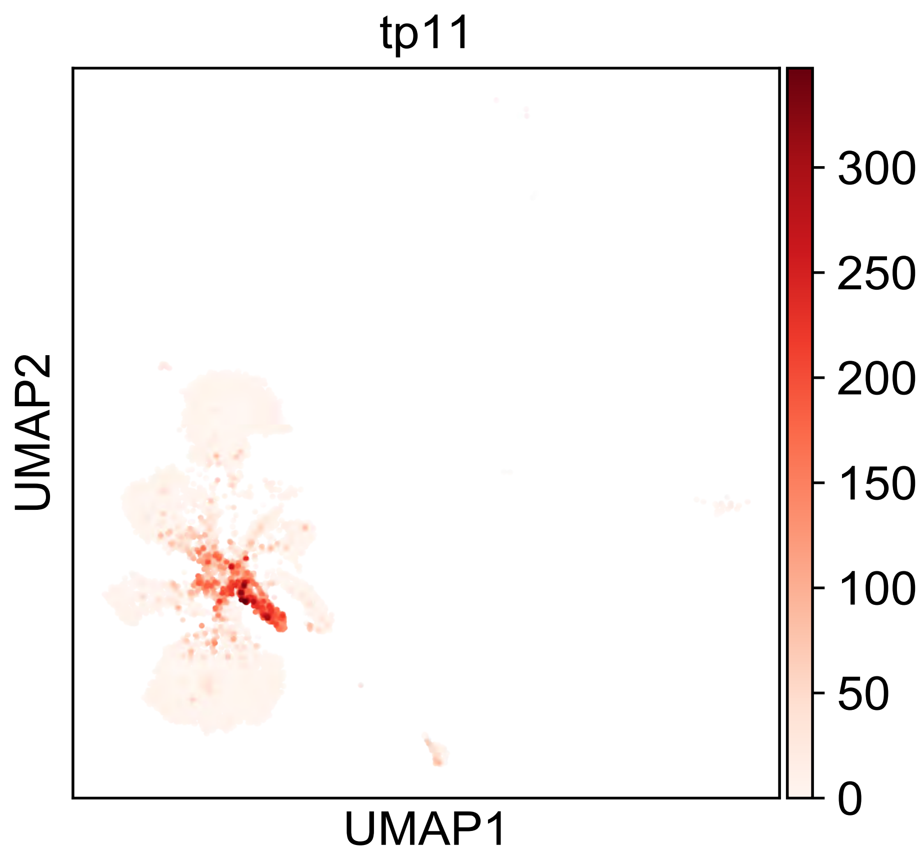

Cell Type

Epi_1

Top 20 genes

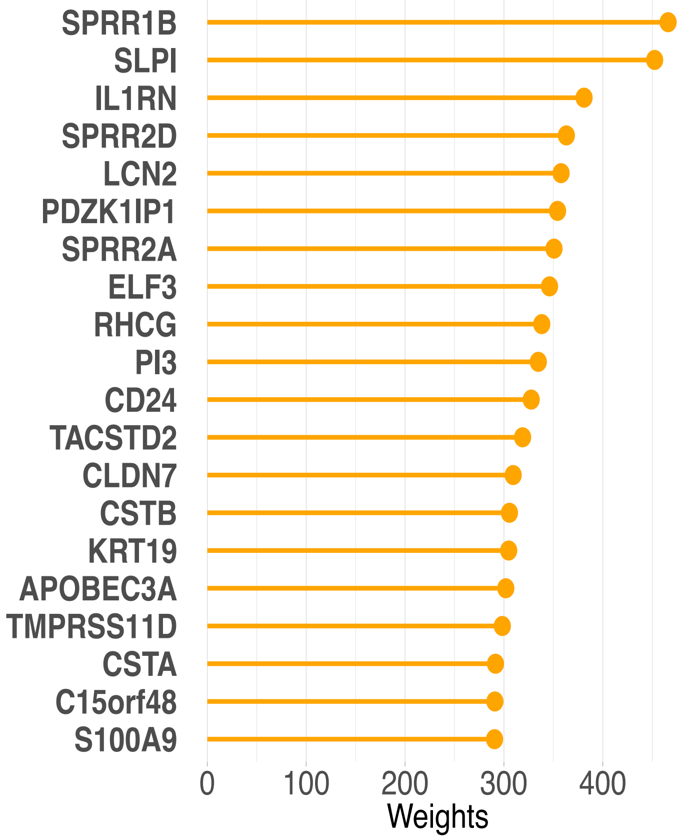

Epi GEM 12

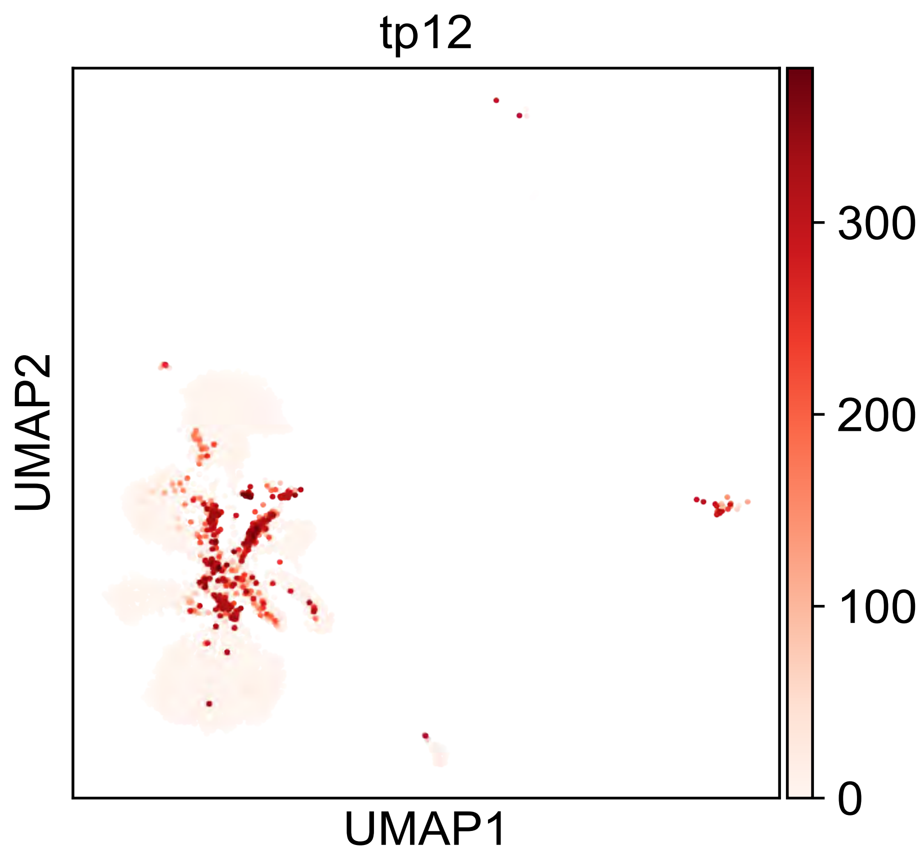

Cell Type

Epi_1

Top 20 genes

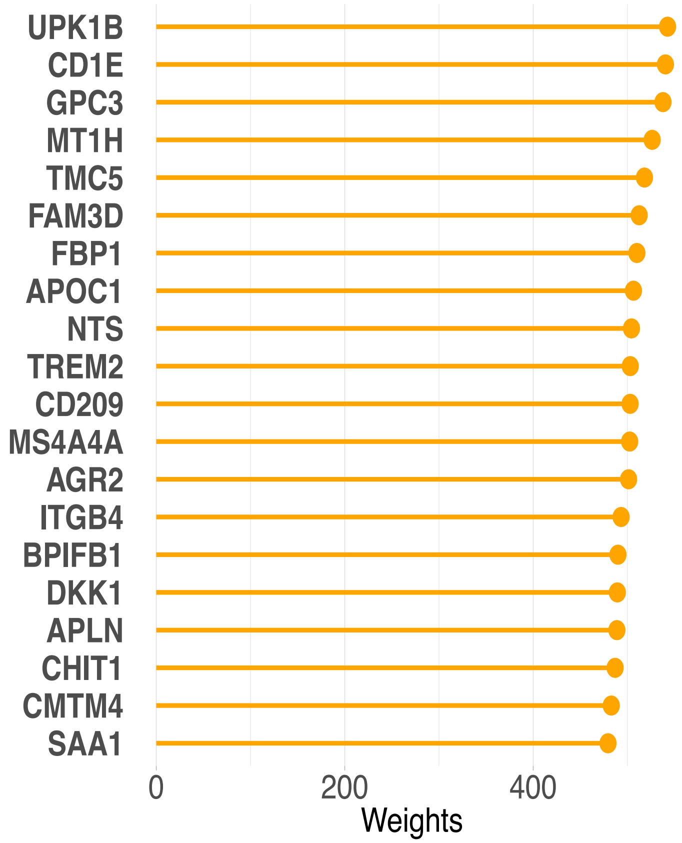

Epi GEM 14

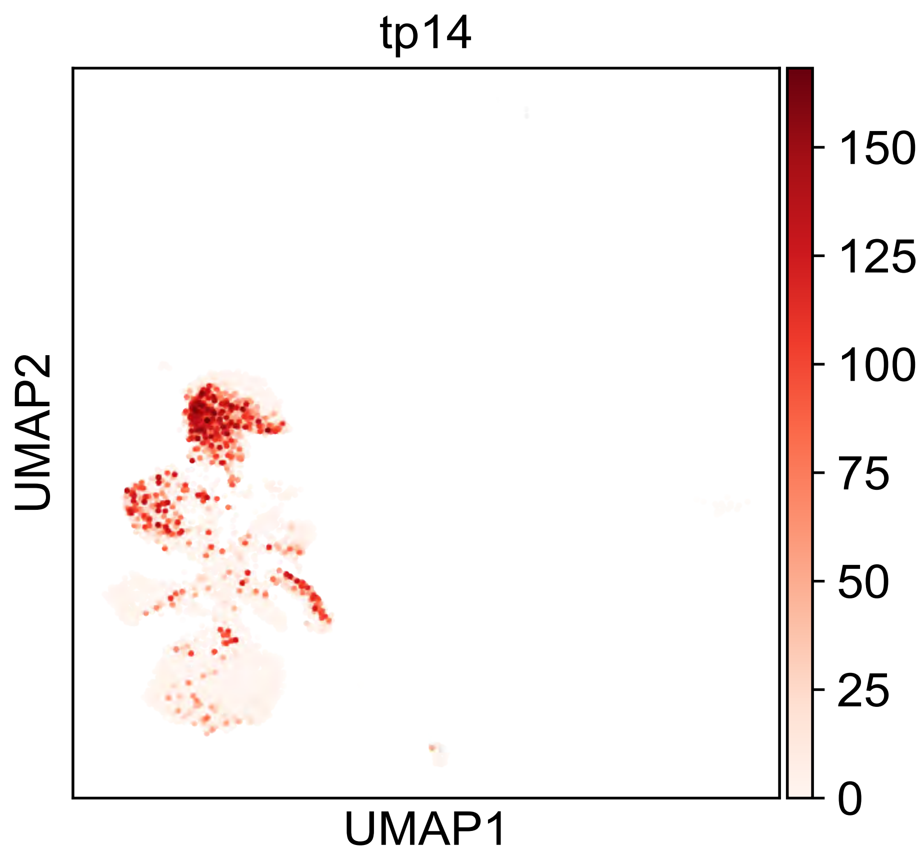

Cell Type

Epi_3

Epi_7

Top 20 genes

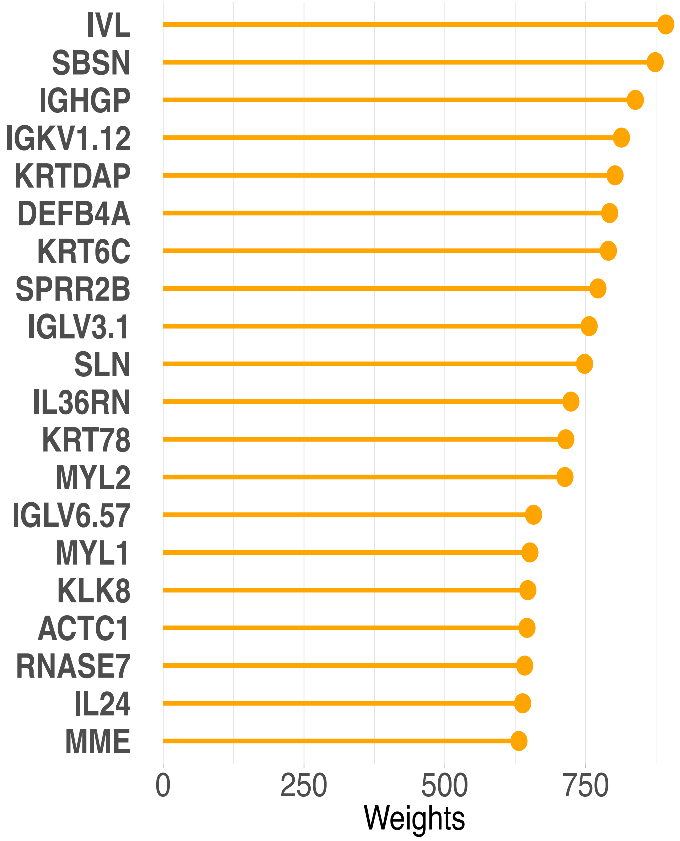

Epi GEM 15

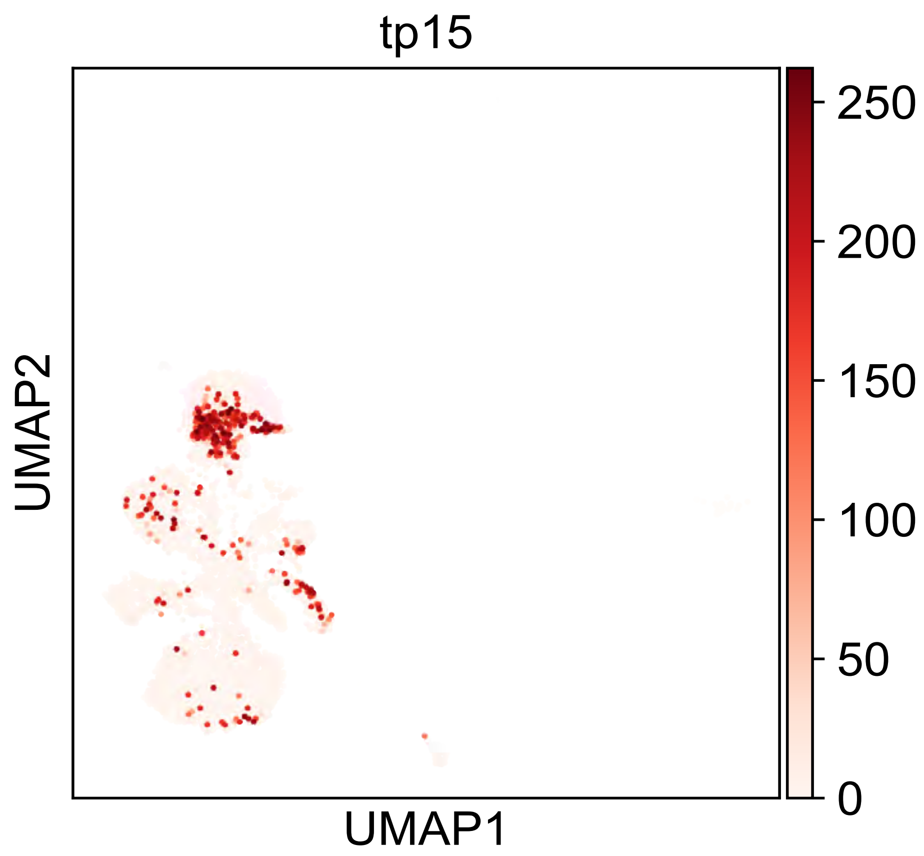

Cell Type

Epi_3

Epi_7

Top 20 genes

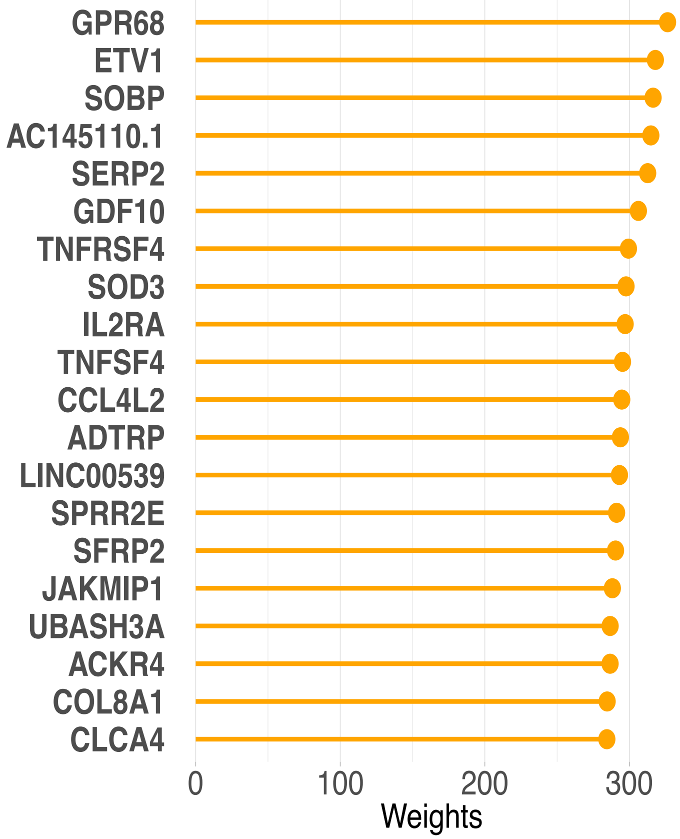

Epi GEM 16

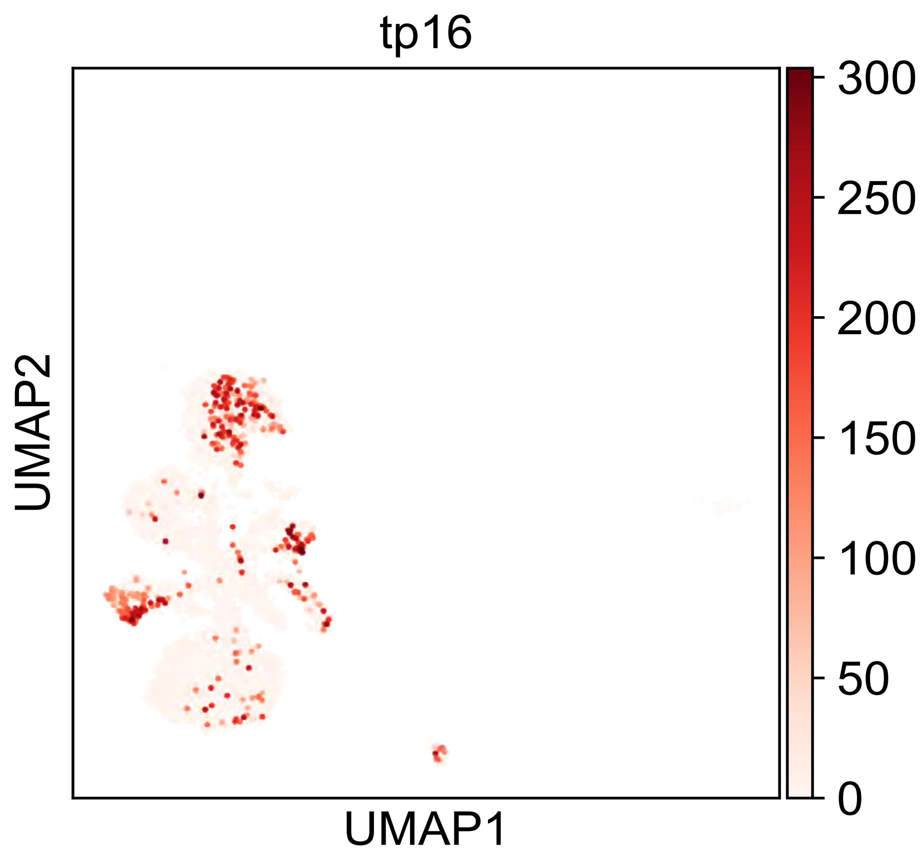

Cell Type

Epi_3

Epi_7

Top 20 genes

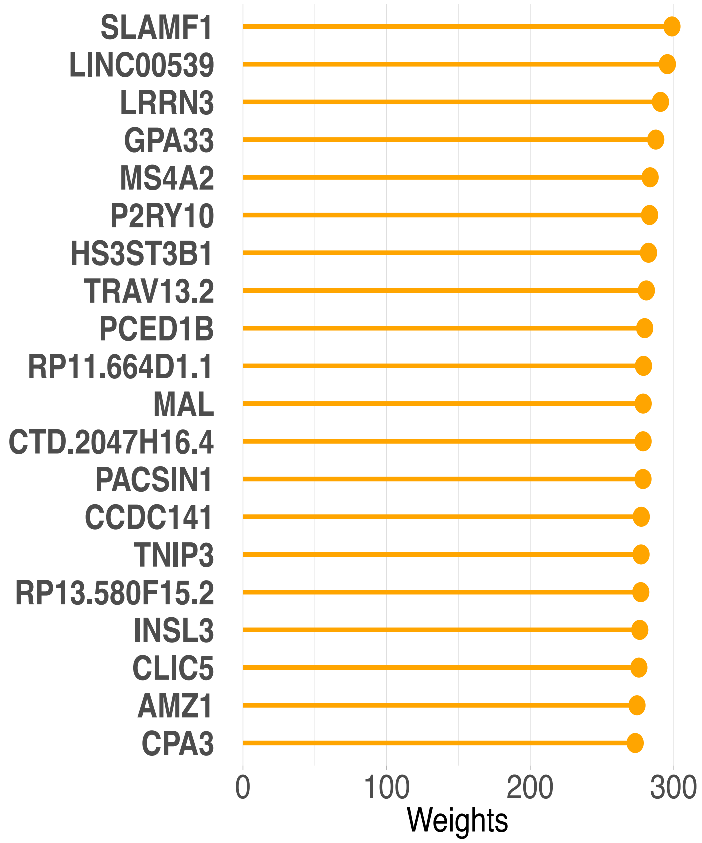

Epi GEM 17

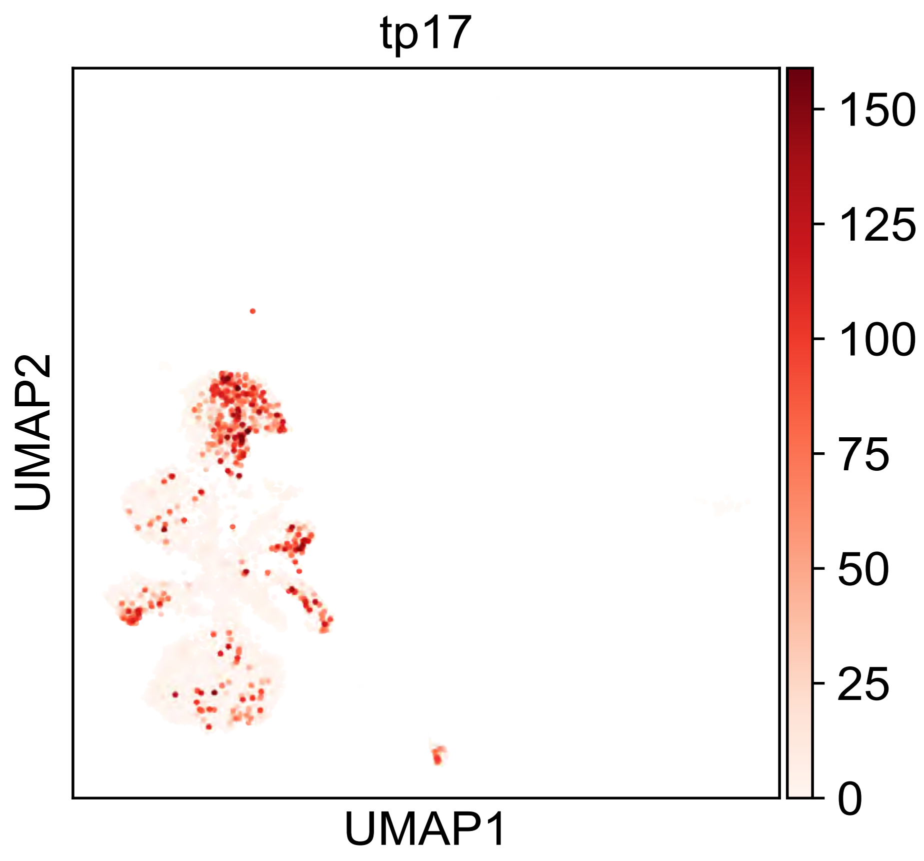

Cell Type

Epi_3

Epi_7

Top 20 genes

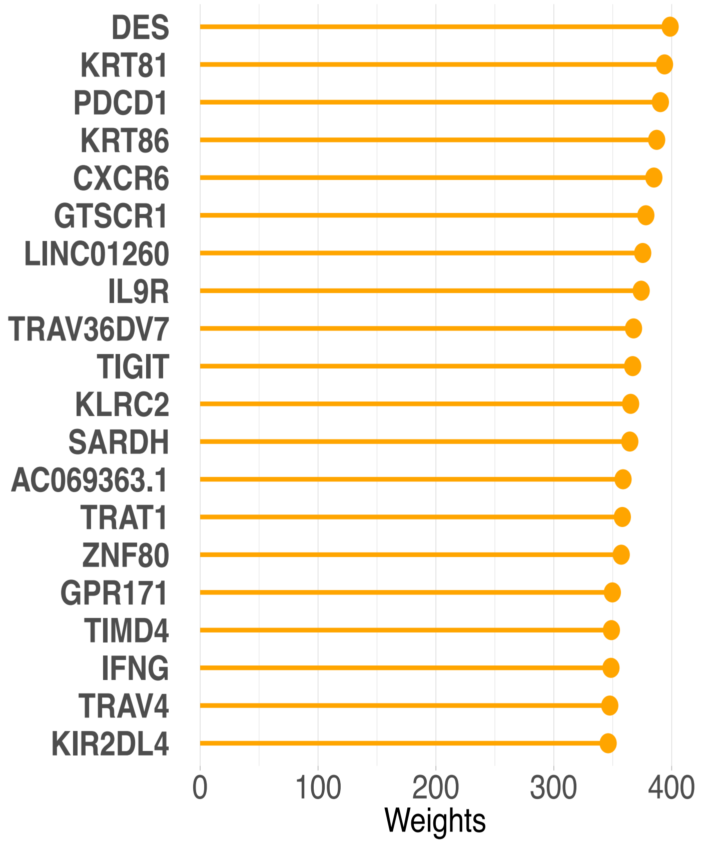

Epi GEM 18

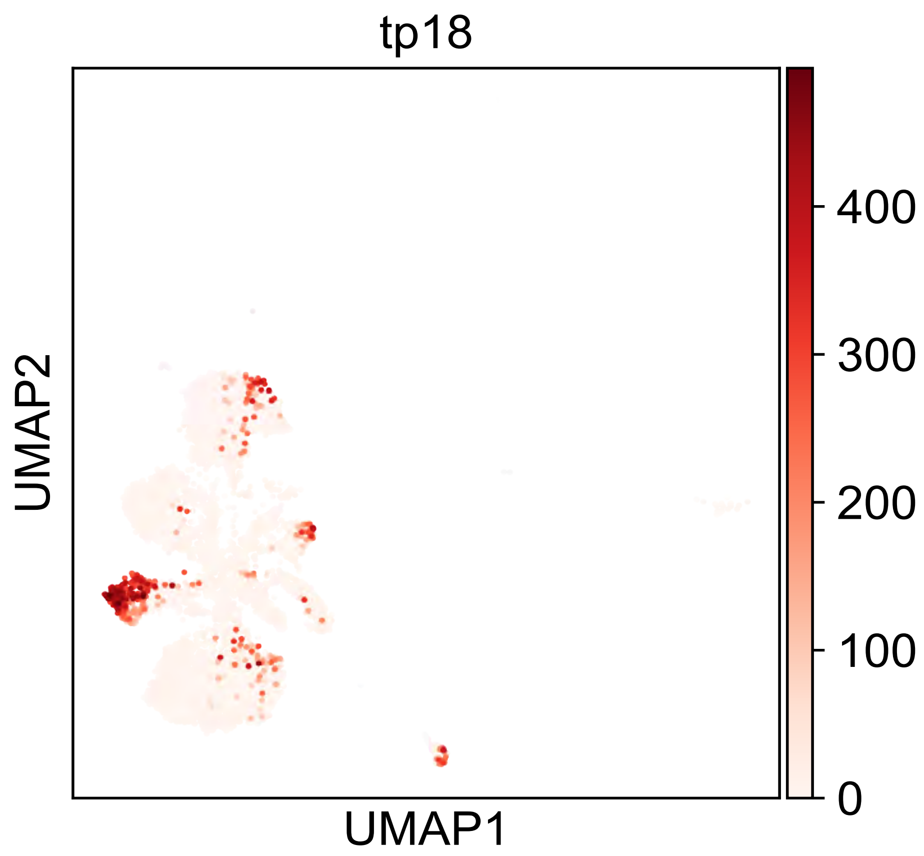

Cell Type

Epi_2

Top 20 genes

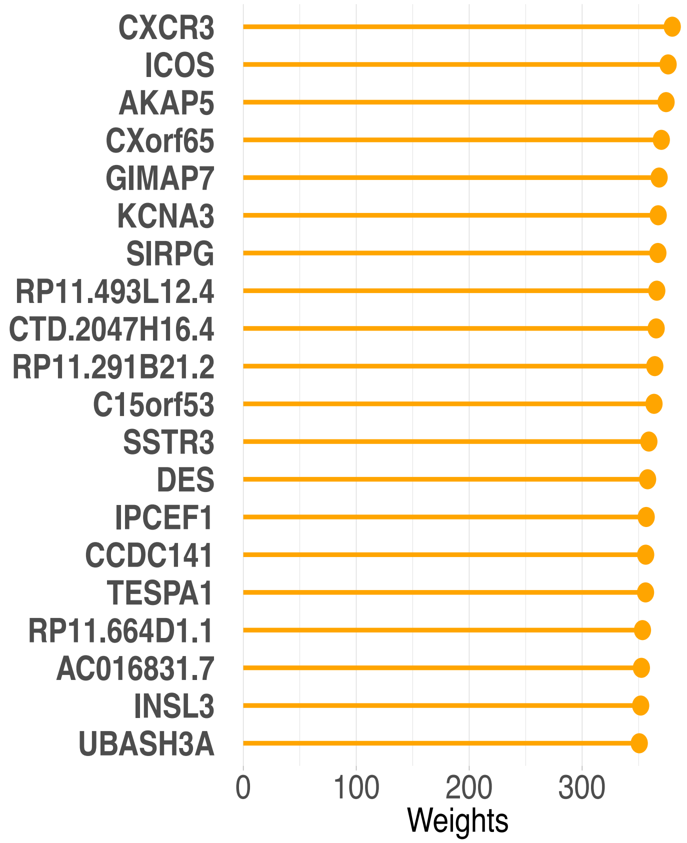

Epi GEM 20

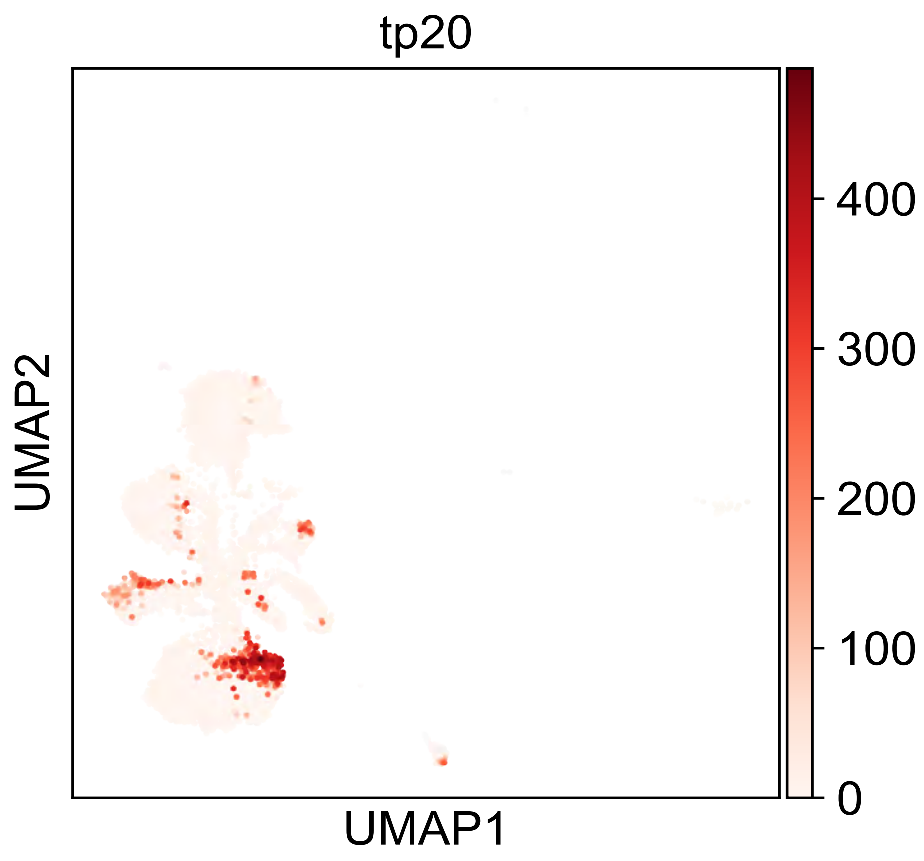

Cell Type

Epi_5

Epi_2

Top 20 genes

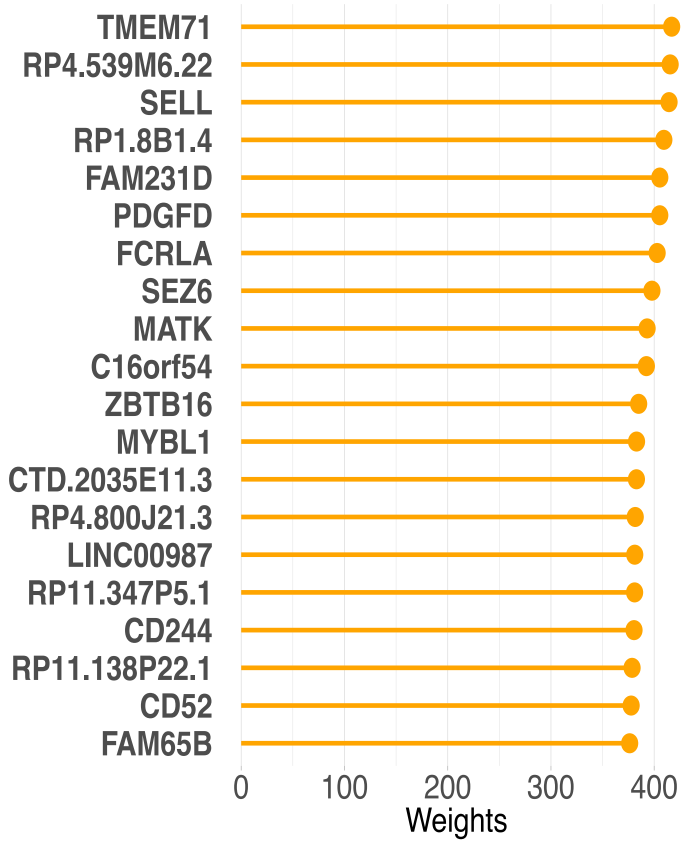

Epi GEM 21

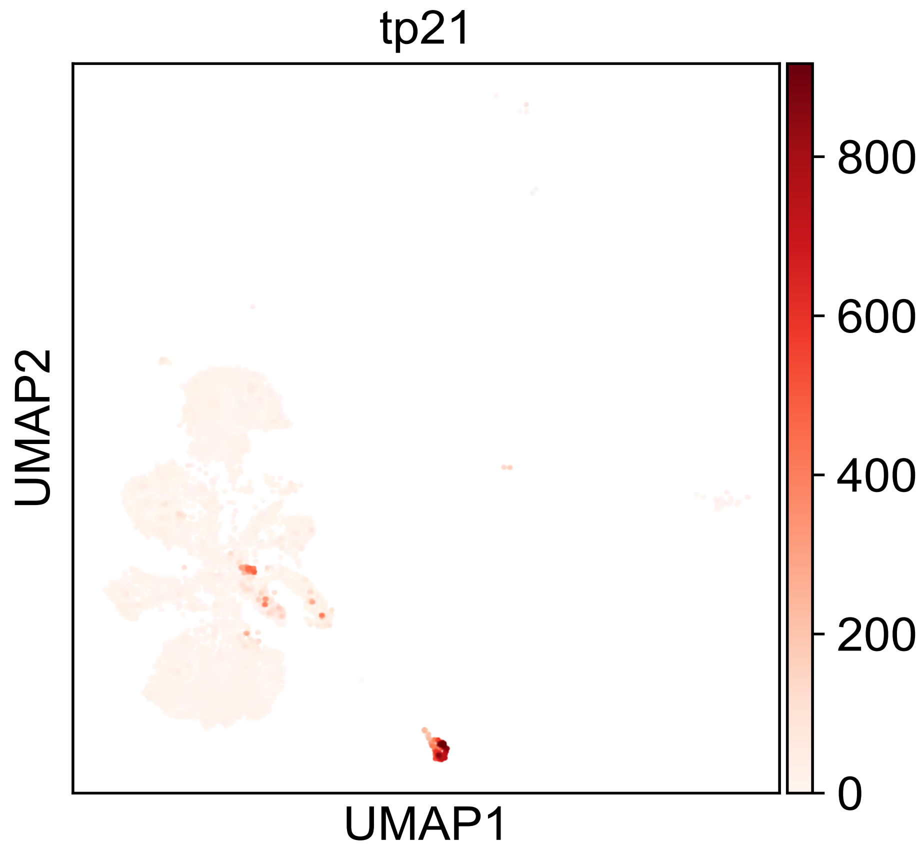

Cell Type

Epi_2

Epi_3

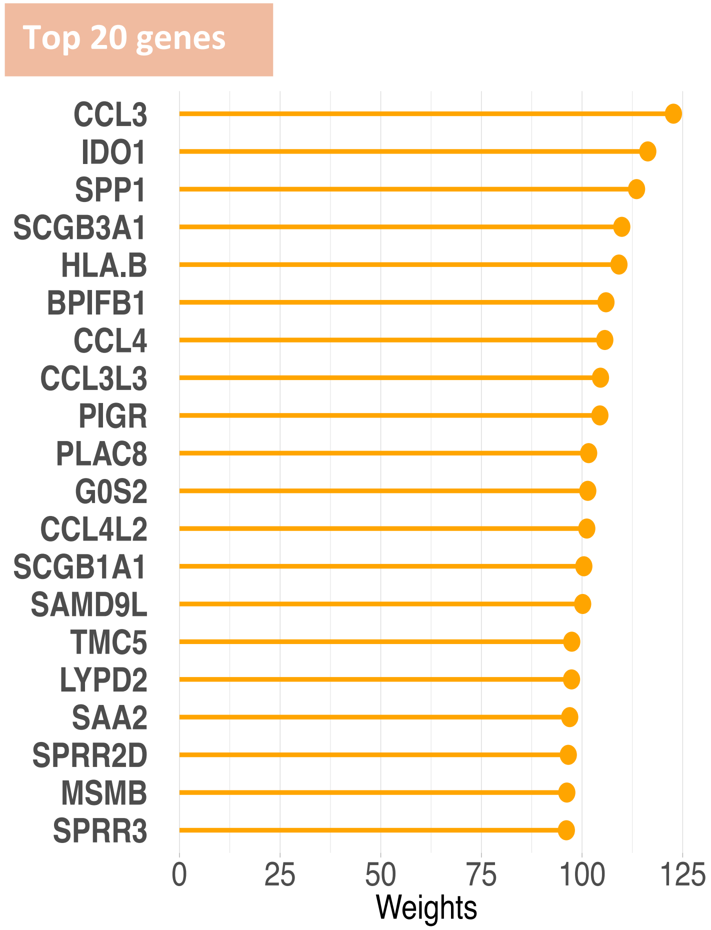

Epi GEM 22

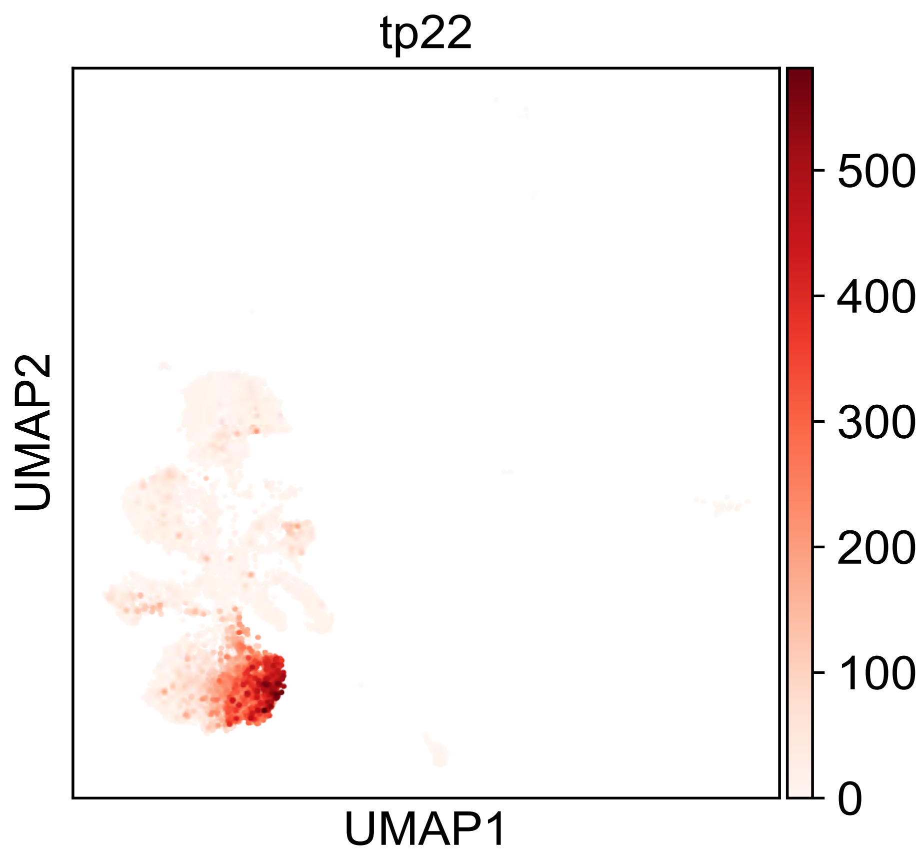

Cell Type

Epi_5

Top 20 genes

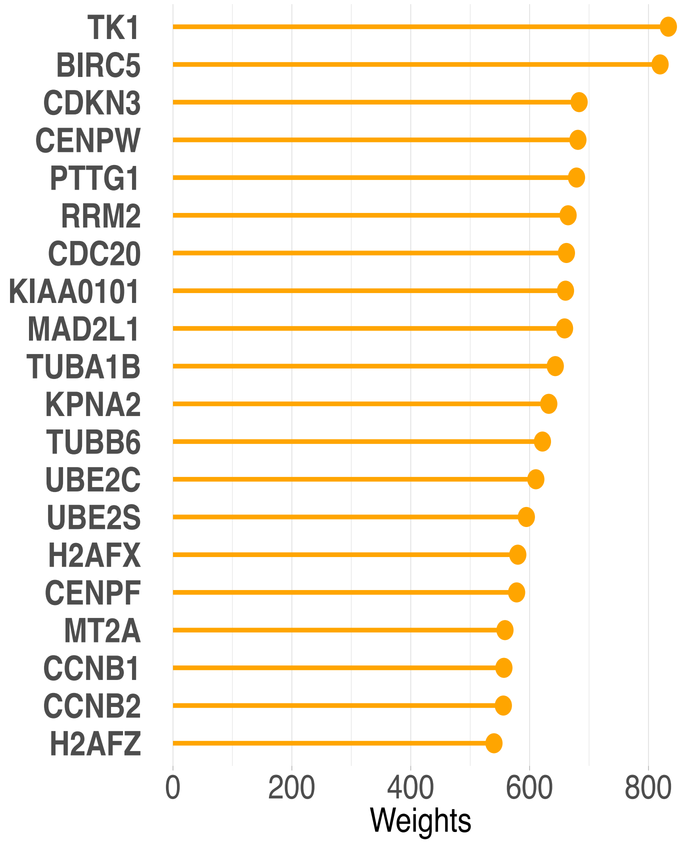

Epi GEM 23

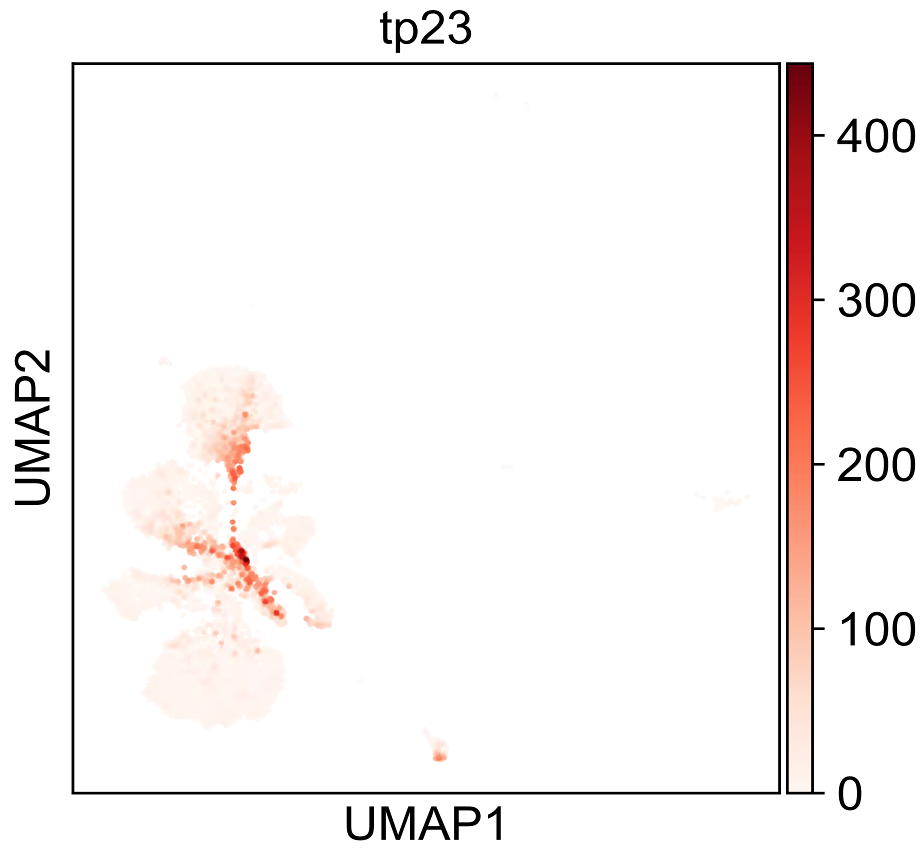

Cell Type

Epi_1

Top 20 genes

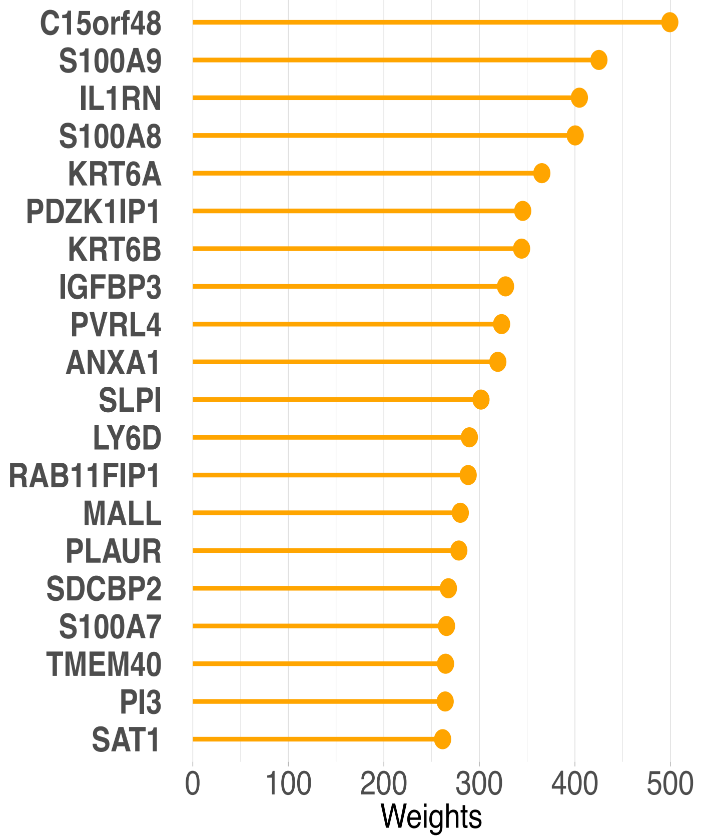

Epi GEM 24

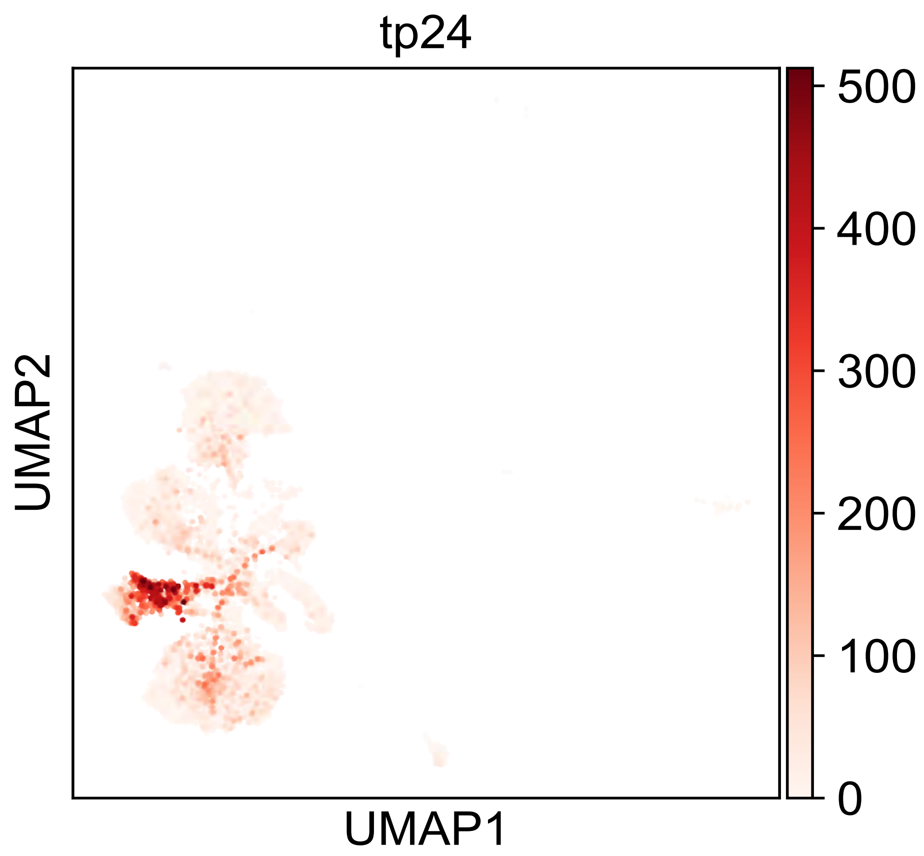

Cell Type

Epi_2

Epi_3

Top 20 genes

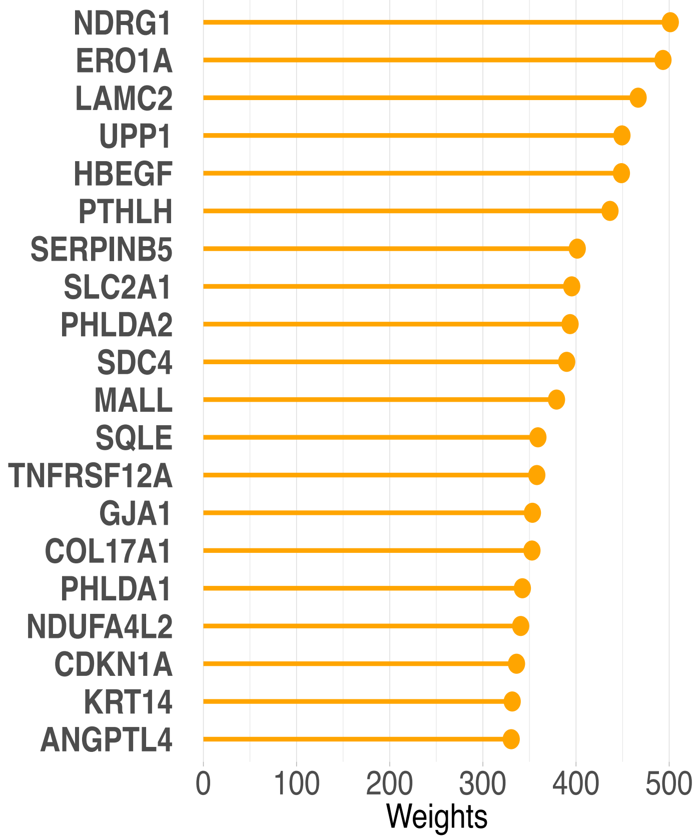

Epi GEM 25

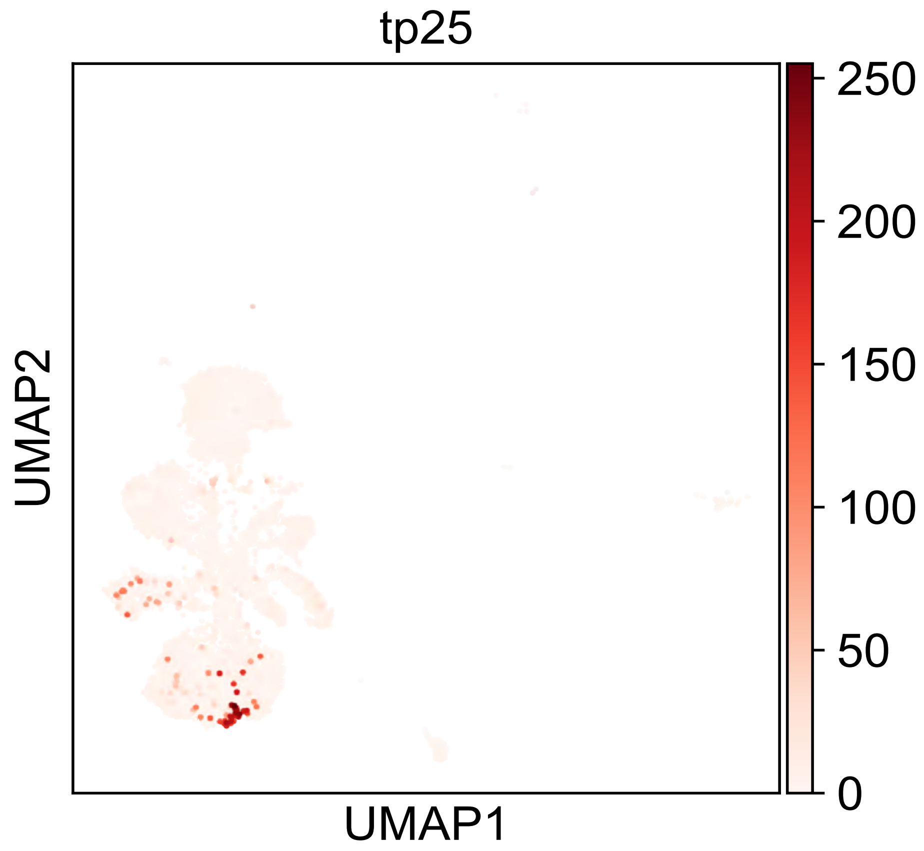

Cell Type

Epi_4

Top 20 genes

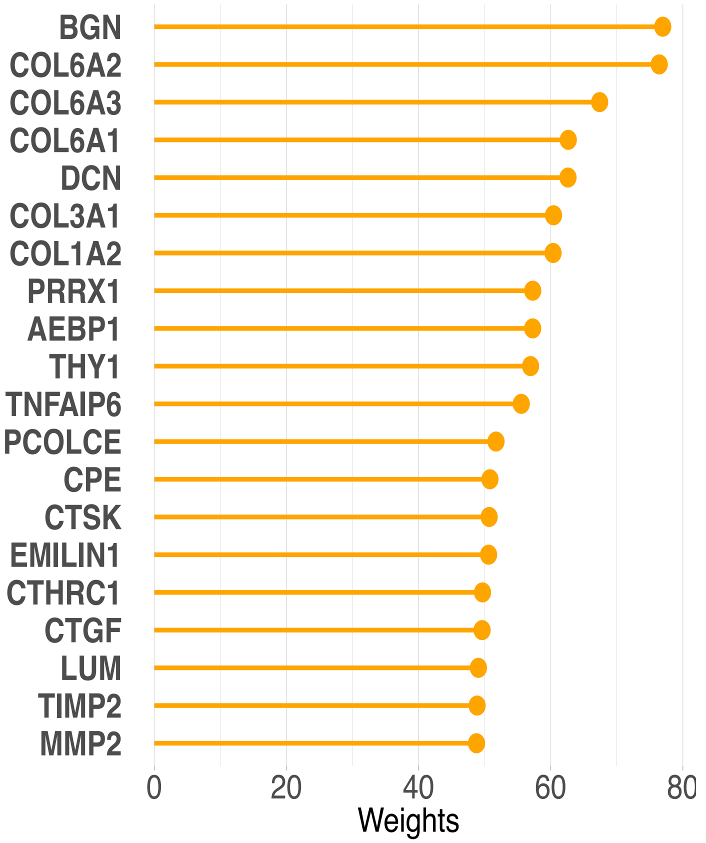

Epi GEM 26

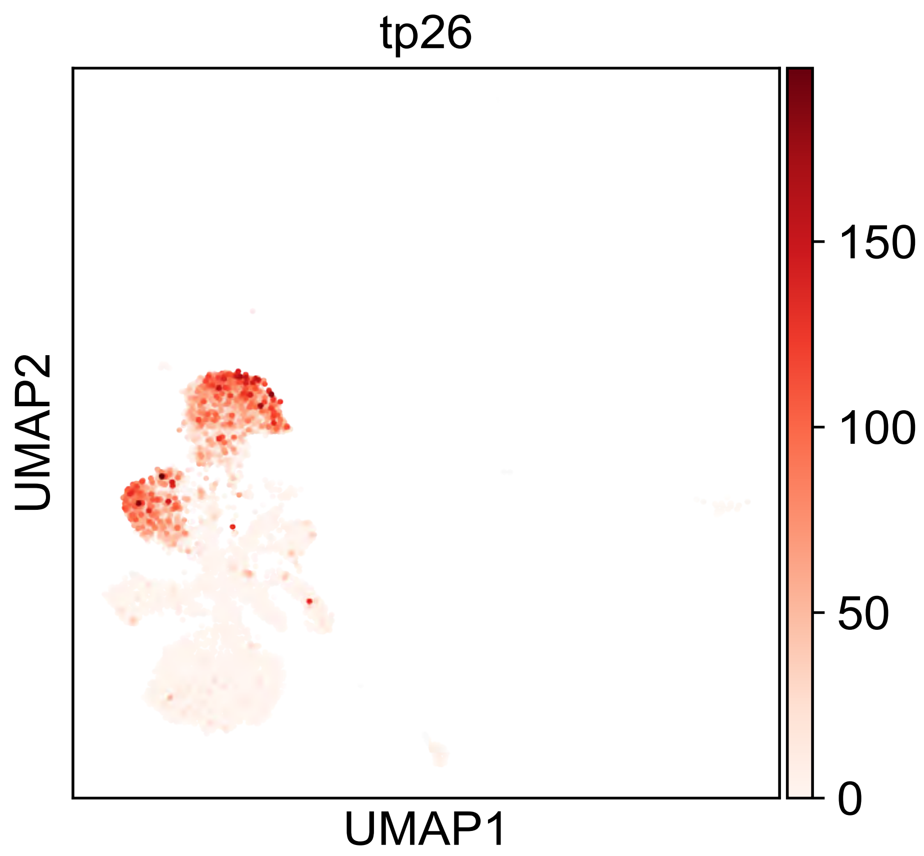

Cell Type

Epi_6

Epi_7

Epi_8

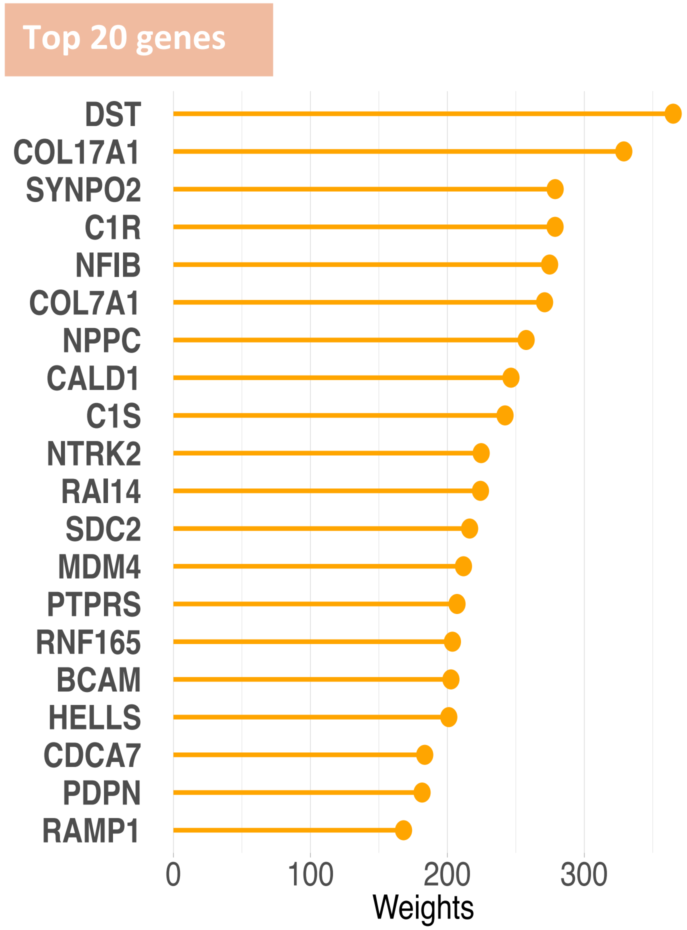

Epi GEM 30

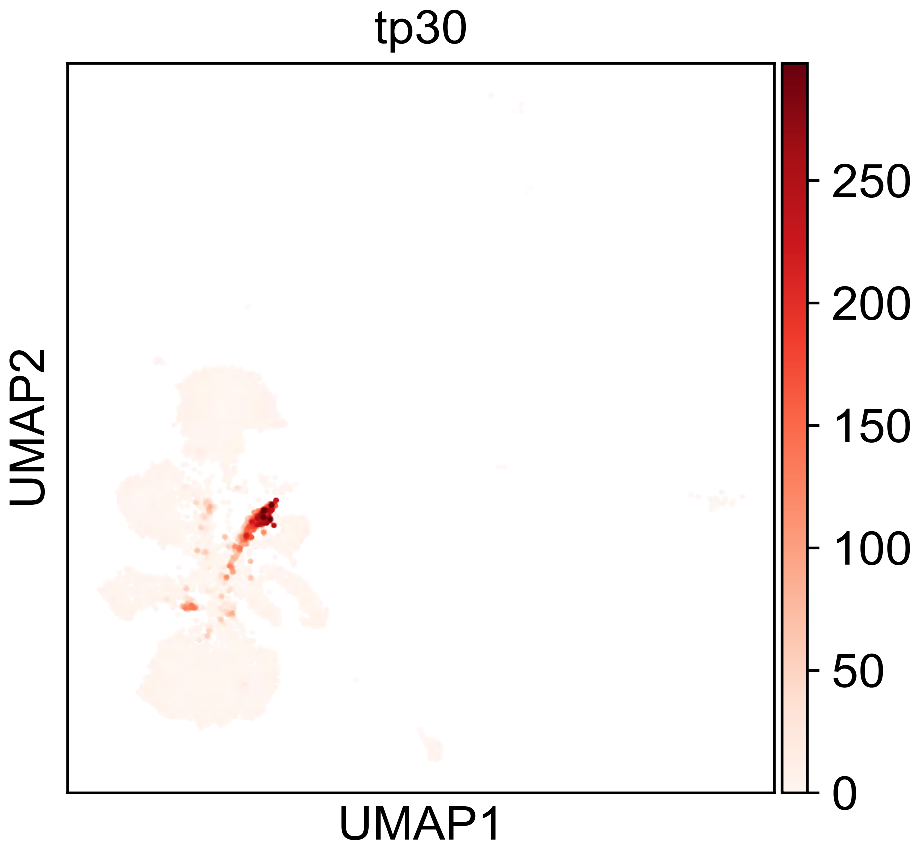

Cell Type

Epi_1

Top 20 genes

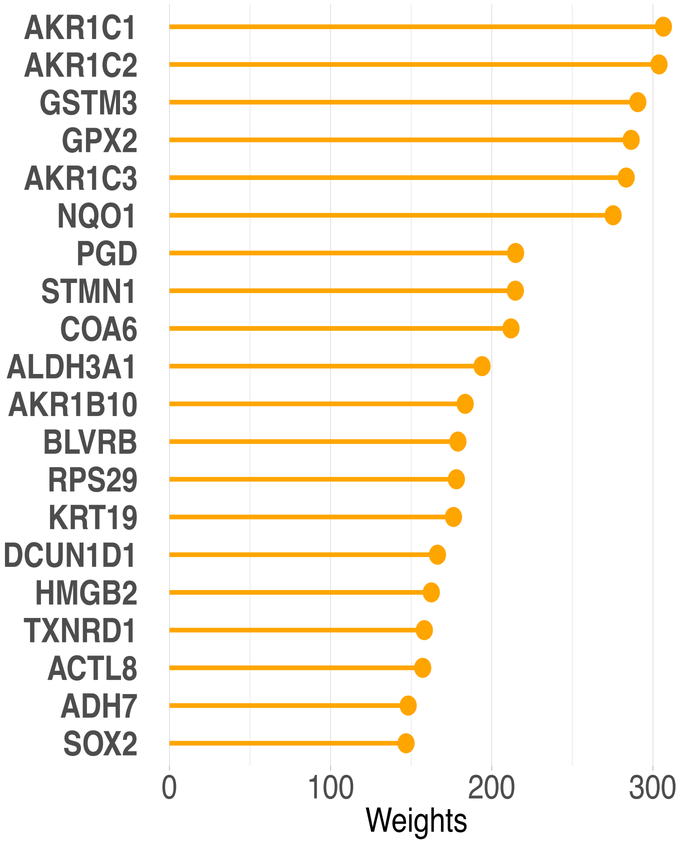

Epi GEM 31

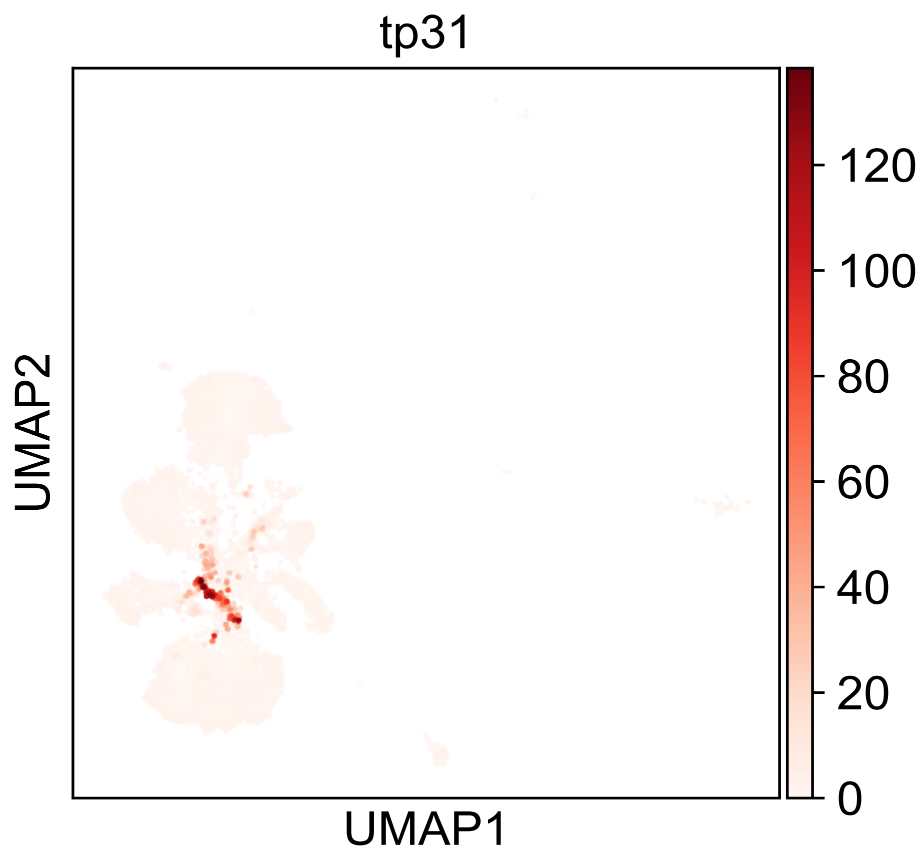

Cell Type

Epi_1

Top 20 genes

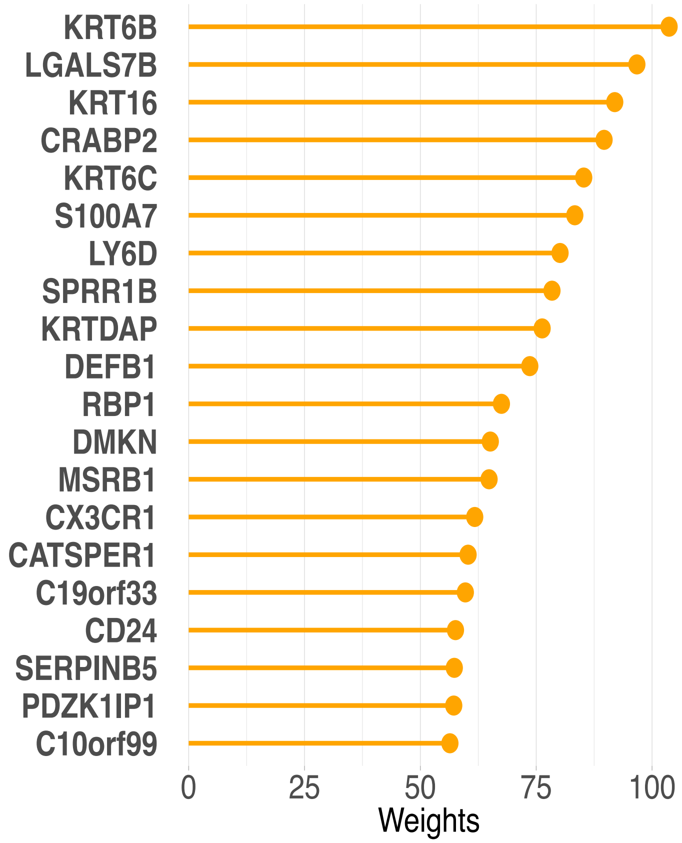

Epi GEM 32

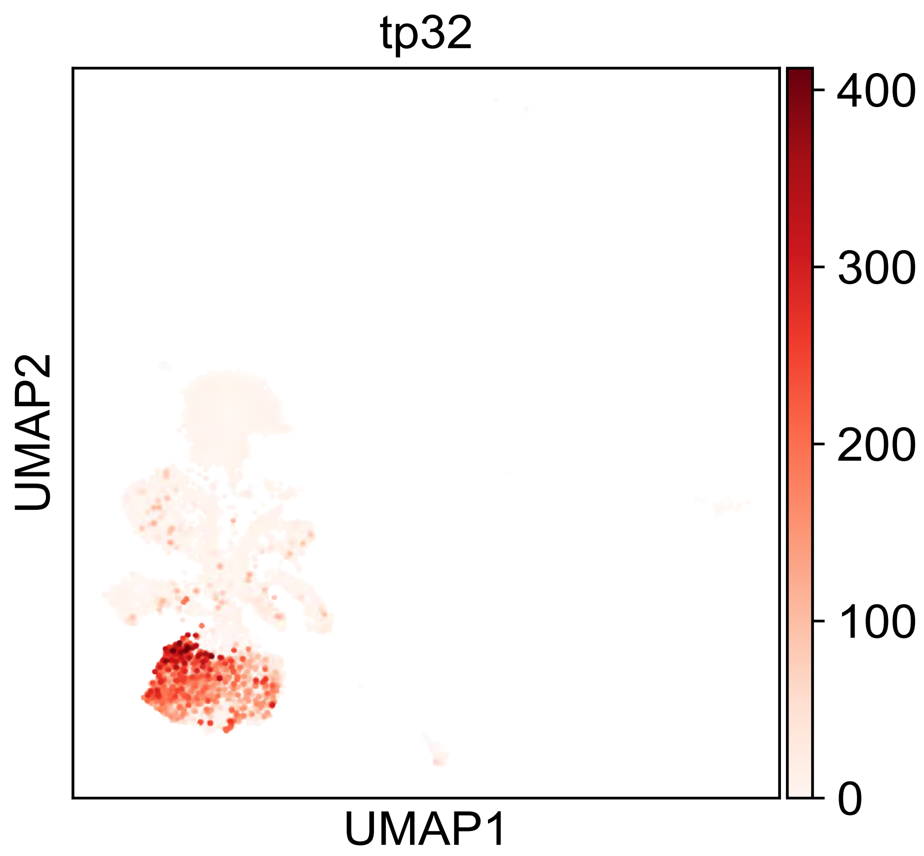

Cell Type

Epi_4

Epi_5

Top 20 genes

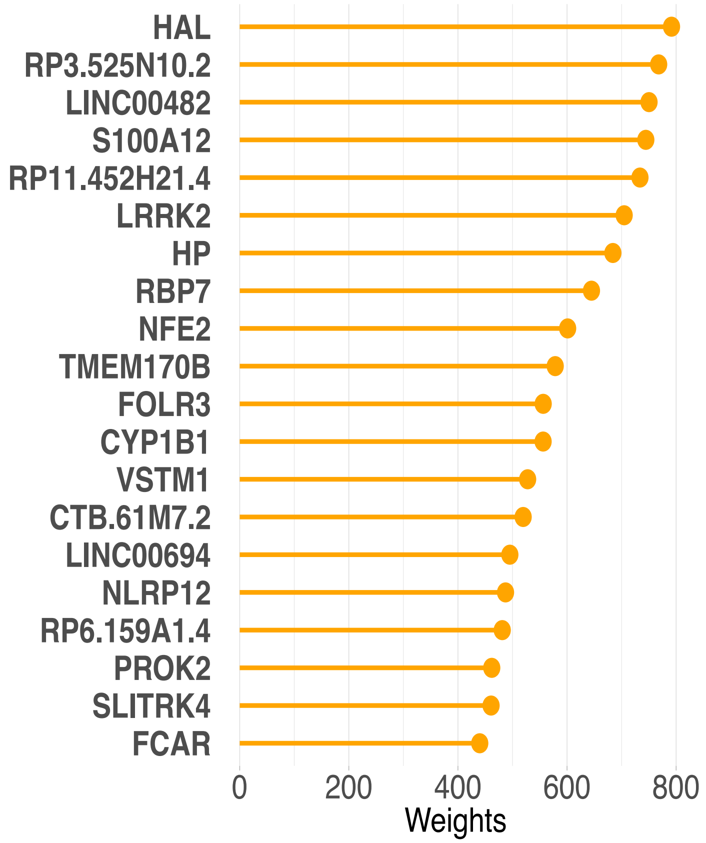

Epi GEM 35

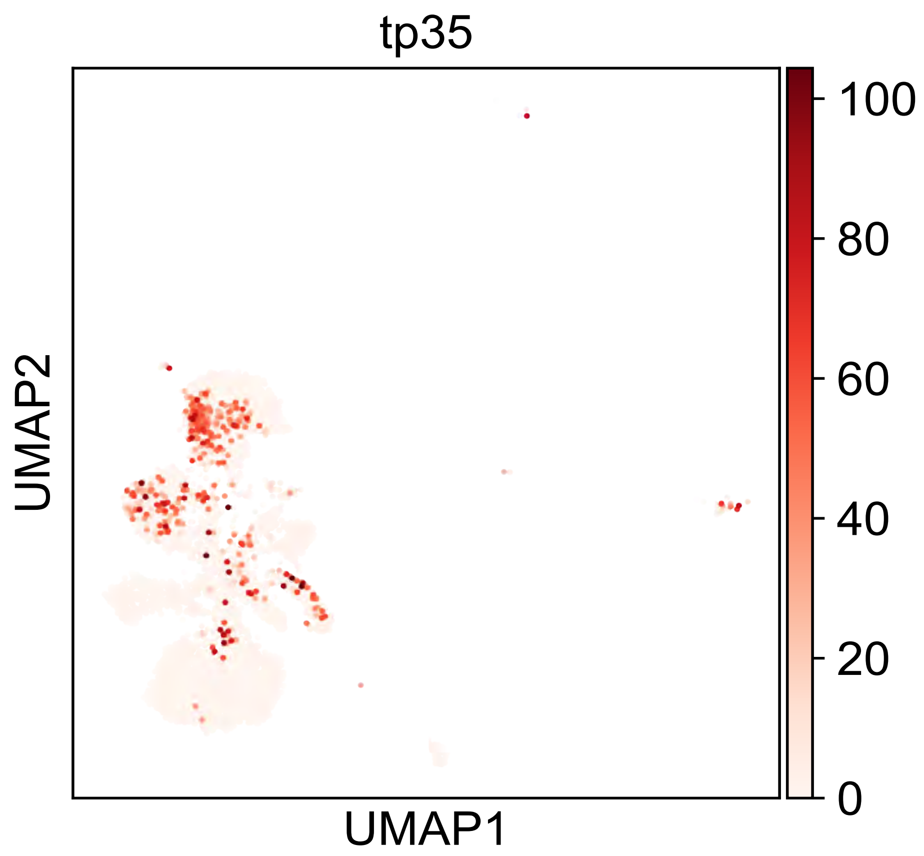

Cell Type

Epi_7

Epi_3

Epi_6

Top 20 genes

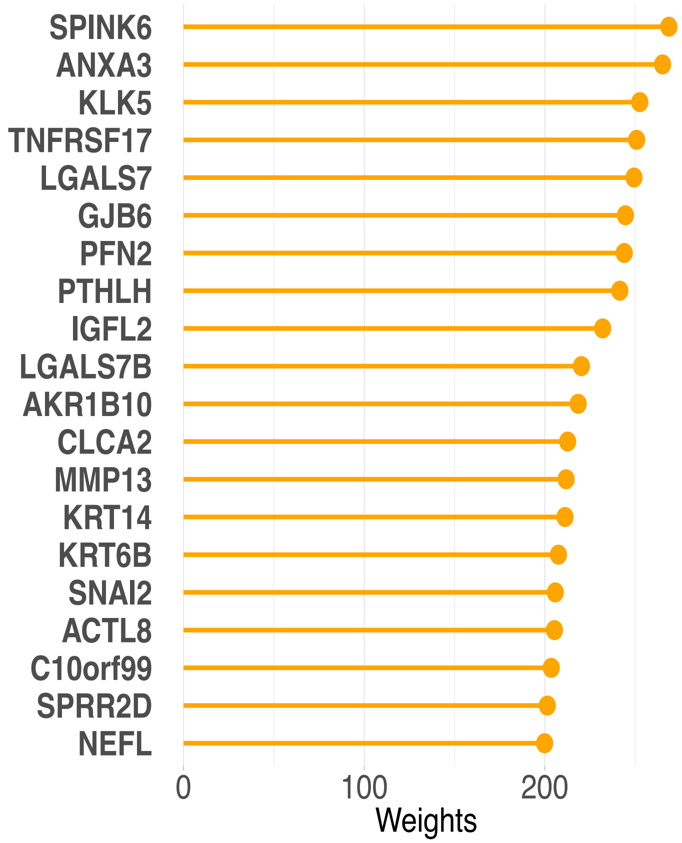

Epi GEM 38

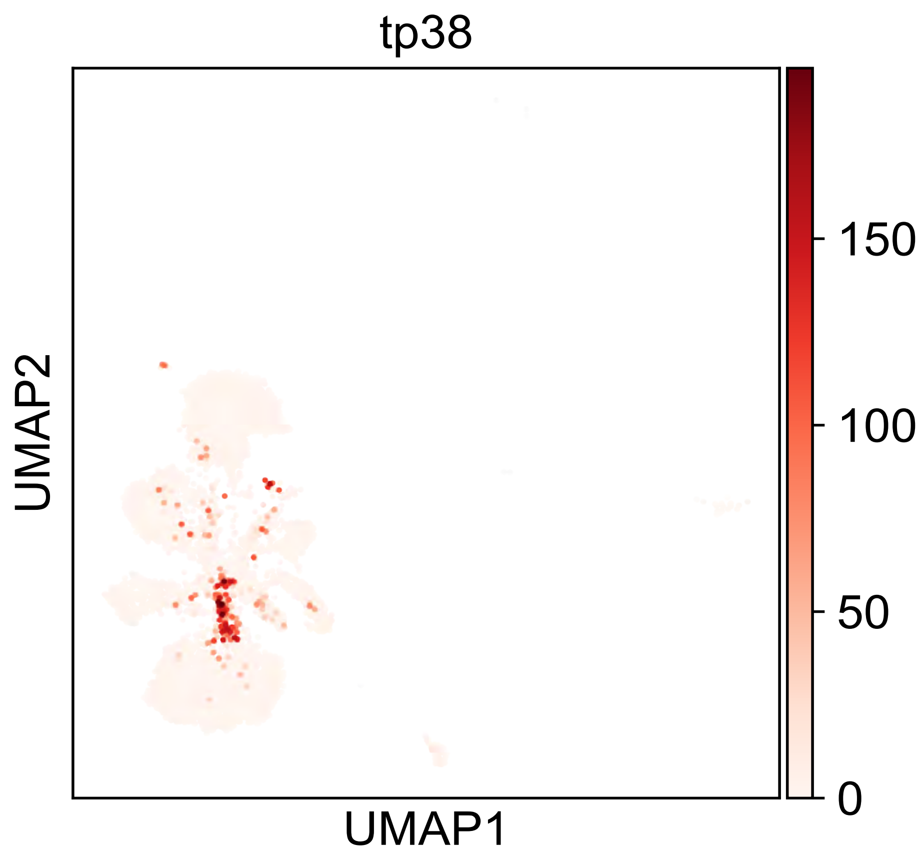

Cell Type

Epi_1

Epi_3

Top 20 genes

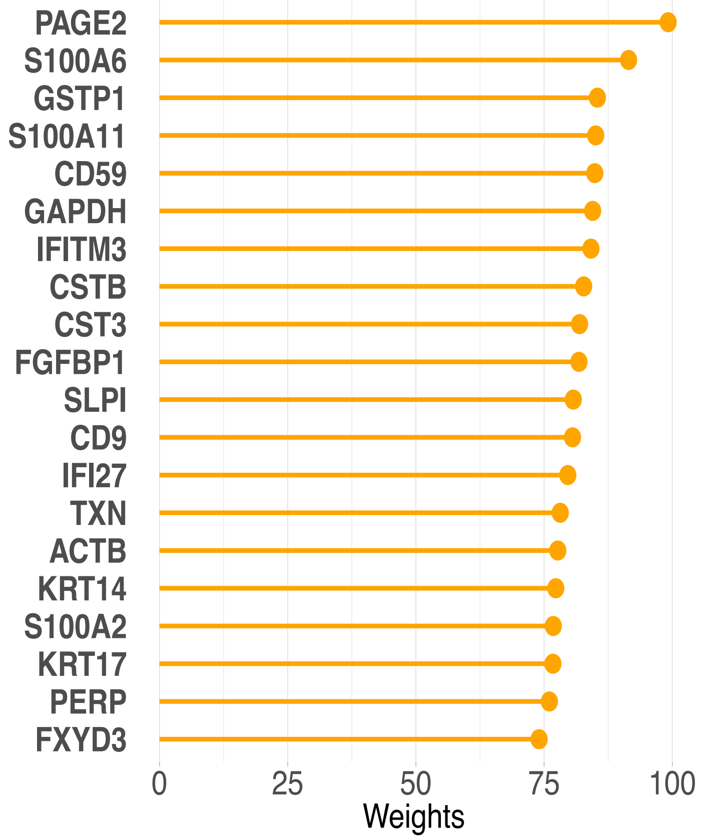

Epi GEM 39

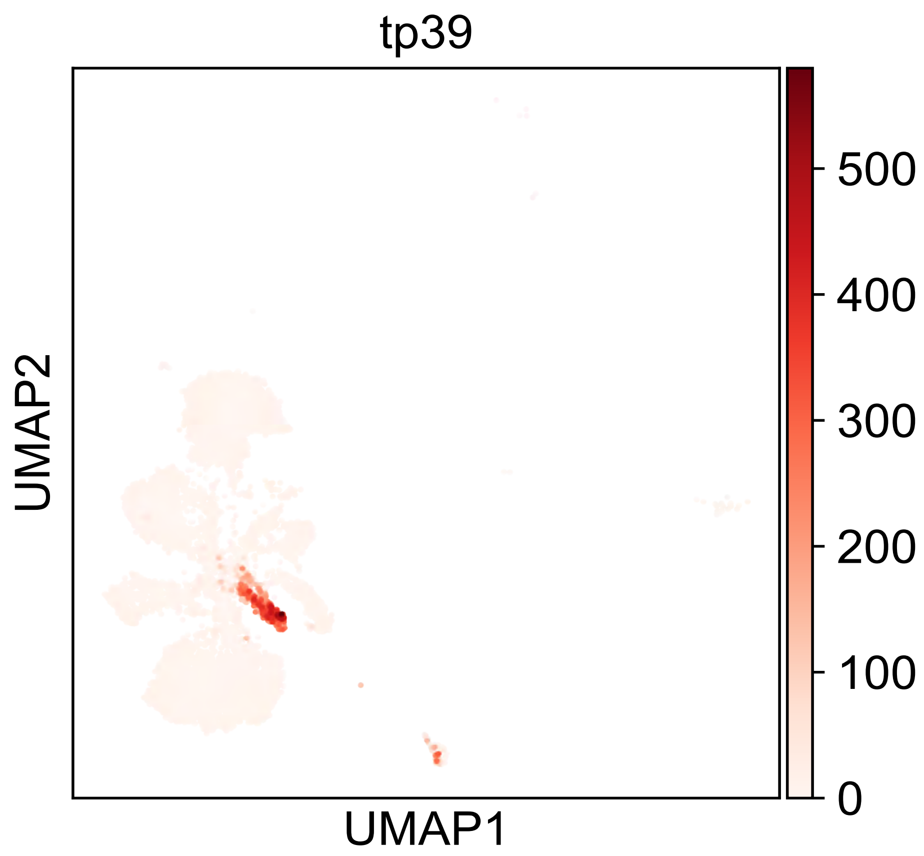

Cell Type

Epi_1

Top 20 genes

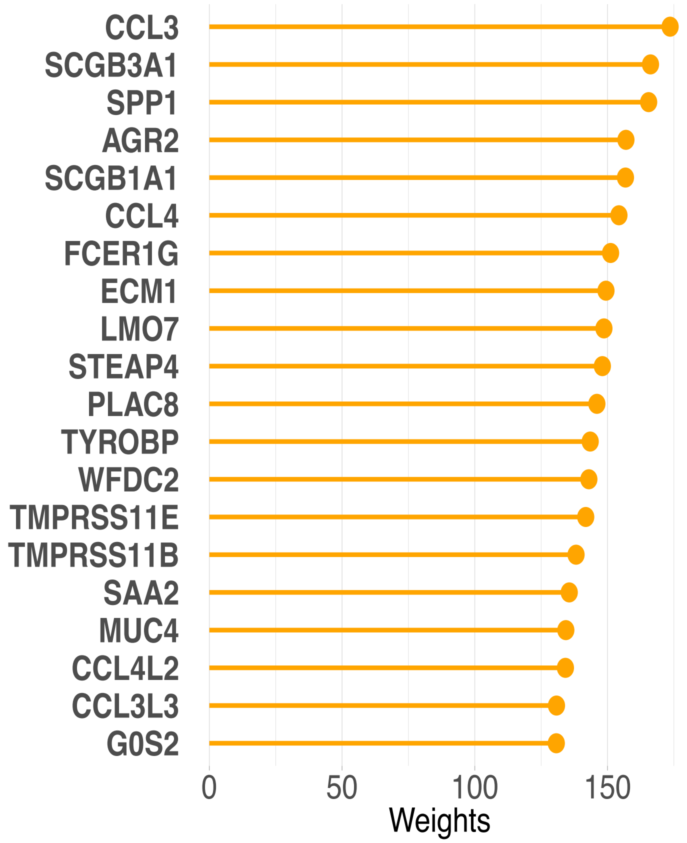

Epi GEM 41

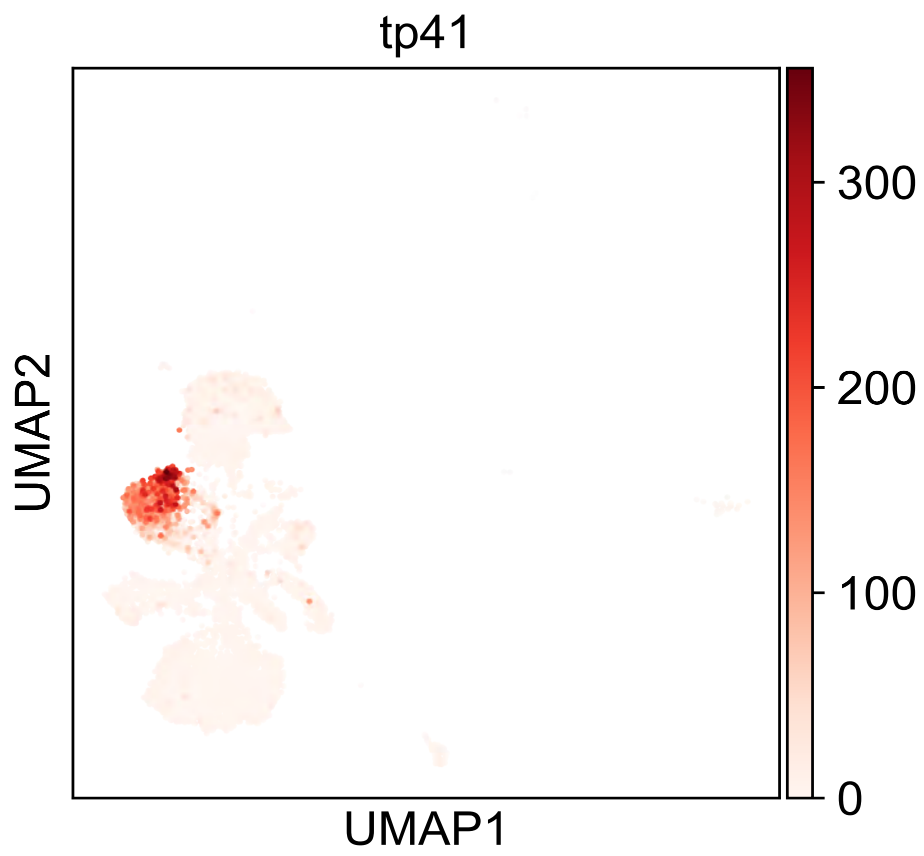

Cell Type

Epi_6

Top 20 genes

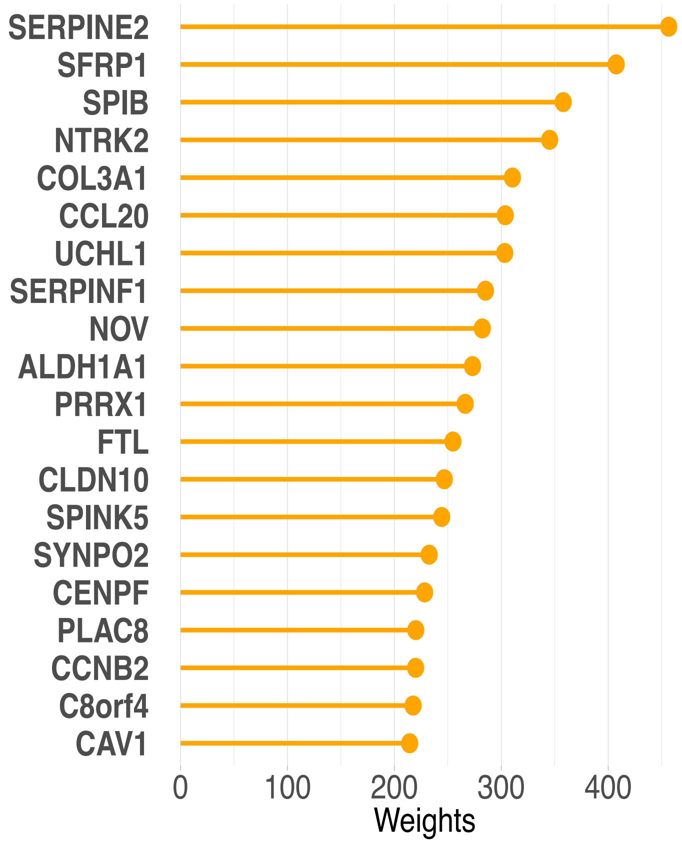

Epi GEM 42

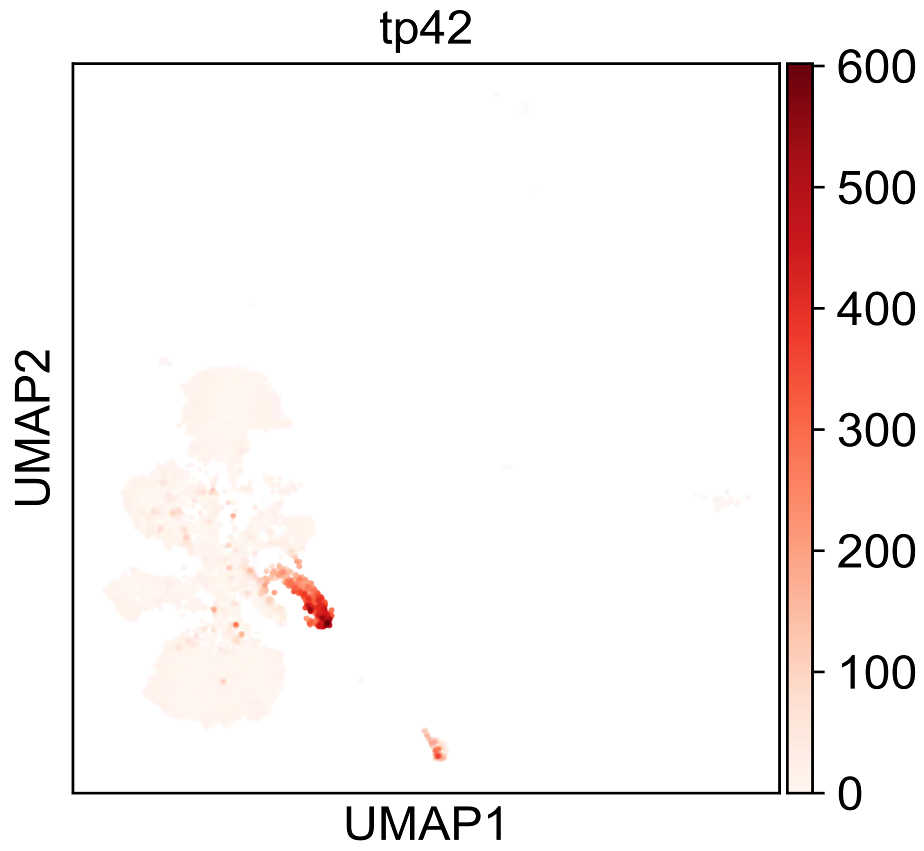

Cell Type

Epi_3

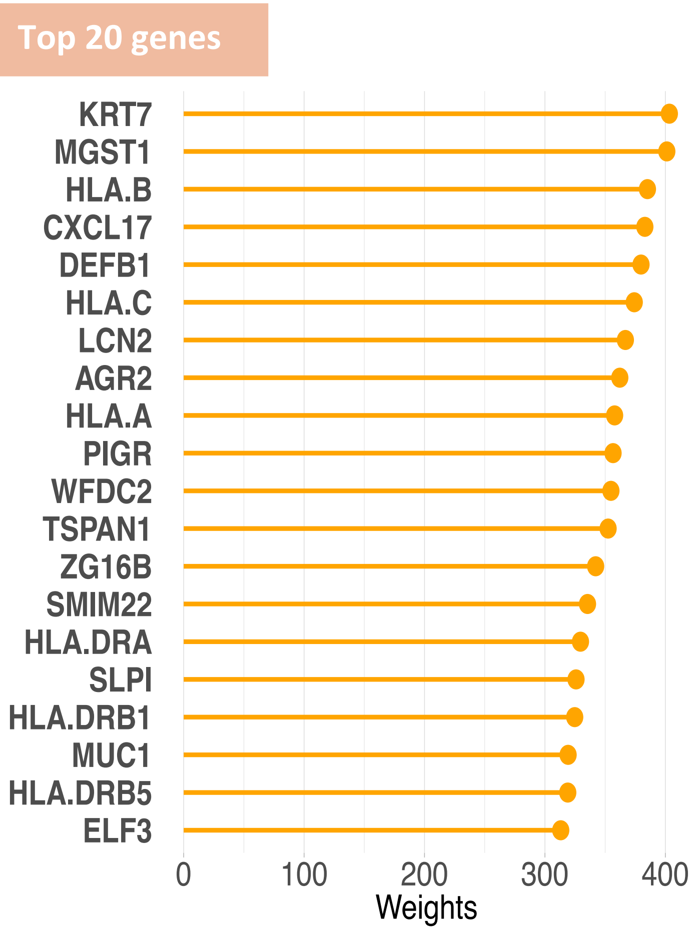

Epi GEM 43

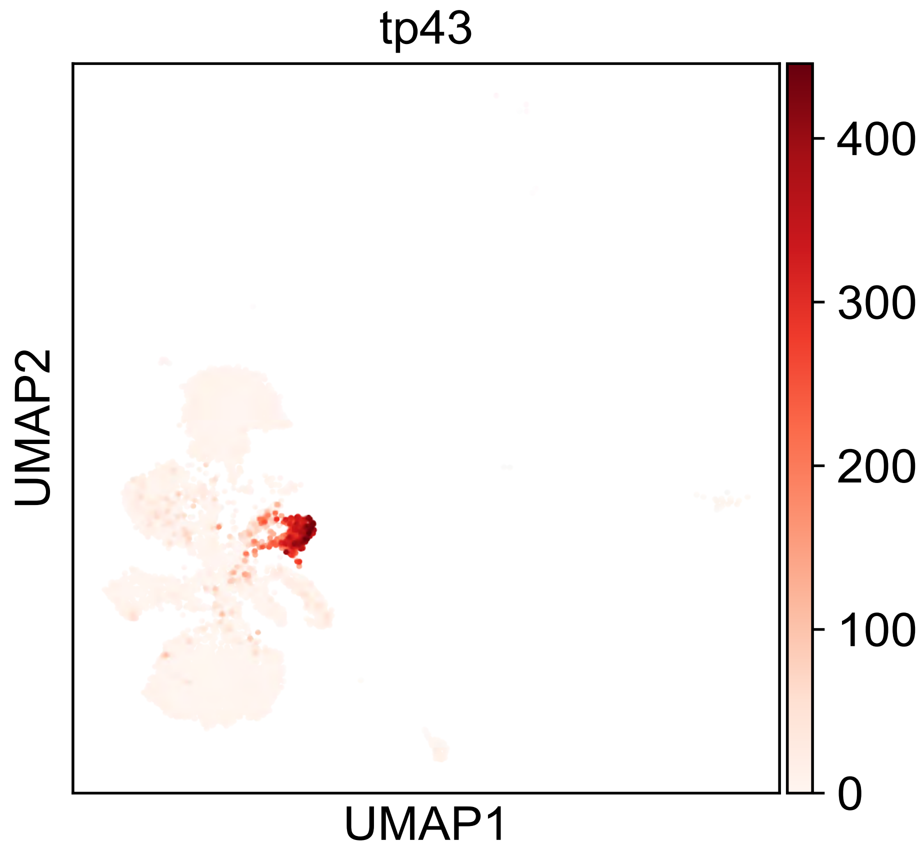

Cell Type

Epi_3

Epi_2

Top 20 genes

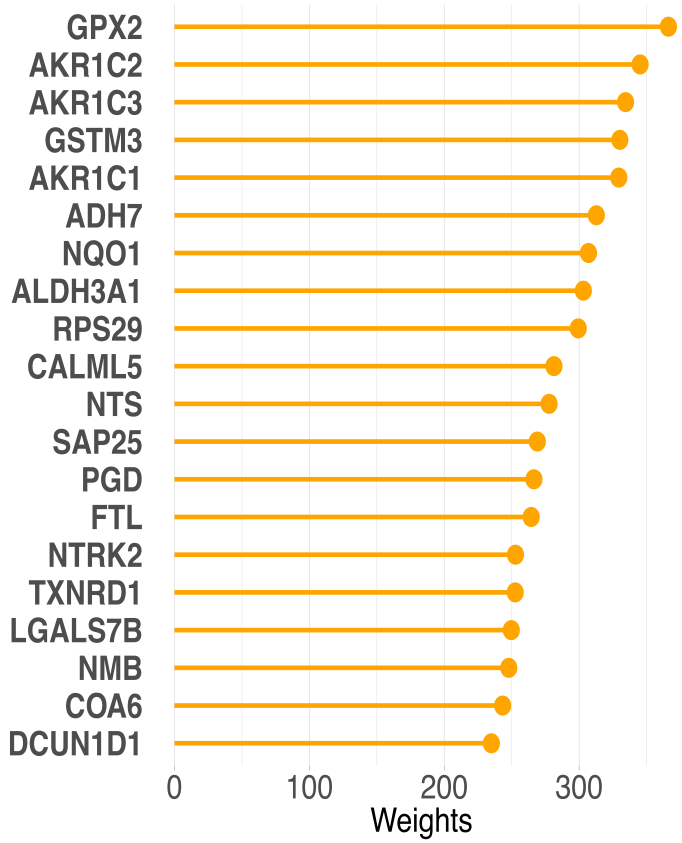

Epi GEM 45

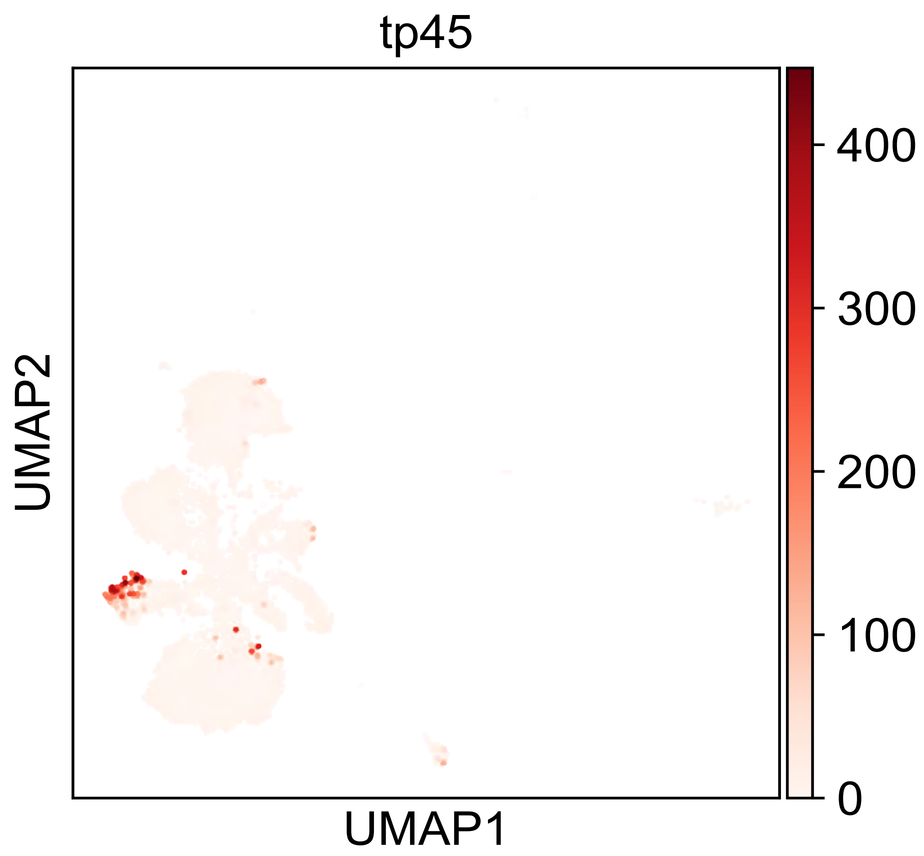

Cell Type

Epi_2

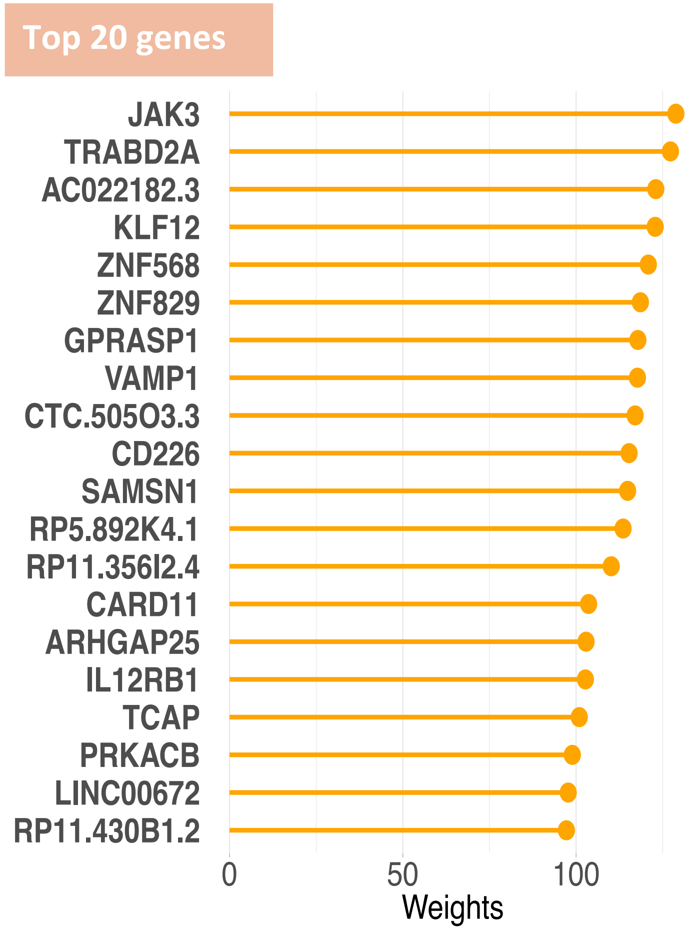

Epi GEM 47

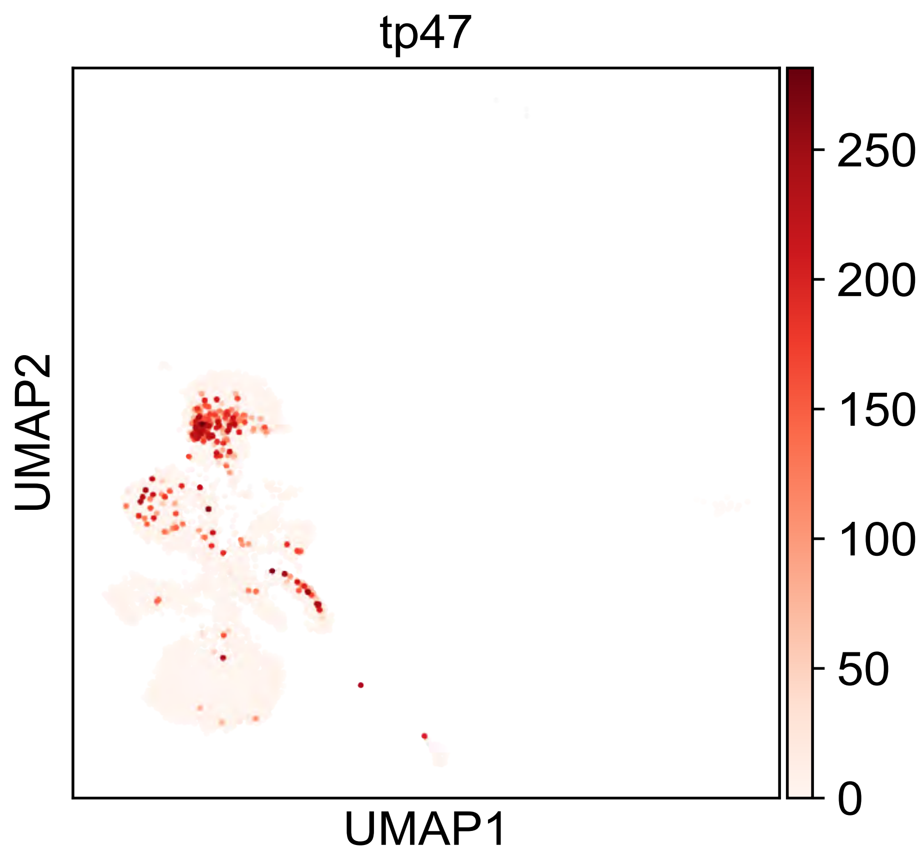

Cell Type

Epi_7

Epi_3

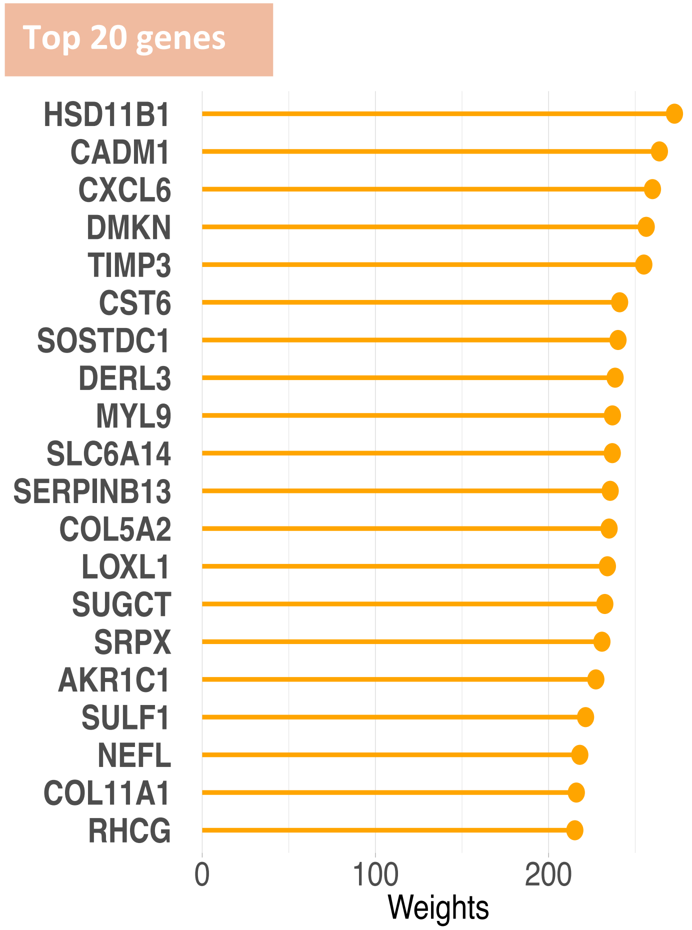

Epi GEM 48

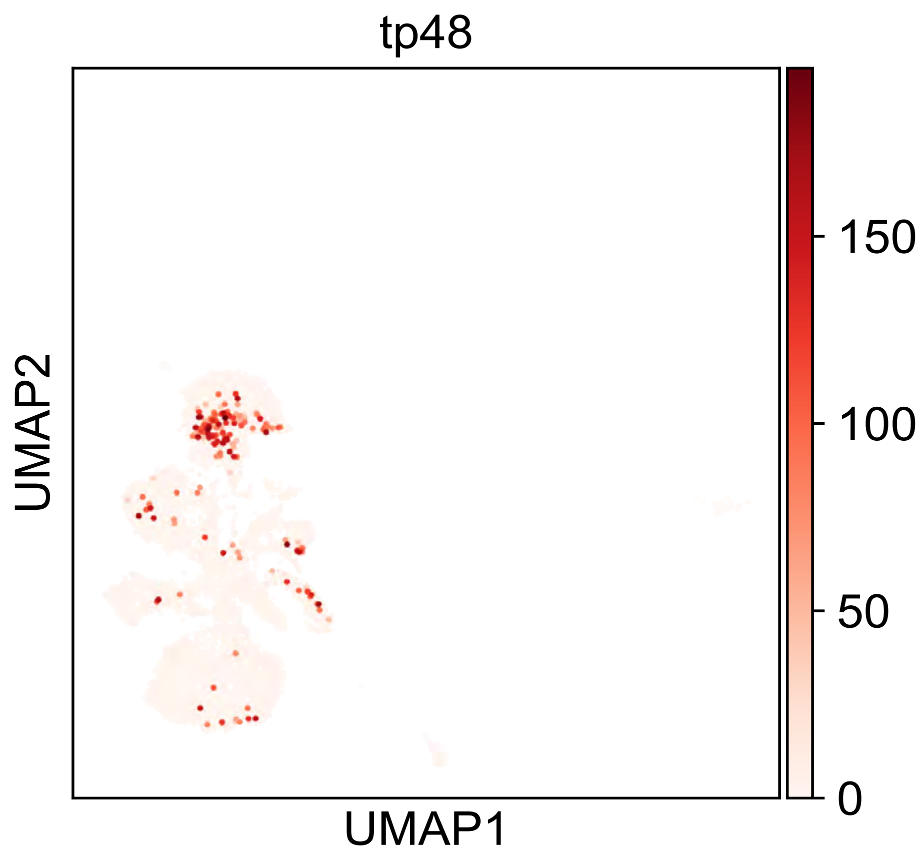

Cell Type

Epi_7

Epi_3

Top 20 genes

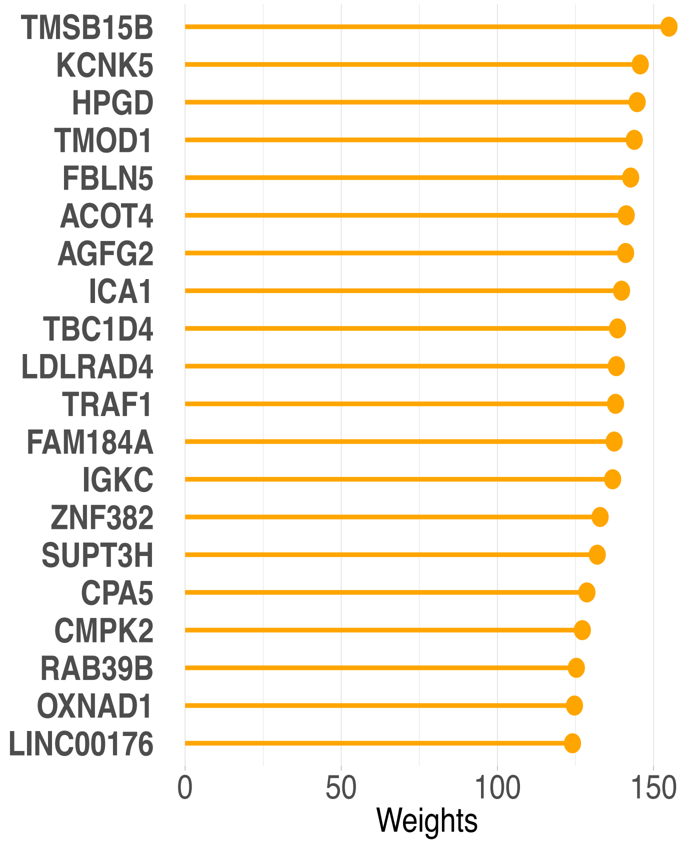

Epi GEM 49

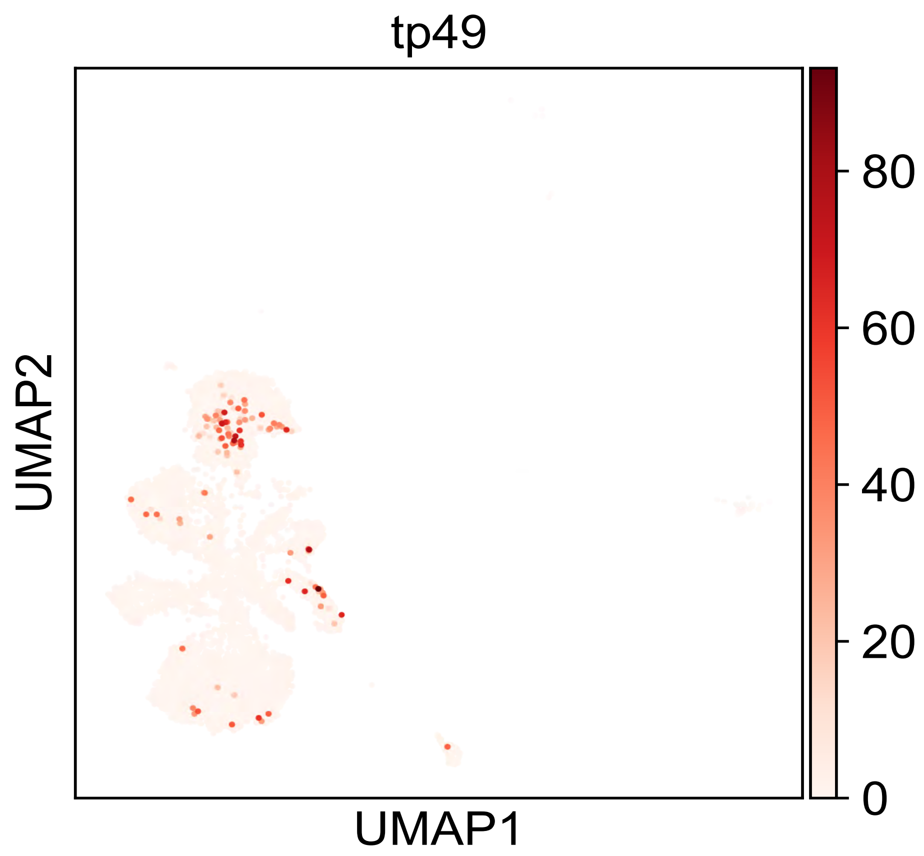

Cell Type

Epi_7

Epi_3

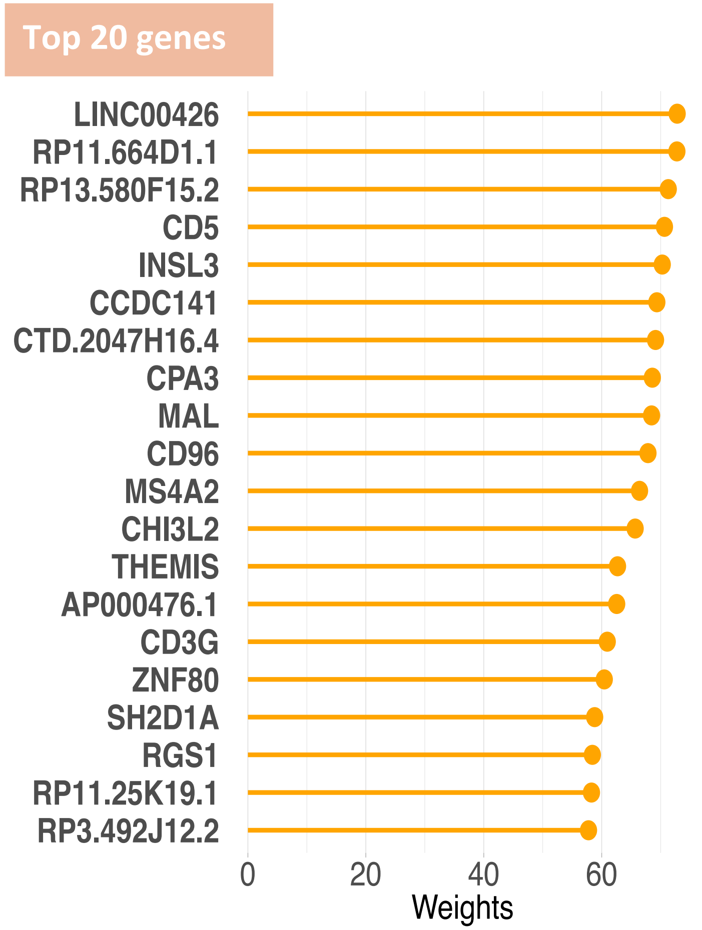

Epi GEM 50

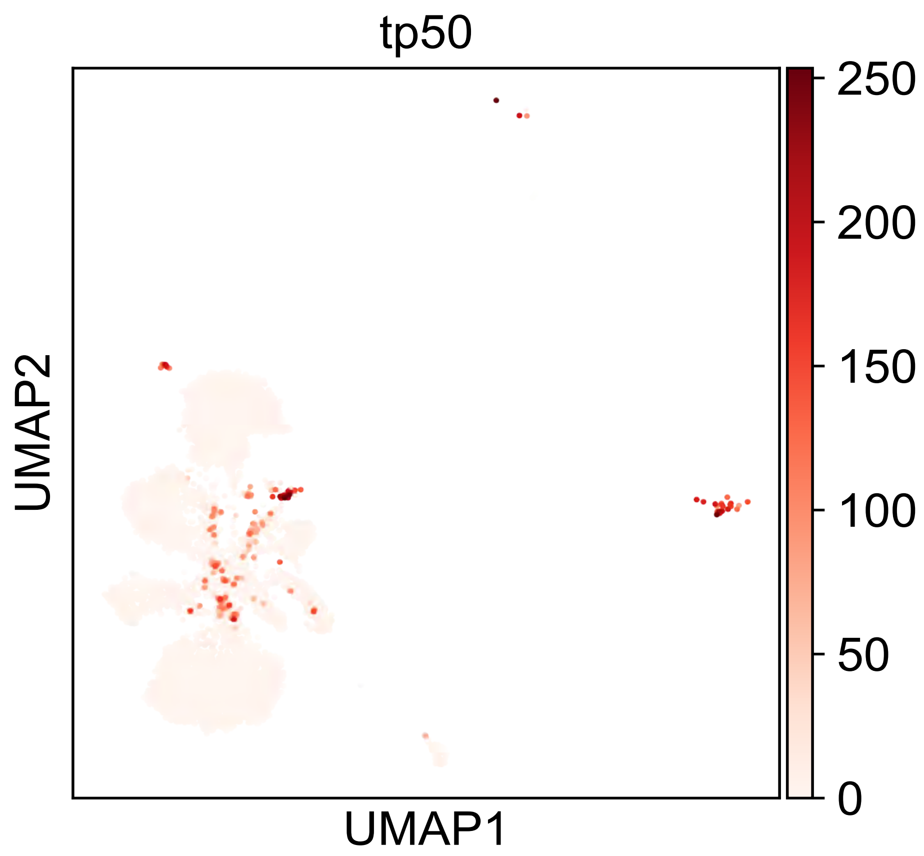

Cell Type

Epi_1

Top 20 genes

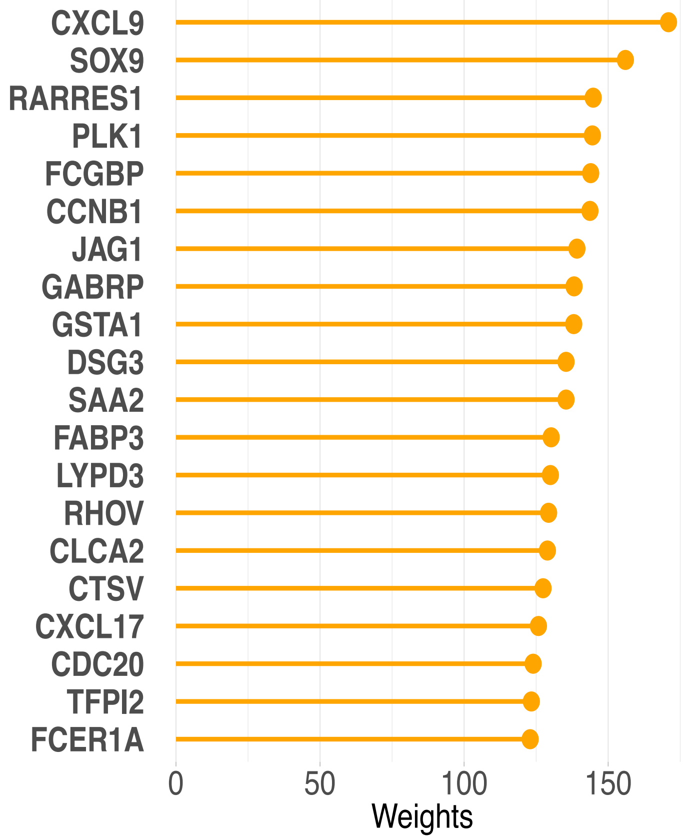

Epi GEM 51

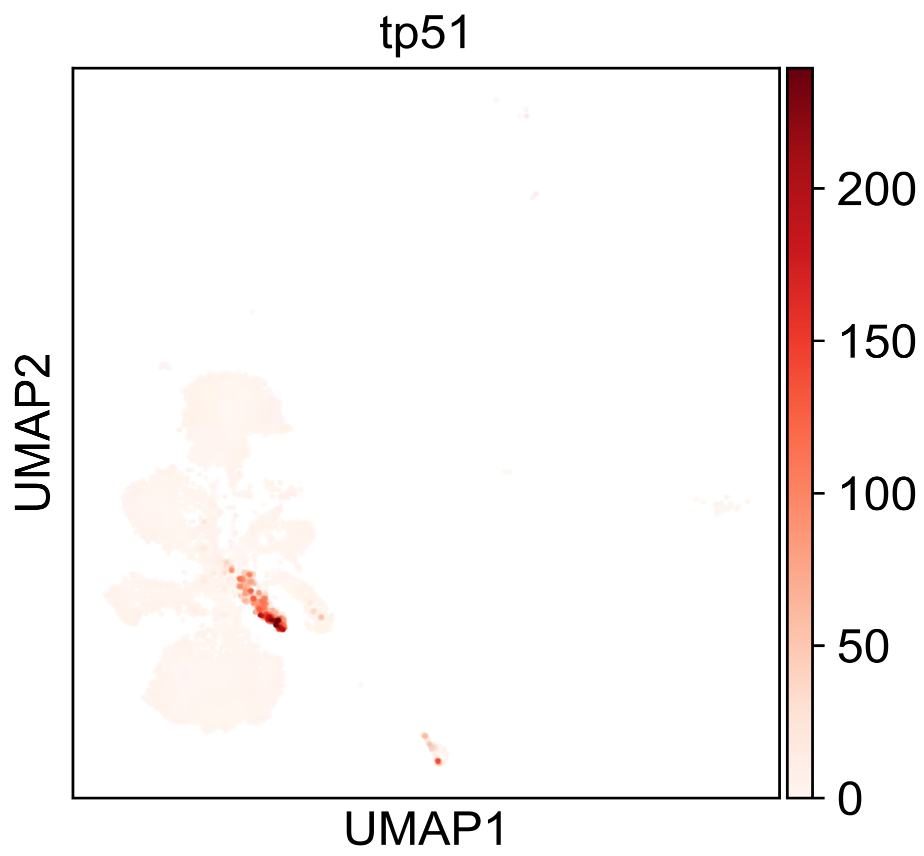

Cell Type

Epi_1

Top 20 genes

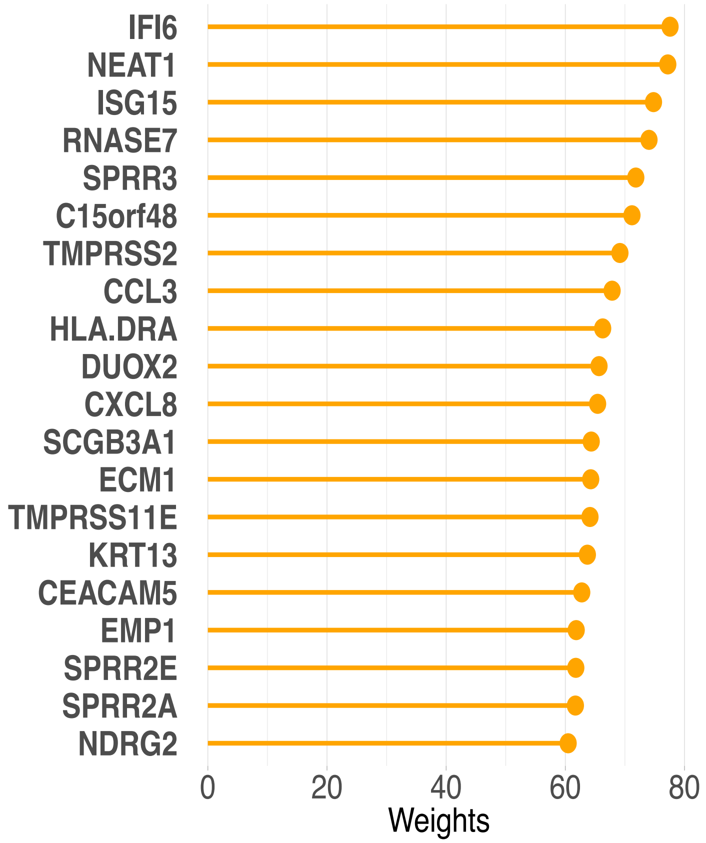

Epi GEM 54

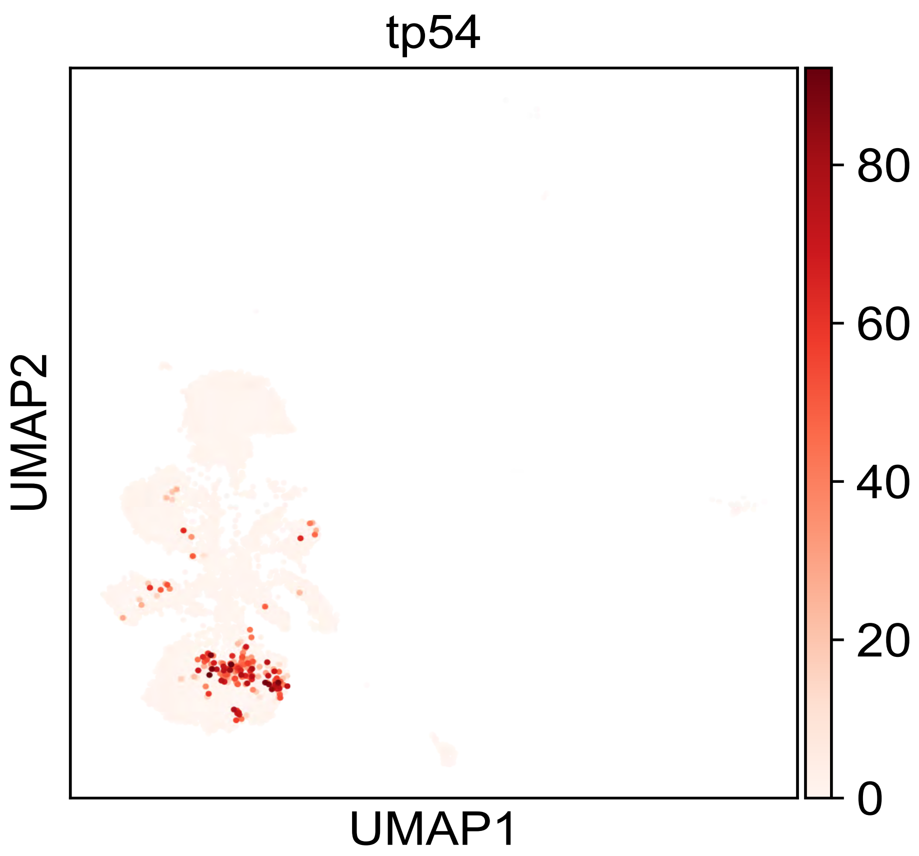

Cell Type

Epi_5

Top 20 genes

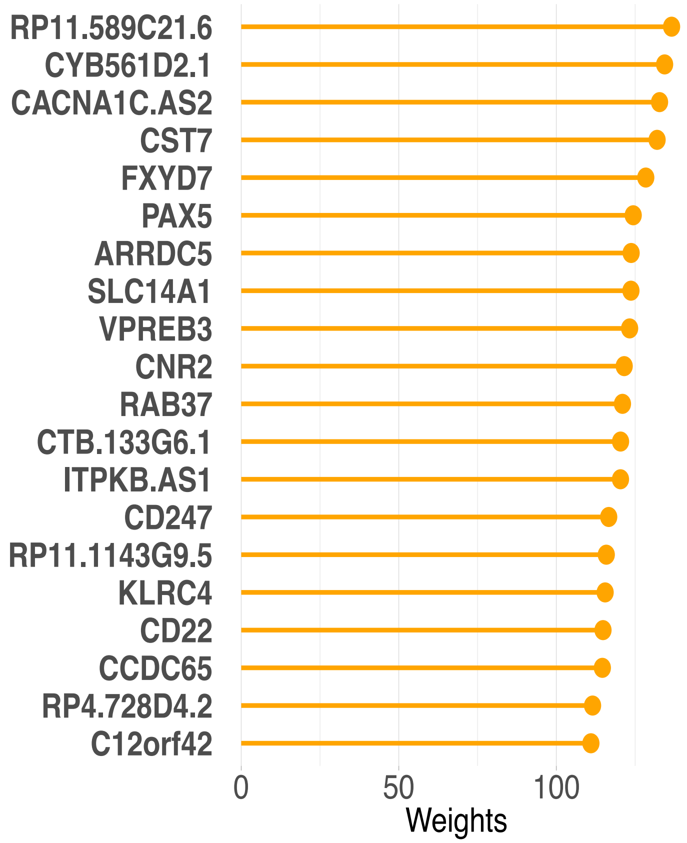

Epi GEM 56

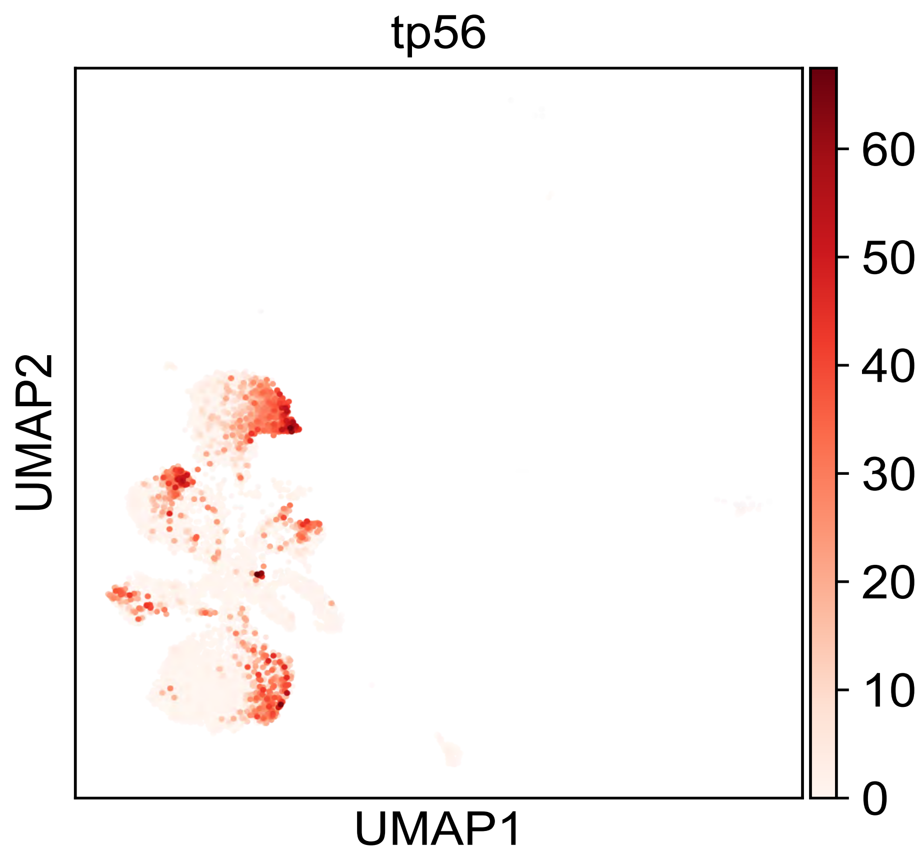

Cell Type

Epi_8

Epi_5

Epi_2

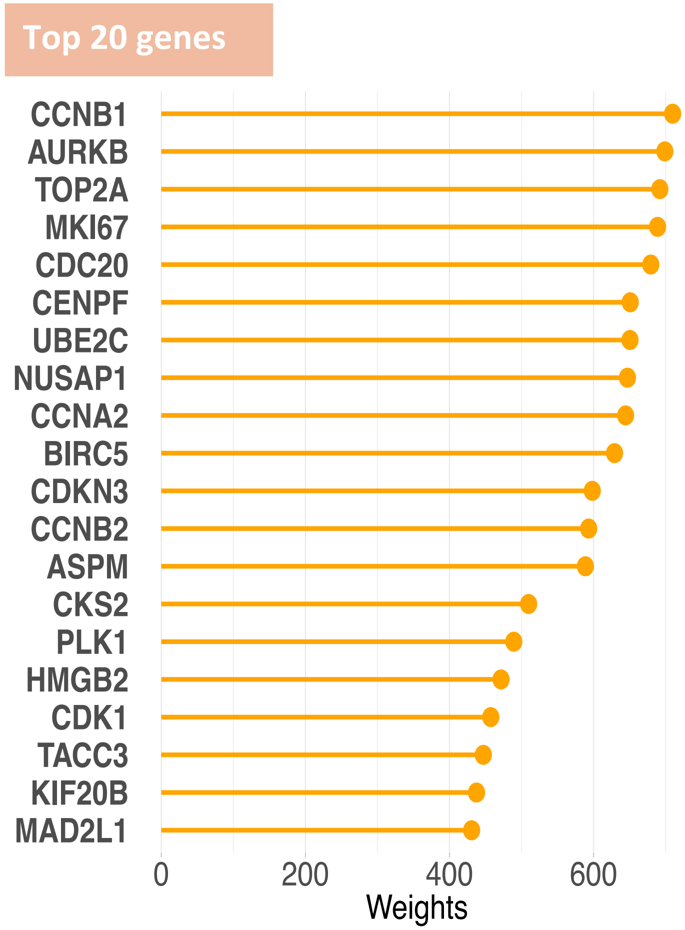

Epi GEM 57

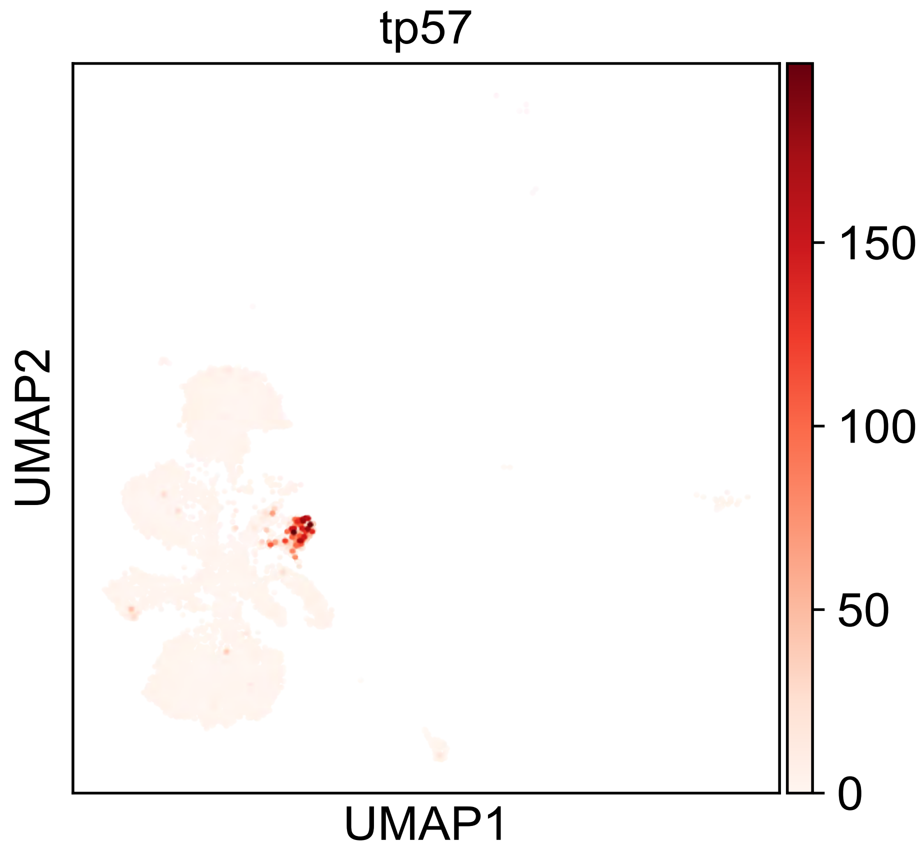

Cell Type

Epi_2

Top 20 genes

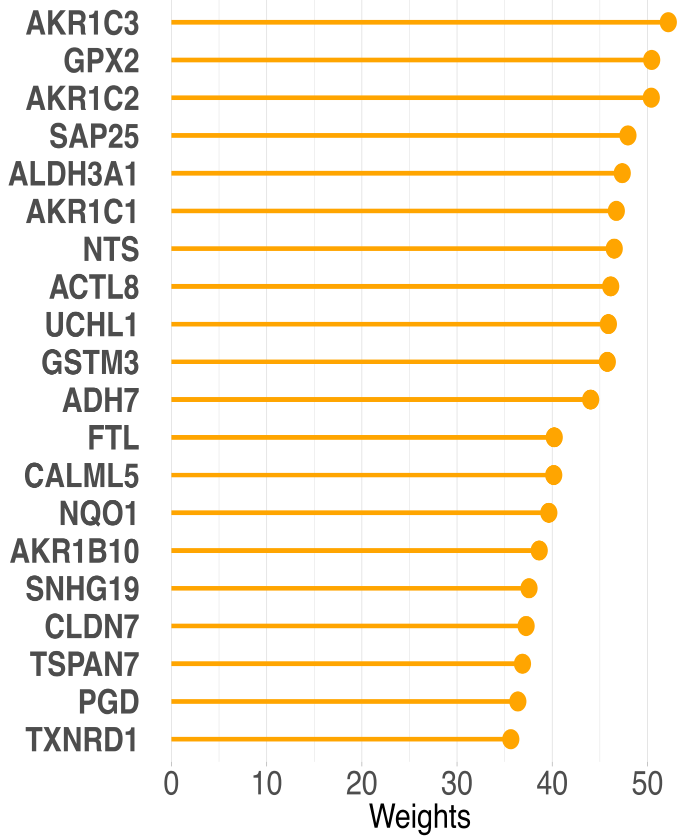

Epi GEM 62

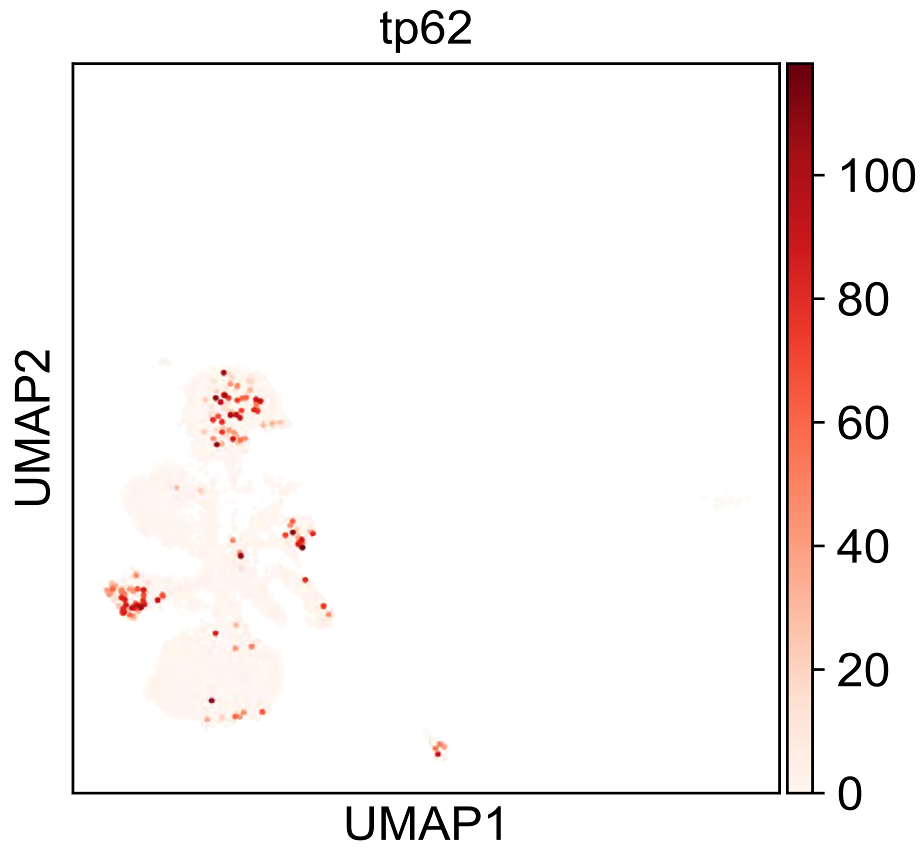

Cell Type

Epi_8

Epi_2

Top 20 genes

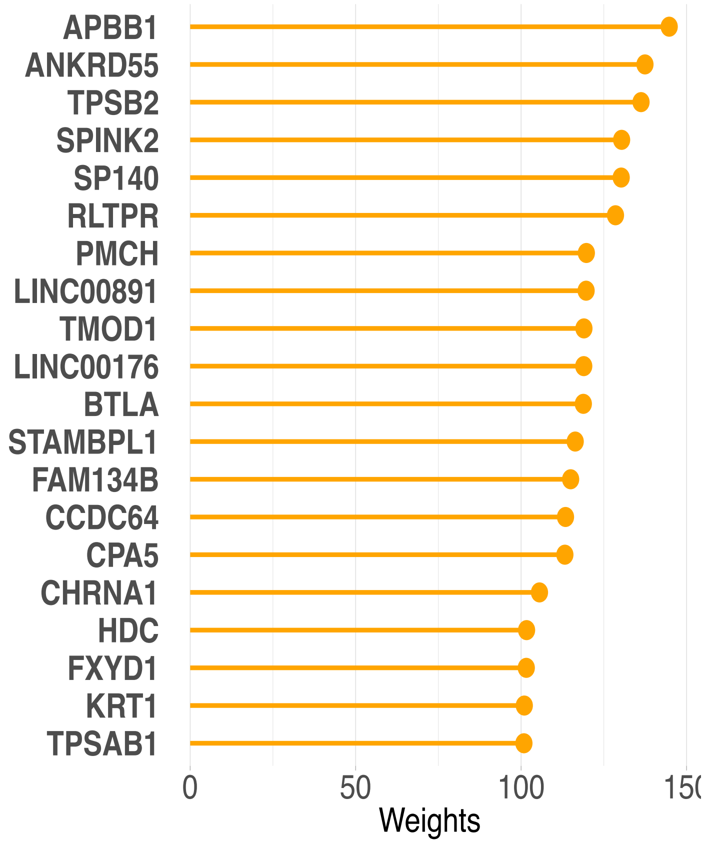

Epi GEM 73

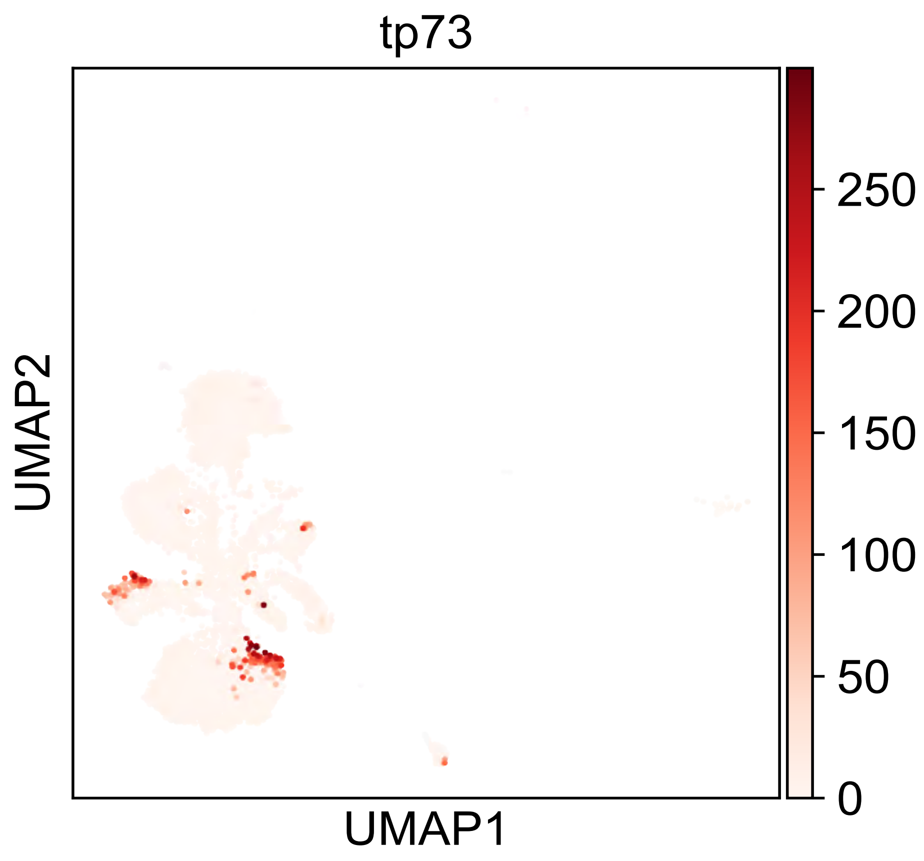

Cell Type

Epi_2

Epi_5

Top 20 genes

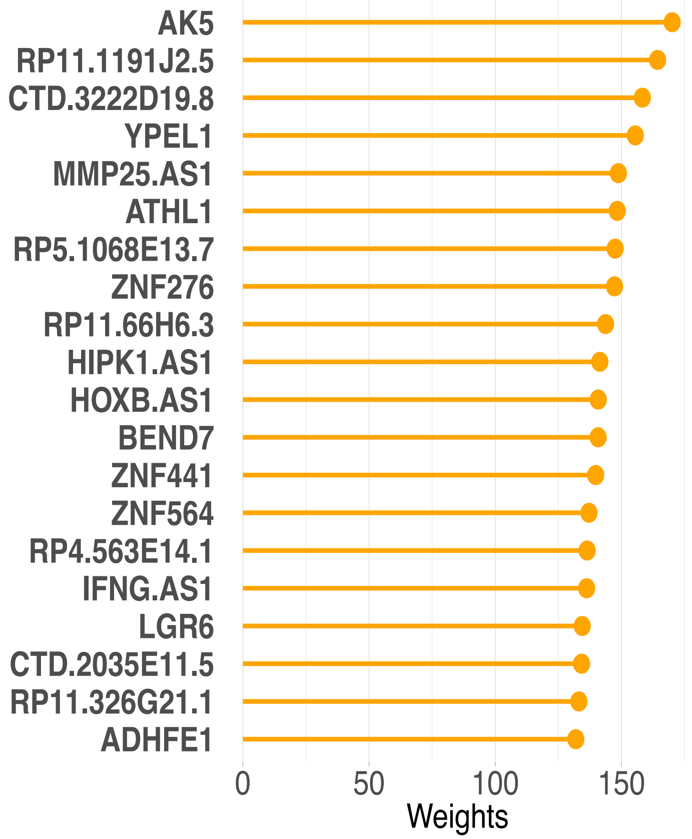

Epi GEM 77

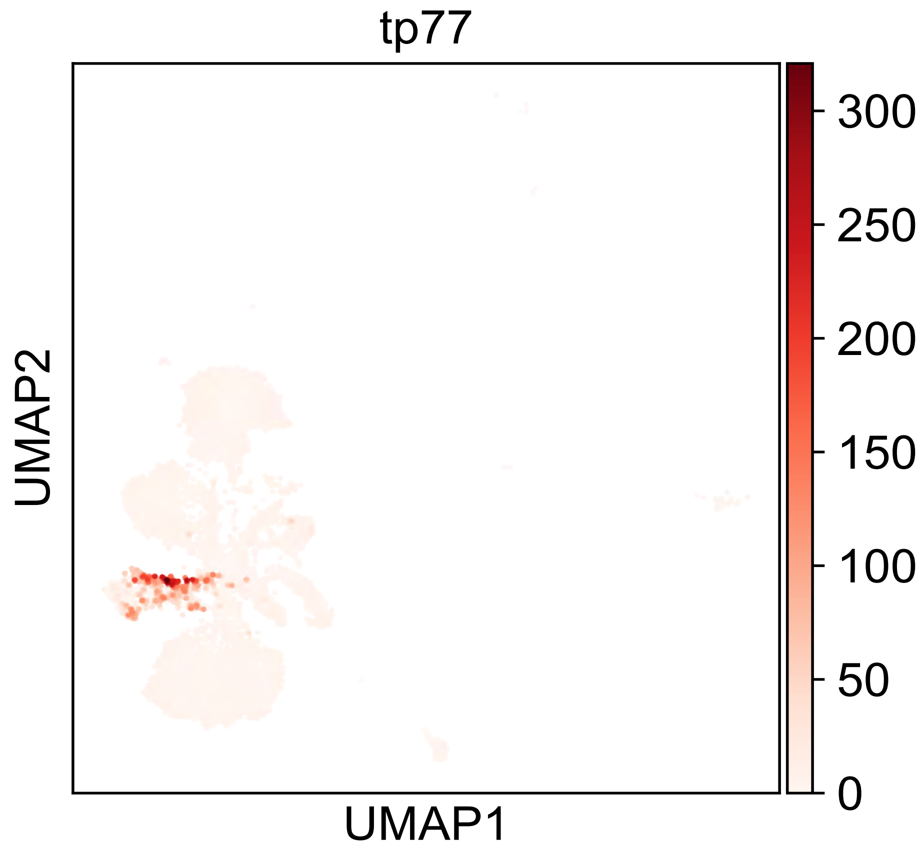

Cell Type

Epi_2

Epi_3

Top 20 genes

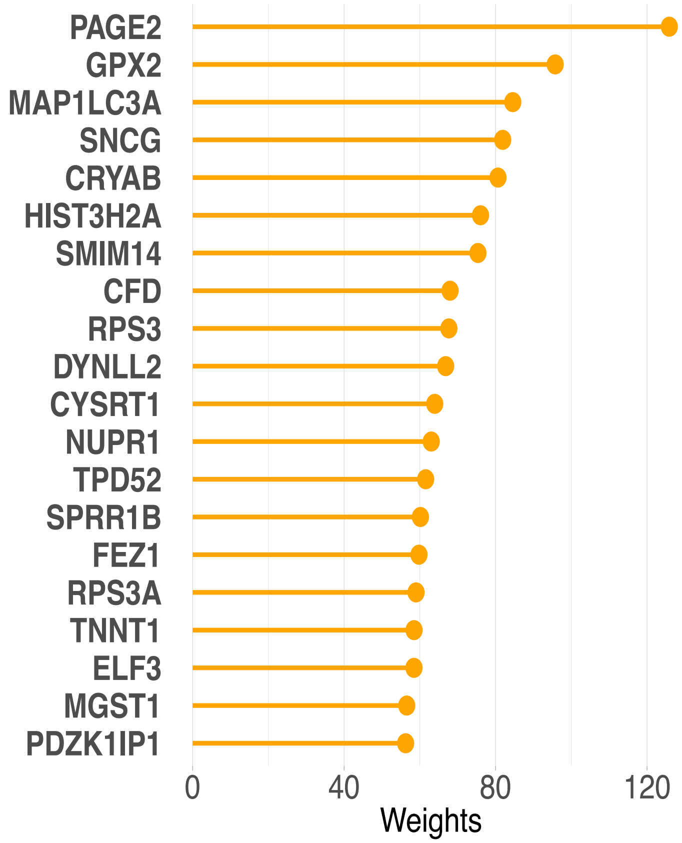

Epi GEM 78

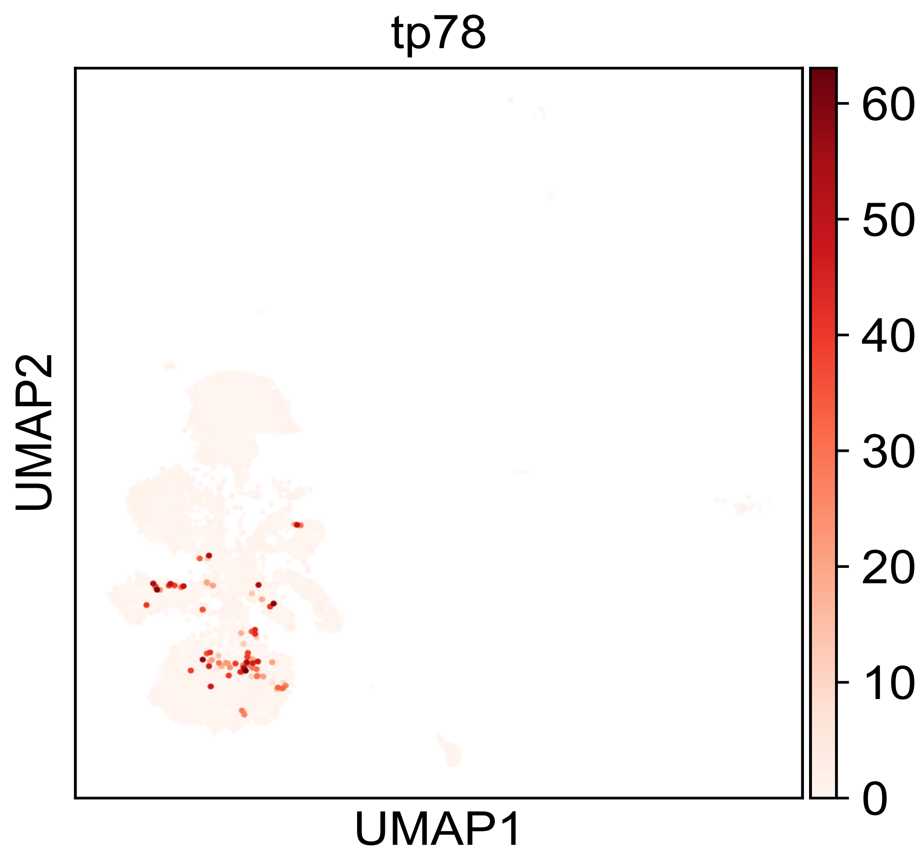

Cell Type

Epi_4

Epi_5

Top 20 genes

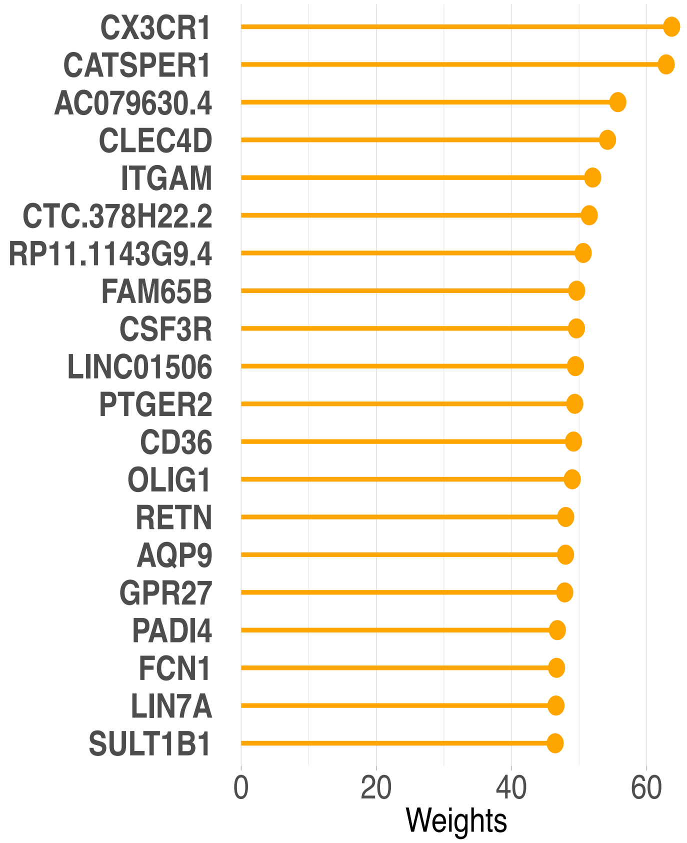

Endo GEM 1

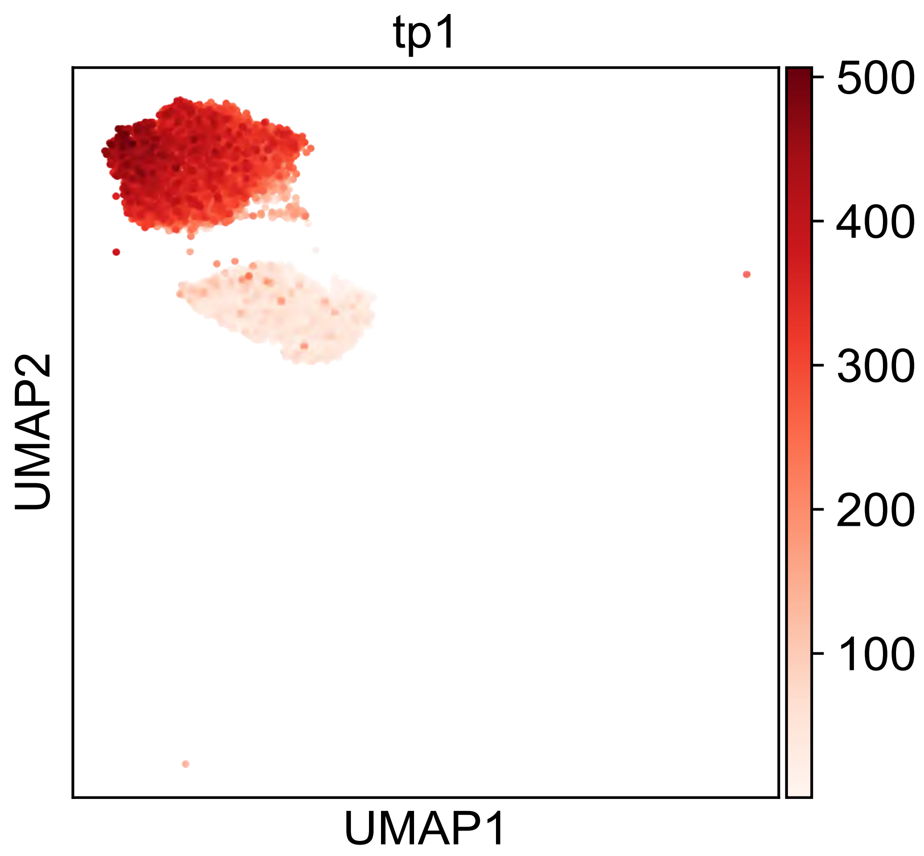

Cell Type

Endo_1

Endo_4

Endo_5

Endo_6

Top 20 genes

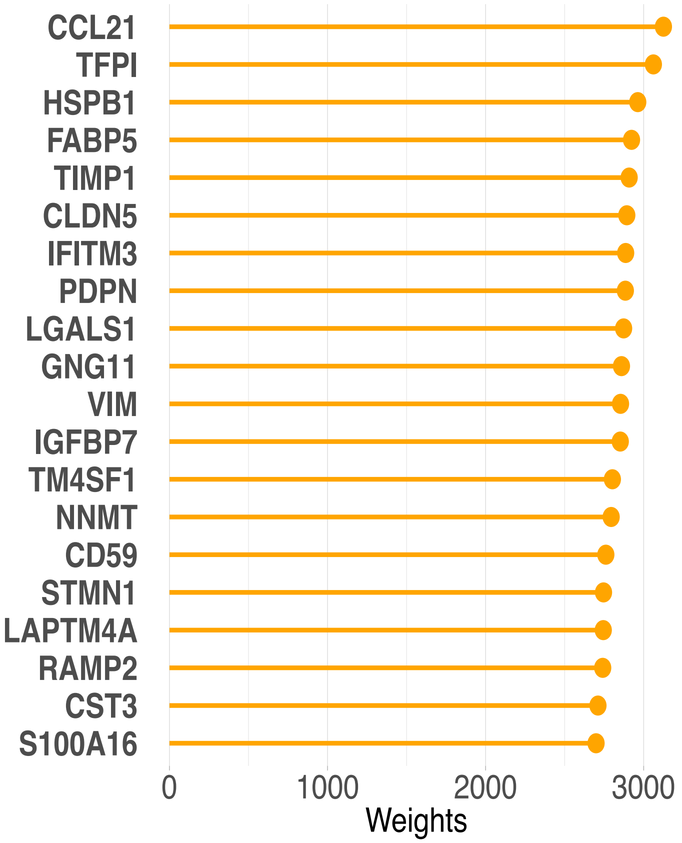

Endo GEM 2

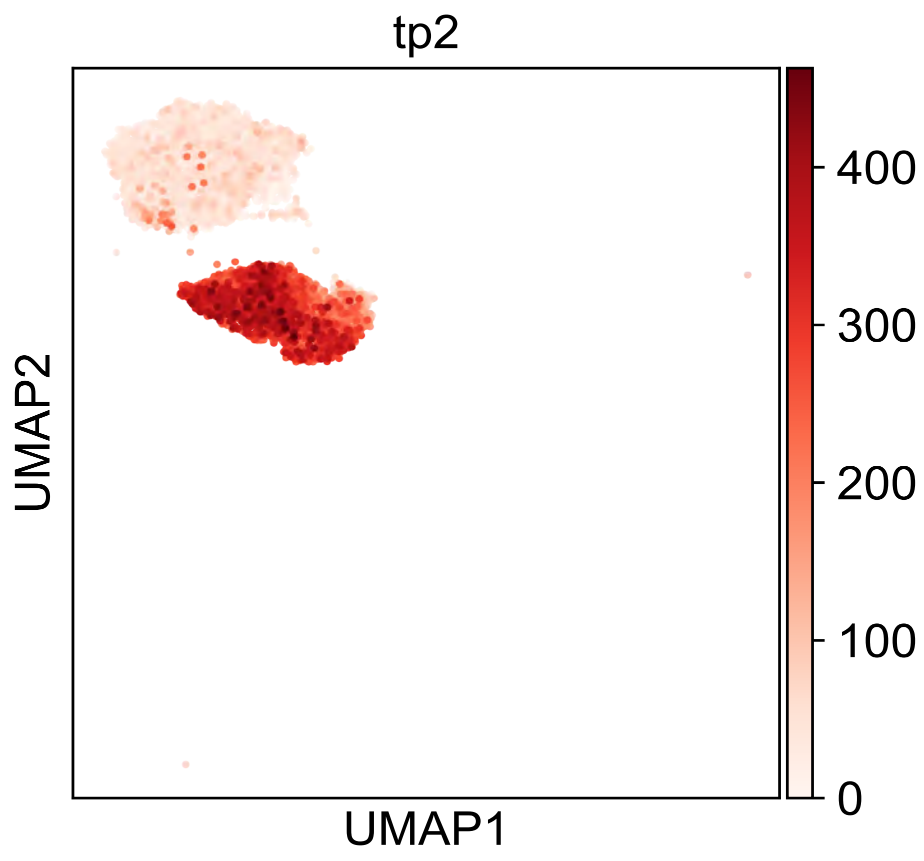

Cell Type

Endo_2

Endo_3

Endo_6

Top 20 genes

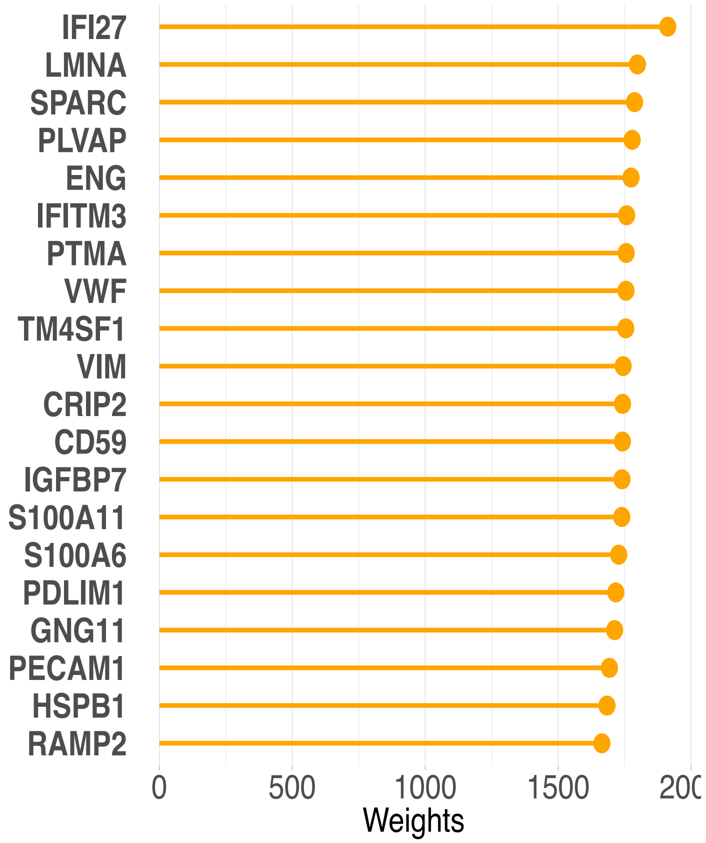

Endo GEM 3

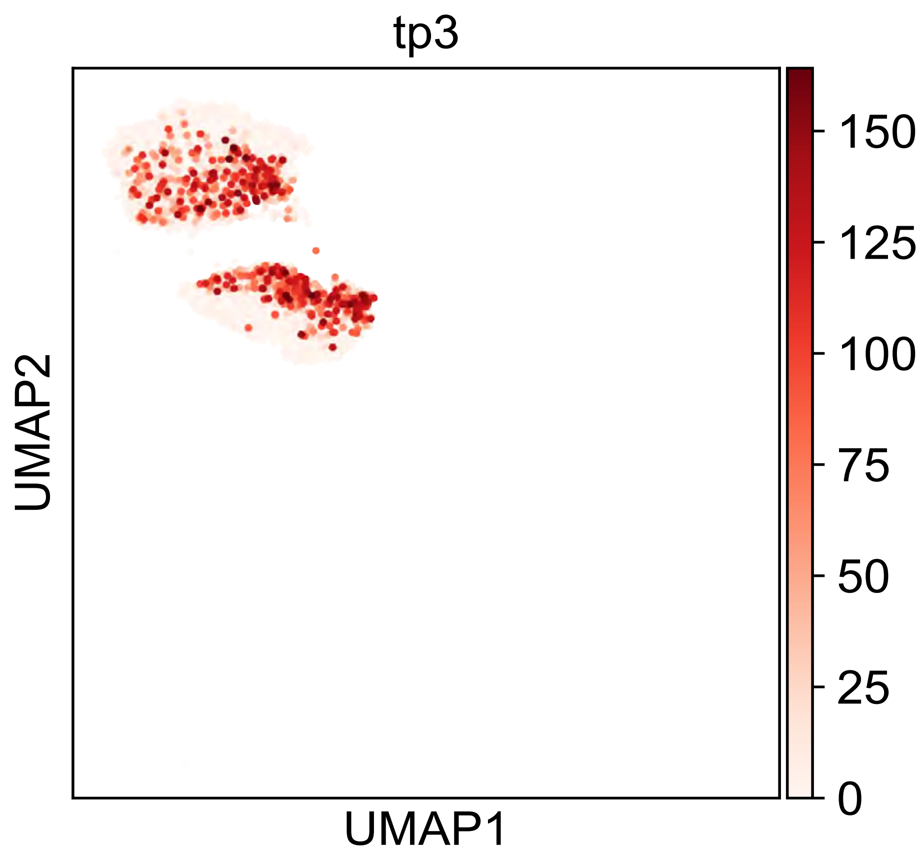

Cell Type

Endo_6

Top 20 genes

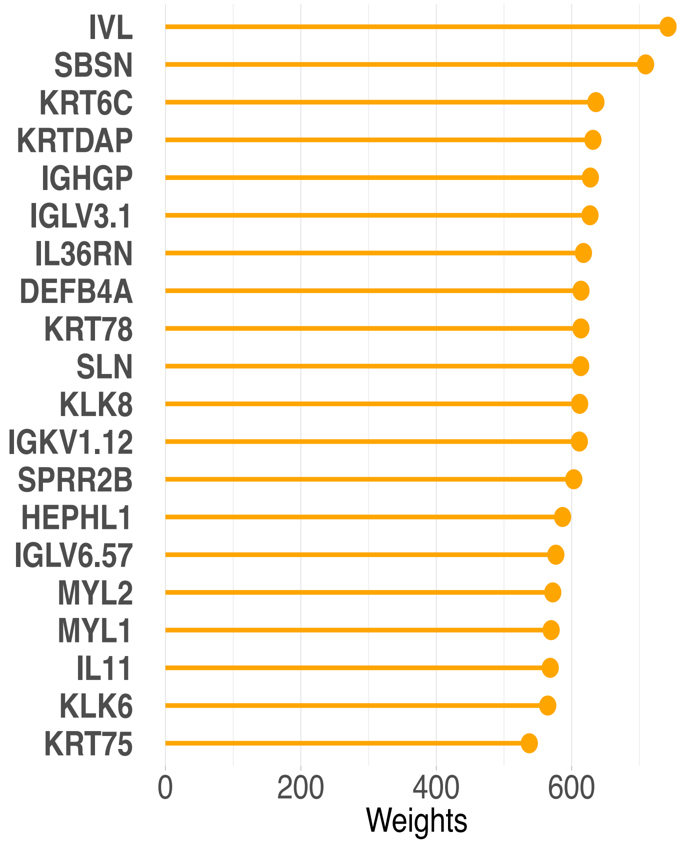

Endo GEM 4

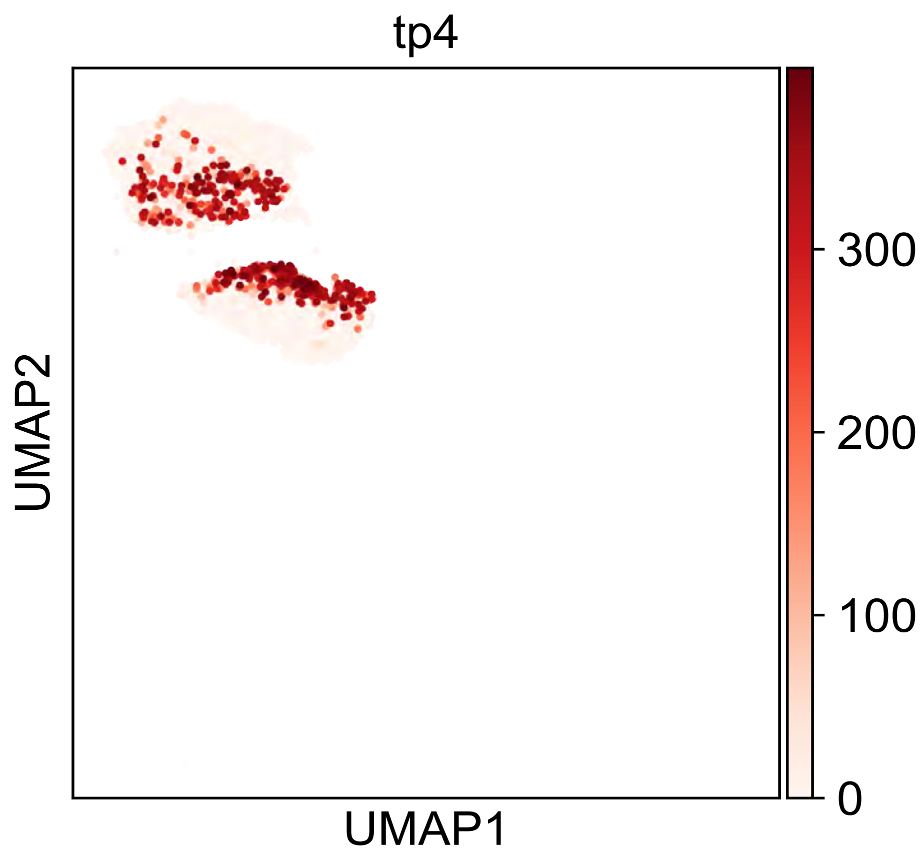

Cell Type

Endo_1

Endo_5

Endo_6

Top 20 genes

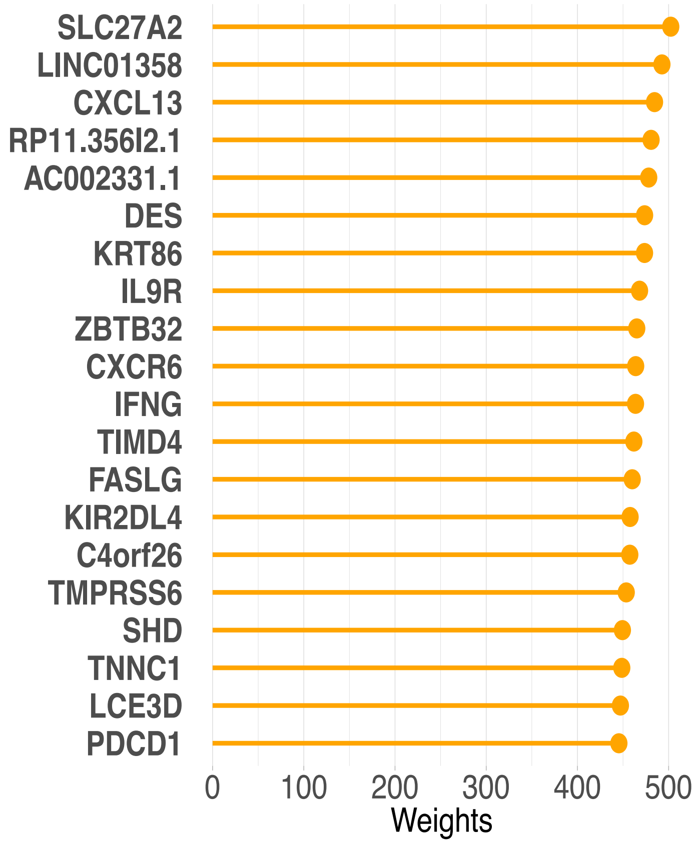

Endo GEM 5

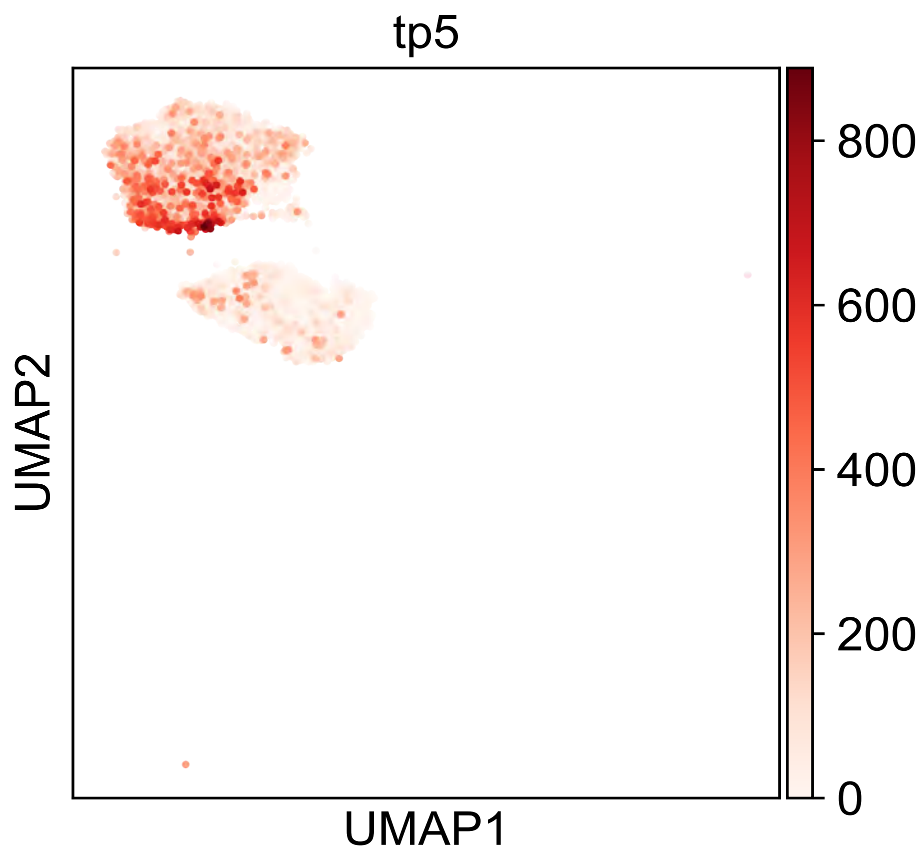

Cell Type

Endo_1

Endo_5

Endo_6

Top 20 genes

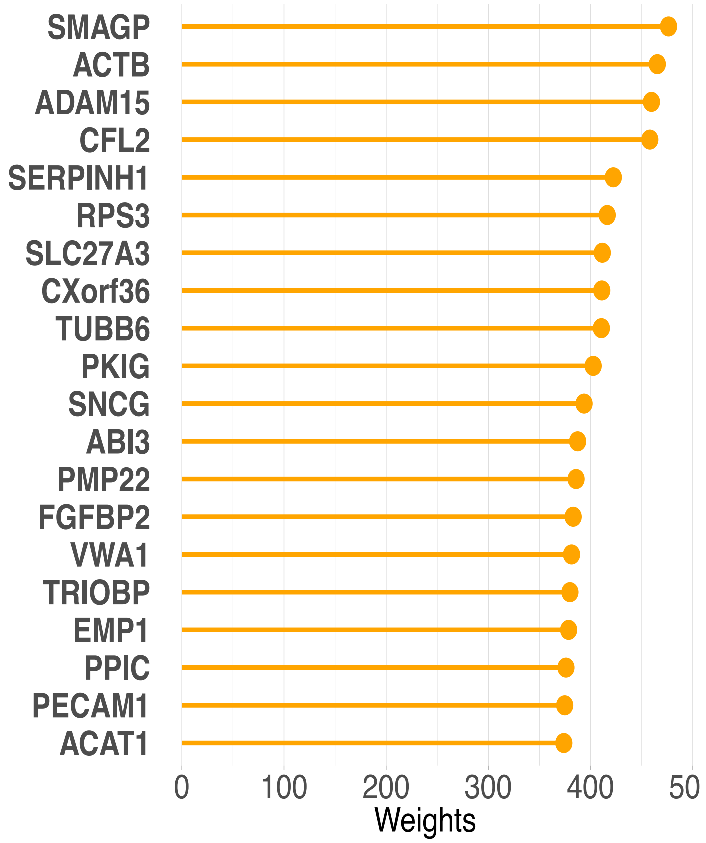

Endo GEM 6

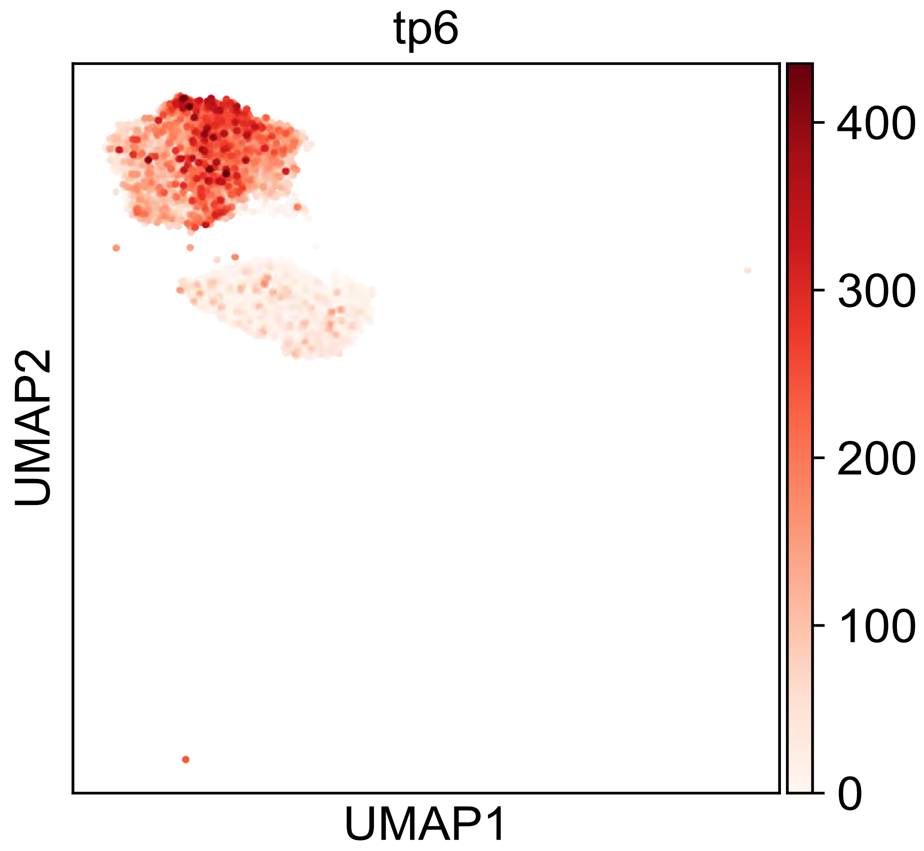

Cell Type

Endo_1

Endo_4

Endo_5

Endo_6

Top 20 genes

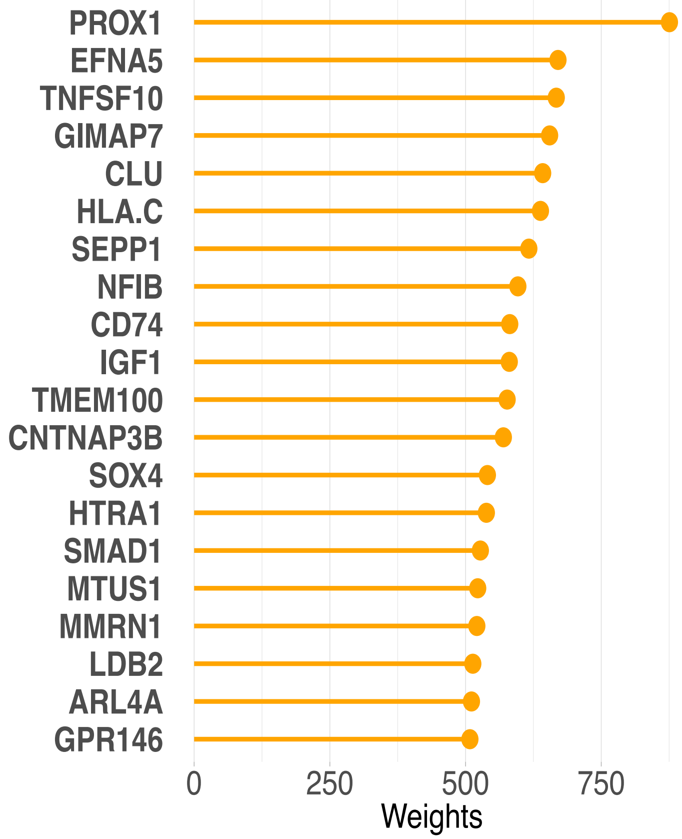

Endo GEM 7

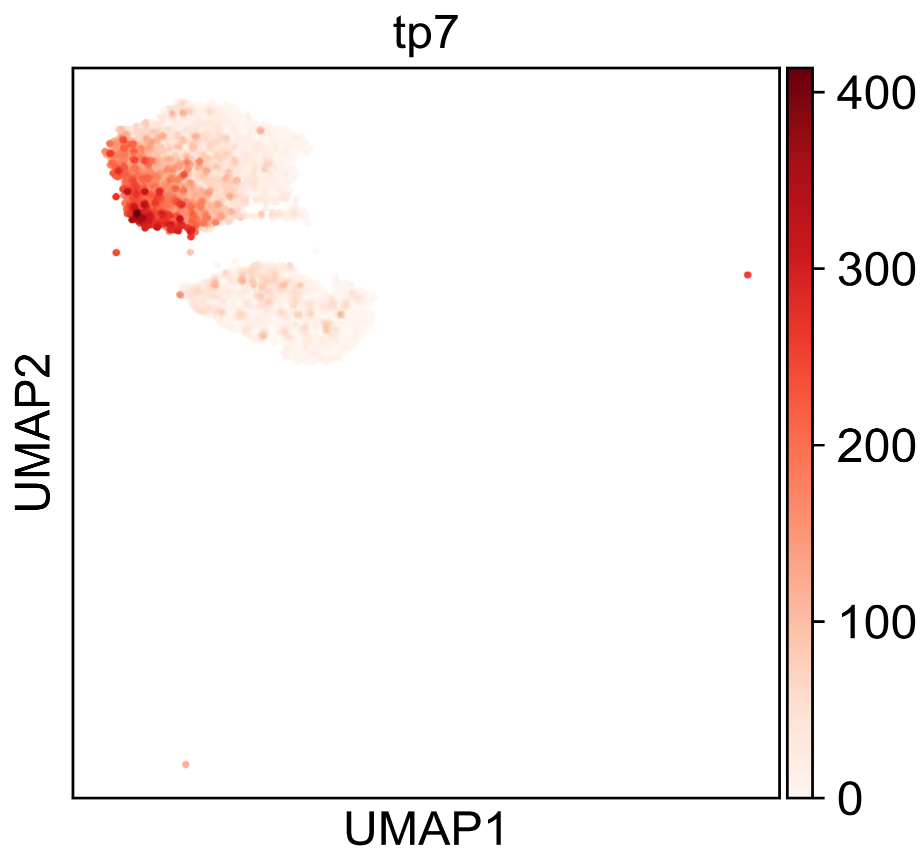

Cell Type

Endo_1

Endo_5

Top 20 genes

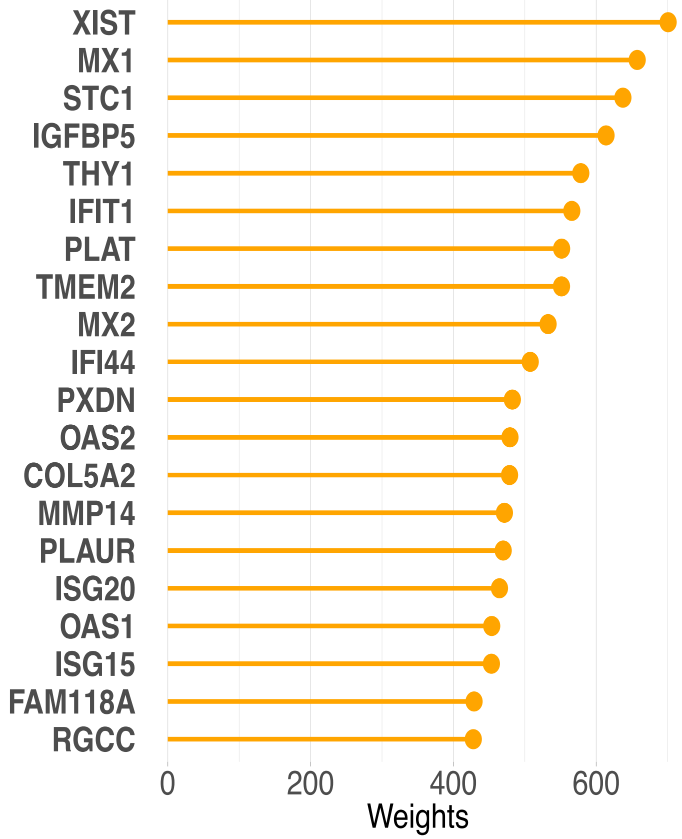

Endo GEM 8

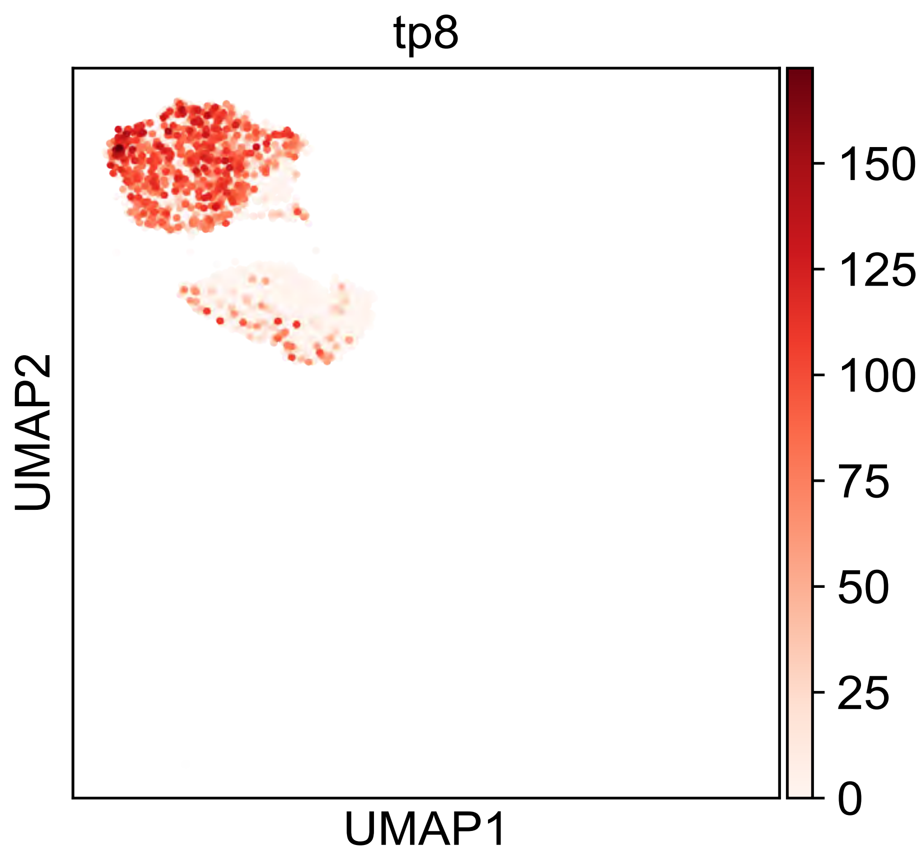

Cell Type

Endo_1

Endo_4

Endo_5

Endo_6

Top 20 genes

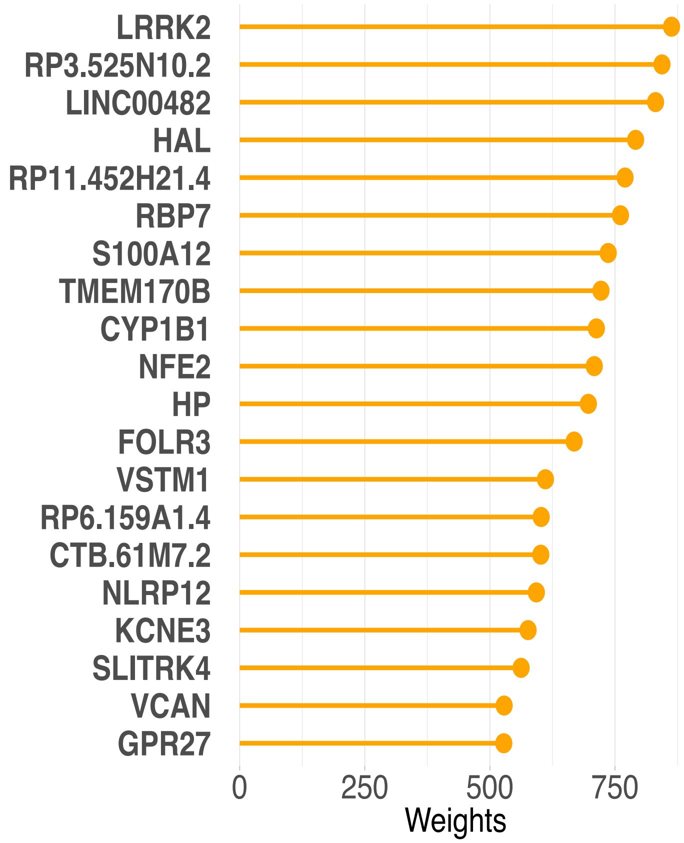

Endo GEM 10

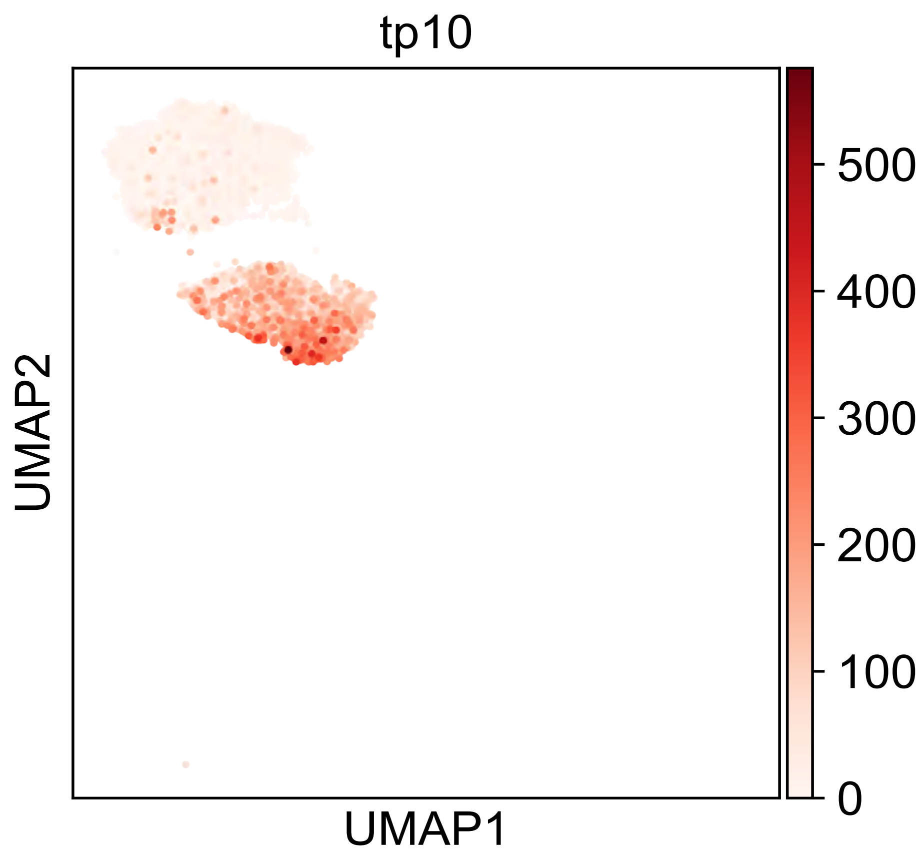

Cell Type

Endo_2

Endo_3

Endo_6

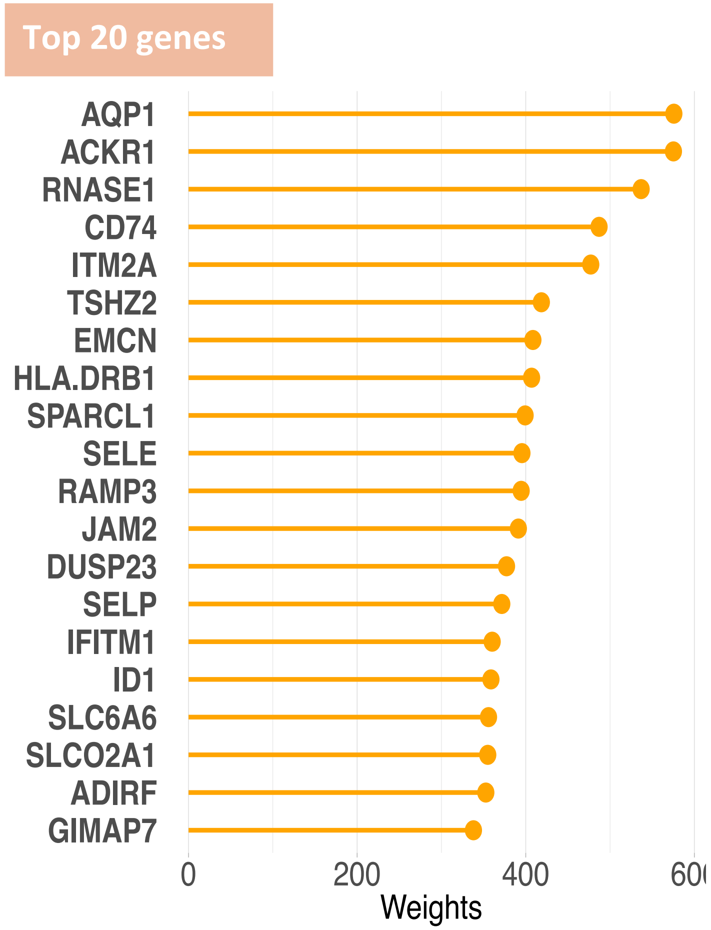

Endo GEM 11

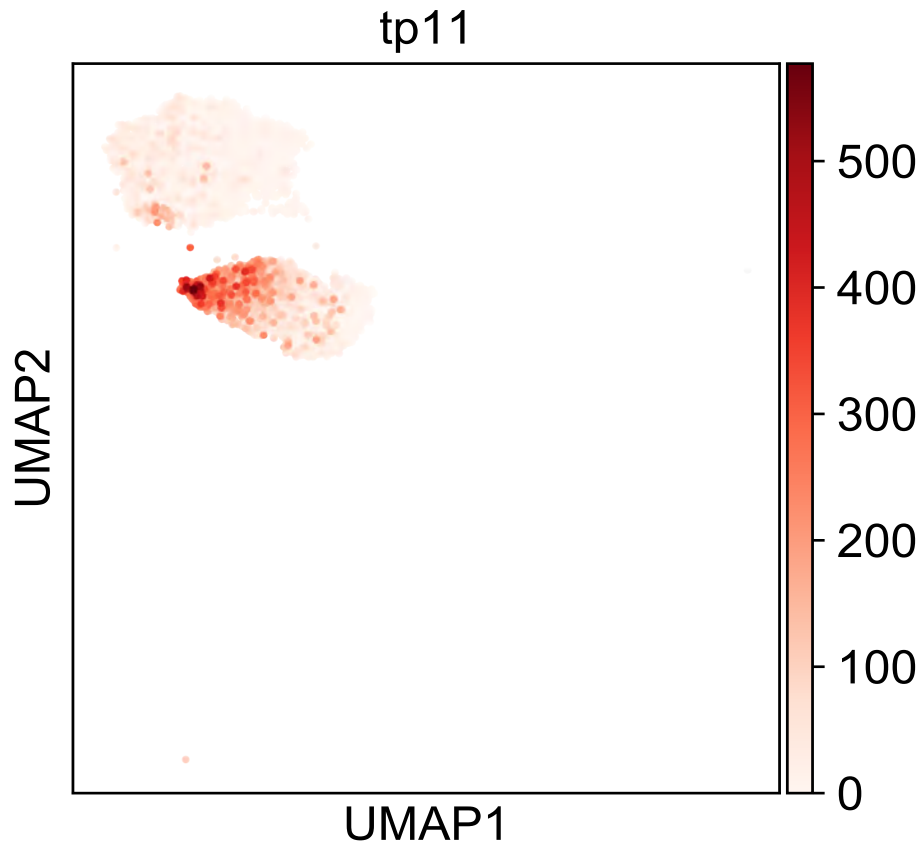

Cell Type

Endo_2

Endo_3

Top 20 genes

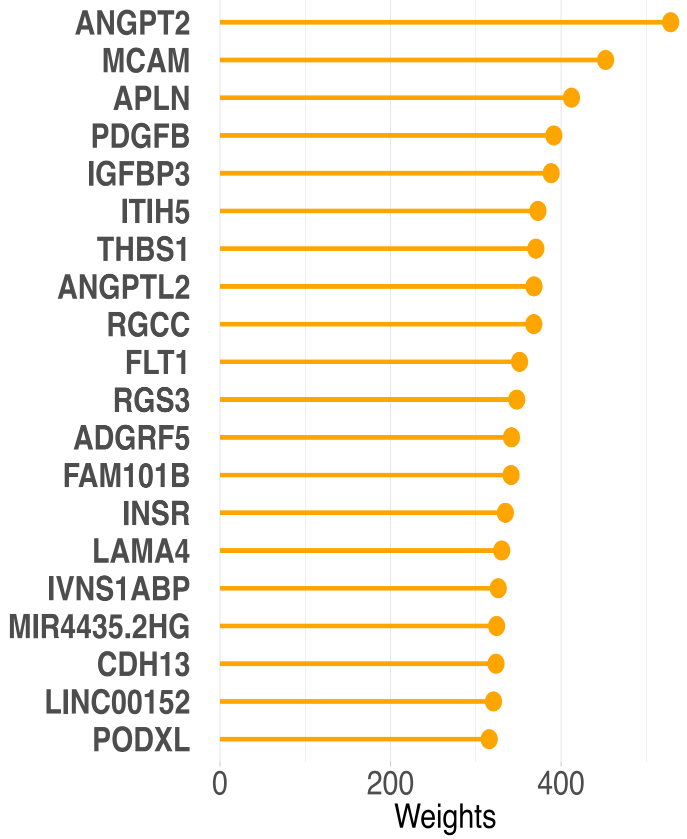

Endo GEM 12

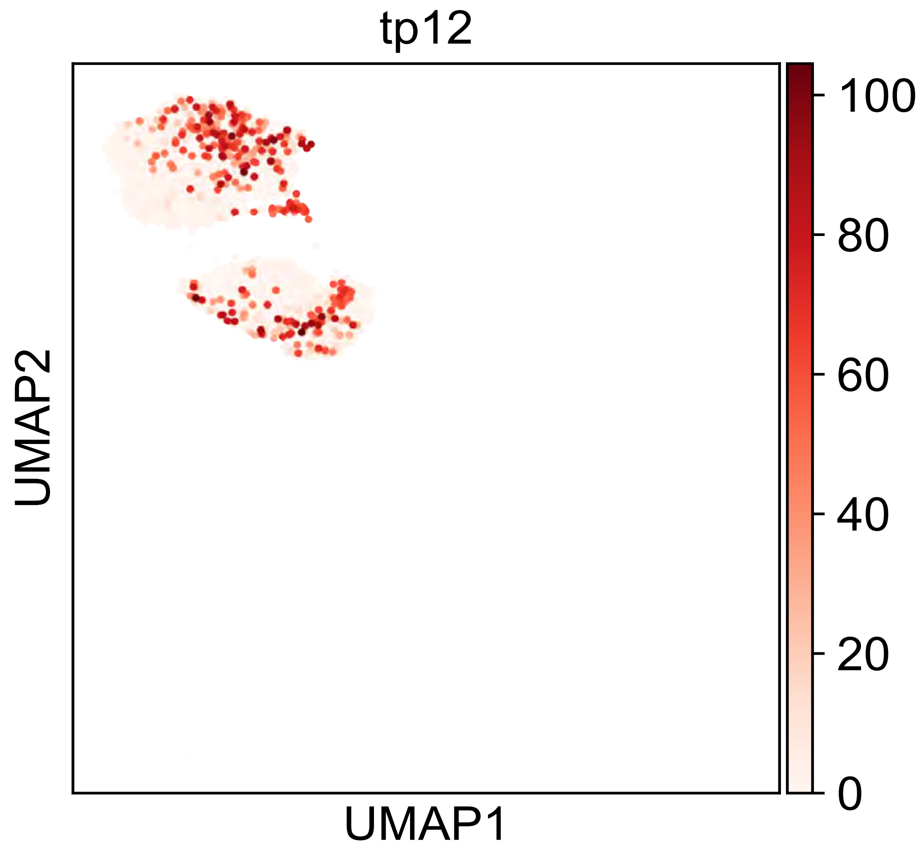

Cell Type

Endo_2

Endo_3

Endo_4

Top 20 genes

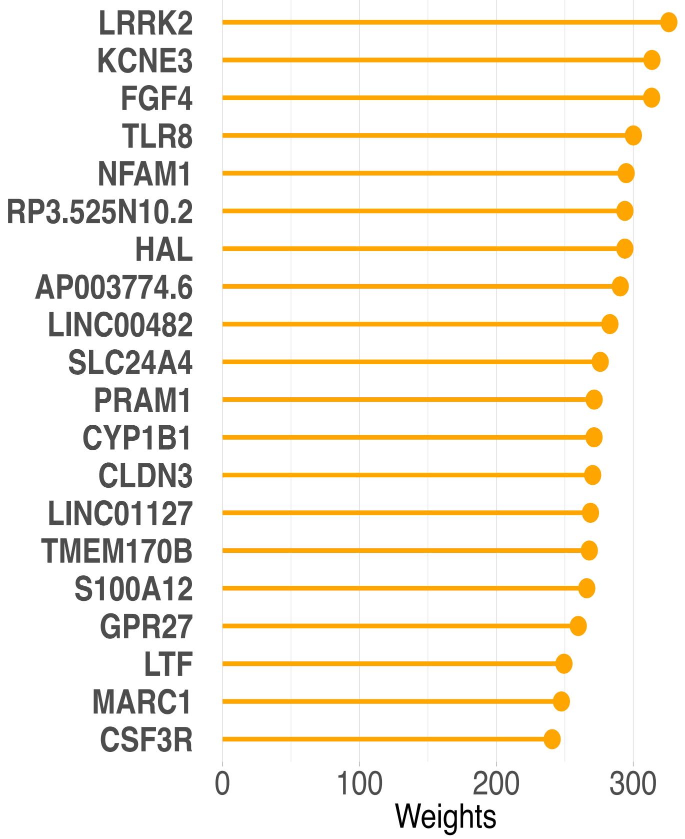

Endo GEM 14

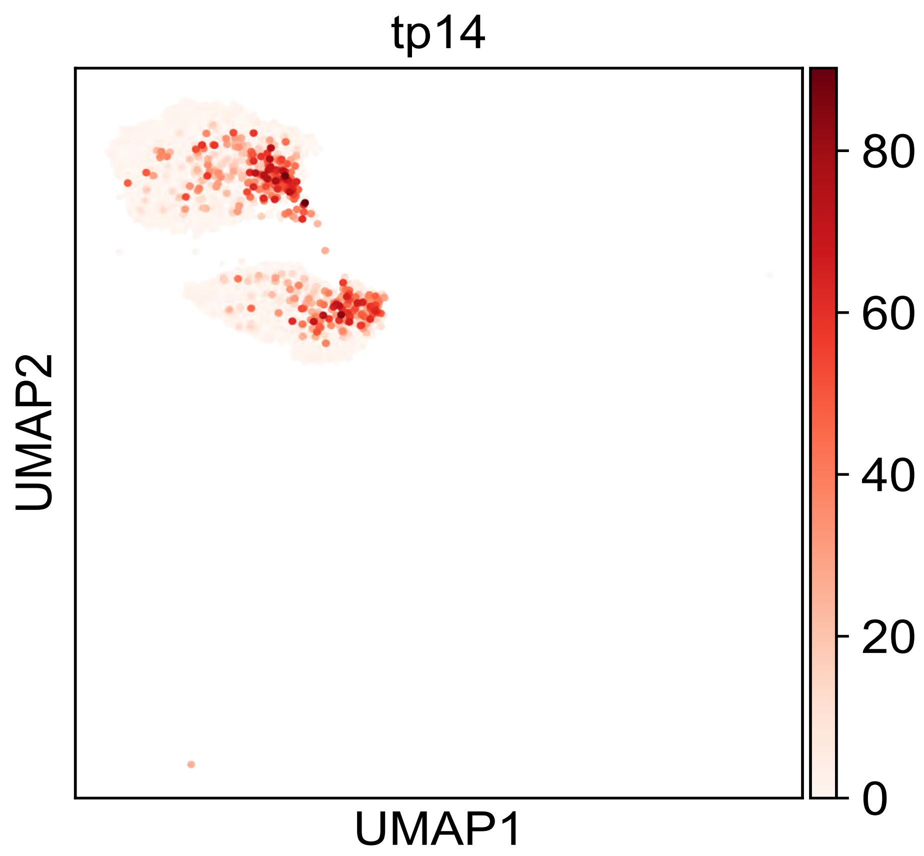

Cell Type

Endo_3

Endo_4

Endo_6

Top 20 genes

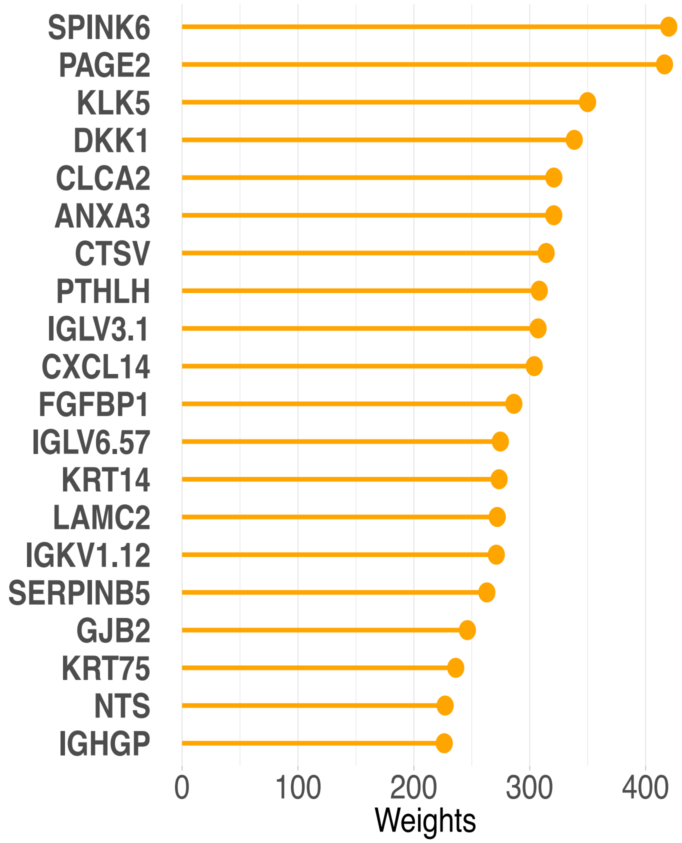

Endo GEM 15

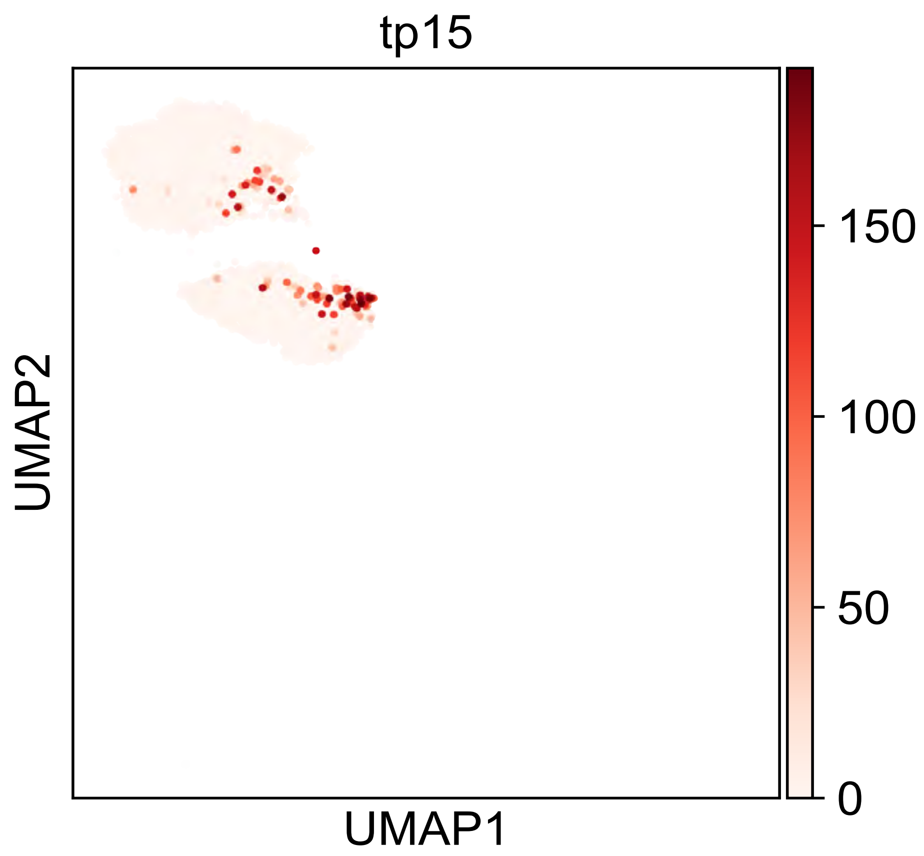

Cell Type

Endo_6

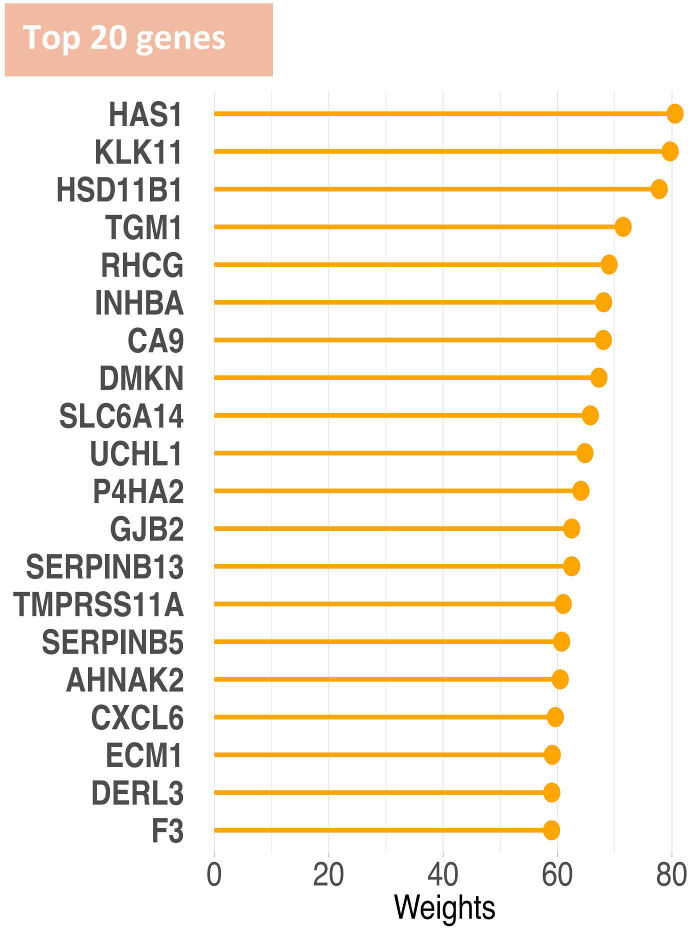

Endo GEM 16

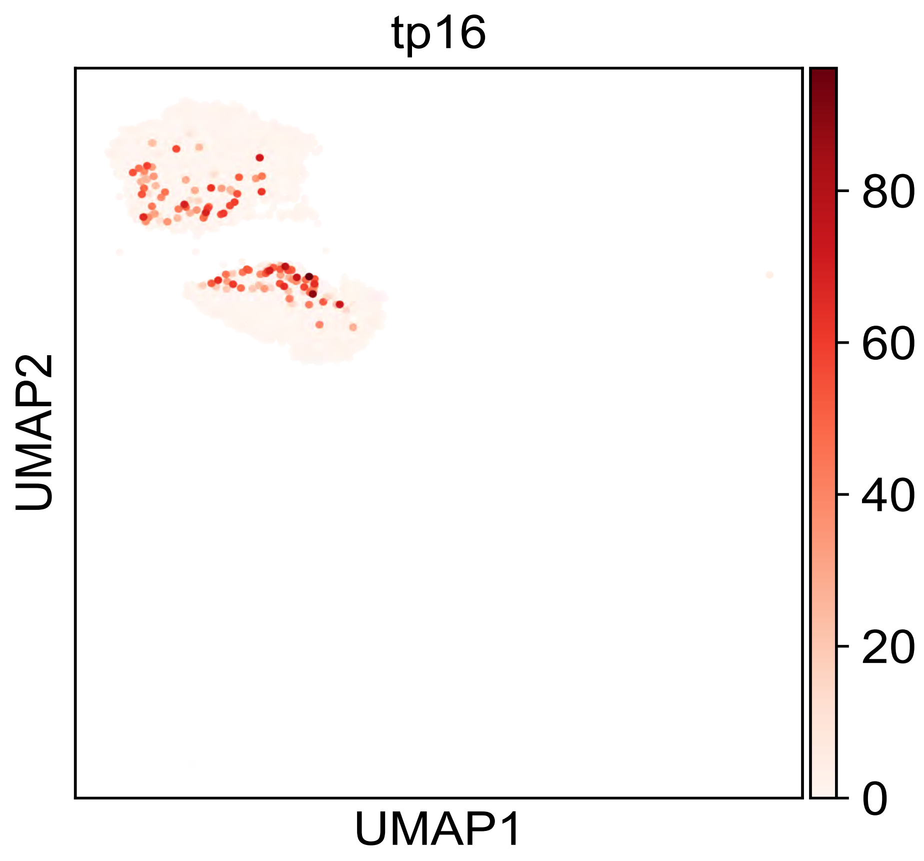

Cell Type

Endo_1

Endo_5

Endo_6

Top 20 genes

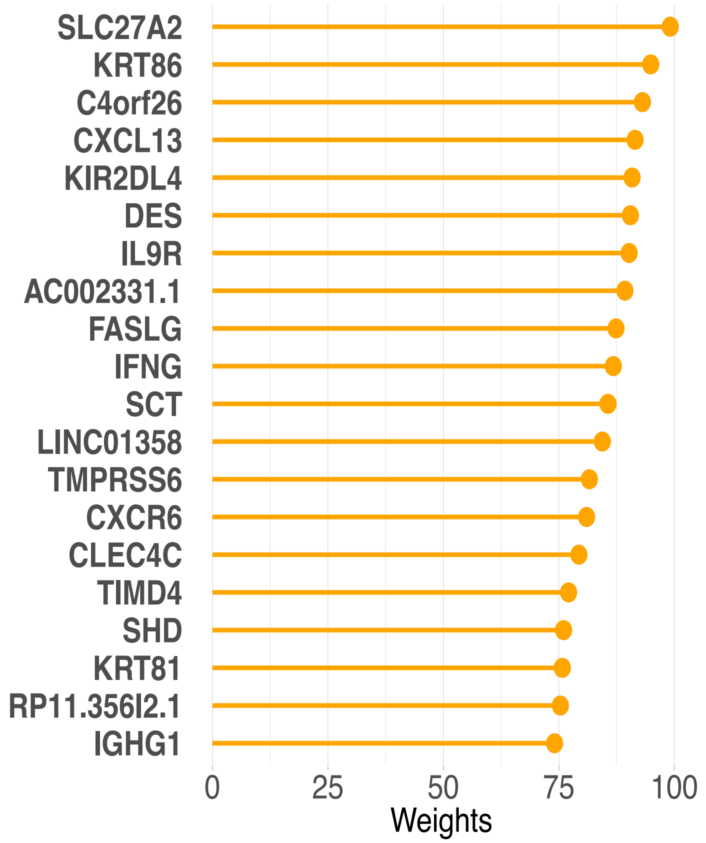

Endo GEM 19

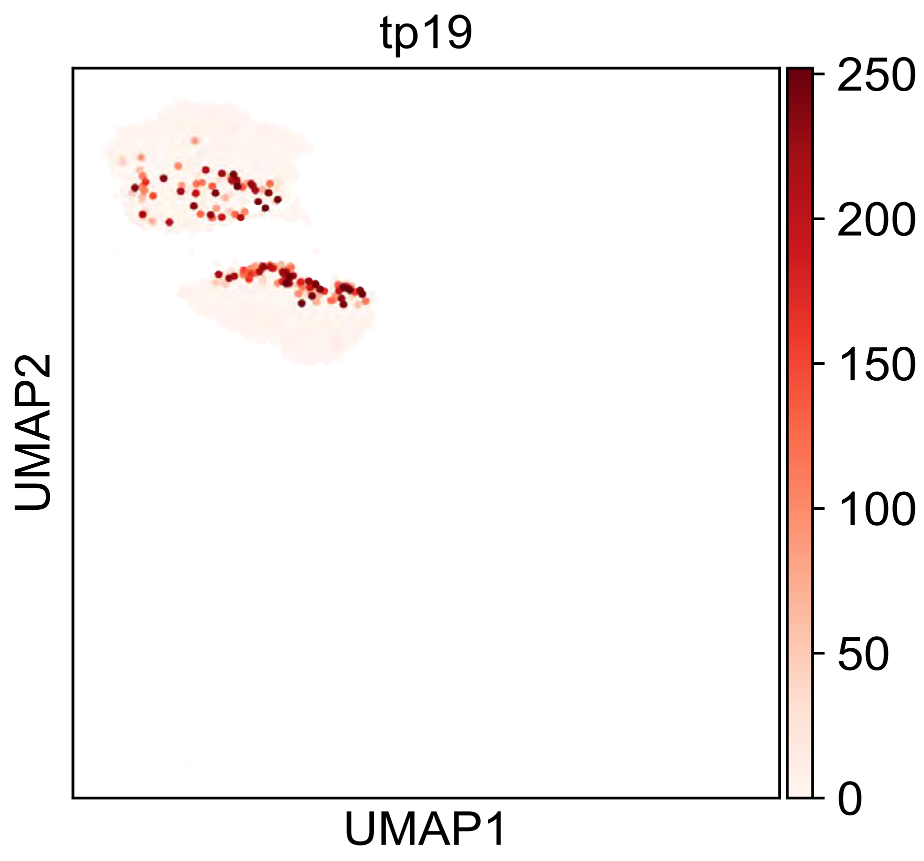

Cell Type

Endo_5

Endo_6

Top 20 genes

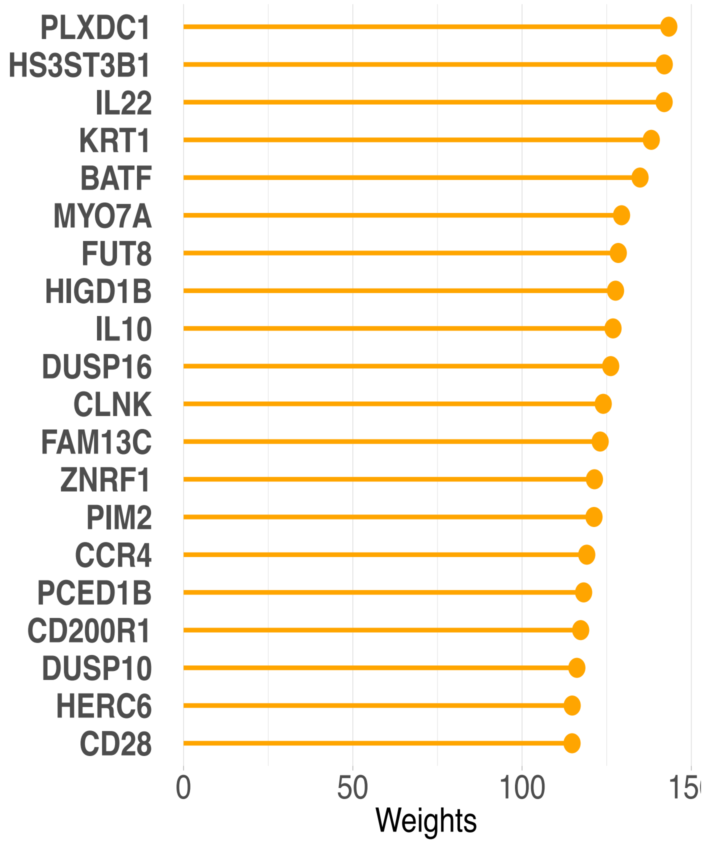

Endo GEM 21

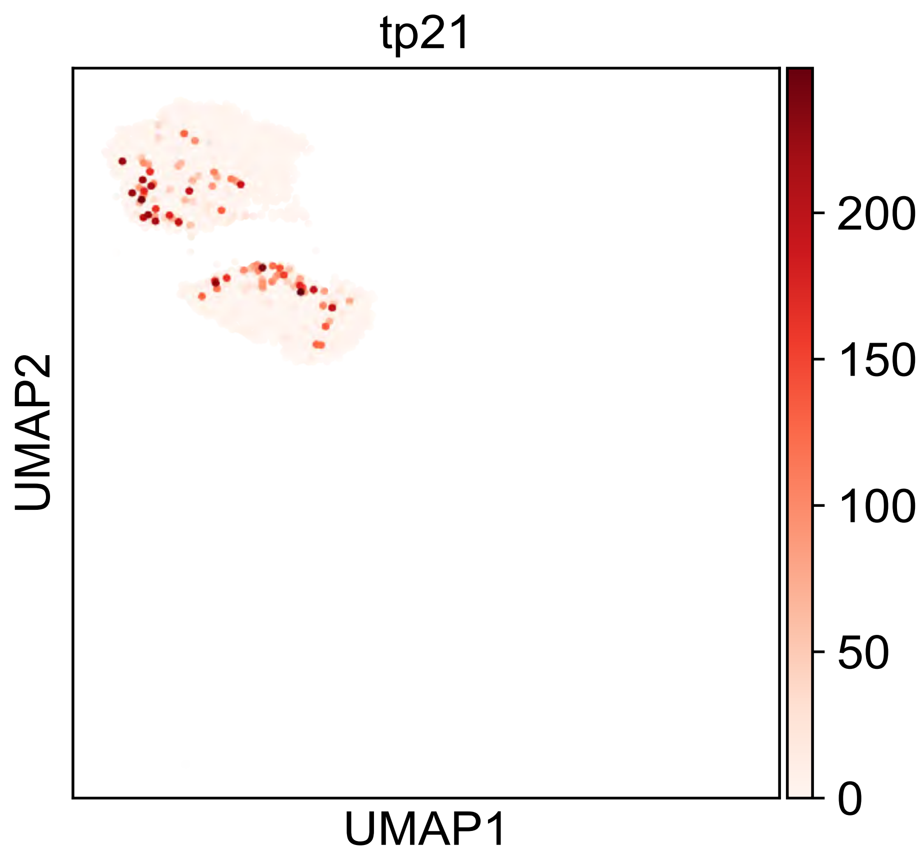

Cell Type

Endo_1

Endo_6

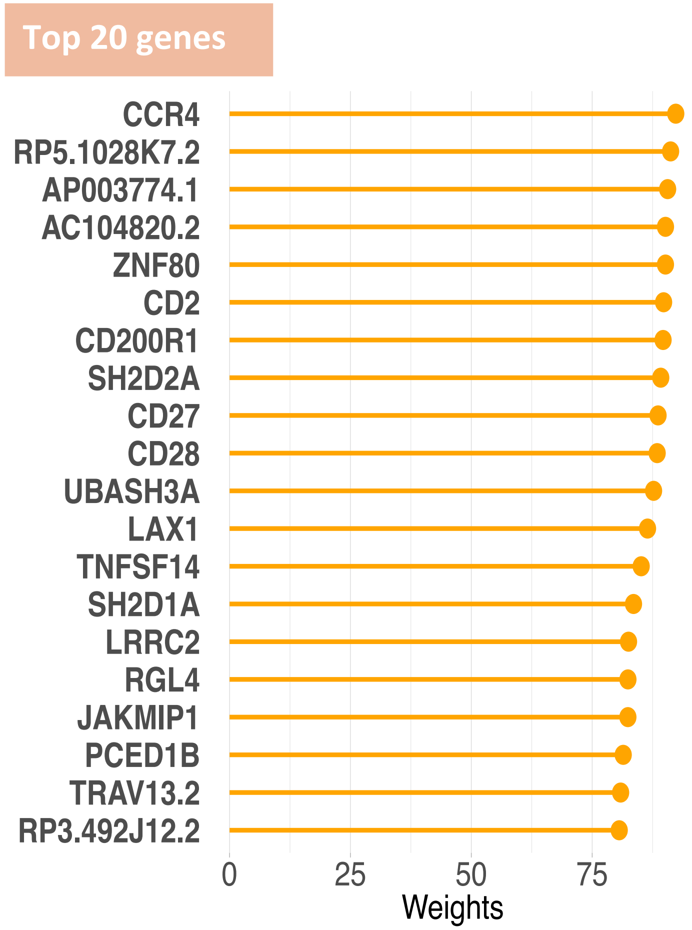

Endo GEM 23

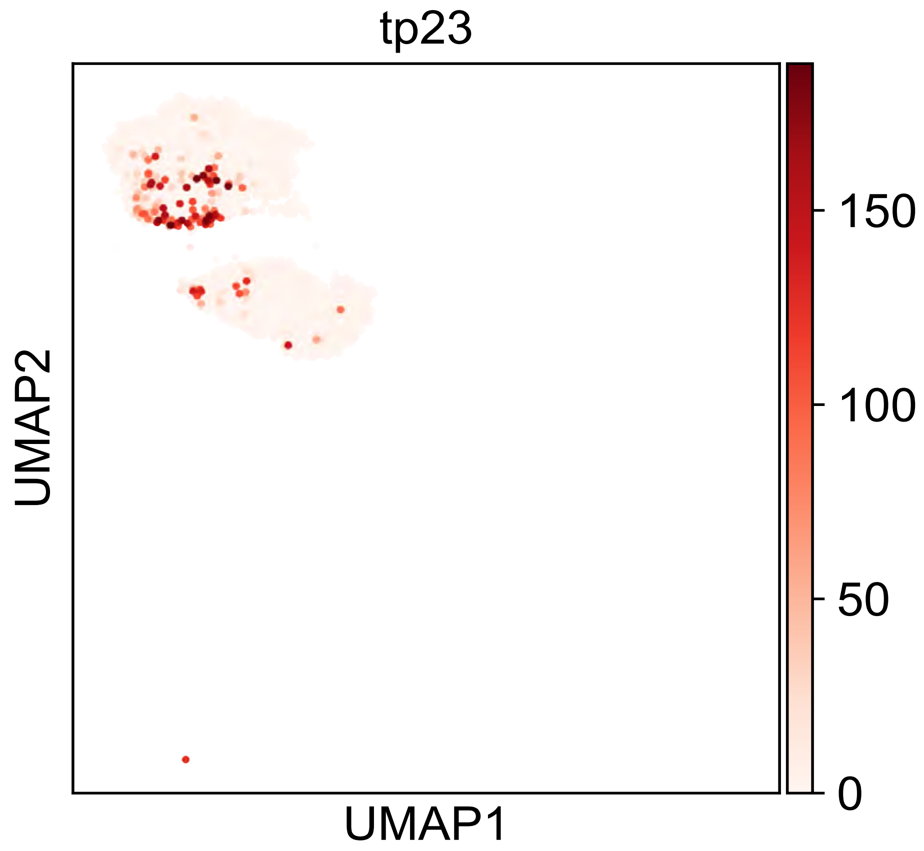

Cell Type

Endo_5

Top 20 genes

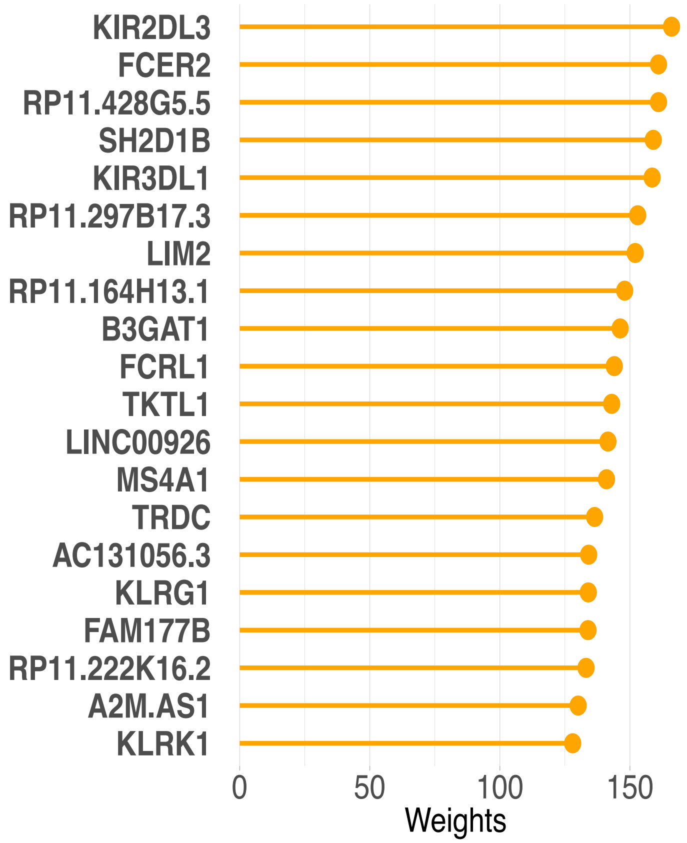

Endo GEM 24

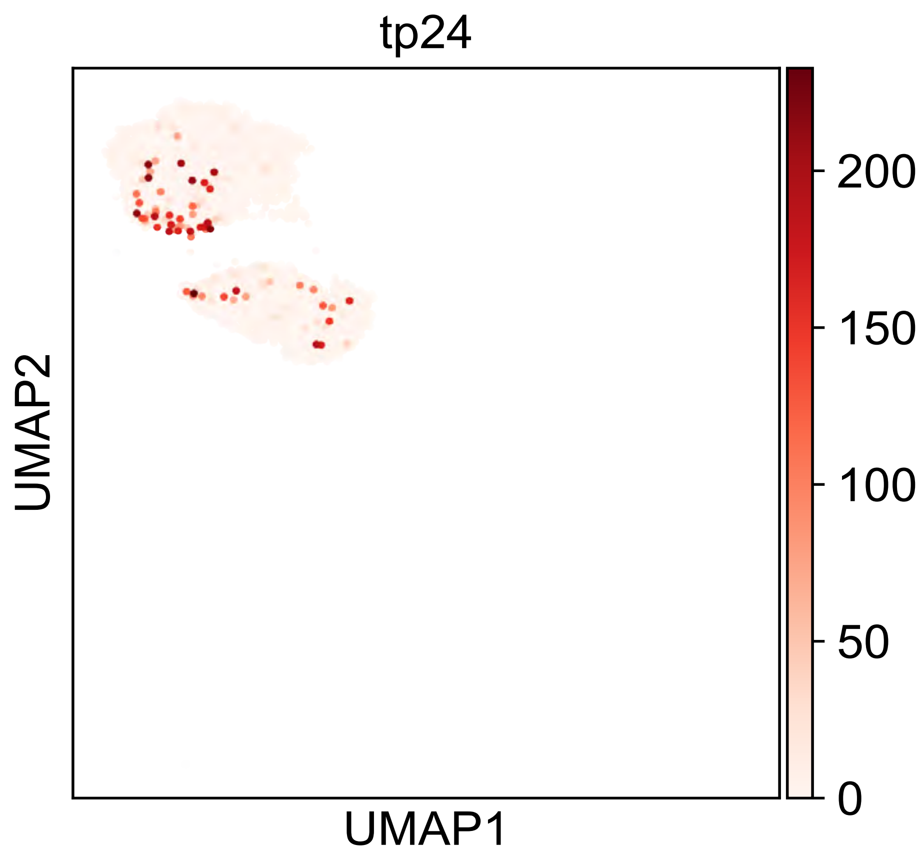

Cell Type

Endo_1

Endo_3

Endo_5

Top 20 genes

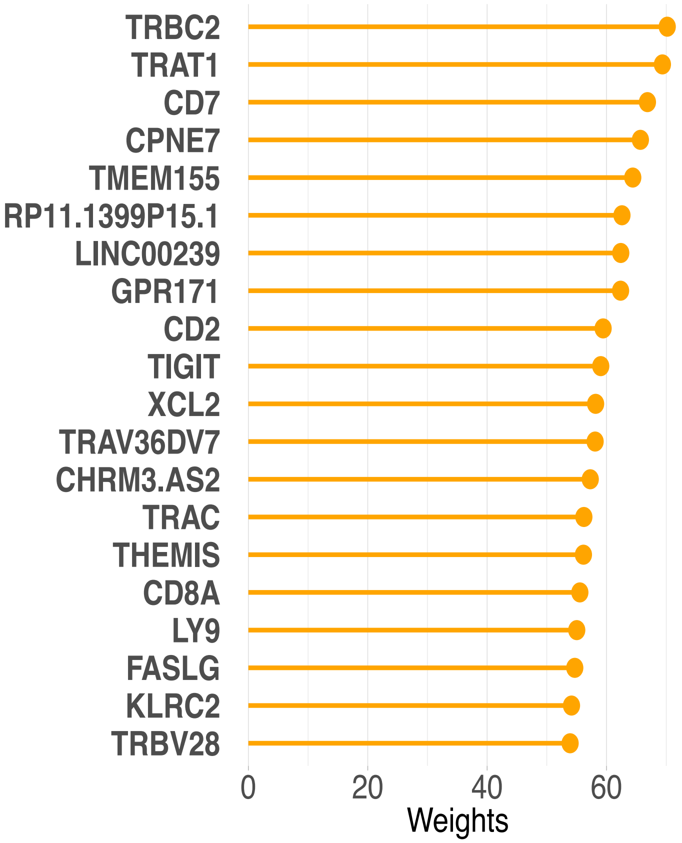

Endo GEM 28

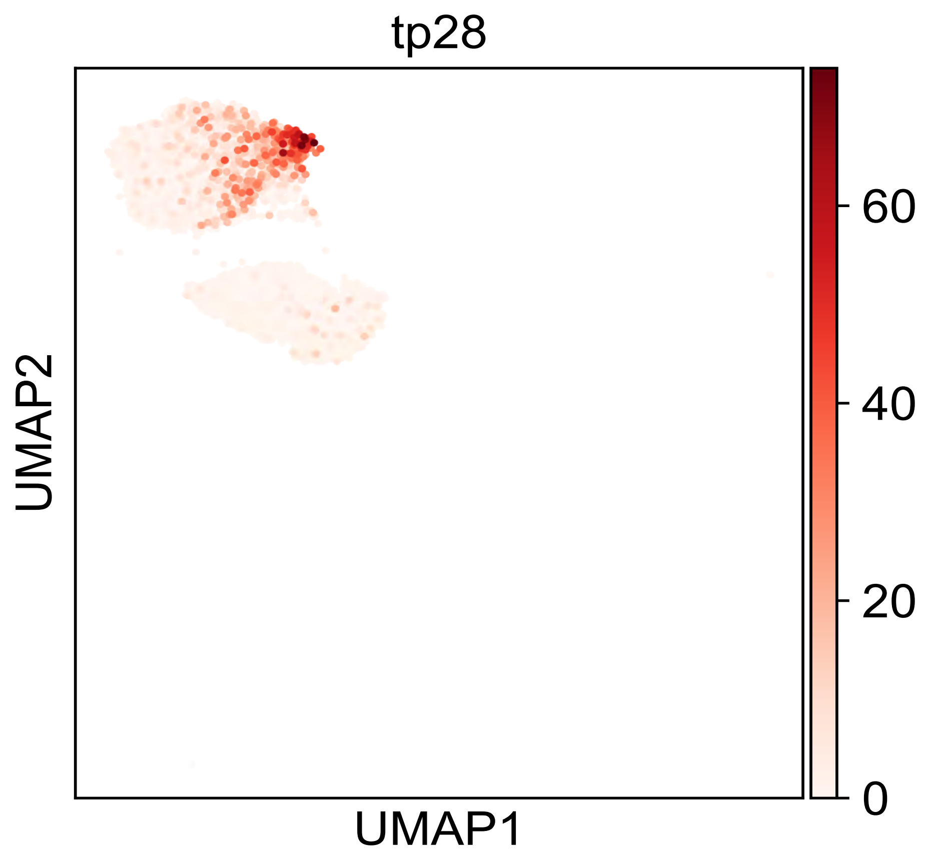

Cell Type

Endo_4

Endo_5

Top 20 genes

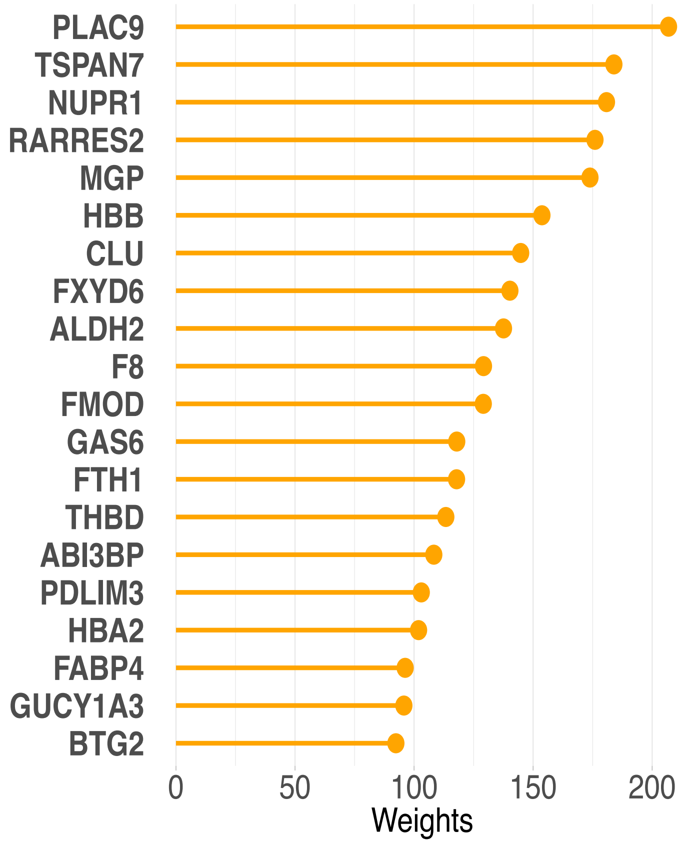

Endo GEM 30

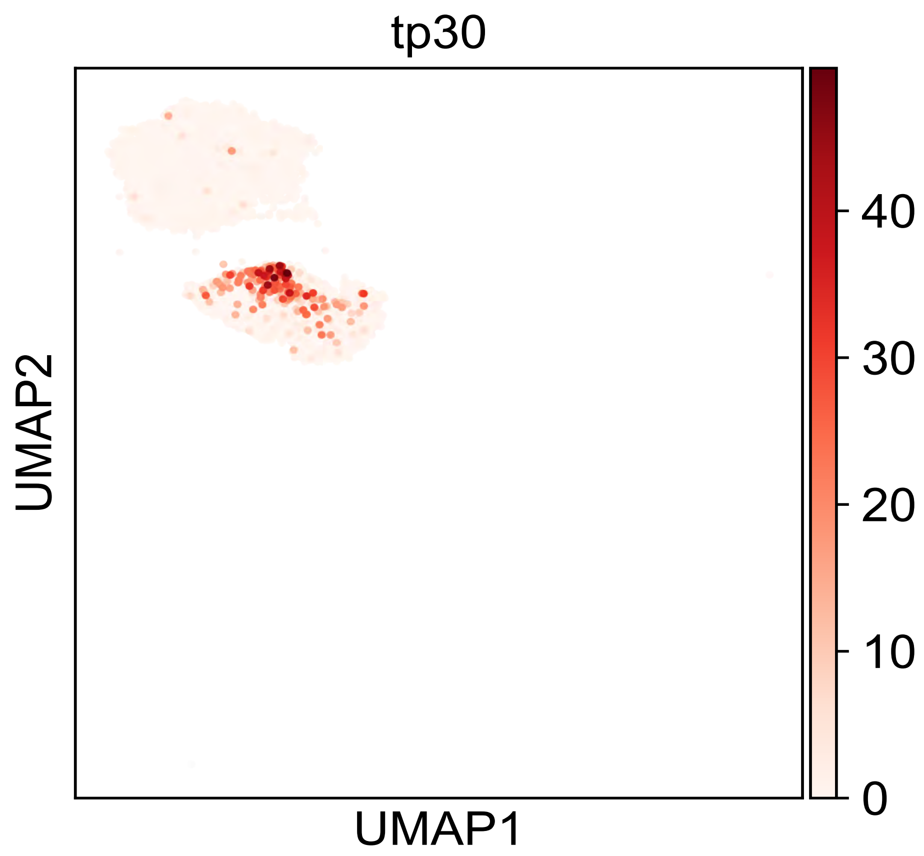

Cell Type

Endo_6

Top 20 genes

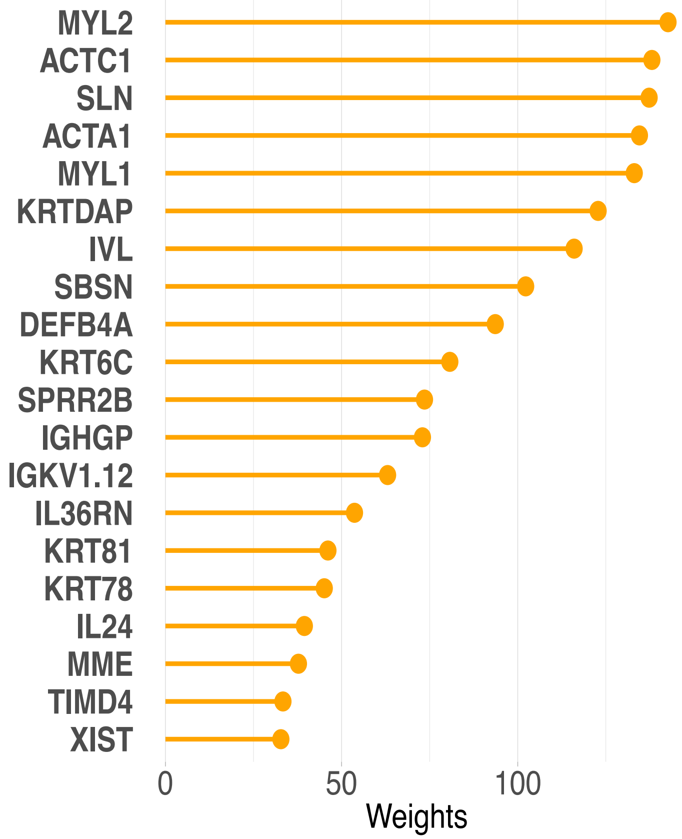

Endo GEM 31

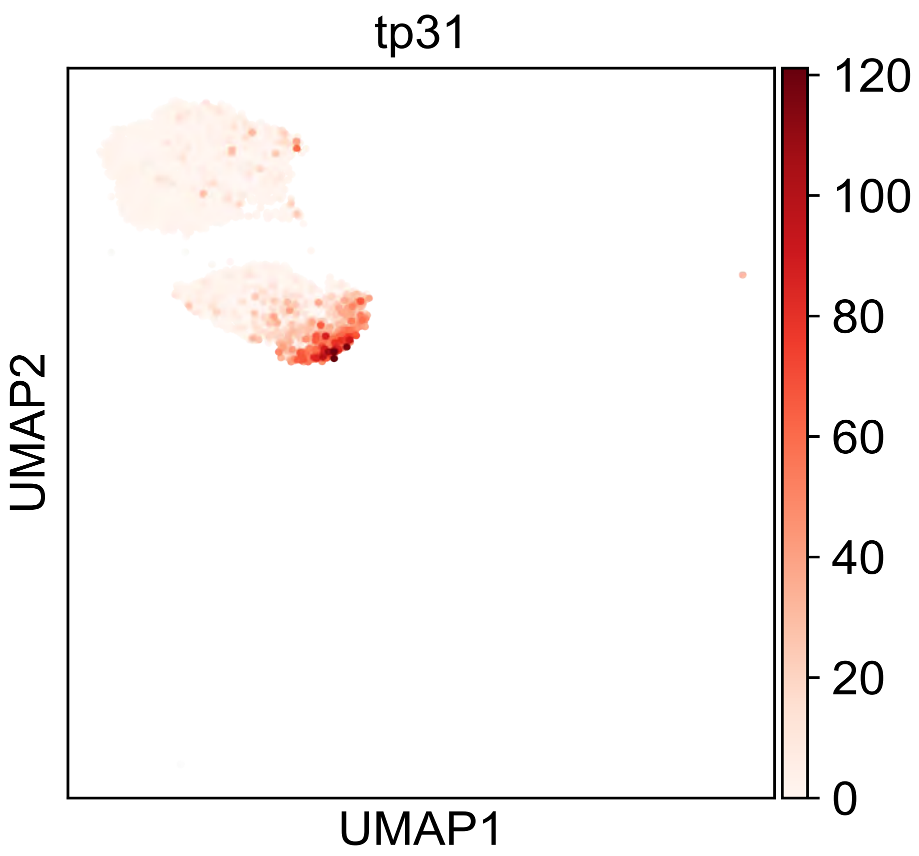

Cell Type

Endo_3

Top 20 genes

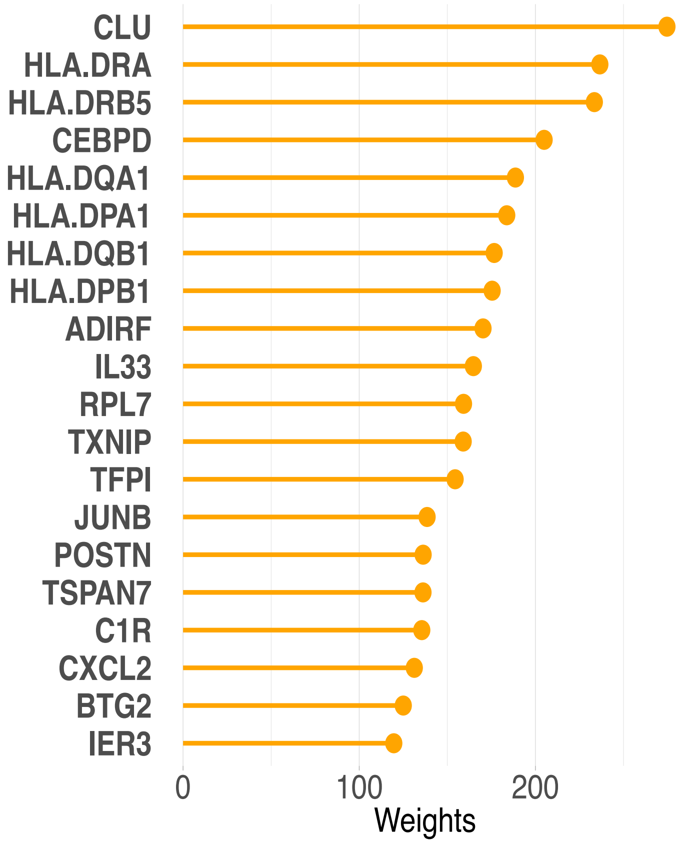

Endo GEM 34

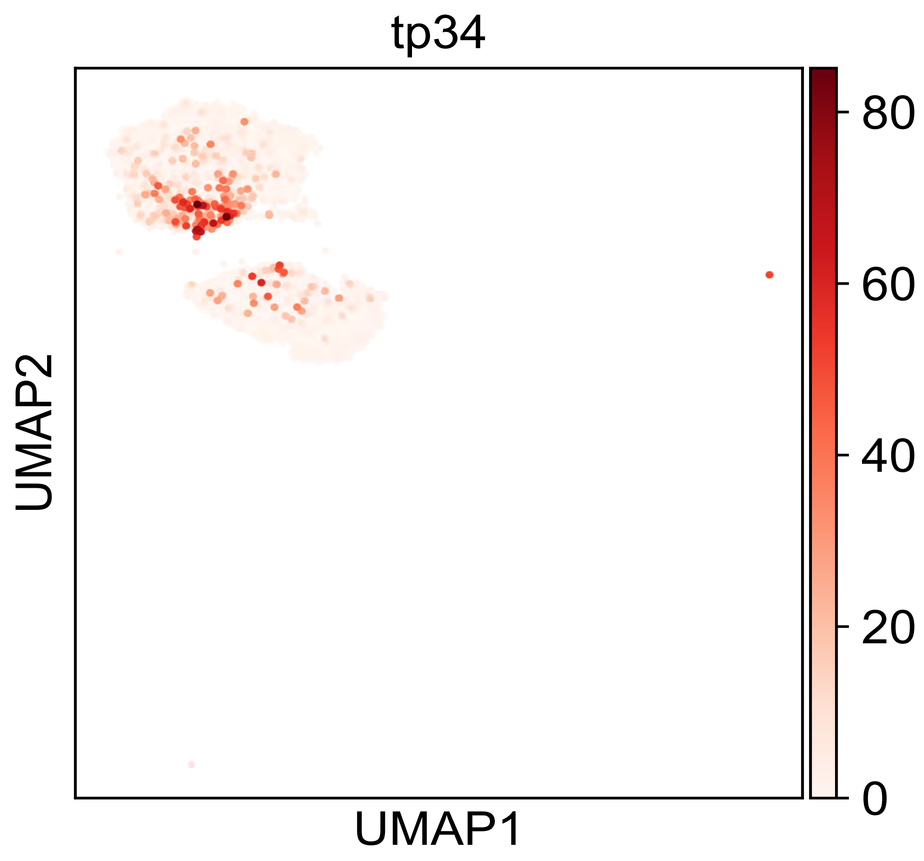

Cell Type

Endo_5

Endo_6

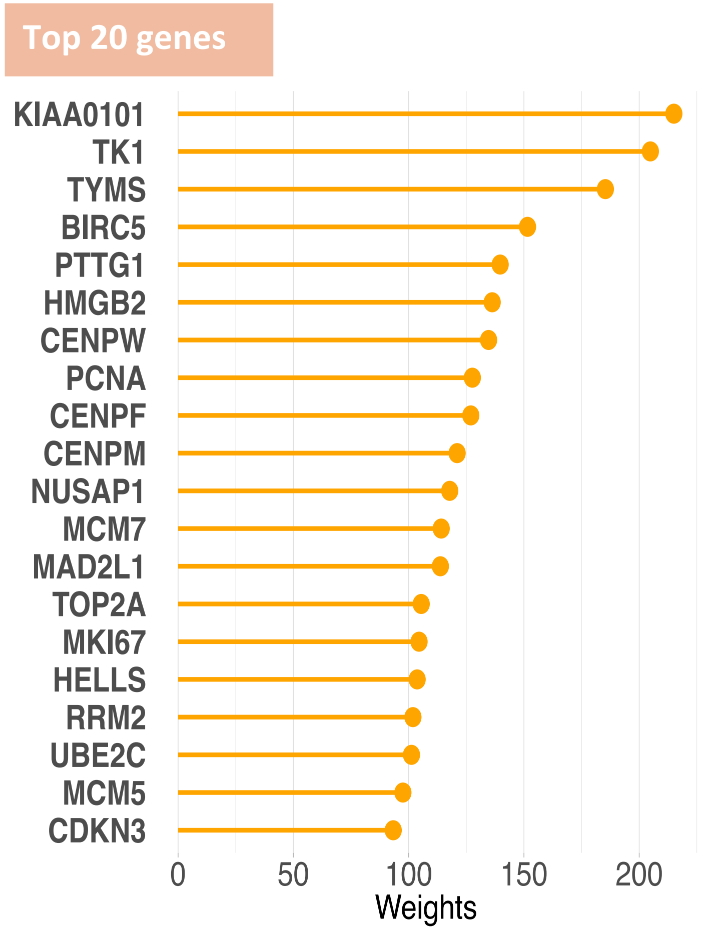

Endo GEM 35

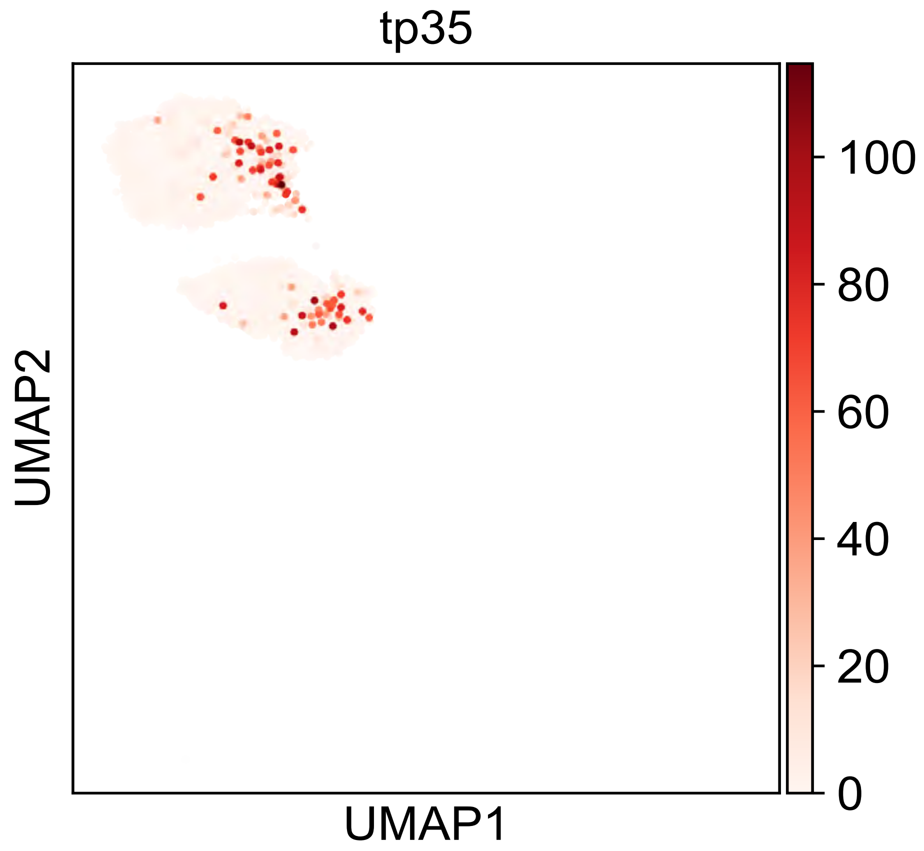

Cell Type

Endo_3

Endo_4

Top 20 genes

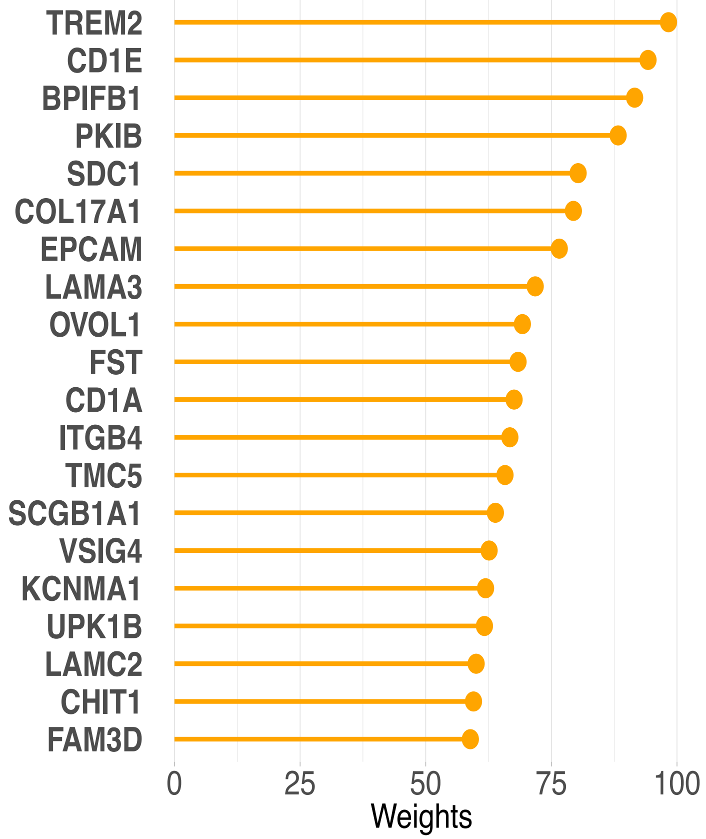

Endo GEM 38

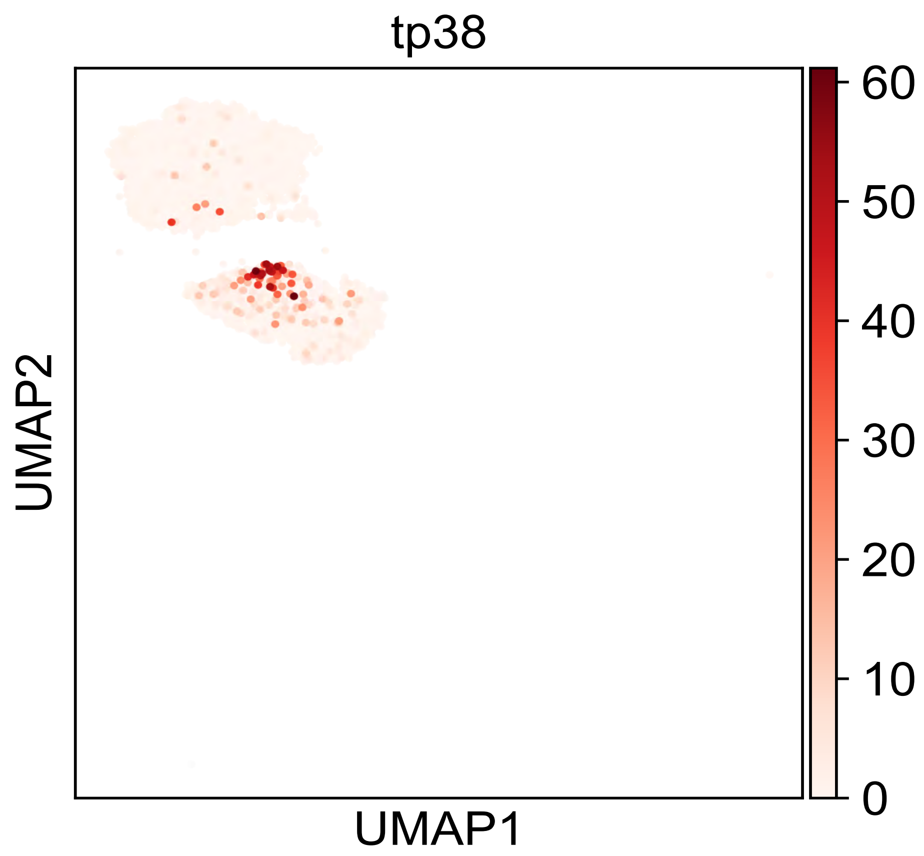

Cell Type

Endo_3

Endo_6

Top 20 genes

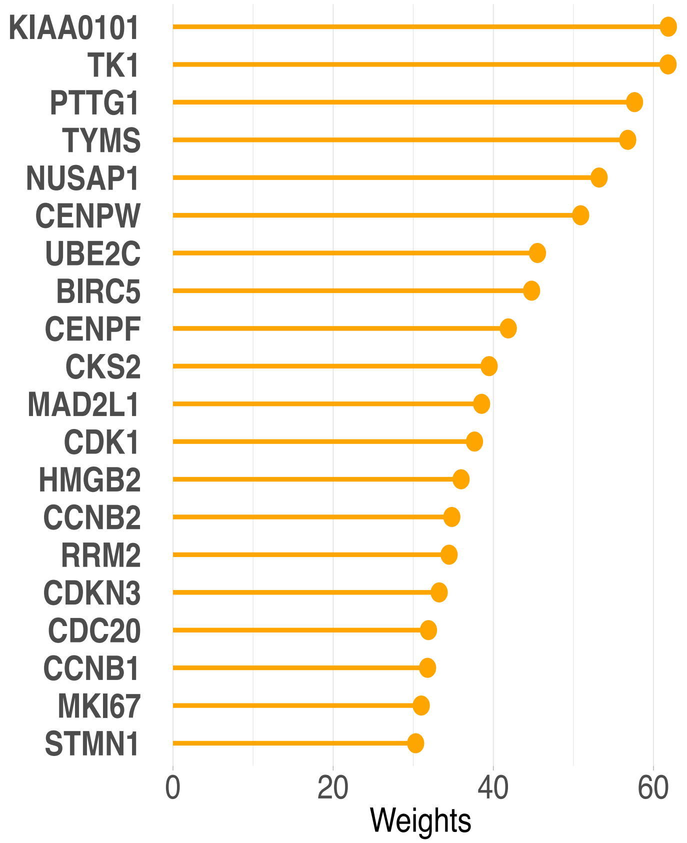

Endo GEM 39

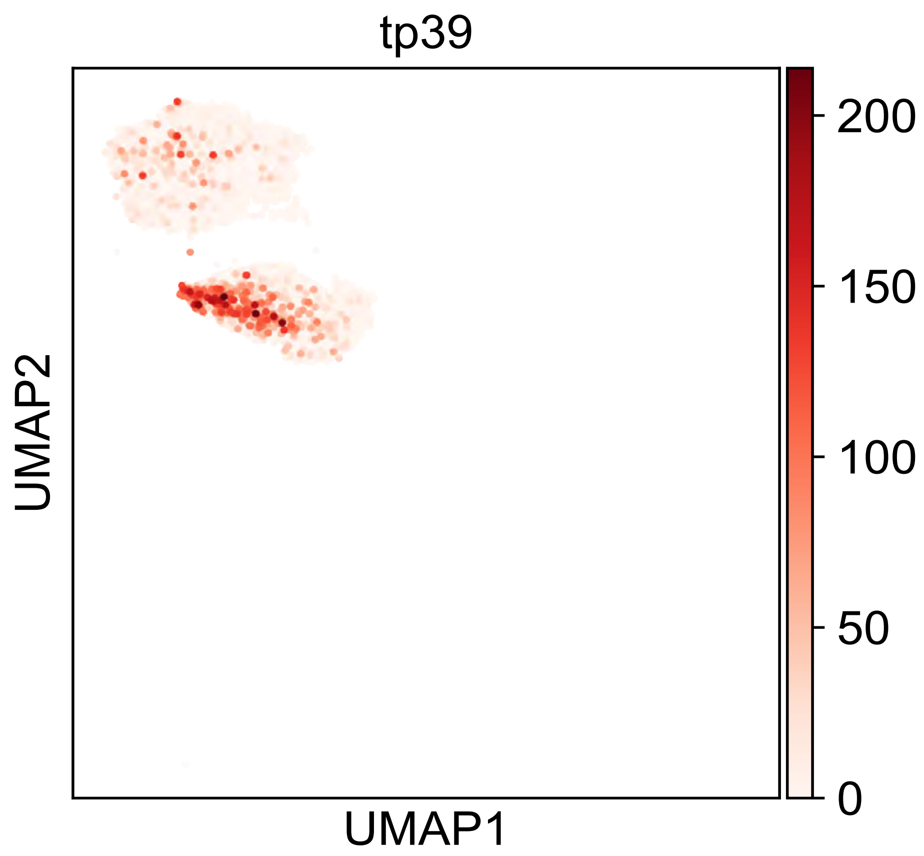

Cell Type

Endo_2

Endo_3

Top 20 genes

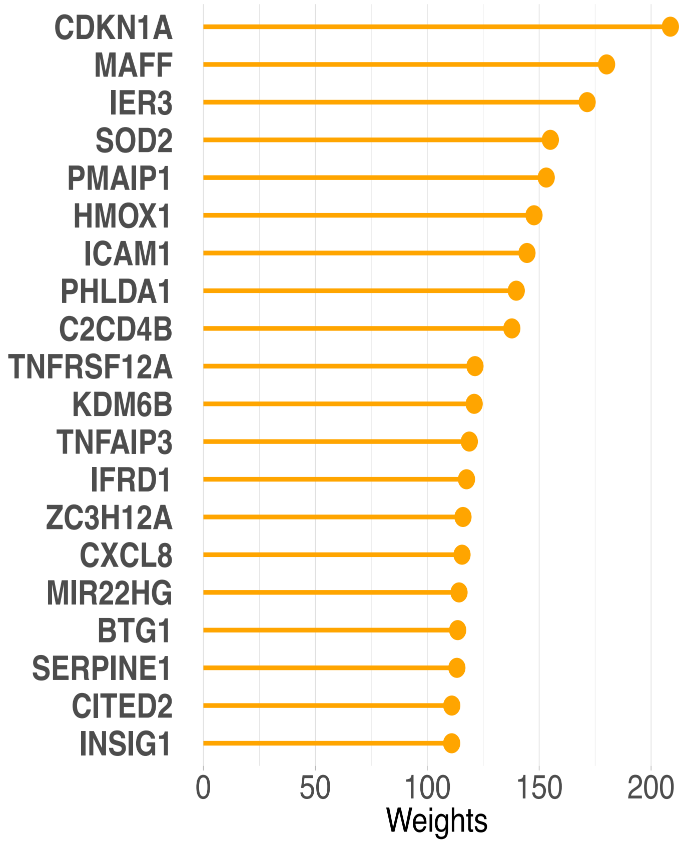

Endo GEM 42

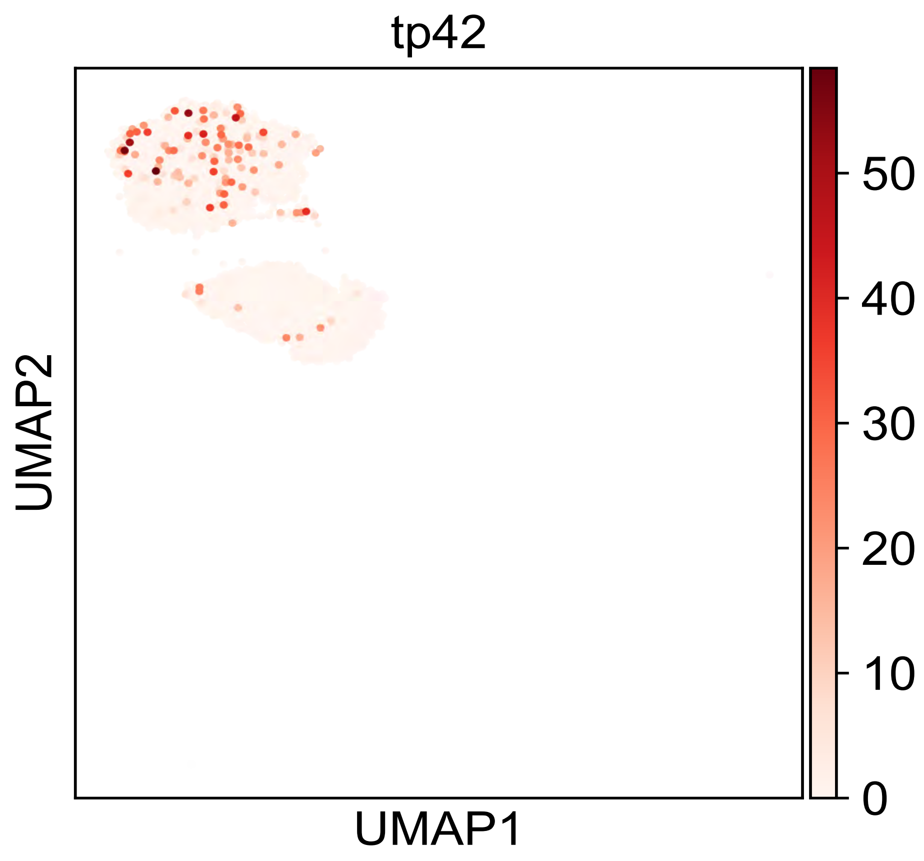

Cell Type

Endo_1

Endo_4

Top 20 genes

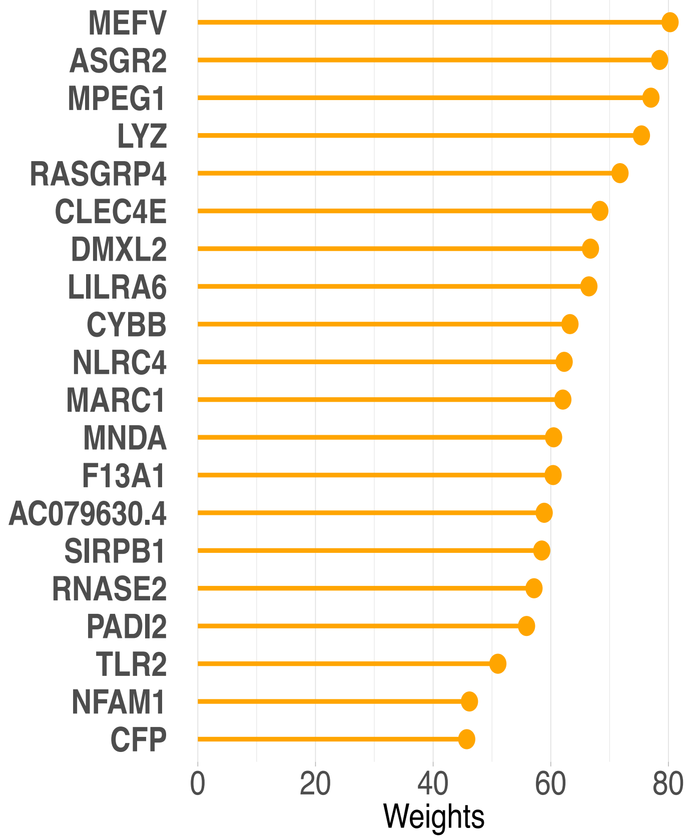

Endo GEM 50

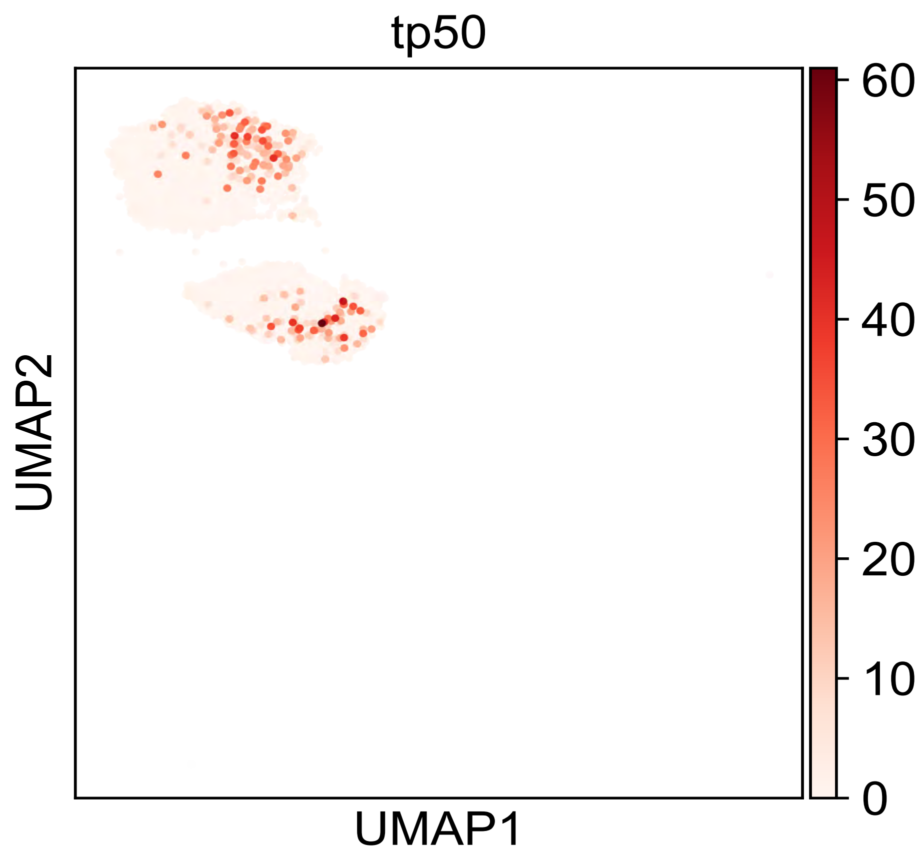

Cell Type

Endo_3

Endo_4

Top 20 genes

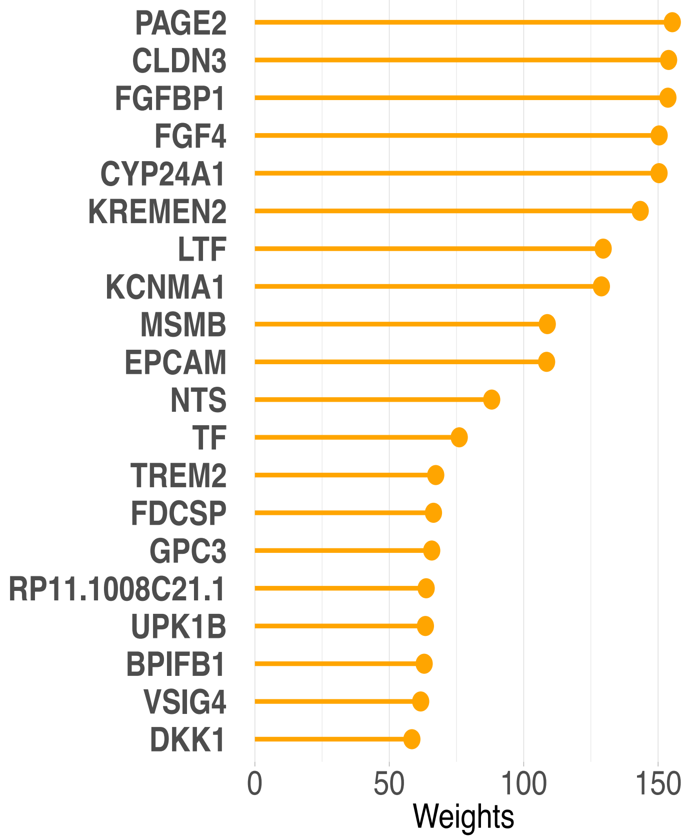

Endo GEM 51

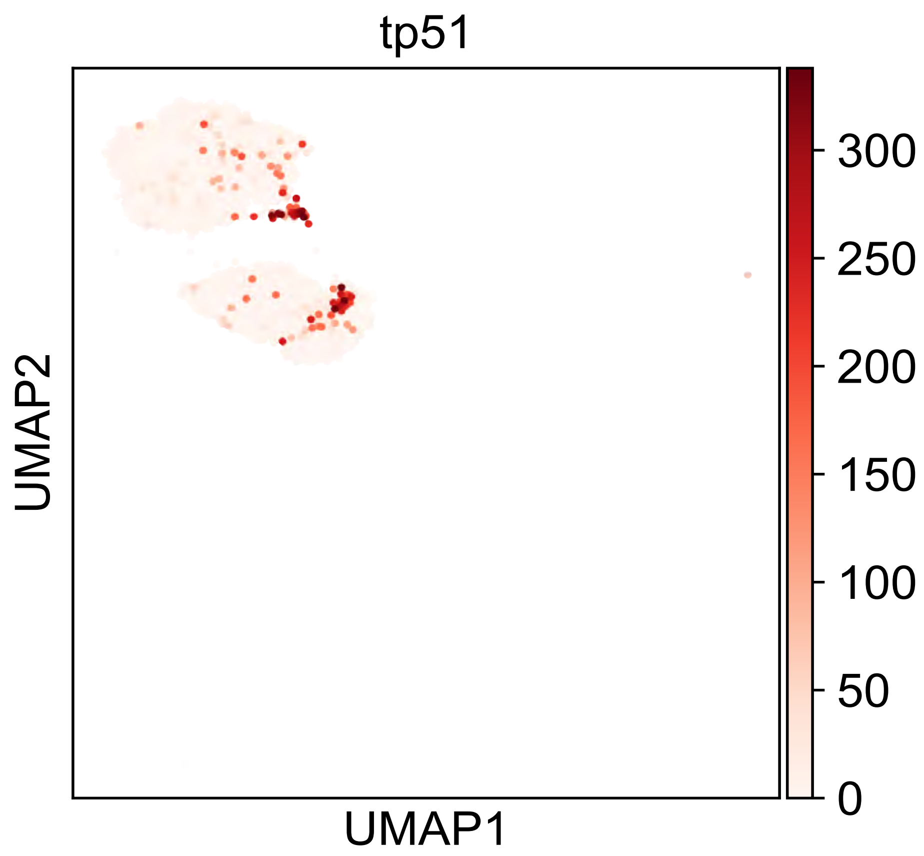

Cell Type

Endo_3

Endo_4

Endo_6

Top 20 genes

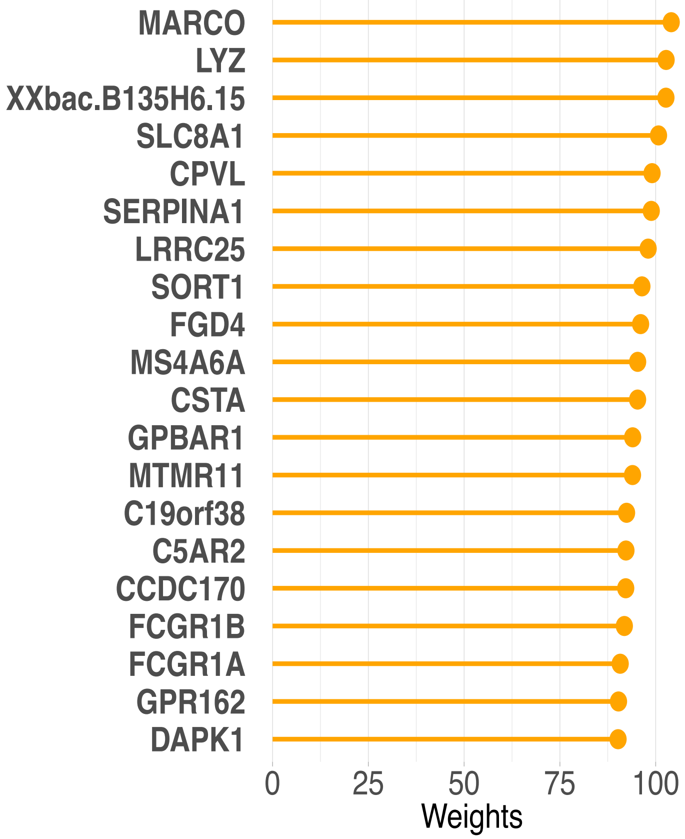

Endo GEM 59

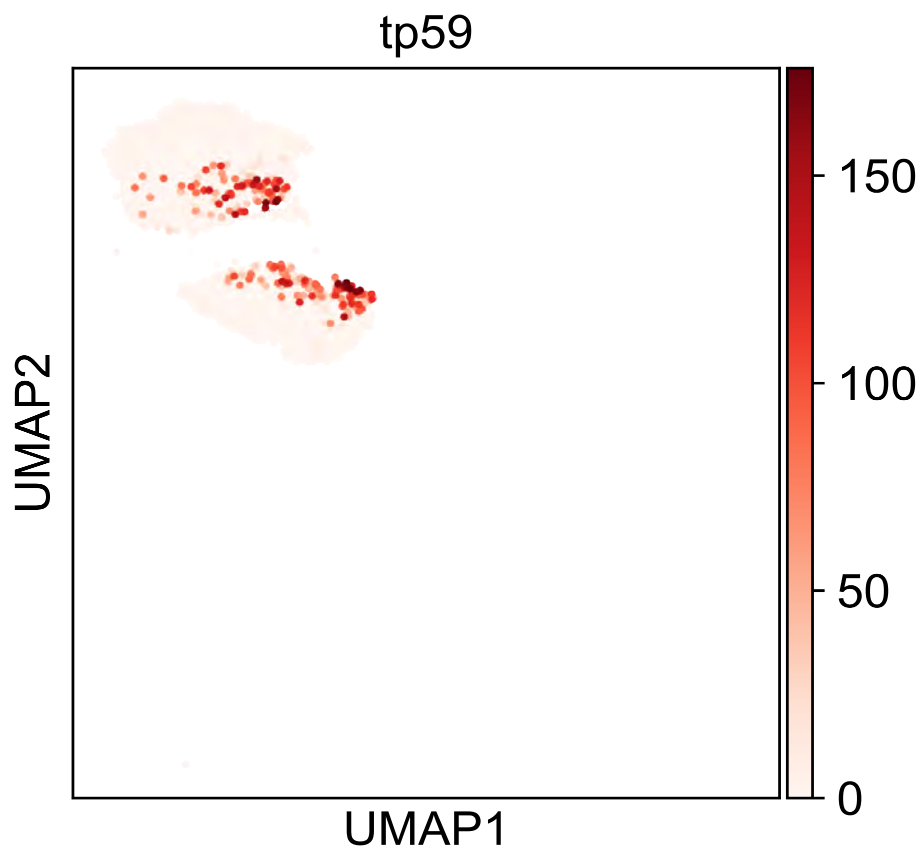

Cell Type

Endo_3

Endo_5

Endo_6

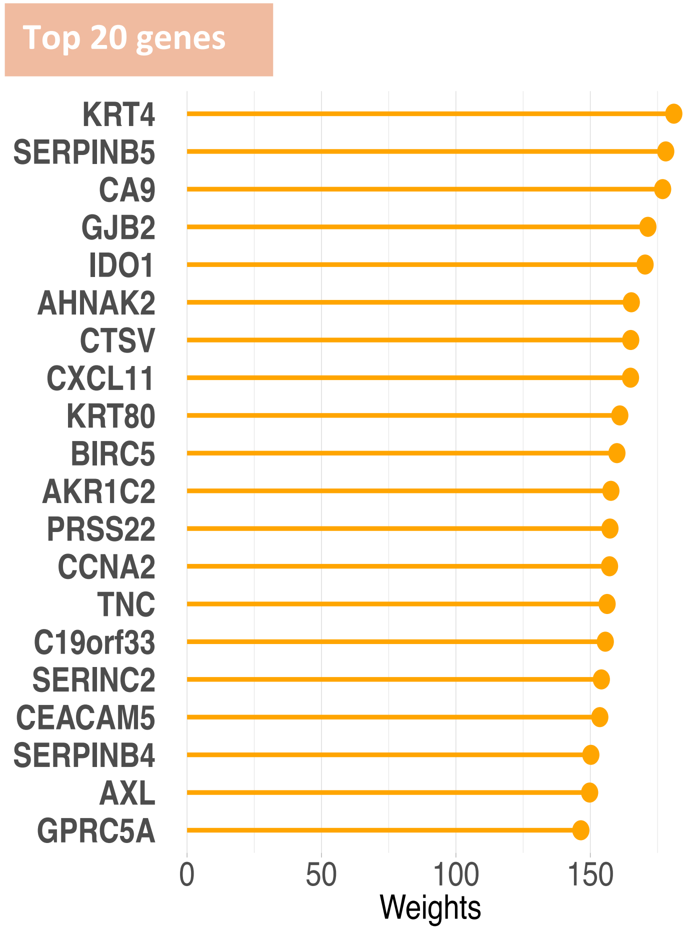

Fib GEM 1

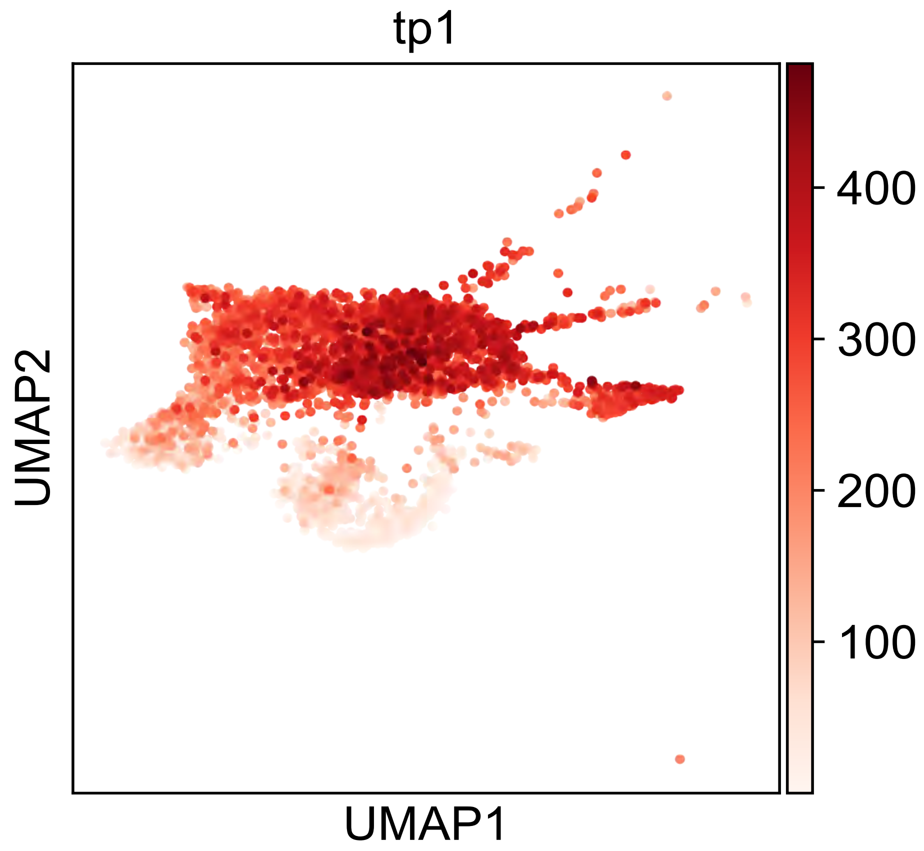

Cell Type

Fib_1

Fib_2

Fib_3

Top 20 genes

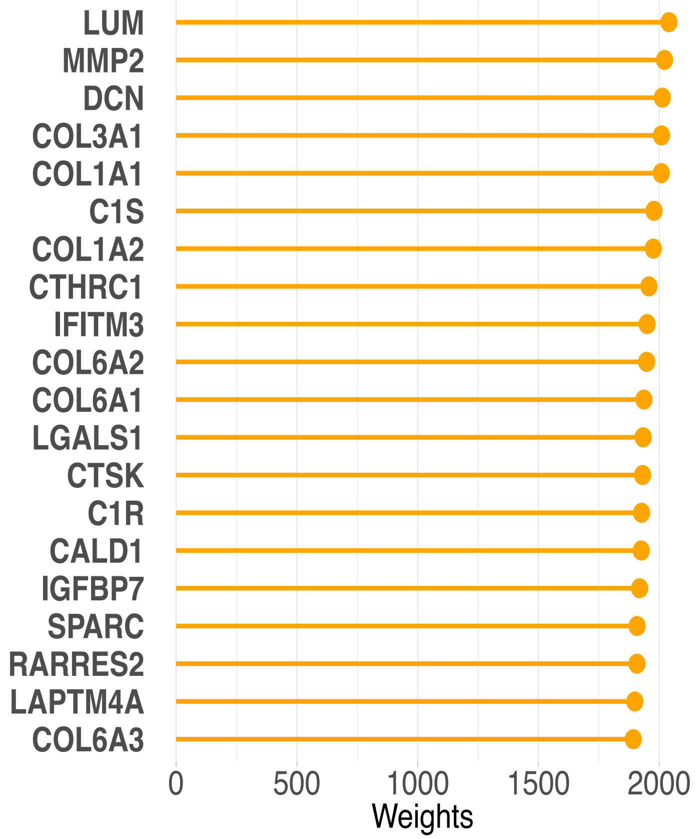

Fib GEM 2

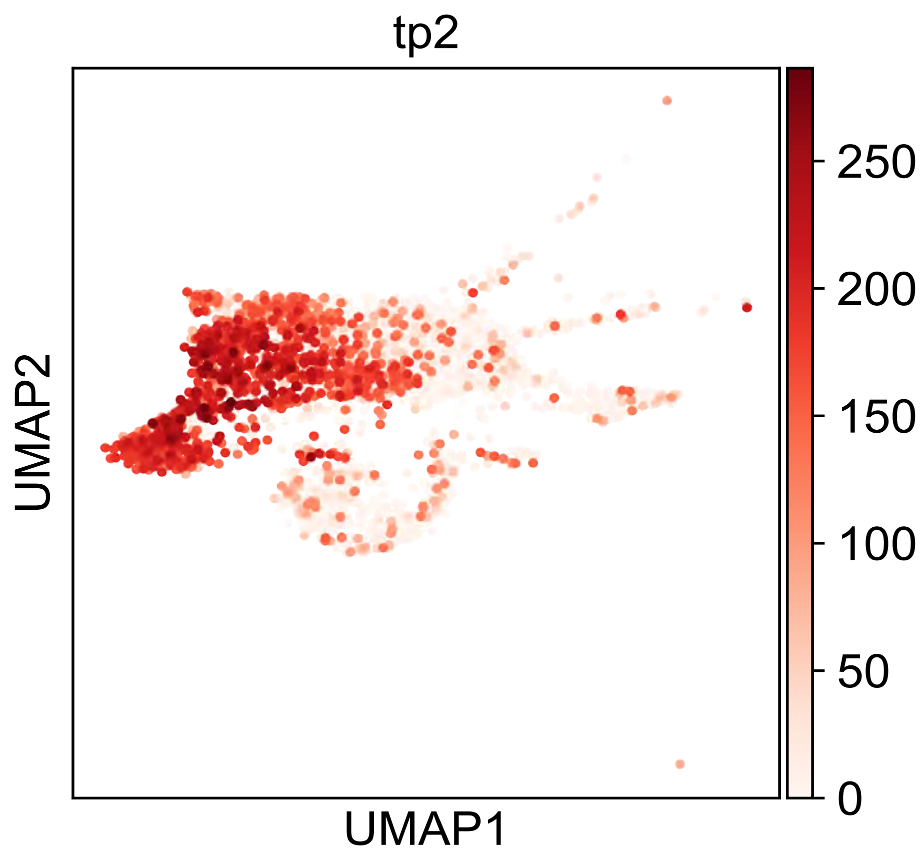

Cell Type

Fib_5

Fib_3

Top 20 genes

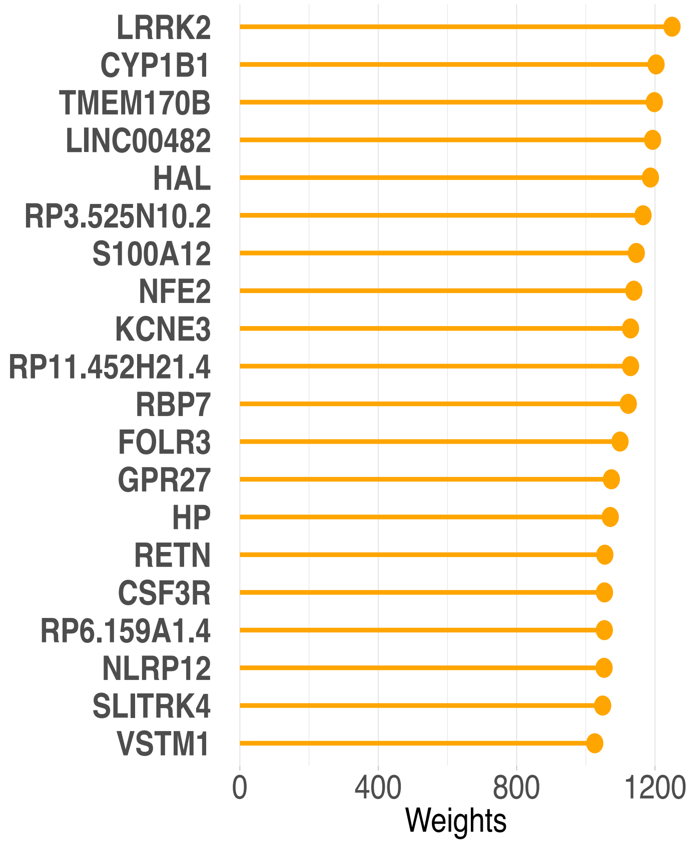

Fib GEM 3

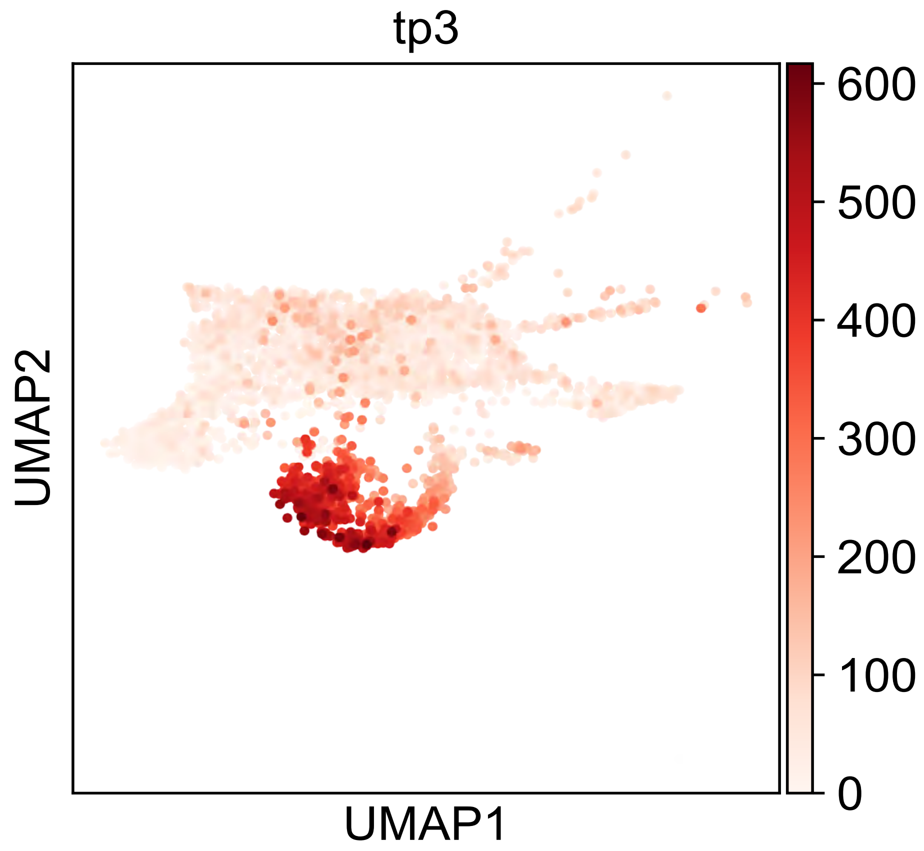

Cell Type

Fib_4

Fib_6

Top 20 genes

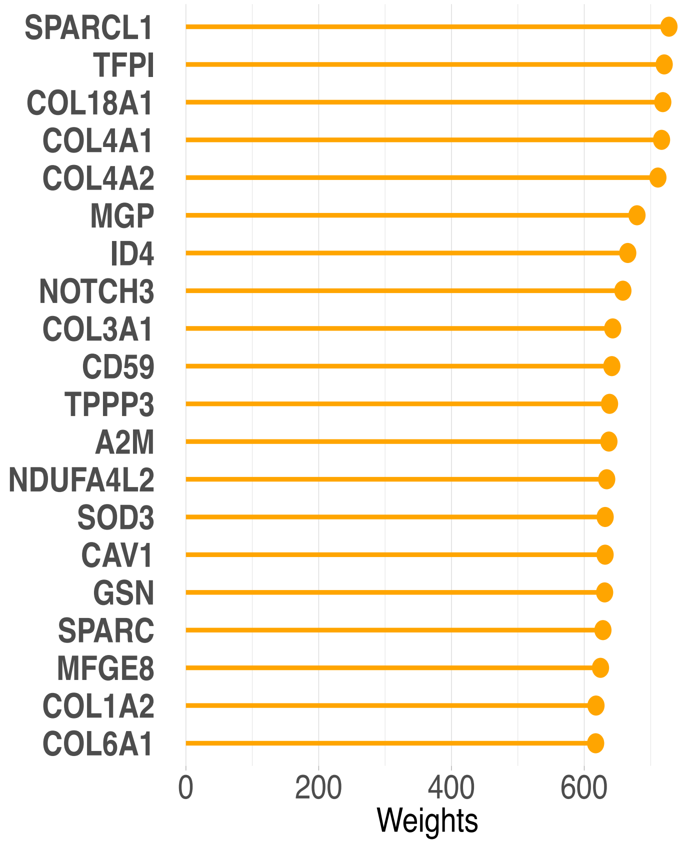

Fib GEM 4

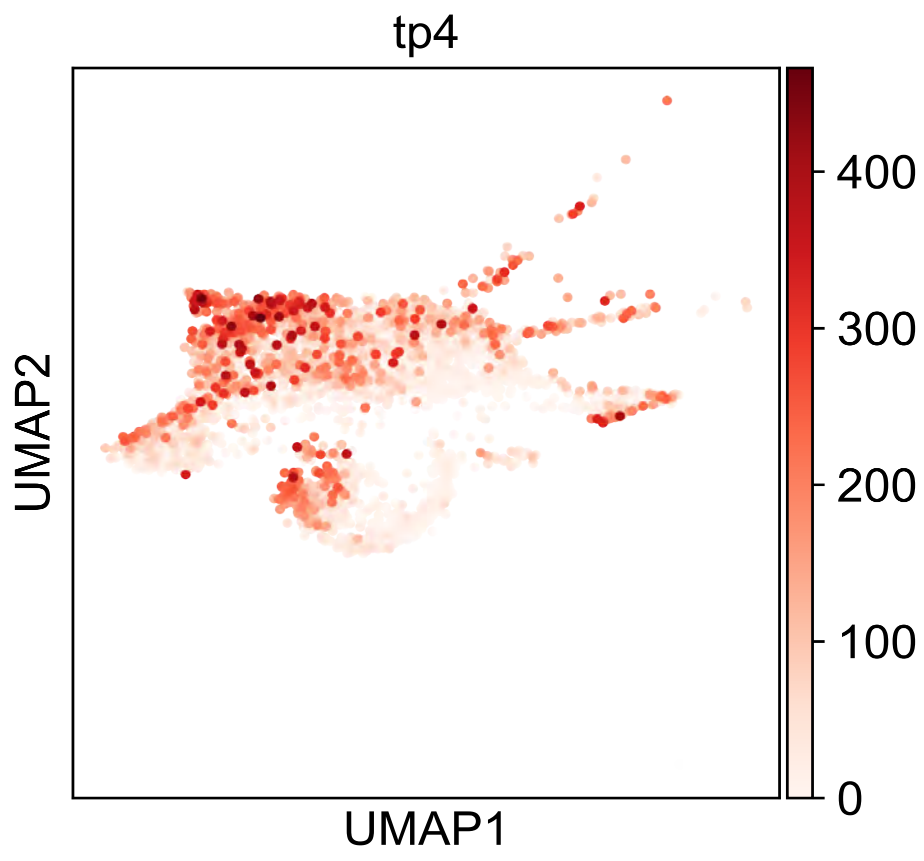

Cell Type

Fib_5

Fib_1

Fib_6

Fib_2

Fib_3

Top 20 genes

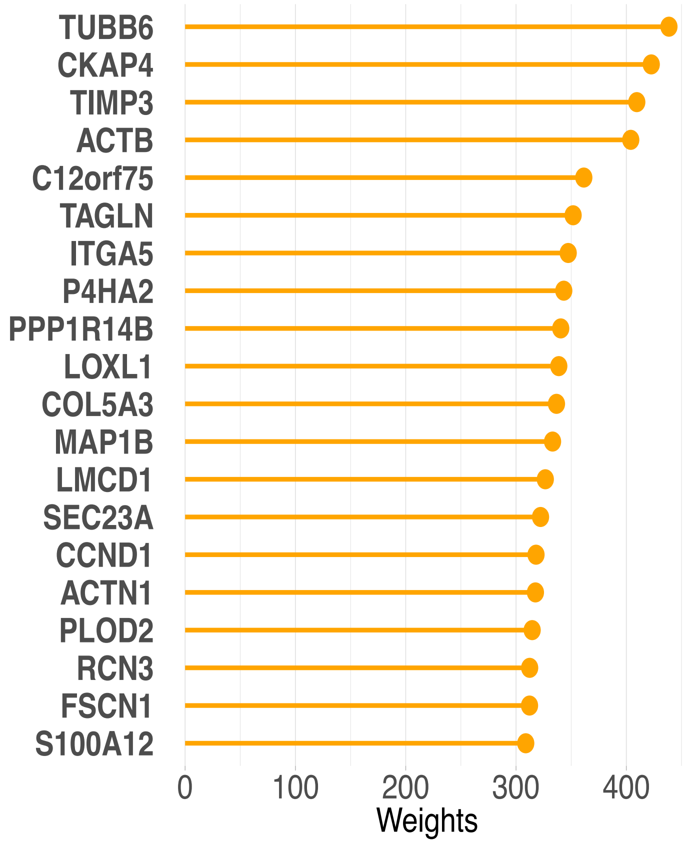

Fib GEM 5

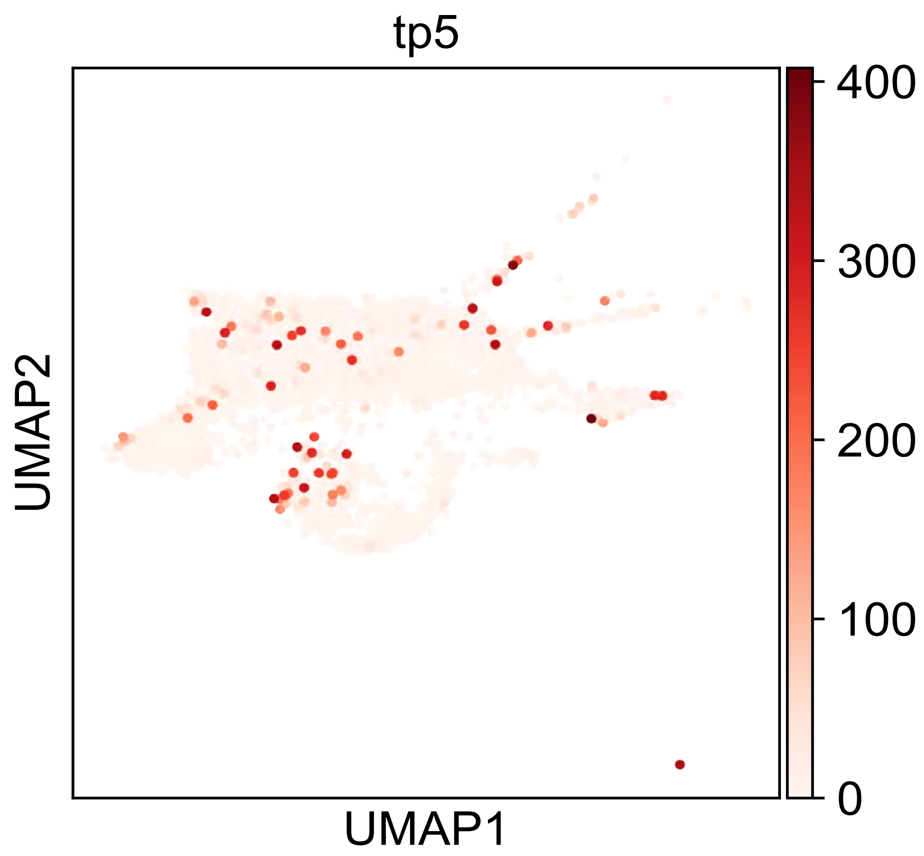

Cell Type

Fib_2

Fib_6

Top 20 genes

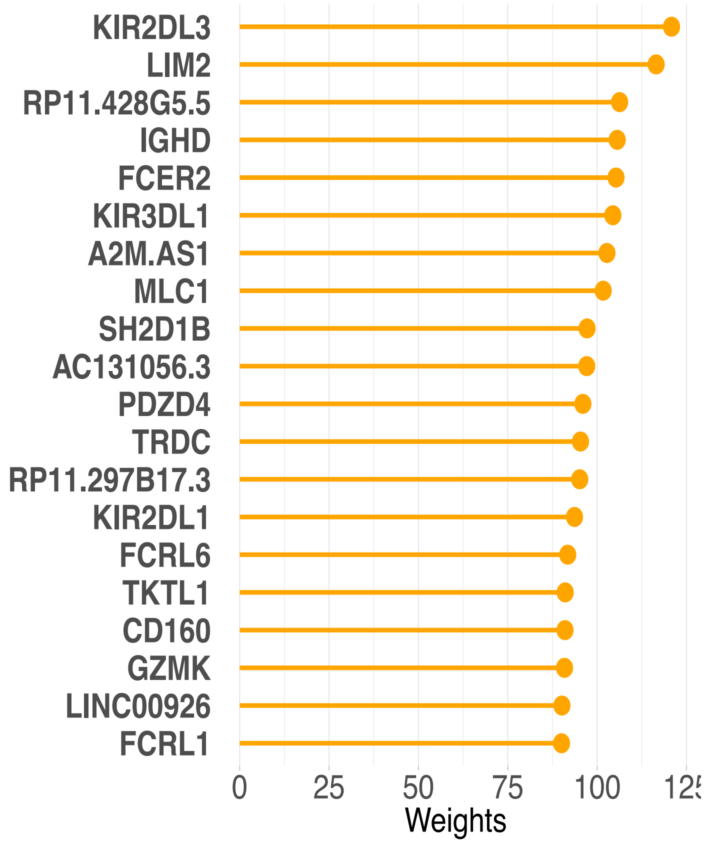

Fib GEM 7

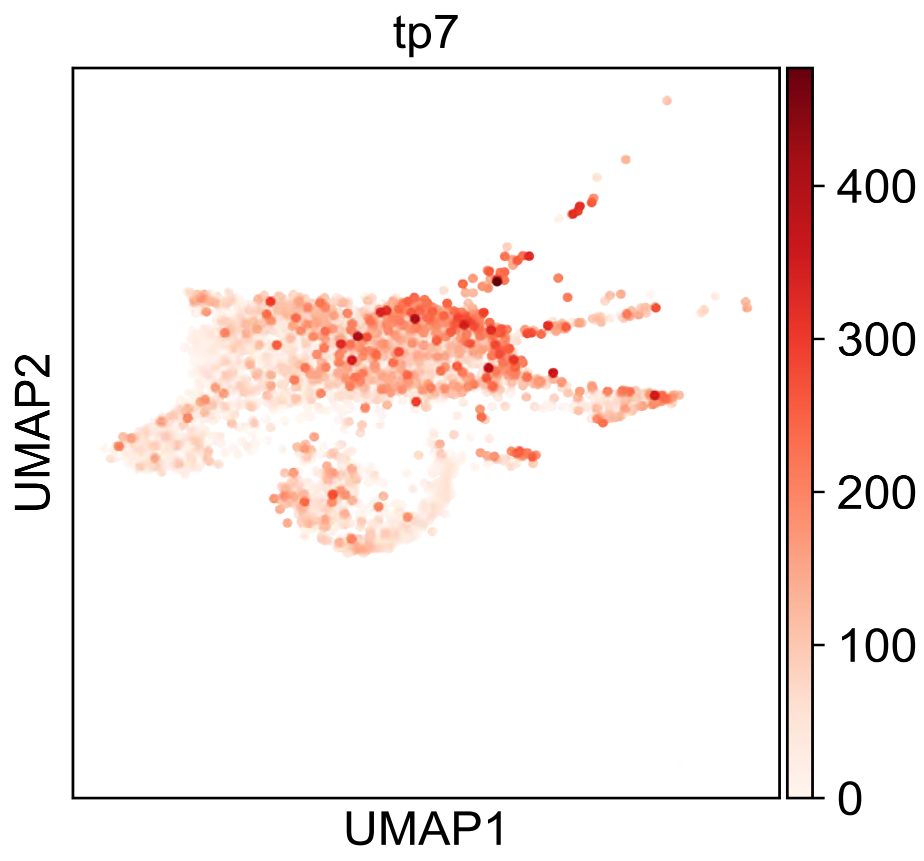

Cell Type

Fib_1

Fib_2

Fib_6

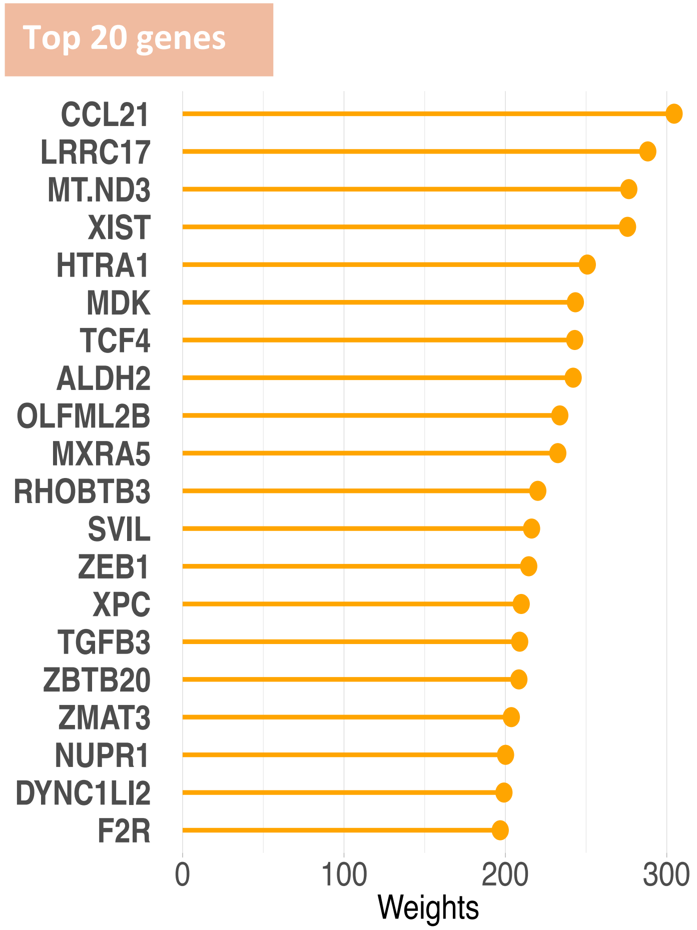

Fib GEM 9

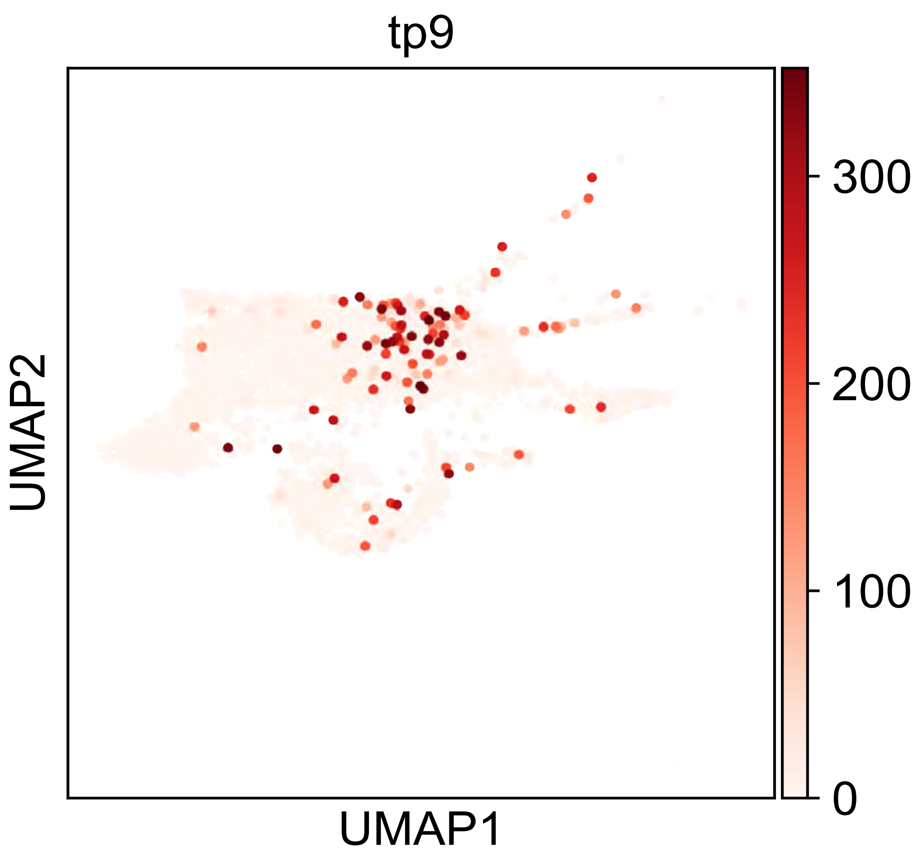

Cell Type

Fib_1

Fib_4

Top 20 genes

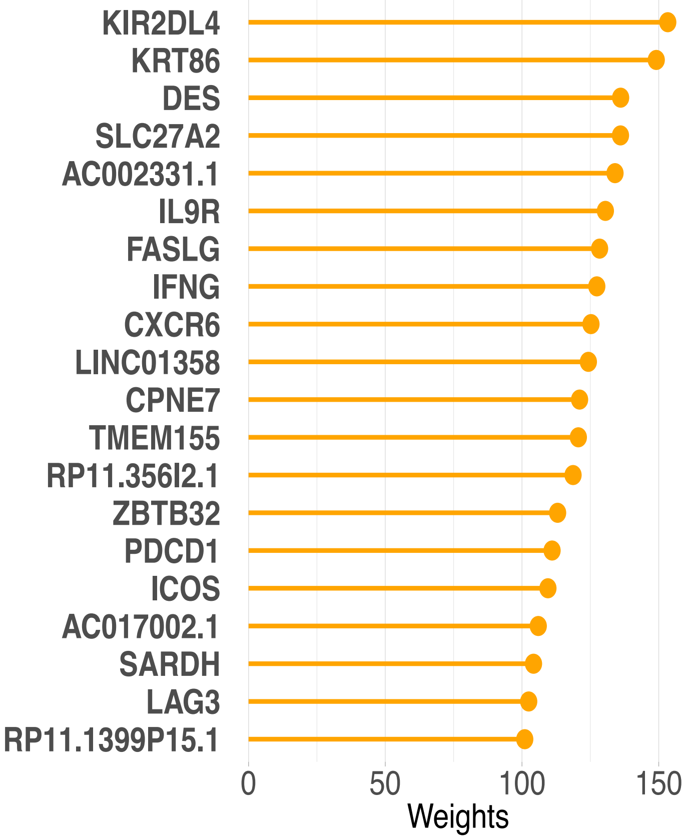

Fib GEM 10

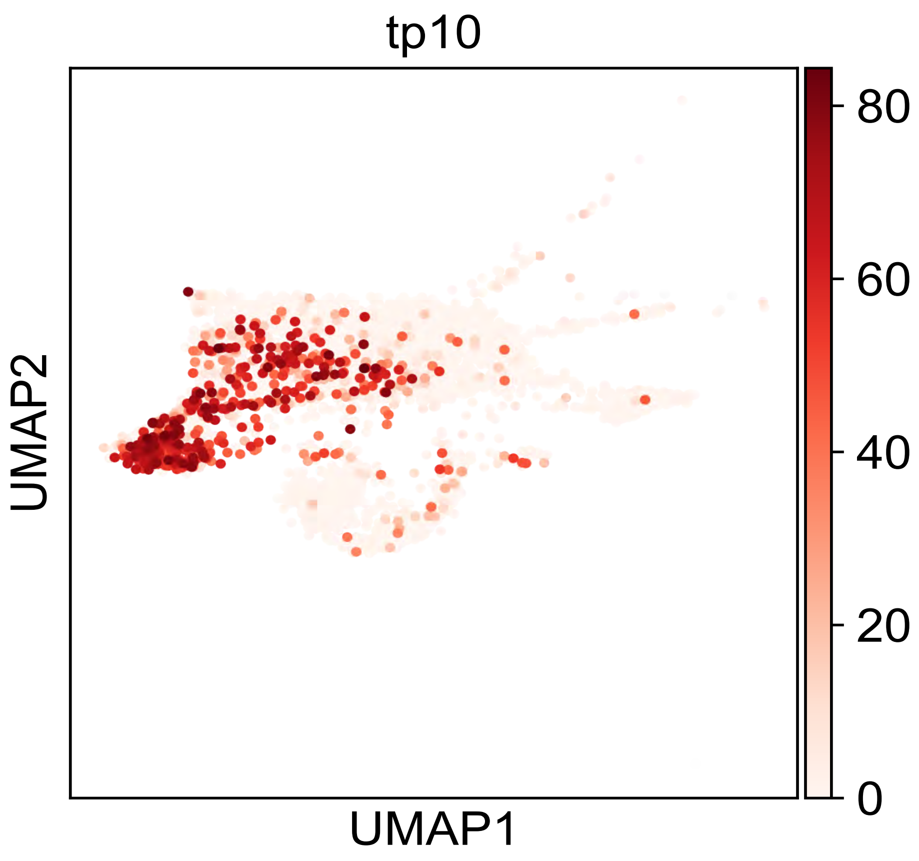

Cell Type

Fib_5

Fib_1

Fib_3

Top 20 genes

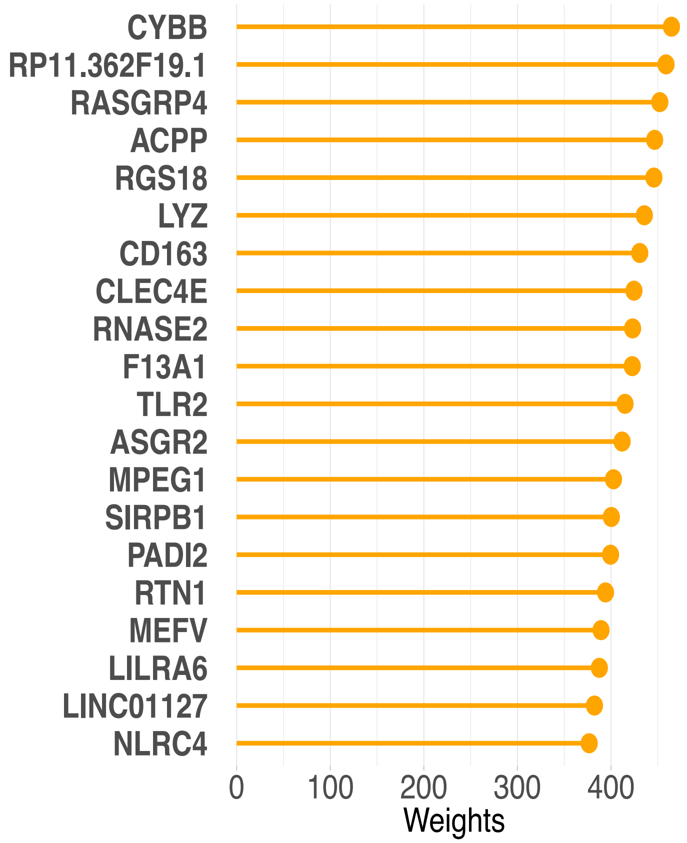

Fib GEM 11

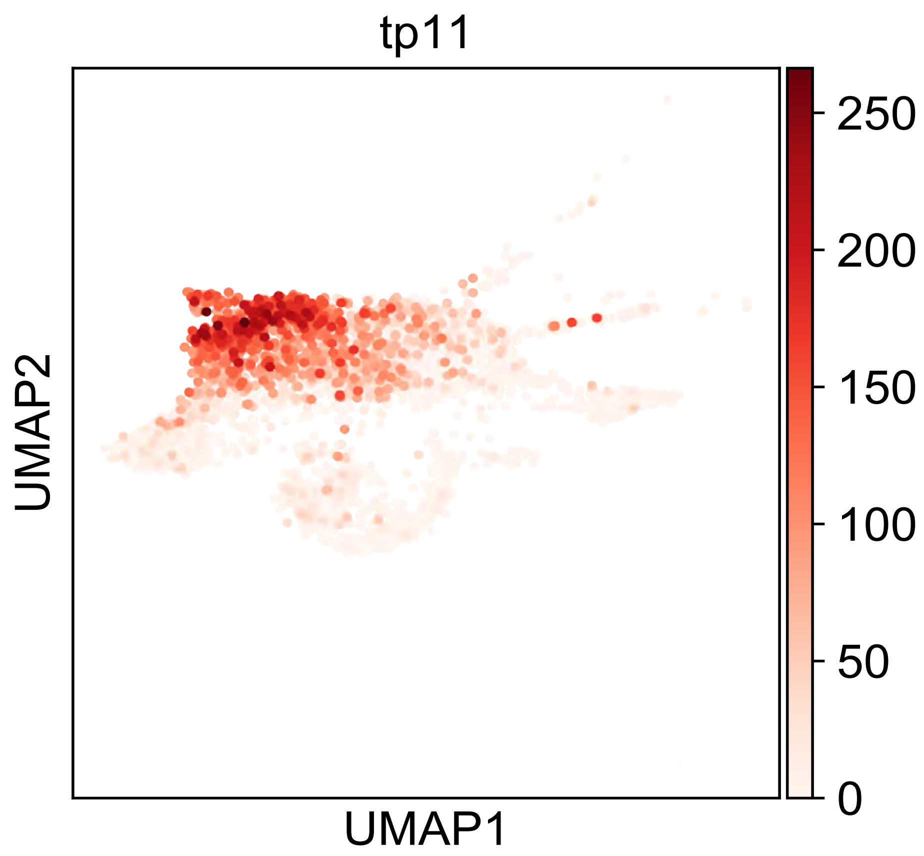

Cell Type

Fib_1

Fib_2

Fib_3

Top 20 genes

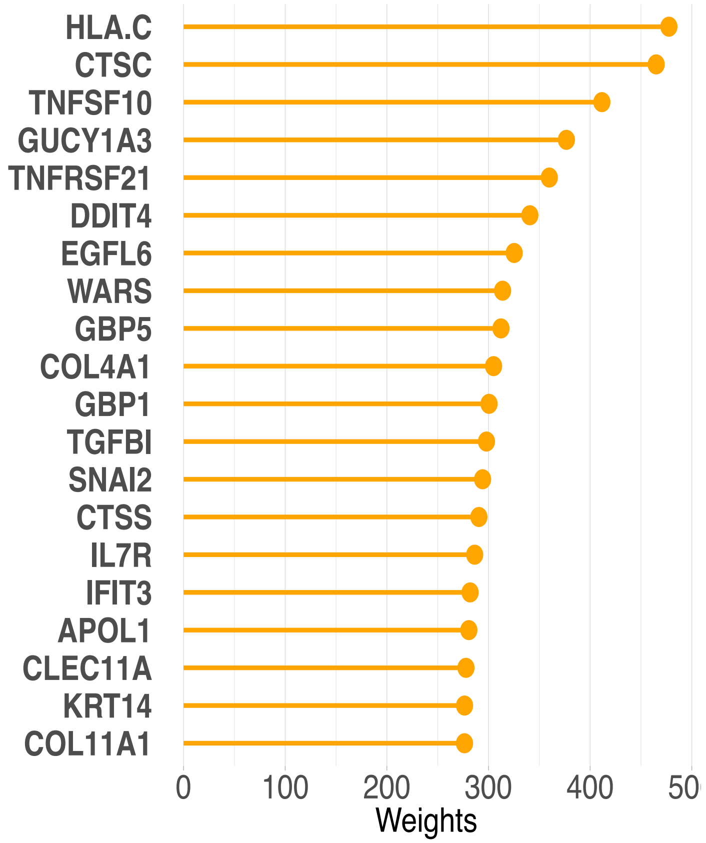

Fib GEM 12

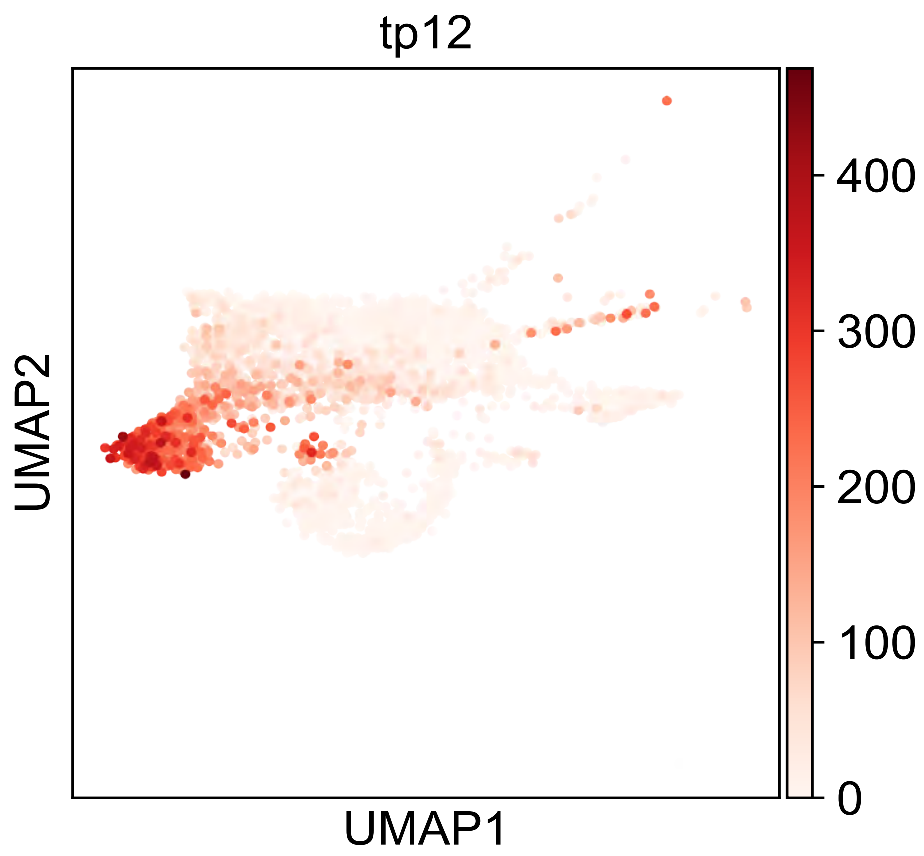

Cell Type

Fib_5

Fib_1

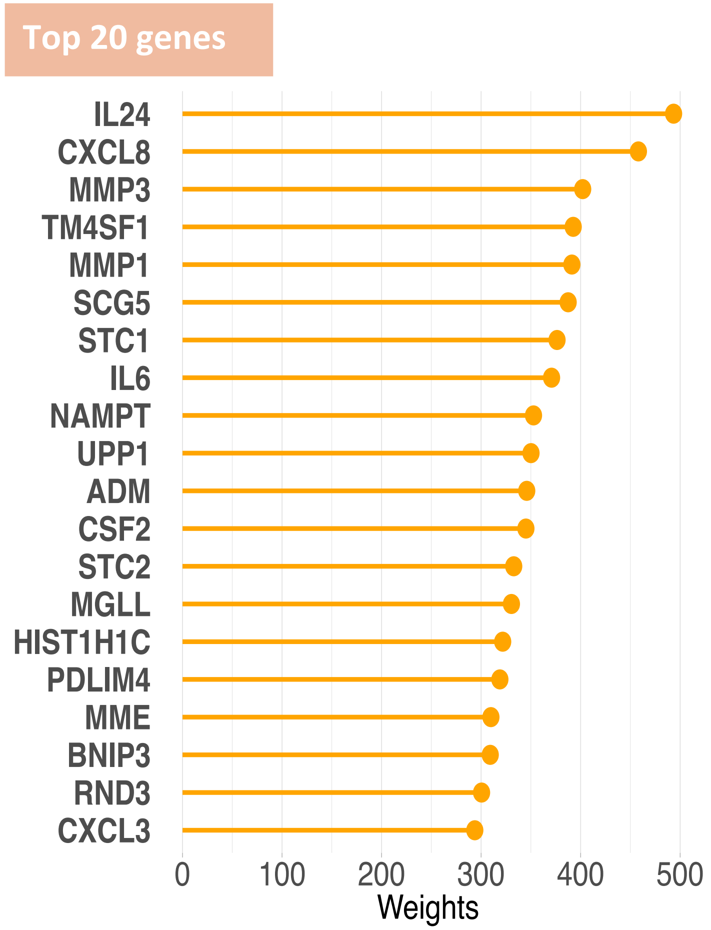

Fib GEM 14

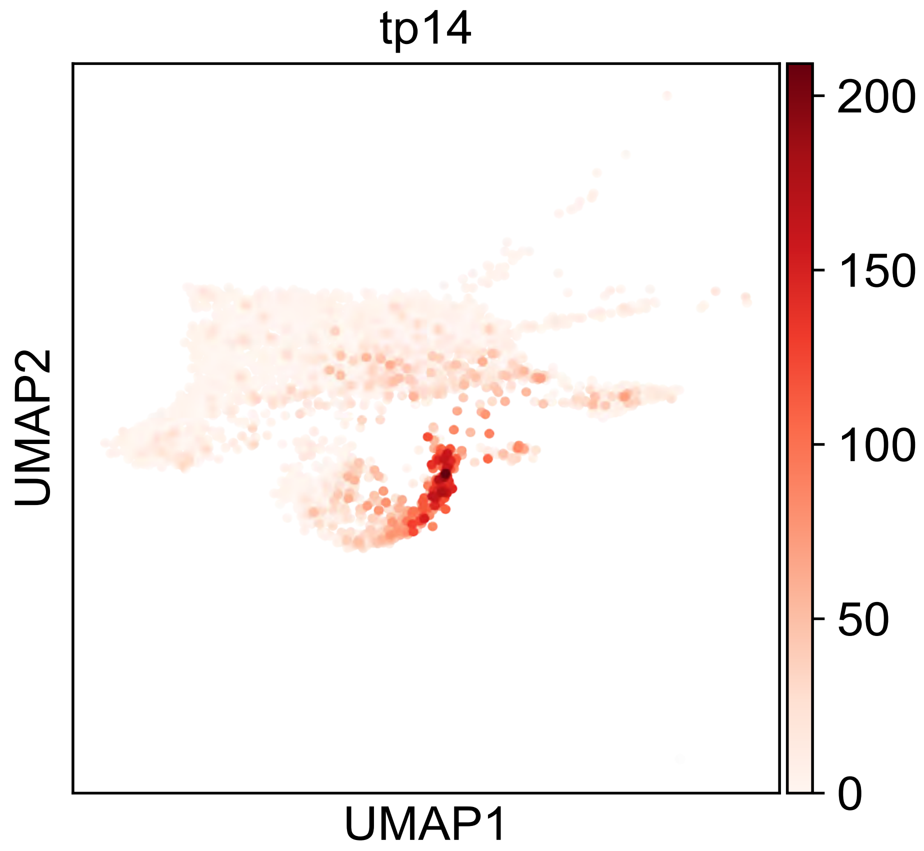

Cell Type

Fib_4

Top 20 genes

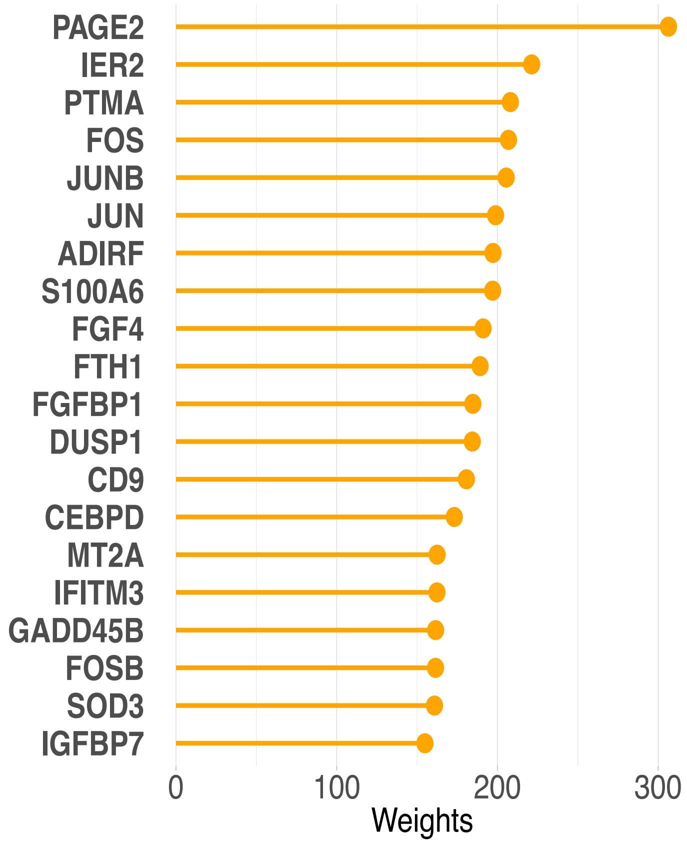

Fib GEM 16

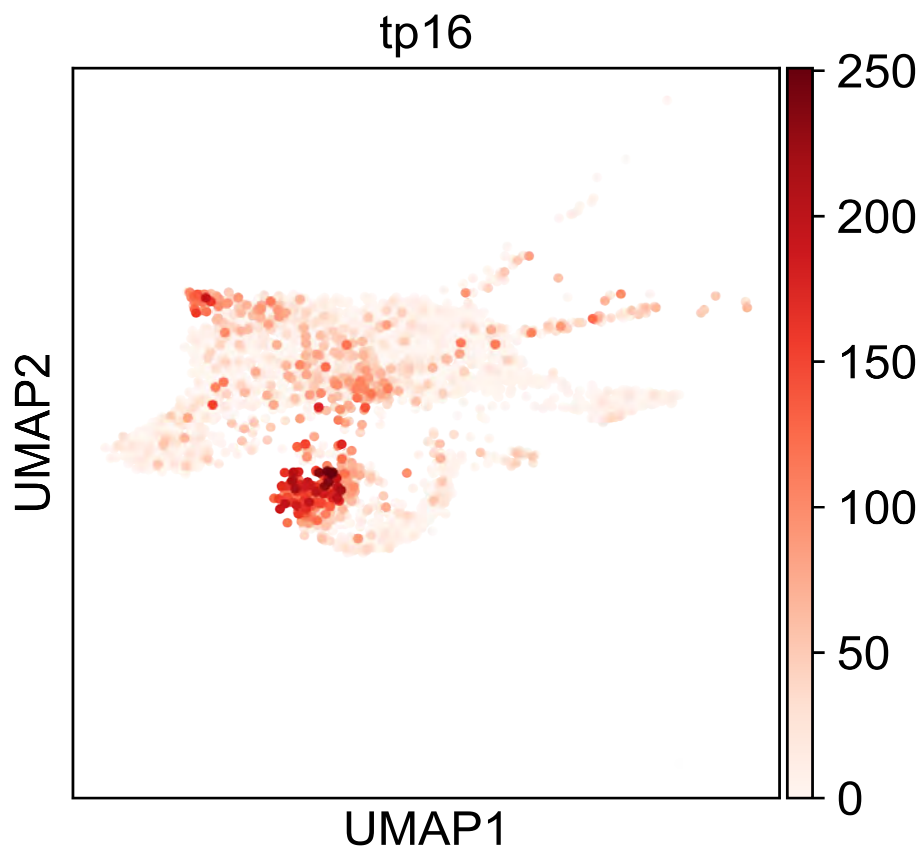

Cell Type

Fib_6

Fib_2

Top 20 genes

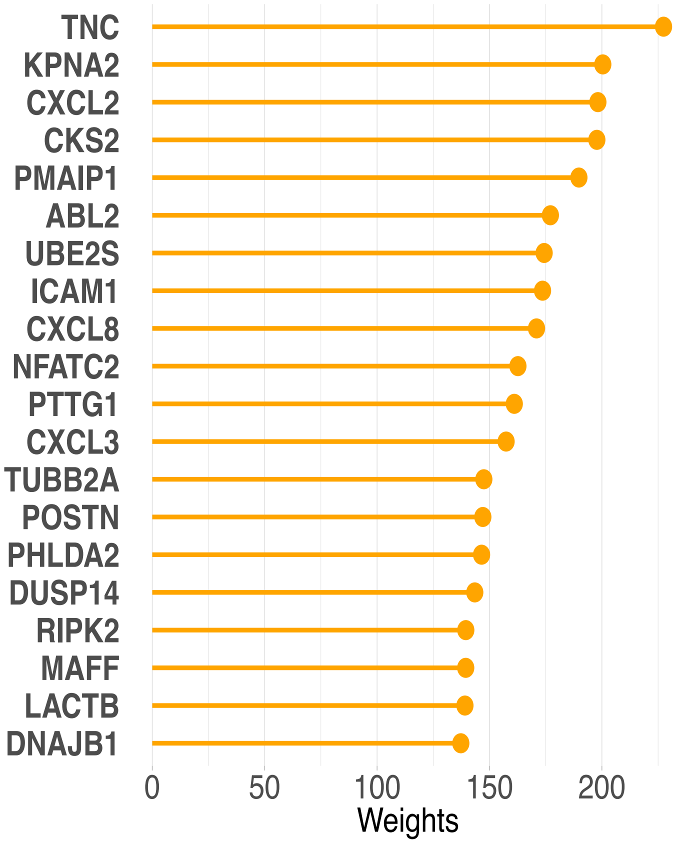

Fib GEM 19

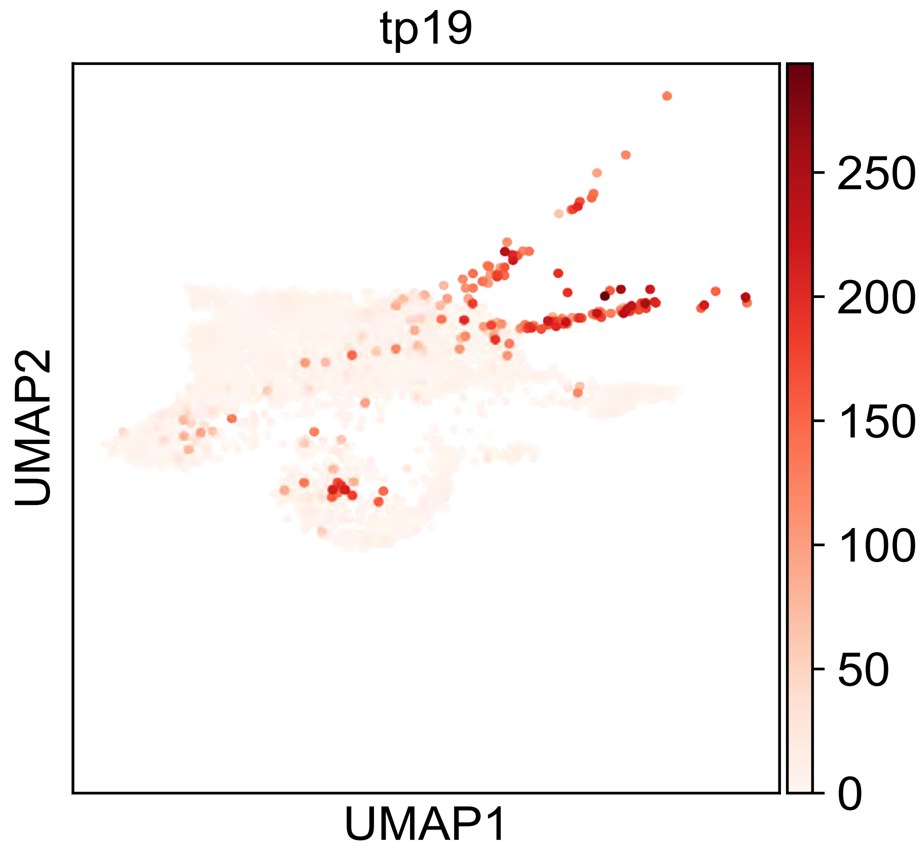

Cell Type

Fib_1

Fib_2

Fib_4

Top 20 genes

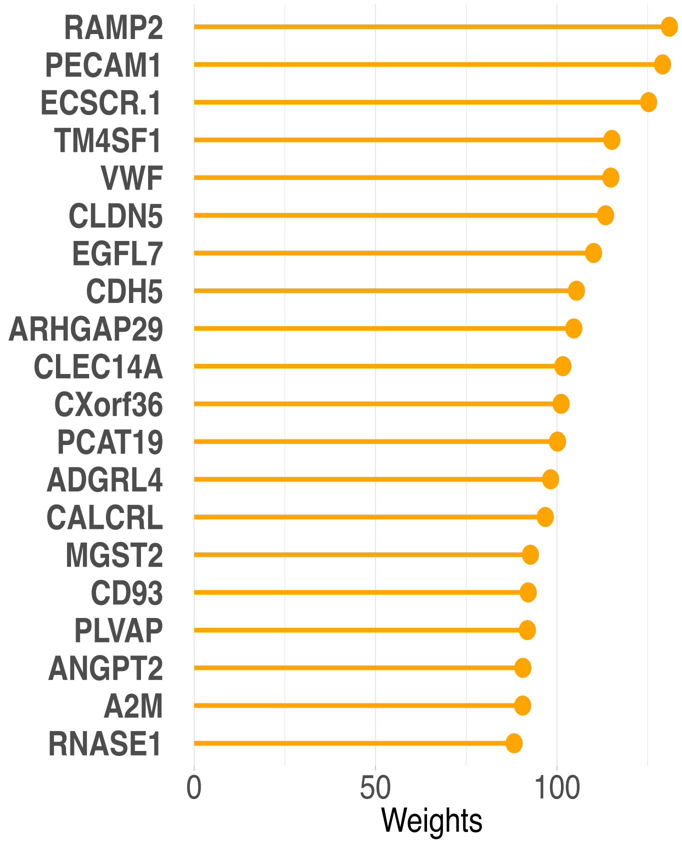

Fib GEM 30

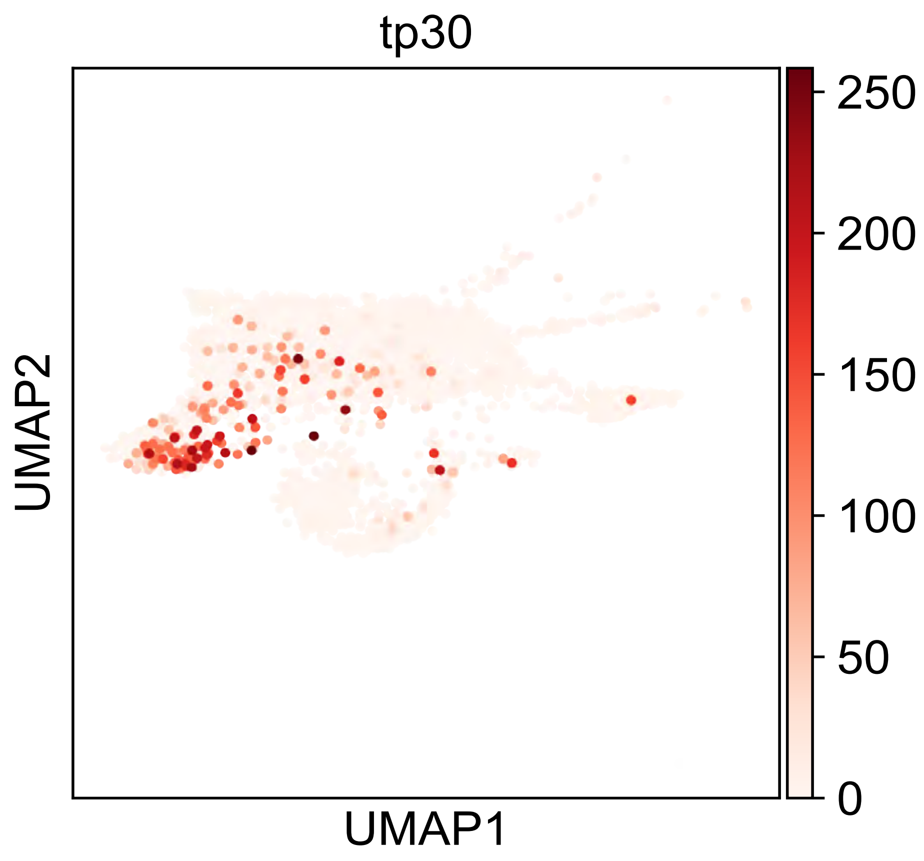

Cell Type

Fib_5

Fib_3

Top 20 genes

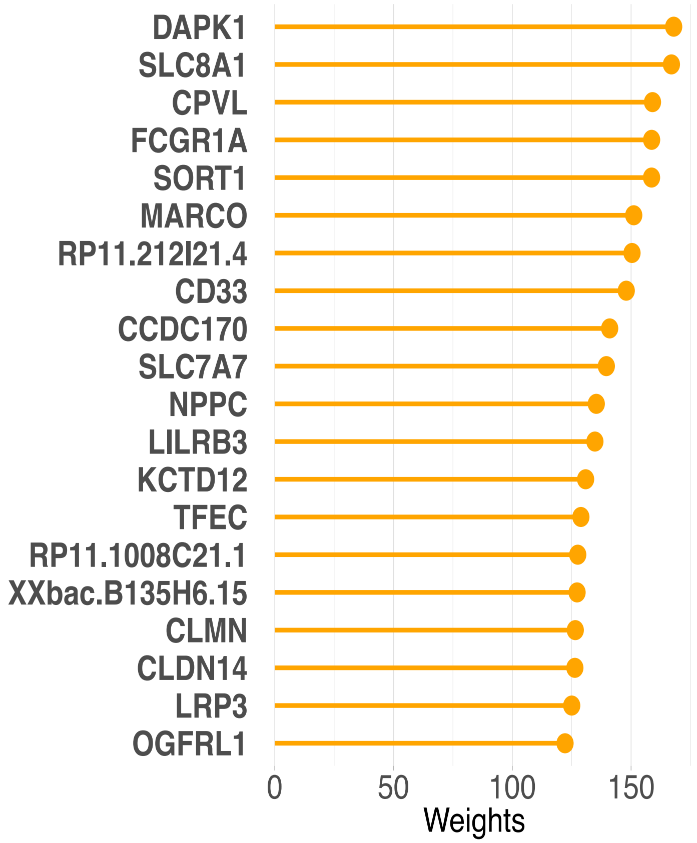

Fib GEM 33

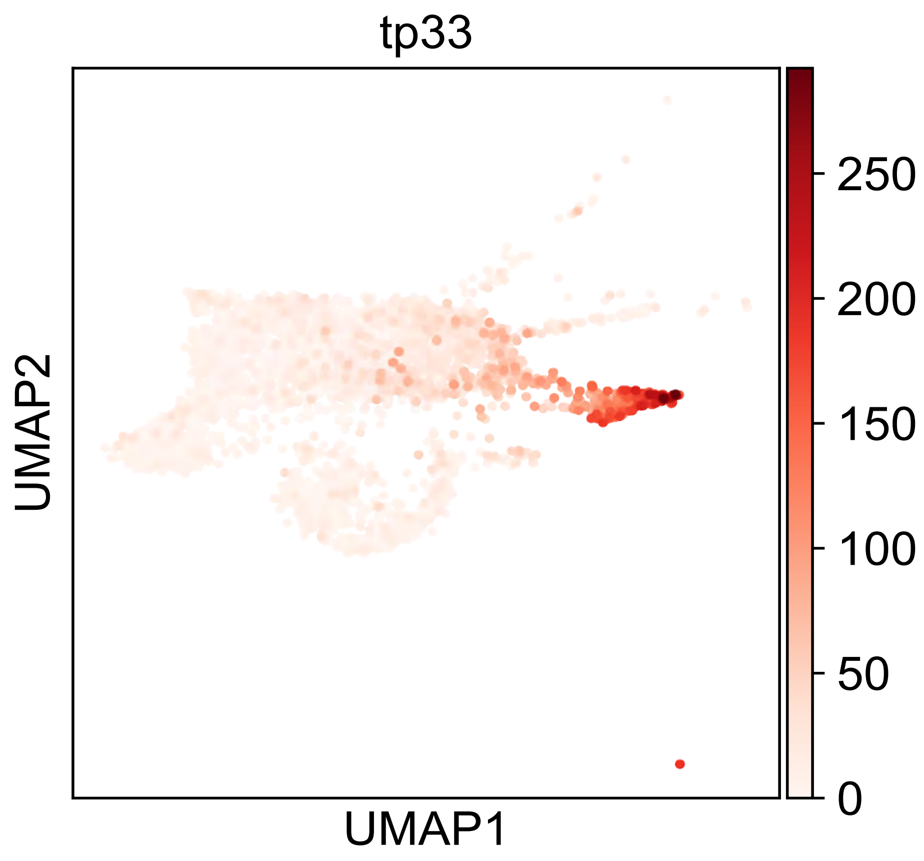

Cell Type

Fib_1

Fib_2

Top 20 genes

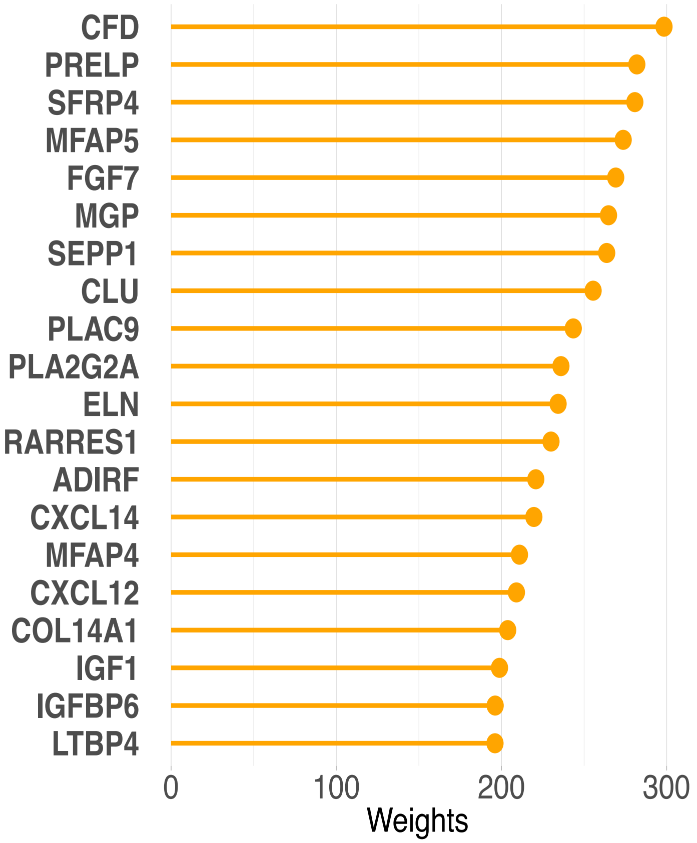

Fib GEM 34

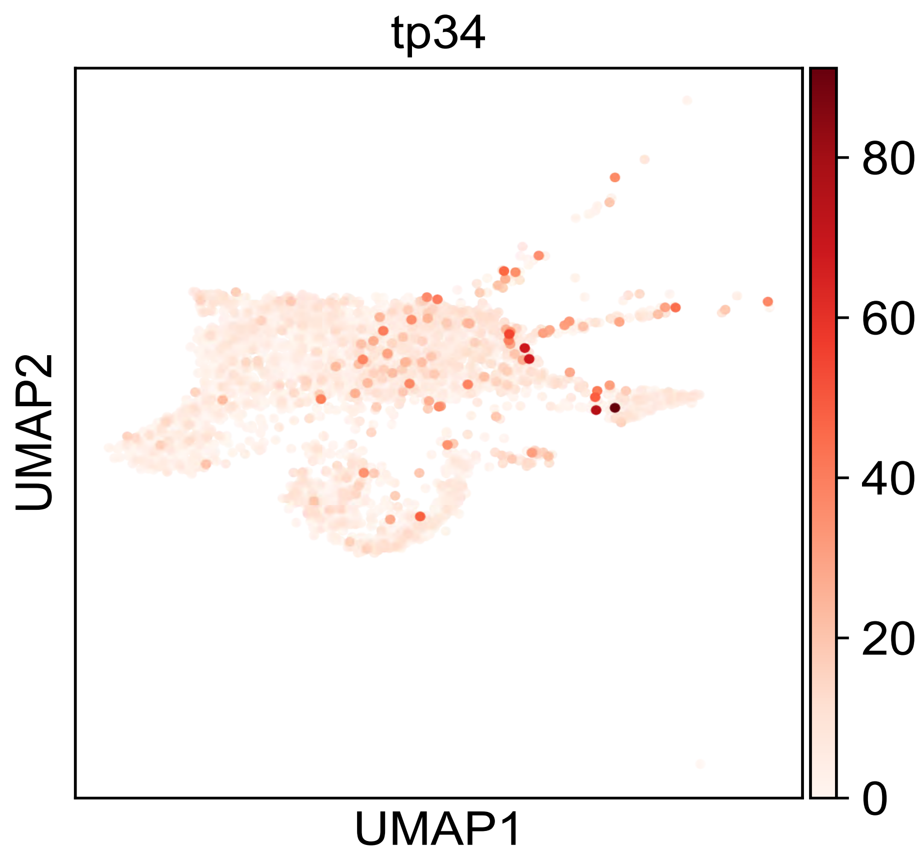

Cell Type

Fib_1

Fib_2

Top 20 genes

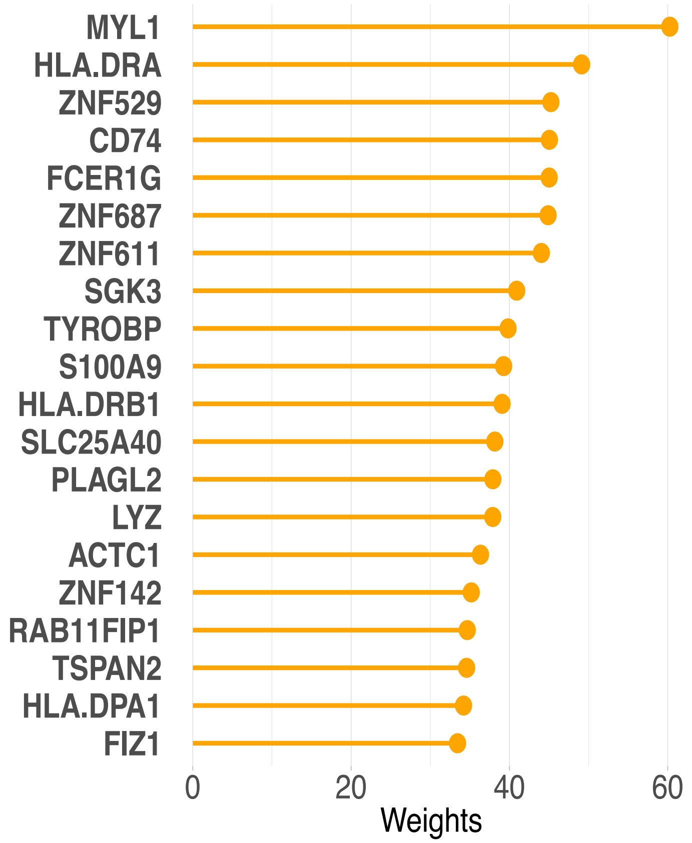

Fib GEM 35

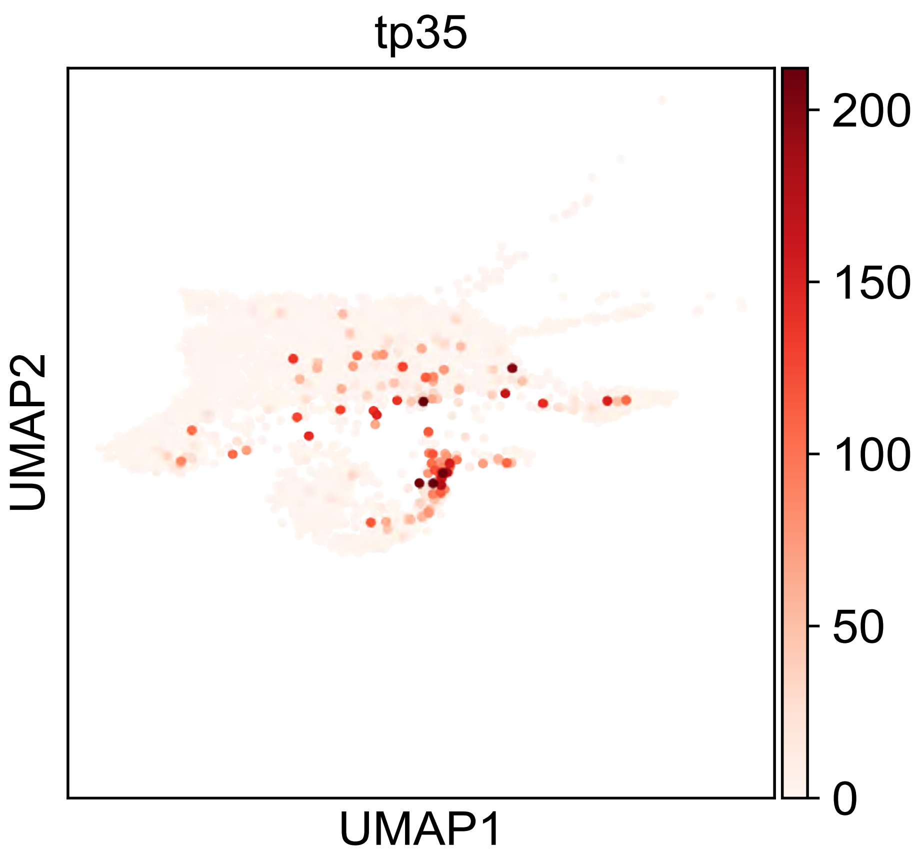

Cell Type

Fib_4

Fib_1

Top 20 genes

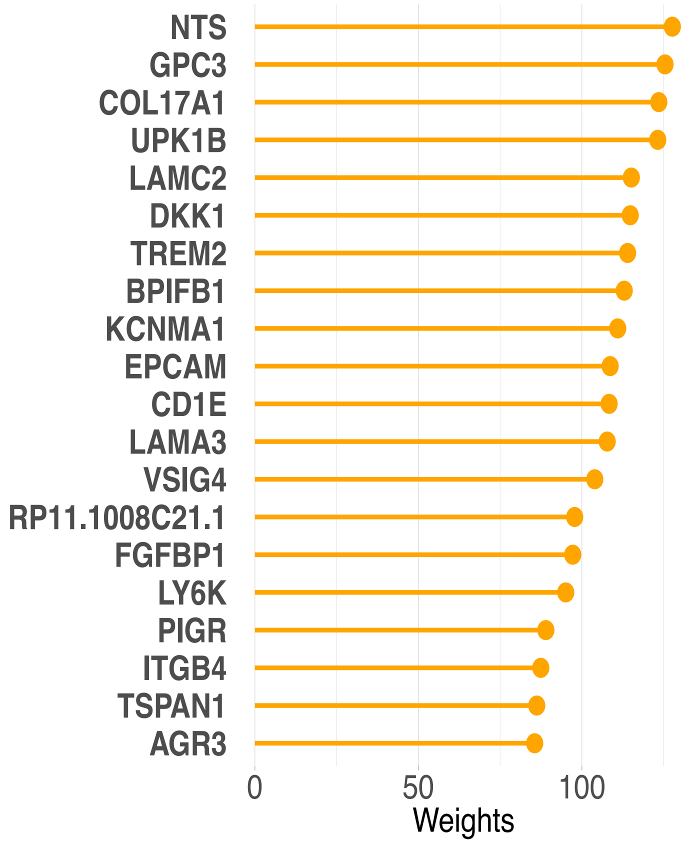

Fib GEM 37

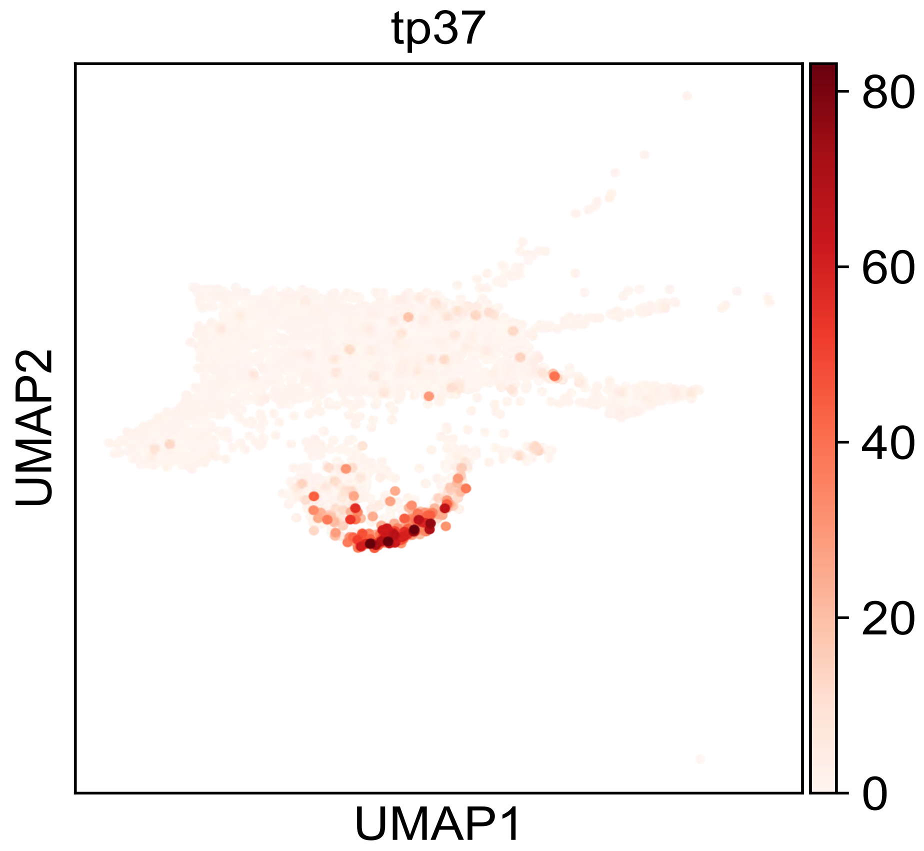

Cell Type

Fib_6

Top 20 genes

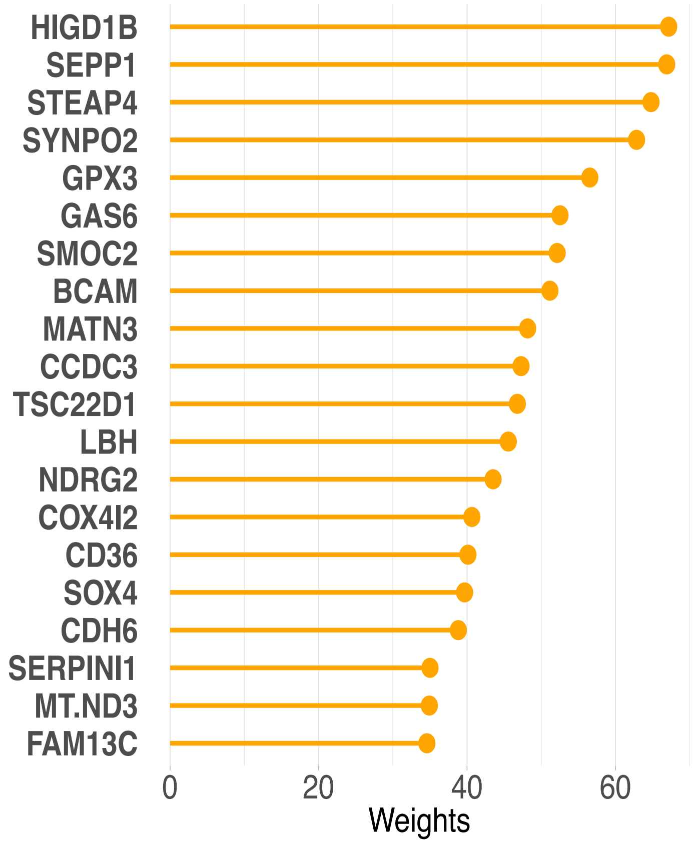

Fib GEM 56

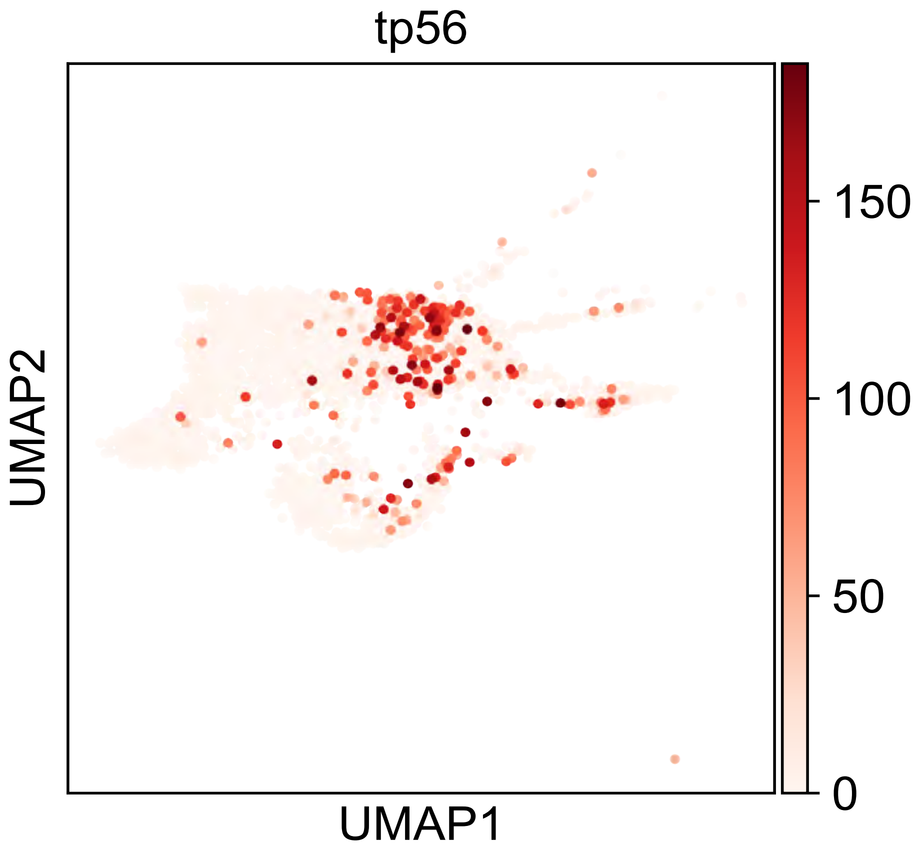

Cell Type

Fib_1

Fib_4

Top 20 genes

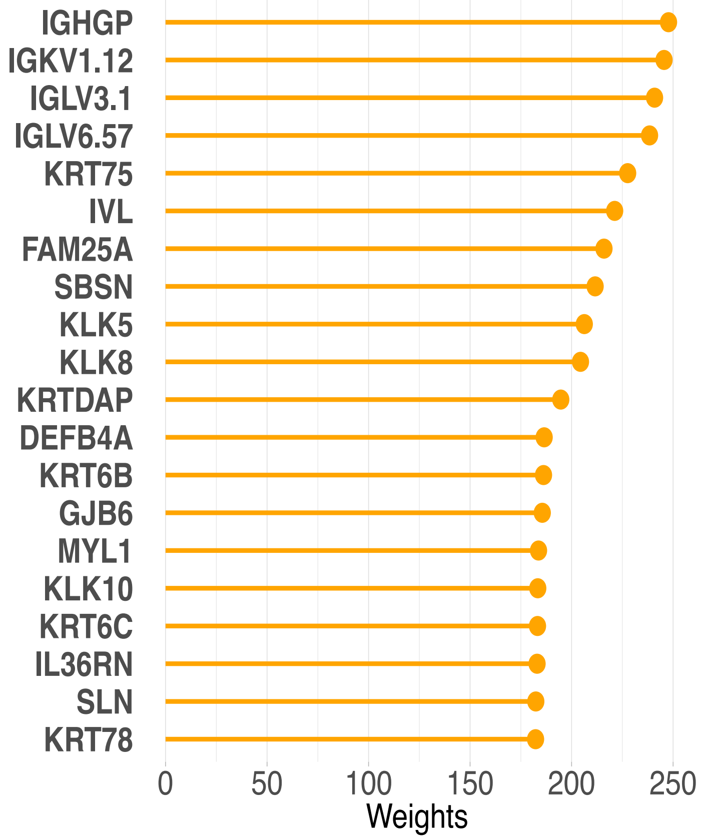

Supplement: S2 Fig — (PDF) [file pcbi.1010761.s002.pdf]
