# Supplementary material for: An individualized causal framework for learning intercellular communication networks that define microenvironments of individual tumors
Source: PLoS Comput Biol. 2022 Dec 22;18(12):e1010761. doi: 10.1371/journal.pcbi.1010761 (PMC9822106; doi:10.1371/journal.pcbi.1010761)
Supplement: Attachment — Submitted filename: PCOMPBIOL-D-22-00679_submitted_review.docx [file pcbi.1010761.s018.docx]

***Manuscript Overview***

The manuscript presents an advanced analytical workflow aimed at the elucidation of cell-cell communication, and its application to the analysis of multiple single cell and bulk RNAseq Head and Neck cancer datasets. In particular, the analyses:

- Identified gene expression modules (GEMs) across cell types using an NHDP model (nested hierarchical Dirichlet processes) in scRNAseq of blood and tumors.
- Deconvoluted TCGA HNSCC by computing GSVA scores for each GEM in each sample and found that the samples clustered into 4 groups with distinct immune environments, that were associated with survival status.
- Looked at pairwise correlation of epithelial, immune, endothelial and fibroblast GEMs. Found correlation across GEM type, indicating intercellular communication. Could predict immune GEM status based on other GEMS in population.
- Applied a Greedy fast casual inference (GFCI) algorithm using GSVA scores as input, which yielded a partial ancestor graph (PAG) reflecting putative causal relationships represented as directed edges. GCFI was applied to HNSCC TCGA data, and intercellular communications were inferred from the PAG.
- Applied an individualized causal discovery algorithm (iGFCI) to produce tumor-specific ICN for 522 TCGA tumors. Clustered networks and found 4 clusters of networks, each exhibiting different survival outcomes.

***Overall Assessment***

The manuscript is well written, and the approaches appear sound. Of particular significance, the study applies advanced causal inference techniques (some developed by the authors themselves, who are among the leading experts in the field) to identify putative causal relationships between cell types and states, and use the inferred individualized intercellular communication networks (ICNs) to cluster the tumors into groups with significantly different prognosis.

The proposed approach to infer cell-cell communication is unique: it does not identify specific L-R pairs, but it can identify new cell cell communications without using known information from databases (non-knowledge-based approach), which is a novel approach and is an important contribution to the field. The PAGs produced in the analysis provide a framework to infer biological activity related to communication events taking place within a tumor.

Some reservations are here raised about the overall scope of the manuscript, whether it should be categorized as a “method” or “biomedical research” study, and how the results should be interpreted.

As a “bio research” paper, an adequate biological interpretation of the findings and the functional validation are missing. As a “method” paper, the manuscript presents at least three (partially) novel approaches: a clustering algorithm (based on NHDP), a causal inference approach based on module projected data, and a further instance-specific causal inference approach applied to the same data. It then applies these advanced approaches to the analysis of HNSC and generates a lot of results, which are only very marginally discussed and interpreted. As a method paper, the evaluation of the methods is somewhat inadequate (e.g., the clustering algorithm should be extensively evaluated on simulated and/or real data, and possibly compared to other methods, see some references below. The causal inference approaches should possibly be applied to one or more datasets with known “ground truths” and evaluate whether those can be rediscovered).

The authors should choose which way to go (I think a method paper is the best fit), and then extend their evaluation to satisfy their choice criteria. I frankly think there is enough for multiple methods paper here, but additional evaluation would be required for each.

***Specific comments***

1) There is no reference to the scRNAseq dataset analyzed. Is this a new dataset? Or previously published? If the former, please provide GSE #, if the latter, please provide reference.
2) Figure S3 is missing
3) The “mathematical test[ing] of the learned ICN” is unclear. In particular, it is not clear whether a train-test procedure was applied (where the ICN and the Markov Blanket-based regression models are learned on the train set, and the R2 reported are based on the test set), or both training (of ICN) and testing (of the regression models) were performed on the same data. If the latter, clearly the results are less convincing. This would still be an acceptable form of evaluation (especially in light of the difficulty of repeating the ICN inference multiple times), but it should be clarified, and the caveats acknowledged.
4) The authors should provide more specific description and discussions of the main interacting ICNs in the context of known communication within literature, i.e., do they identify interactions already known to occur, and what novel communications are identified? They do this once at the top of page 14, when they point out Treg/DC interaction and two CD8 T cell subtypes involved in immune surveillance. They should extend it.
5) The authors describe two clustering results (the one based on GEMs and the one based on ICNs), both yielding 4 groups. What is the relationship between the two? How should they be used?
6) Overall, the authors adequately reference relevant literature.
   a) On page 4, they say “Particularly, a new family of individualized (or instance-specific) causal discovery methods has been developed, which can learn a causal network based on the data of an individual case, be it a single cell or a single tumor.” They could elaborate on existing applications of causal discovery and limitations of its use in this application in the discussion section. (Although they do go into great detail on the method in the methods section).
   b) The NHDP-based method described has some similarities to CELDA (https://www.camplab.net/celda/) and to K2Taxonomer (PMID: 34226941). It shares with the former some modeling aspects (Celda implements a Bayesian hierarchical model based the latent Dirichlet allocation), and with the latter the hierarchical decomposition and its use of different sets of markers at different splits in the hierarchy (“Unlike conventional clustering analysis based on overall transcriptomics, our approach clearly delineated the transcriptomic processes that contributed to such development.”), and the subsequent use of tree node signatures (similar to the GEMs?) to perform GSVA-based projection in bulk data. This does not detract from the approach presented here (I believe the method is different enough to remain significant) but differences and similarities should be discussed/acknowledged.
7) The NDHP-based method is very scantily described. On the other hand, the authors reference their previously developed methods for GFCI and iGFCI in references 80 and 81 and explain them in great and adequate detail in the methods. However, I do not see any code provided. I believe it is not required, but it would definitely be helpful. At the very least, they should provide a github repository with the scripts used for the analysis.
