# Supplementary material for: An individualized causal framework for learning intercellular communication networks that define microenvironments of individual tumors
Source: PLoS Comput Biol. 2022 Dec 22;18(12):e1010761. doi: 10.1371/journal.pcbi.1010761 (PMC9822106; doi:10.1371/journal.pcbi.1010761)
Supplement: Attachment — Submitted filename: Point-to-point response to review final.docx [file pcbi.1010761.s019.docx]

We appreciate the constructive critiques provided by the reviewers, and we would like to thank them for their time and suggestions.

Below is a point-by-point response to the reviewers’ comments and suggestions. We used text in blue Times font to indicate our response.

Reviewer's Responses to Questions

**Comments to the Authors:
Please note here if the review is uploaded as an attachment.**

Reviewer #1: The authors discuss how cellular states of cells in TMEs are coordinated through intercellular communication networks (ICNs) that enable multi-way communications among epithelial, fibroblast, endothelial, and immune cells. They also present that the capability of estimating individual ICNs reveals heterogeneity of ICNs and importance of intercellular communication in disease progression. The authors claim that the analyses of individual ICNs revealed structural patterns that were shared across subsets of tumors led to discovery of four different subtypes of tumor microenvironment (TME) networks of head and neck squamous cell carcinoma (HNSCC). The authors cleverly used the nested hierarchical Dirichlet processes model to the scRNAseq data to identify gene expression modules (GEMs) and employed gene set variation analysis to determine the expression status of the GEM in each tumor.

One major concern I have is how the authors claim that the patients with distinct TMEs exhibited significantly different clinical outcomes however the realistic clinical outcomes are not discussed. Below, I have listed a few questions and comments for the authors to address.


Major comments:

Although it is understood that the authors aimed to study the underlying TME associated events that are present in HNSCC (and as such can be detected with single cell analyses), the study design did not incorporate any commonly cogitated in clinic subtypes related to survival/response, i.e., subtypes classified by HPV status (cite: PMID: 34072836 or other bibliography).

1) Given that current challenges in the treatment of HNSCC include especially the HPV- patients, it would be advantageous to find out how the classes identified here (especially in relationship to the causal networks and immune response components) correspond to the HPV+ (with majority of patients responding well to treatment) vs. HPV- (patients will not do well).

Thanks for the suggestion. We have included tumor HPV status in Figure 3 and Figure 5, which illustrate the cancer microenvironment subtypes derived from GEM expression and tumor-specific ICNs. The results show that HPV+ tumors were enriched in a patient cluster identified based on GEMs and ICNs. We also added descriptions of the findings on pp 10 & 16.

2) By mining the patterns of ICNs of individual tumors, authors discovered distinct patterns of ICNs under TMEs of HNSCCs and claimed they were associated with significantly different patient outcomes. However, authors do not specify clinical details and known subtypes. Since the authors identified 39 immune GEMs and their signature genes, such analysis could benefit patients who would potentially respond to immune therapy and the value to this manuscript/work.

Thanks for the suggestion. We identified a recent publication on studying biomarkers for predicting the PD-1 checkpoint inhibitor in HNSCC (Obradovic, et al. "Immunostimulatory cancer-associated fibroblast subpopulations can predict immunotherapy response in head and neck cancer." Clinical Cancer Research 28.10 (2022): 2094-2109). Indeed, we found that certain immune GEMs and stromal GEMs are informative in predicting immune therapy response. We have added a brief subsection reporting these proof-of-concept results. Since the study has a small sample size, we did not elaborate too much regarding these results, and we are in the process of collecting more cases to perform a more comprehensive study and will report the results in a separate manuscript.

Minor comments:

Avoid jargon such as e.g., ‘paper’.

We replaced ‘paper’ with ‘article.’

**Reviewer #2:**

The manuscript presents an advanced analytical workflow aimed at the elucidation of cell-cell communication, and its application to the analysis of multiple single cell and bulk RNAseq Head and Neck cancer datasets. In particular, the analyses:

- Identified gene expression modules (GEMs) across cell types using an NHDP model (nested hierarchical Dirichlet processes) in scRNAseq of blood and tumors.
- Deconvoluted TCGA HNSCC by computing GSVA scores for each GEM in each sample and found that the samples clustered into 4 groups with distinct immune environments, that were associated with survival status.
- Looked at pairwise correlation of epithelial, immune, endothelial and fibroblast GEMs. Found correlation across GEM type, indicating intercellular communication. Could predict immune GEM status based on other GEMS in population.
- Applied a Greedy fast casual inference (GFCI) algorithm using GSVA scores as input, which yielded a partial ancestor graph (PAG) reflecting putative causal relationships represented as directed edges. GCFI was applied to HNSCC TCGA data, and intercellular communications were inferred from the PAG.
- Applied an individualized causal discovery algorithm (iGFCI) to produce tumor-specific ICN for 522 TCGA tumors. Clustered networks and found 4 clusters of networks, each exhibiting different survival outcomes.

***Overall Assessment***

The manuscript is well written, and the approaches appear sound. Of particular significance, the study applies advanced causal inference techniques (some developed by the authors themselves, who are among the leading experts in the field) to identify putative causal relationships between cell types and states, and use the inferred individualized intercellular communication networks (ICNs) to cluster the tumors into groups with significantly different prognosis.

The proposed approach to infer cell-cell communication is unique: it does not identify specific L-R pairs, but it can identify new cell cell communications without using known information from databases (non-knowledge-based approach), which is a novel approach and is an important contribution to the field. The PAGs produced in the analysis provide a framework to infer biological activity related to communication events taking place within a tumor.

Some reservations are here raised about the overall scope of the manuscript, whether it should be categorized as a “method” or “biomedical research” study, and how the results should be interpreted.

As a “bio research” paper, an adequate biological interpretation of the findings and the functional validation are missing. As a “method” paper, the manuscript presents at least three (partially) novel approaches: a clustering algorithm (based on NHDP), a causal inference approach based on module projected data, and a further instance-specific causal inference approach applied to the same data. It then applies these advanced approaches to the analysis of HNSC and generates a lot of results, which are only very marginally discussed and interpreted. As a method paper, the evaluation of the methods is somewhat inadequate (e.g., the clustering algorithm should be extensively evaluated on simulated and/or real data, and possibly compared to other methods, see some references below. The causal inference approaches should possibly be applied to one or more datasets with known “ground truths” and evaluate whether those can be rediscovered).

The authors should choose which way to go (I think a method paper is the best fit), and then extend their evaluation to satisfy their choice criteria. I frankly think there is enough for multiple methods paper here, but additional evaluation would be required for each.

We view our manuscript as a methodology paper introducing the framework of modeling intercellular communication as causal networks.

The simulation experiments examining the capability of iGFCI in discovering instance-specific networks were performed in the original GFCI [article](https://scholar.google.com/citations?view_op=view_citation&hl=en&user=QiEizloAAAAJ&citation_for_view=QiEizloAAAAJ:eQOLeE2rZwMC) that is cited in the methods section of the current paper.

In terms of evaluating the results of causal inference using other datasets with “known ground truths”, we would like to point out that our algorithms infer the causal relationships between latent signaling processes (represented by expression status of GEMs) across cell boundary as shown in Fig M1. At the current stage, it is difficult to map the latent signals (*X* and *Y*) to real biological entities, and thus it is difficult to evaluate the inferred causal relationship between *X* and *Y* using established biological “ground truths.” Furthermore, to our knowledge, this is the first attempt to use this framework to model cell-cell communication; thus, we could not compare our methods to other computational methods performing similar tasks on our data.

***Specific comments***

1) There is no reference to the scRNAseq dataset analyzed. Is this a new dataset? Or previously published? If the former, please provide GSE #, if the latter, please provide reference.

We provided information regarding the data availability, which is deposited at the GEO data base with an accession number of [GSE164690](https://www.ncbi.nlm.nih.gov/geo/query/acc.cgi?acc=GSE164690)

2) Figure S3 is missing

The figure was present in previous submission but mistakenly labeled in its legend, which is corrected now.

3) The “mathematical test[ing] of the learned ICN” is unclear. In particular, it is not clear whether a train-test procedure was applied (where the ICN and the Markov Blanket-based regression models are learned on the train set, and the R2 reported are based on the test set), or both training (of ICN) and testing (of the regression models) were performed on the same data. If the latter, clearly the results are less convincing. This would still be an acceptable form of evaluation (especially in light of the difficulty of repeating the ICN inference multiple times), but it should be clarified, and the caveats acknowledged.

The results were from a 10-fold train-test experiments. We clarified this point in the Results (pp 14) and Methods (pp 21) sections.

4) The authors should provide more specific description and discussions of the main interacting ICNs in the context of known communication within literature, i.e., do they identify interactions already known to occur, and what novel communications are identified? They do this once at the top of page 14, when they point out Treg/DC interaction and two CD8 T cell subtypes involved in immune surveillance. They should extend it.

We have added more discussion of the inferred communications relative to current knowledge, such as enrichment of HPV+ cases in a subtyped discovered by clustering tumors based on ICNs (pp 16). We also added a section describing the value of GEMs in predicting patient responses to immune therapy.

5) The authors describe two clustering results (the one based on GEMs and the one based on ICNs), both yielding 4 groups. What is the relationship between the two? How should they be used?

The results of comparing relationship of clustering results using the two methods were presented as Figure S7. We also added more information (chi-squared test) about this comparison (pp 15).

Overall, the clustering based on GEMs and based on ICNs agree with each other, which supports the notion that ICNs underlie the GEM profiles of tumors.

6) Overall, the authors adequately reference relevant literature.
   a) On page 4, they say “Particularly, a new family of individualized (or instance-specific) causal discovery methods has been developed, which can learn a causal network based on the data of an individual case, be it a single cell or a single tumor.” They could elaborate on existing applications of causal discovery and limitations of its use in this application in the discussion section. (Although they do go into great detail on the method in the methods section).

We have added more references and discussed the assumptions and limitations of our methods (pp 17, 30, and 35)

b) The NHDP-based method described has some similarities to CELDA (https://www.camplab.net/celda/) and to K2Taxonomer (PMID: 34226941). It shares with the former some modeling aspects (Celda implements a Bayesian hierarchical model based the latent Dirichlet allocation), and with the latter the hierarchical decomposition and its use of different sets of markers at different splits in the hierarchy (“Unlike conventional clustering analysis based on overall transcriptomics, our approach clearly delineated the transcriptomic processes that contributed to such development.”), and the subsequent use of tree node signatures (similar to the GEMs?) to perform GSVA-based projection in bulk data. This does not detract from the approach presented here (I believe the method is different enough to remain significant) but differences and similarities should be discussed/acknowledged.

Thanks for pointing out the references. We have cited the references (pp 18) and discussed the differences from the methods.

We applied CELDA to our dataset. As shown in the figure below, many topics learned by CELDA tend to overlap on the UMAP of single cells. This is due to the fact that many house-keeping genes are distributed (“bleed over”) to different topics, a common short coming of non-hierarchical topic models. The NHDP model (Fig S2) uses topics close to root of the tree to capture house-keeping genes expressed by most cells, such that distal topics can capture topics that more likely reflect specific transcriptomic processes.

7) The NDHP-based method is very scantily described. On the other hand, the authors reference their previously developed methods for GFCI and iGFCI in references 80 and 81 and explain them in great and adequate detail in the methods. However, I do not see any code provided. I believe it is not required, but it would definitely be helpful. At the very least, they should provide a github repository with the scripts used for the analysis.

We added addition descriptions of the characteristics of NHDP (pp 18)

The code for GFCI and iGFCI are available at the Center for Causal Discovery websites, and links are provided (pp 1).

Reviewer #3: In this work, Lu et al. develop a framework to individualized causal networks (ICNs) that describe intercellular communication relationships from single-cell RNA-sequencing data (scRNA-seq) sampled from patient tumors. The authors collected scRNA-seq data from 18 head and neck squamous cell carcinoma (HNSCC) patients. After identifying present cell types, the authors infer cell-type-specific gene expression modules (GEMs), sets of co-expressed genes that characterize cell state. From these GEMs, the authors use a variant of the famous Fast Causal Inference method, dubbed individualized Greedy Fast Causal Inference (iGFCI) to infer the ICNs, using Gene Structural Variant Analysis (GSVA) scores derived by deconvolving bulk RNA-seq data as proxy variables for GEMs. Both a global ICN for HNSCC and individualized ICNS are inferred, where the latter are inferred from 522 HNSCC tumors from the TGCA. Causal edges and trends in ICNS across patient groups are then analyzed.

While I think the ideas presented in this work are interesting and this work is a welcome step towards applying causal discovery methods to single-cell transcriptomics data, I think the results are difficult to interpret and several claims need to be validated in more detail. Therefore, I can only recommend publication after a major revision.

In particular, I would like the following comments to be addressed:
- The work would benefit from a more thorough comparison between the insights derived from analysis of patient-specific ICNs vs. the global ICN obtained for HSNCC. That is, what is gained or lost, in terms of biological insight, from analyzing only individual ICNs or analyzing only the global, “aggregated” ICN? Or, for example, could the individualized networks be used as additional prior knowledge when inferring the global network, or vice versa?

The percentage of conserved edges between global and iGFCI is 106. 106/524 for iGFCI and 106/493 for global.

We added this information in main text (pp 14-15).

- Given that the inferred GEMs appear to vary with cell type, what is the difference between a GEM and, say, the set of marker genes inferred by differential expression analysis? From looking at the supplementary information, is it not immediately clear how a GEM differs from a set of differentially expressed genes.

In Figure S2, we show the combined expression values of the member genes of a GEM in each cell. A GEM consists of a set of genes that tend to co-express in a subpopulation of cells. If one can identify such a subpopulation (e.g., through clustering) and compare them to remaining cells, the expression value of the GEM (combining expression of member genes) is likely to be statistically different from those in other cells. However, it is not necessarily always true that expression value of an individual member gene is differentially expressed between the two populations.

Often, we can see that a GEM is expressed in cells across the boundaries of cell clusters detected by clustering analysis at a given setting. To detect a GEM through differential expression analysis is difficult because it requires first identifying the subpopulation of cells expressing a set of genes not expressed in other clusters and then performing differential expression analyses.

- Did the authors verify whether or not all GEMs used in ICN inference actually contain cell signaling ligands? Otherwise, it may not be possible to claim that GEM_A -> GEM_B implies cell-cell communication. For example, at first glance, the top 20 genes of Imm GEM 1–5 do not contain signal ligands.

Indeed, GEMs 1 – 5 capture genes that are commonly expressed in a large subpopulation, aka, “house-keeping” genes of major lineages. For example, Imm GEM1 is expressed in all T cells, B cells, and NK, while Imm GEM2 covers all myeloid, dendritic cells, and monocytes. This is the strength of NHDP in that it organizes gene into a hierarchy based on breadth of the expression, which allows it to capture generally expressed genes using topics close to the root and then to use distal topics to capture genes expressed in highly specialized cells. Other non-hierarchical topic models, likely the CELDA shown on page 6 of this letter, would distribute genes expressed in large number of cells to multiple topics (“bleed over”), which makes topic less specific and thereby less biologically meaningful.

- If there are signal ligands, why not try use cell-cell communication inference tools (e.g., CellChat) for an analysis? At minimal, the authors should discuss the possibility of using such tools and their potential outcomes.

Based on the suggestion by this reviewer, we included a supplementary table summarizing the ligands/receptors included the top 50 genes of each GEM (now Table S2).

We did not report any results of using CellChat and comparing them to our results because the problem settings are quite different. Firstly, CellChat is based on a procedure of first clustering results and then assessing whether two clusters tend to express a ligand-receptor pair respectively that likely interact. As we mentioned before, the distribution of GEMs does not always align well with clustering methods. Secondly, expression of a ligand and receptor in two clusters of cells does not necessary entail physical interaction between them in TME. Finally, CellChat cannot detect the cellular signals in the cells of two clusters, thus it is unclear what signal a pair of ligand receptor transmits.

To overcome the above challenges encountered by CellChat or similar methods, we are developing rigorous causal models to search for ligand-receptor pairs as shown in the following model.

*V*

*X*

*Y*

*GEM-X*

*GEM-Y*

*W*

*V*

*GEM-Y*

*GEM-X*

*W*

*LR*

*LR*

Here we can search for ligand-receptor pairs as an integrated component of search causal chain between two GEMs across cell boundary. This requires a substantial amount of work and analyses, and we will publish the results in a separate manuscript.

- The authors make the good point that current methods to infer cell-cell communication from scRNA-seq can only infer interactions that are known with respect to literature knowledge. Did the ICNs yield any novel insight, or did they confirm what was already known?

We appreciate the comment. ICN analysis did generate novel hypotheses. However, it is difficult for us to experimentally validate them, because as a dry lab, we do not have resources to carry out wet-lab experiments. Furthermore, as pointed out by our ICN analysis, certain causal edges only exist in a specific subtype of tumors, which requires one to first identify such tumors and then perform experiments at the tumor level (not at cell line level), which is quite challenging.

- It appears that the scRNA-seq is used to infer cell-type-specific GEMs, which is then used to deconvolve bulk RNA-seq data. The ICNs are then inferred using the deconvolved bulk RNA-seq data. Did the authors infer ICNs directly from the scRNA-seq data as well? Are there substantial differences between ICNs obtained from scRNA-seq data vs. ICNs obtained from bulk data?

This is an intriguing suggestion, which will be followed up in our future studies. When we perform bulk RNA analysis, our hypothesis is that if a sufficient number of cells from two subpopulations (e.g., Tregs expressing ImmGEM16 and dendritic cells expressing ImmGEM19) are interacting and influencing each other in multiple tumors, our causal discovery algorithms can detect the signal.

It is difficult to use scRNA derived from an individual or a collection of tumors to detect causal relationships, because current scRNA data are collected from cells in suspension, and we do not know which cells are within the vicinity of other cells allowing them to interact. The lack of spatial information makes it difficult to infer causal relationships at the single cell level, i.e., given transcriptomes of a pair of Cell_A and Cell_B, determining whether signals within the cells causally influence each other.

However, this problem can be addressed in the spatial transcriptome setting, where we can model cell-cell communication among cells within a spatial vicinity. This is an ongoing work in our group.

- One claim is that testing if GEM_A -> GEM_B is equivalent to testing the relationship between two proxy variables, e.g. GSVA_A -> GSVA_B. The implication then is that these proxy variables are in one-to-one relationship with the GEMs. Did the authors check this?

The original paper introducing GSVA has performed extensive evaluation demonstrating it can capture the **variance of a gene set** (not an individual gene in the set) among samples.

Per this comment, we performed a simulation study using another larger scRNA sample (for more reliable estimation of correlations), in which we pooled the data of scRNA from a sample to create a “bulk” RNA sample. We then applied GSVA methods to such samples to infer the expression status of a GEM in tumors. We then regressed the GSVA score with the sum of top 50 genes of a GEM among the single cells expressing the GEM in samples and found that the correlation coefficients for most of GEMs are significant. The figure below shows the scatter plot of GSVA score from pseudo-bulk RNA and pooled GEM expression values inferred by NHDP in tumors (T) and adjacent normal tissues (N).

**Pooled GEM scRNA exprs**

- There’s an overall lack of interpretability behind the results presented in Figure 1¬–4. That is, while networks between GEMs are presented, there is a lack of discussion of what key genes are contained in these GEMs and what biological conclusions can be drawn. It would strengthen results to move some of the information from the Supplementary Figures.

As discussed in the response to the general comments by Reviewer #2 (pp 4), we view our manuscript as a Methodology article in which our method can be used as a tool to generate hypotheses regarding intercellular communication, rather than a “bio research” article reporting and discussing discoveries. In our response to Reviewer #2, we also mention the difficulty of interpreting causal relationships between latent variables, whose biological entities are unknown. As such, there is little, if any, “ground truth” knowledge available to interpret the causal edges discovered in this study.

We believe some of the hypotheses generated by our method are biologically sensible, even though we cannot interpret individual causal edges from the conventional biological point of view. For example, certain GEMs and causal edges are strongly associated with patient survival beyond random chance, indicating the GEMs and edges are associated with biological signals. Another example is that certain ICN edges are conserved in hundreds of tumors that are associated with HPV infection. These results indicate that collectively our results demonstrate the potential biological impact of the intercellular communication, although it is difficult to interpret individual edges at this time.

Finally, as mentioned above, we are planning to search for ligand-receptor pairs that likely mediate the signal transduction of a causal edge, which will significantly enhance the interpretability of our results. We will report the results in a separate manuscript.

- In general, the Figures could be improved by using text labels with larger font sizes, plot titles, and using vector graphics instead of PNG/JPEG files.

We have followed the standard of most journal regarding the font size. We find that sometimes vector plots carry all the data points, which inflate the file size.

In this revision, we embed pdf figures in the submission. We will use high quality JPEG or vector plots per journal production guidelines.

- In Figure 4, including color legends would really help a lot with interpretability of networks.

We used the prefix (imm, endo, etc) to indicate the cell type, which function as color legends.

**Have the authors made all data and (if applicable) computational code underlying the findings in their manuscript fully available?**
The [PLOS Data policy](https://nam12.safelinks.protection.outlook.com/?url=https%3A%2F%2Fjournals.plos.org%2Fploscompbiol%2Fs%2Fmaterials-and-software-sharing&data=05%7C01%7Cxinghua%40pitt.edu%7C210550a5a69c428ca6ce08da6ff6189f%7C9ef9f489e0a04eeb87cc3a526112fd0d%7C1%7C0%7C637945400169412694%7CUnknown%7CTWFpbGZsb3d8eyJWIjoiMC4wLjAwMDAiLCJQIjoiV2luMzIiLCJBTiI6Ik1haWwiLCJXVCI6Mn0%3D%7C3000%7C%7C%7C&sdata=Vhrc9rQ6G%2BtorFubqzAllpIeDXAc65tXzomflTmrKk4%3D&reserved=0) requires authors to make all data and code underlying the findings described in their manuscript fully available without restriction, with rare exception (please refer to the Data Availability Statement in the manuscript PDF file). The data and code should be provided as part of the manuscript or its supporting information, or deposited to a public repository. For example, in addition to summary statistics, the data points behind means, medians and variance measures should be available. If there are restrictions on publicly sharing data or code —e.g. participant privacy or use of data from a third party—those must be specified.

Reviewer #1: **No:** Authors specify the data source. They don't specify the code(s) availability.

Reviewer #2: **No:** I do not see reference to the scRNAseq data analyzed (GSE or SRA #)

Reviewer #3: None

In this revision, we have included the sources:

The code for iGFCI: [https://github.com/fattaneh/tetrad-IGFci](https://nam12.safelinks.protection.outlook.com/?url=https%3A%2F%2Fgithub.com%2Ffattaneh%2Ftetrad-IGFci&data=05%7C01%7Cxinghua%40pitt.edu%7C7eeece1bf56346b6b96608da8db85e9f%7C9ef9f489e0a04eeb87cc3a526112fd0d%7C1%7C0%7C637978120253499609%7CUnknown%7CTWFpbGZsb3d8eyJWIjoiMC4wLjAwMDAiLCJQIjoiV2luMzIiLCJBTiI6Ik1haWwiLCJXVCI6Mn0%3D%7C3000%7C%7C%7C&sdata=3p7KuP6Ko3zsNS%2FbLub%2B%2Bxsu4s3jf1MfzYkKDJeFCzM%3D&reserved=0)

The code for NHDP is available from Dr. John Paisley’s website: <http://www.columbia.edu/~jwp2128/code/nHDP.zip>.

The scRNA data is available from our previous publication with a GEO access number of [GSE164690](https://www.ncbi.nlm.nih.gov/geo/query/acc.cgi?acc=GSE164690)
