# Supplementary material for: An individualized causal framework for learning intercellular communication networks that define microenvironments of individual tumors
Source: PLoS Comput Biol. 2022 Dec 22;18(12):e1010761. doi: 10.1371/journal.pcbi.1010761 (PMC9822106; doi:10.1371/journal.pcbi.1010761)
Supplement: Attachment — Submitted filename: authors_reply_letter.docx [file pcbi.1010761.s020.docx]

Dear Editor,

We would like to thank the reviewers for the positive and constructive comments. We have addressed the minor concerns from Reviewer #2 and #3. We have modified the manuscript and provided the following response to the comments and suggestions (shown as blue font)

Reviewer #1: Thanks to the Authors for working on the revisions; the article is solid and ready for publication.

Reviewer #2: The authors addressed most of the criticisms. However, some minor concerns remain.

Some of the authors’ responses refer to changes and additions made in the revised version, but those were difficult to identify, and sometime were not to be found at all.

The major ones are listed below:

• In response to point (3), the authors state: “The results were from a 10-fold train-test experiments. We clarified this point in the Results (pp 14) and Methods (pp 21) sections.” I found the reference to 10-fold CV on pp. 14 and 21, but didn’t notice any acknowledgement of the the procedure’s caveat. The main criticism was that using the same model (i.e., the network structure learned on all data) is the main source of “information leakage” here when predicting a GEM from others (akin to performing feature selection outside the CV loop, which can significantly inflate accuracy), and this point is not addressed.

Authors’ reply: Thanks for pointing out the potential caveat. We thus use an independent cohort of HNSC samples as a test set for the prediction model. The independent cohort was not included in the training of NHDP process nor in the process of learning CBN. Thus, it is unlikely that “information leakage” occurs in this setting. We reported the details on page 16.

• In response to point (6), the authors state: “Thanks for pointing out the references. We have cited the references (pp 18) and discussed the differences from the methods.” However, if I look at p. 18 (or elsewhere), I couldn’t find the “cited references” nor a “discuss[ion of the] differences”.
The comparison to Celda should not be for this reviewer only. Rather, if this is a method paper, comparison of NHDP to other methods should be explicit part of the evaluation (given the manuscript is already pretty long, perhaps including this comparison in the supplementary document would be ok). The expanded description of NHDP does indeed help (although it remains scant for a method paper), but if the method is presented as a way of capturing “hierarchical” (lineage-like) structures, then comparison to (or at least discussion of) other methods aiming to do the same (e.g., K2Taxonomer, and trajectory-based methods such as Monocle, PAGA, etc.), and conjectures about why the new method should be preferable, should be included.

Thanks for the suggestion. We applied Celda on our CD45+ immune cells to search equal number of topics as we did in our study. We then examined and compared the GEMs returned by the Celda and NHDP. The hierarchical structure mentioned by Celda represents the biological hierarchies of cell organization, i.e., multiple cell types define tissues, different type of cells within a tissue, different GEMs within a cell, and finally a set of genes within a GEM. While Celda and NHDP are conceptually similar in defining GEMs within a cell and genes within a GEM, they differ in that presence/absence of GEMs in a cell is deemed to be independent in Celda. On the other hand, NHDP captures the tendency of co-occurrence of GEMs within a cell using a topic tree. This difference sufficiently leads to significantly different results. We show the results of Celda as Figure S3, and briefly discuss the difference in the main text (page 8).

Reviewer #3: I am satisfied with the new manuscript and think the authors have done a good job in responding to all reviewer comments and making the paper clearer in its focus and potential biological applications. I therefore recommend that the paper be published now, with some additional minor comments that can be fixed easily.

Some minor comments:
- Please make sure the formatting of spacing, particularly between references and citations is correct, e.g. on page 4, ‘communications[1-6]’, and page 6, ‘(NHDP)[33]’, but also ‘top30’ on page 38
- Please check consistency in punctuation, e.g. ‘Almet et al. [25]’ vs ‘Armingol et al [26]’ on page 4 and ‘Obradovic et al [39]’ on page 12
- Figure 4c caption; as the subgraph contains only direct causal edges, I think you can remove the part about how to interpret blunt tailed edges.

Authors’ reply: thanks for pointing out the formatting issues. We have corrected the formatting issues.
